# Supplementary material for: Stable salts of the hexacarbonyl chromium(I) cation and its pentacarbonyl-nitrosyl chromium(I) analogue
Source: Nat Commun. 2019 Feb 7;10:624. doi: 10.1038/s41467-019-08517-2 (PMC6367395; doi:10.1038/s41467-019-08517-2)
Supplement: Supplementary file 1 — Supplementary Information [file 41467_2019_8517_MOESM1_ESM.pdf]

Supplementary Information for

Stable Salts of the Hexacarbonyl Chromium(I) Cation and its Pentacarbonyl-Nitrosyl Chromium(I) Analogue

Bohnenberger et al.

Table of Contents

1. Supplementary Methods 3
2. Experimental glassware and pictures of complexes 1 and 2 7
3. Synthesis of NO[F-{Al(OR^F)$_3$}$_2$] 9
4. Detailed Synthesis and Characterization of compounds 1–4 12
5. NMR Spectra 16
6. Evans NMR Method 26
7. Vibrational Analysis 30
8. Gas Phase IR Spectra 34
9. EPR Spectroscopy 35
10. IR and Raman Spectra 41
11. UV/Vis Spectra 47
12. Elemental Analyses 48
13. Powder XRD Data and Rietveld Refinement 49
14. Single-Crystal XRD Data 59
15. Hirshfeld Plots 81
16. Solution Thermodynamics Calculations 82
17. Details on the gase phase energetics and DFT calculations 83
18. Additional information on the DFT calculations 84

1. Supplementary Methods

General Conditions. All manipulations on substrates and products were undertaken in a MBraun glovebox filled with Ar (O_2, H_2O < 1 ppm). All experiments were carried out in special double-Schlenk tubes[1] (Supplementary Figure 1) separated by a G3 or G4 frit with grease-free PTFE or glass valves in an inert atmosphere using vacuum and standard Schlenk techniques. Solvents were dried by standard methods using CaH_2 or P_4O_{10} and distilled prior to use. $Cr(CO)_6$ (VWR/Merck) was purified by sublimation before use. $NO[Al(OR^F)_4]$ $\{R^F = C(CF_3)_3\}$ was synthesized according to literature[2]. $NO[F\text{-}\{Al(OR^F)_3\}_2]$ $\{R^F = C(CF_3)_3\}$ was prepared from $NO[PF_6]$ (Acros) and $Me_3Si\text{-}F\text{-}Al(OR^F)_3$[3] (see Experimental Section for a detailed synthesis).

NMR Spectroscopy. NMR samples were prepared in 5 mm thick walled NMR tubes with J. Young valves. The ^1H, $^{13}C\{^1H\}$, ^{19}F and ^{27}Al spectra were recorded either on a Bruker Avance II+ 400 MHz, on a Bruker Avance III HD 300 MHz or on a Bruker Avance 200 MHz spectrometer either in 1,2-$F_2C_6H_4$ (*ortho*-difluorobenzene, *o*DFB) or CD_2Cl_2 (0.4-0.6 mL) at r.t. Measurements conducted in 1,2-$F_2C_6H_4$ were calibrated by using the ^{19}F signal of the solvent 1,2-$F_2C_6H_4$ (δ = –139.0 ppm[4], rel. to CCl_3F). The field corrections of other nuclei were adjusted accordingly. Measurements conducted in CD_2Cl_2 were calibrated to the residual solvent signal for ^1H (δ = 5.32 ppm[5], rel. to TMS) and $^{13}C\{^1H\}$ (δ = 53.84 ppm[5], rel. to TMS). Measurements conducted in 1,2,3,4-$F_4C_6H_2$ were calibrated to the residual solvent signal for ^1H (δ = 7.04 ppm[6], rel. to TMS). The field corrections of other nuclei were adjusted accordingly. The Bruker Topspin software package (version 3.2) was used for measuring and processing of the spectra. Typical very small impurities were detected in the ^{19}F NMR at –74.8 ($HOC(CF_3)_3$) and –75.5 ppm. Paramagnetic shifts via Evans' method[7–9] were measured in a 1:6 solution of CD_2Cl_2/1,2-$F_2C_6H_4$ or CH_2Cl_2/1,2-$F_2C_6H_4$ respectively with the ^1H NMR shift of dichloromethane (δ = 5.32 ppm) as standard in an open 3 mm thick NMR tube inside the J. Young NMR tube containing the solution of the paramagnetic substance. All graphical representations were performed using Topspin (version 3.2).

Vibrational Spectroscopy. FTIR measurements were performed on a FTIR Bruker ALPHA with a QuickSnap Platinum ATR sampling module inside the glovebox. The data were processed with the Bruker OPUS 7.5 software package. Unless otherwise stated, the spectra were recorded in the range of 4000-550 cm^{-1} with a resolution of 2 cm^{-1} at r.t. and a base line correction with 3 iterations was applied. The gas phase IR spectra were measured on a Nicolet

Magna 760 IR spectrometer with a custom-made IR cell (length: 20 cm, diameter: 4 cm) with a J. Young valve (see the respective spectra for more apparative details). FT Raman spectra were recorded on a Bruker VERTEX 70 spectrometer equipped with a RAM II module (1064 nm exciting line of a NdYAG laser) by using a highly sensitive liquid N_2 cooled Ge detector. The samples were measured in flame sealed soda-lime glass Pasteur pipettes in the range of 4000-50 cm^{-1} with a resolution of 4 cm^{-1} at r.t. The data were processed with the Bruker OPUS 7.5 software package. Unless otherwise noted, the Raman spectra were cut off below 75 cm^{-1} and a baseline correction with 5 iterations was applied. All IR and Raman spectra were normalized to 1 and intensities are given as follows: vvw = very very weak (< 0.1), vw = very weak (< 0.2), w = weak (< 0.3), mw = medium weak (< 0.4), m = medium (< 0.5), ms = medium strong (< 0.6), s = strong (< 0.7), vs = very strong (< 0.8), vvs = very very strong (≥ 0.9). Extremely weak bands (< 0.025) are not reported. Graphical representations have been done with OPUS 7.5 or with OriginPro (version 9.2).

Single-Crystal X-ray Diffraction. Single crystals were selected at r.t. under perfluoropolyalkylether oil (AB128330, ABCR GmbH & Co. KG) on 0.1, 0.2 or 0.3 mm micromounts (M1-L19-100/200/300). Structural data were collected from shock-cooled crystals on a Bruker SMART APEX II Quazar CCD area detector diffractometer using a D8 goniometer with an Incoatec Mo-Microfocus Source IμS with mirror-monochromated Mo-K_α radiation (λ = 0.71073 Å) at 100(2) K. The diffractometer was equipped with an Oxford Cryosystem 800 low temperature device. The data were processed with APEX v2013.6-2, integrated with SAINT[10] (V8.37A) and an empirical absorption correction using SADABS 2014/5[11] or SADABS 2016/2[11] was applied. The structures were solved by direct methods using SHELXT[12,13]. Unless otherwise stated, all non-hydrogen atoms were refined anisotropically by full matrix least squares methods against weighted F^2 values based on all independent reflections by using SHELXL-2014/7[12,14] with ShelXle as GUI software[15]. Disordered fragments were modelled with the help of the DSR software[16]. The graphical presentation of crystal structures was prepared either with Mercury (version 3.9)[17] or with OLEX2 (version 1.2)[18]. CCDC codes 1844666 (compound **2**), 1844667 (**1**), 1844668 (**3**), 1844669 (**4**) contain the supplementary crystallographic data for this paper. These data can be obtained free of charge from The Cambridge Crystallographic Data Centre via: https://summary.ccdc.cam.ac.uk/structure-summary-form. Hirshfeld-surface plots were generated using the CrystalExplorer[19] software package with an Isovalue of 0.5.

Powder Diffraction. The powder diffractograms were recorded with the sample in a 0.5 mm thick capillary (Hilgenberg GmbH, wall thickness 0.01 mm) sealed with perfluoropolyalkylether oil (AB128330, ABCR GmbH & Co. KG), at about 100(10) K in the Θ range 3–42° with a STOE STADI P powder diffractometer with Mo-$K_{\alpha 1}$ radiation (λ = 0.709300 Å) equipped with Ge-(111) monochromator and Mythen 1K detector. Data acquiring, processing and the calculation of powder diffractograms from single-crystal data were performed using STOE WinXPOW® package. All powder diffractograms were background corrected.

UV/Vis spectroscopy. UV/Vis reflectance spectra were recorded at r.t. on a Thermoscientific Evolution 600 spectrometer equipped with an integration sphere. The baseline was measured against Spectralon®. To protect the powdered samples from air and moisture during measurements, a special sample holder with a quartz window was used.

EPR spectroscopy. EPR spectra were recorded using a Bruker EMXplus continuous wave (cw) X-Band spectrometer with nitrogen cooling for 100 K measurements and liquid helium cooling for 4 K measurements, respectively. Samples were filled into fused silica glass tubes. Solvents were vacuum transferred to the samples and afterwards the sample tubes were sealed under vacuum. EPR spectra were analysed and simulated using the EasySpin MATLAB toolbox[20,21].

Computational Details. Quantum chemical calculations were performed with the TURBOMOLE[22,23] program package (version 7.0). All investigated molecular structures were optimized at the density functional theory (DFT) and were run in redundant internal coordinates using the BP86[24,25] functional with the resolution-of-identity (RI) approximation[26,27] together with the basis set def2-TZVPP[28] and with dispersion correction (DFT-D3BJ)[29,30]. A fine integration grid (m4) and the default SCF convergence criteria (10^{-6} a.u.) were used. All optimized structures were checked for minima (no imaginary frequencies) with the implemented module AOFORCE[31] and for proper spin occupancies using the implemented module EIGER. Entropic contributions to enthalpy and Gibbs free energy with inclusion of zero point energies (ZPE) were calculated at the BP86-D3BJ/def2-TZVPP level for standard conditions with the FREEH module. IR and Raman intensities were calculated with the Gaussian[32] software at BP86/def2-TZVP.

Symmetry restricted structure optimizations of the $[Cr(CO)_6]^+$ cation were performed with the TURBOMOLE 7.1 software package on the TPSSh[33,34]/def2-TZVPP level of theory with RI approximation and D3-BJ dispersion correction (integration grid m5, SCF convergence 10^{-9} a.u., density convergence $\leq 10^{-7}$, cartesian gradient $\leq 10^{-4}$). DLPNO-CCSD(T)[35,36]/def2-TZVPP

single point energy calculations were performed with the ORCA 4.0.1[37] program code without symmetry restriction. We have chosen tight thresholds for the domain-based local pair natural orbital method (DLPNO), i.e. pair correlation energy threshold $T_{CutPairs}=10^{-5}$ E_h, PNO occupation threshold $T_{CutPNO}=10^{-7}$ and domain size for PNO expansion $T_{CutMKN}=10^{-4}$. We made use of the RIJCOSX procedure[38–40].

State Averaged (SA) Complete Active Shell Self Consistent Field (CAS-SCF)[41] calculations were done with ORCA 4.0.1 using Dunning's correlation consistent cc-pVTZ basis set[42,43]. Correlation corrections were taken into account by Strongly-Contracted N-Electron Valence State Perturbation Theory (SC-NEVPT2)[44,45]. The CAS-SCF wave function served to calculate g-tensors for minimum structures of the $[Cr(CO)_6]^{\bullet+}$ cation by the Effective Hamiltonian formalism[46]. The SA-CAS-SCF calculations were carried out in the basis of 13 doublet, 17 quartet and 1 sextet roots arising from the ^6S, ^4G, ^4P, ^4D and ^2I terms of the free Cr^+ cation. The active space consists of 9 electrons in 10 orbitals of the first and second chromium *d*-shell and two additional chromium-ligand binding orbitals, i.e. CAS(9,12).

2. Experimental glassware and pictures of complexes 1 and 2

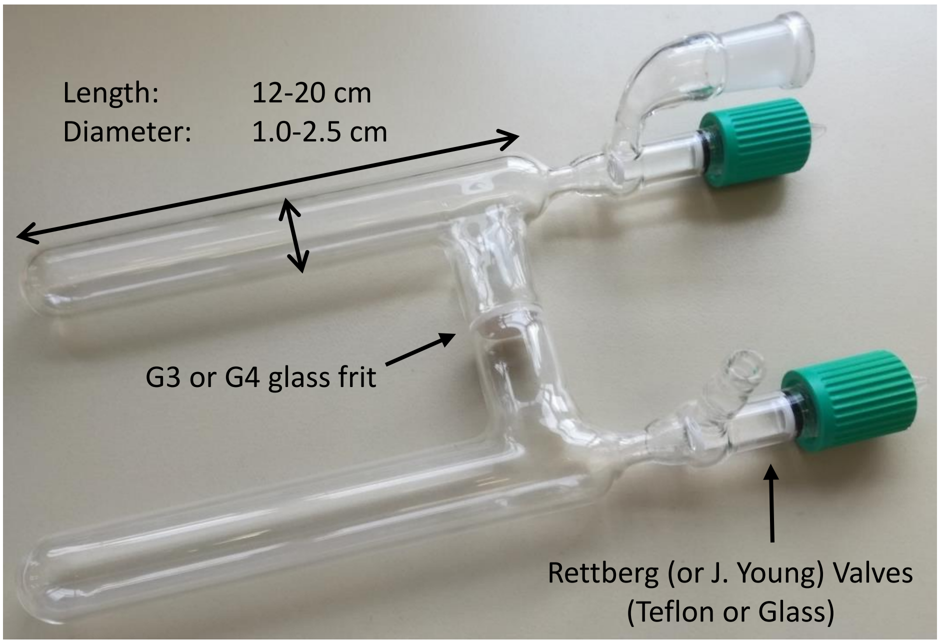

Supplementary Figure 1. Double-Schlenk tube that was typically used for most reactions and crystallizations. Note that different varieties (sizes, Rettberg or J. Young valves) were used.

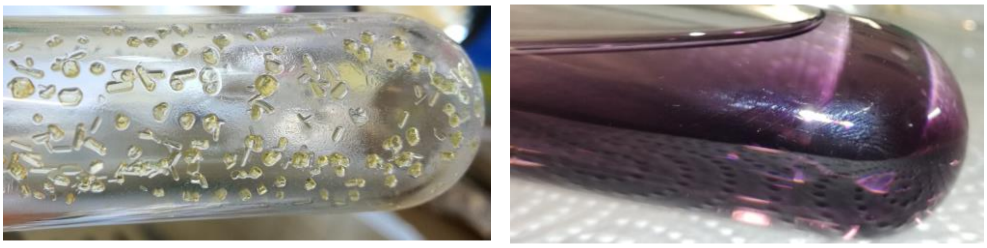

Supplementary Figure 2. Yellow crystals and purple *o*DFB solution of **1**.

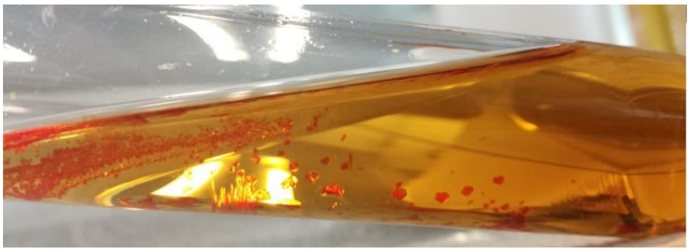

Supplementary Figure 3. Orange crystals and orange *o*DFB solution of **3**

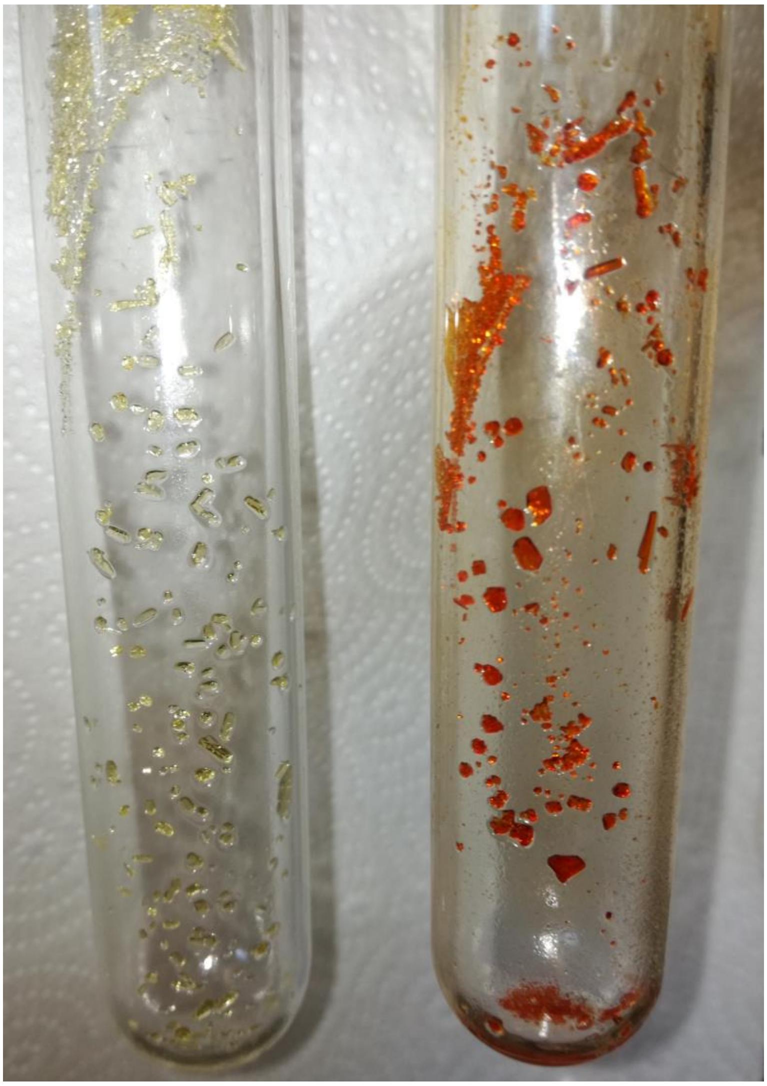

Supplementary Figure 4. Yellow (left, **1**) and orange (right, **2**) block-shaped crystals.

3. Synthesis of $NO[F\text{-}\{Al(OR^F)_3\}_2]$

A double-Schlenk flask was equipped inside the glove box with $Me_3Si\text{-}F\text{-}Al(OR^F)_3$ (1970 mg, 2.39 mmol, 2 eq.) and $NO[PF_6]$ (207 mg, 1.18 mmol, 1 eq.). The flask was equipped with a bubbler and SO_2 (ca. 5 mL) was condensed onto the reaction mixture at –196 °C. The vessel was then carefully vented with Ar and the bubbler was opened towards the fume hood while the reaction mixture was stirred at –35 °C for 45 min (evolution of PF_5!). Then, the volatiles were removed at 0 °C and a white solid was obtained, which was further dried *in vacuo* (Yield: 1700 mg, 1.12 mmol, 95%).

FT Raman (1000 scans, 100 mW, 4 cm^{-1}): $\tilde{\nu}/cm^{-1}$ (intensity) = 2339 (vw, $\nu(NO^+)$), 1357 (vvw), 1319 (vw), 1280 (vw), 1255 (vw), 977 (vvw), 815 (mw), 753 (vvs), 571 (w), 539 (mw), 365 (w), 326 (ms), 292 (w), 235 (vw), 119 (vvw), 94 (vvw).

^{1}H NMR (400.17 MHz, 1,2,3,4-$F_4C_6H_2$, 298 K): *minor impurities at ~8.7, ~4.0 (solvent) and ~1.0 ppm*; **^{14}N NMR** (28.92 MHz, 1,2,3,4-$F_4C_6H_2$, 298 K): δ /ppm = 31 (br. s., 1N, [**N**O×(1,2,3,4-$F_4C_6H_2)_x]^+$); **^{19}F NMR** (376.54 MHz, 1,2,3,4-$F_4C_6H_2$, 298 K): δ/ppm = –76.1 (s, 54F, F-$\{Al(OC(C\mathbf{F}_3)_3)_3\}_2$, –184.7 (br. s, 1F, **F**-$\{Al(OR^F)_3\}_2$), *minor impurities at –76 and –76.5 ppm, minor impurities (coming from the solvent) at –116, –134 and –144 ppm*; **^{27}Al NMR** (104.27 MHz, 1,2,3,4-$F_4C_6H_2$, 298 K): δ /ppm = ~32 (v.br. s, 2Al, F-$\{\mathbf{Al}(OR^F)_3\}_2$). (See Supplementary Figure 5 to Supplementary Figure 8).

Note: $NO[PF_6]$ was dried in high vacuum (10^{-5} mbar) over the period of several days prior to use; upon dissolution in 1,2,3,4-$F_4C_6H_2$, an intensely coloured $[Arene\text{-}NO]^+$ charge-transfer complex is formed.

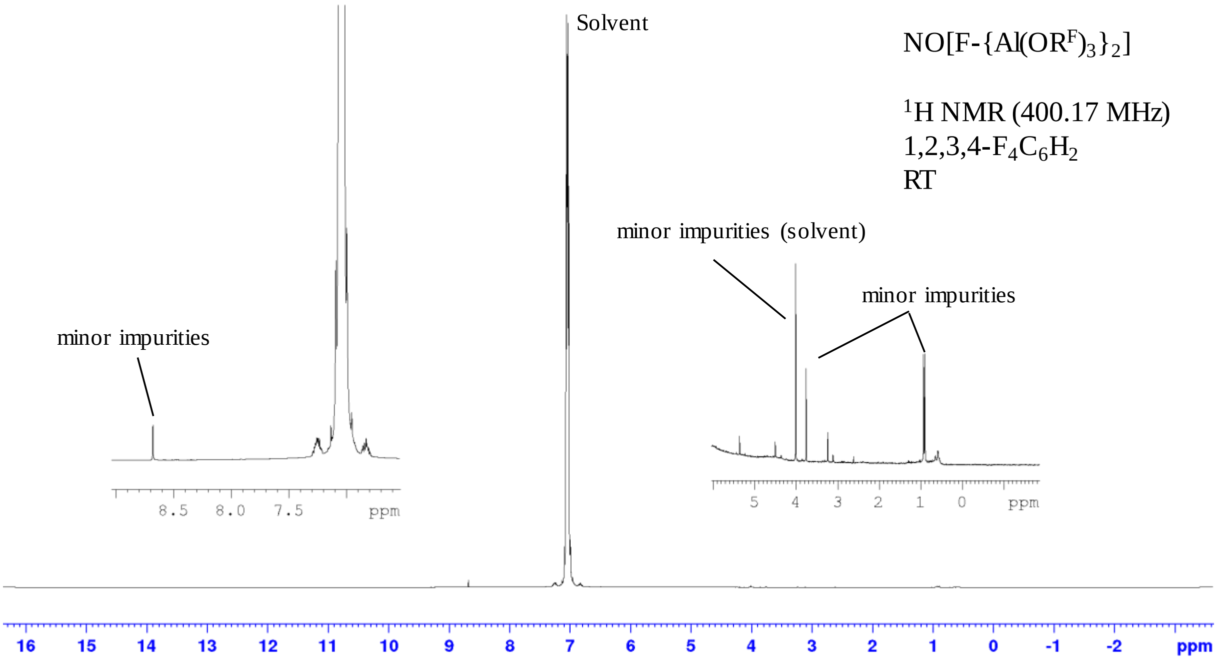

Supplementary Figure 5. ^{1}H NMR spectrum (400.17 MHz, 1,2,3,4-$F_4C_6H_2$, RT) of NO[F-{Al(OR^F)$_3$}$_2$].

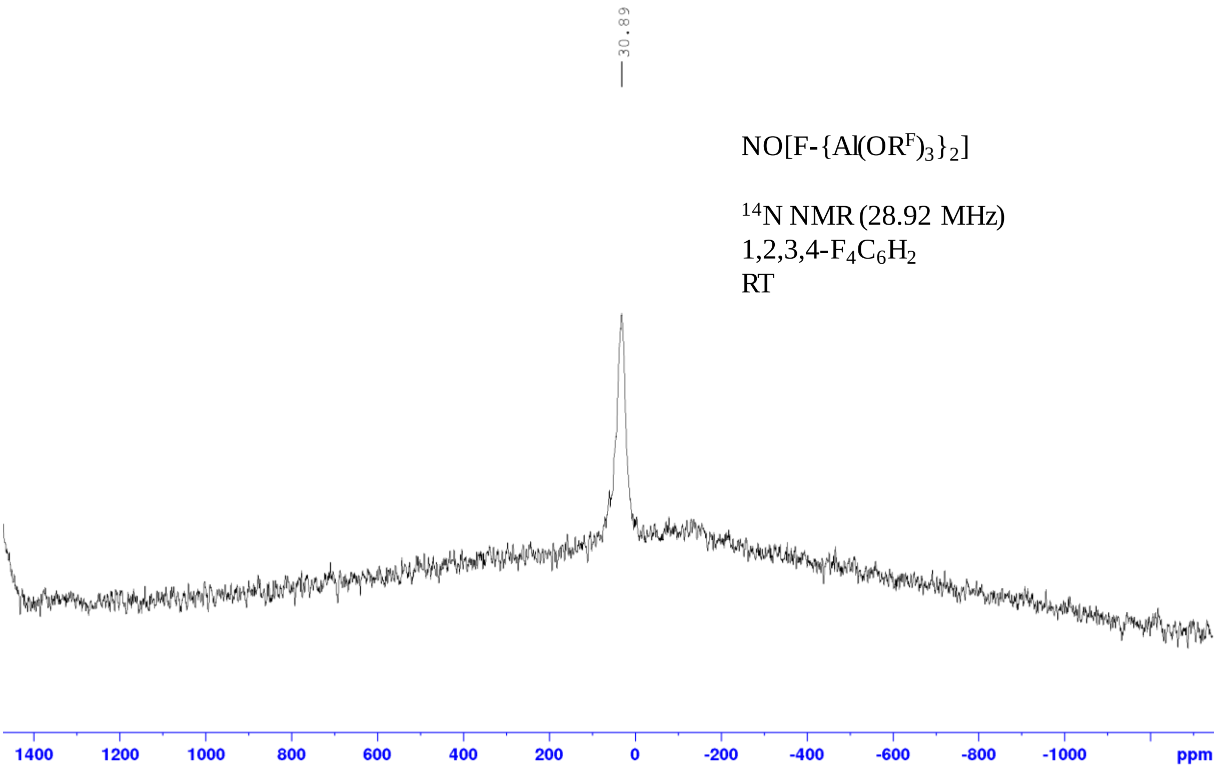

Supplementary Figure 6. ^{14}N NMR spectrum (28.92 MHz, 1,2,3,4-$F_4C_6H_2$, RT) of NO[F-{Al(OR^F)$_3$}$_2$].

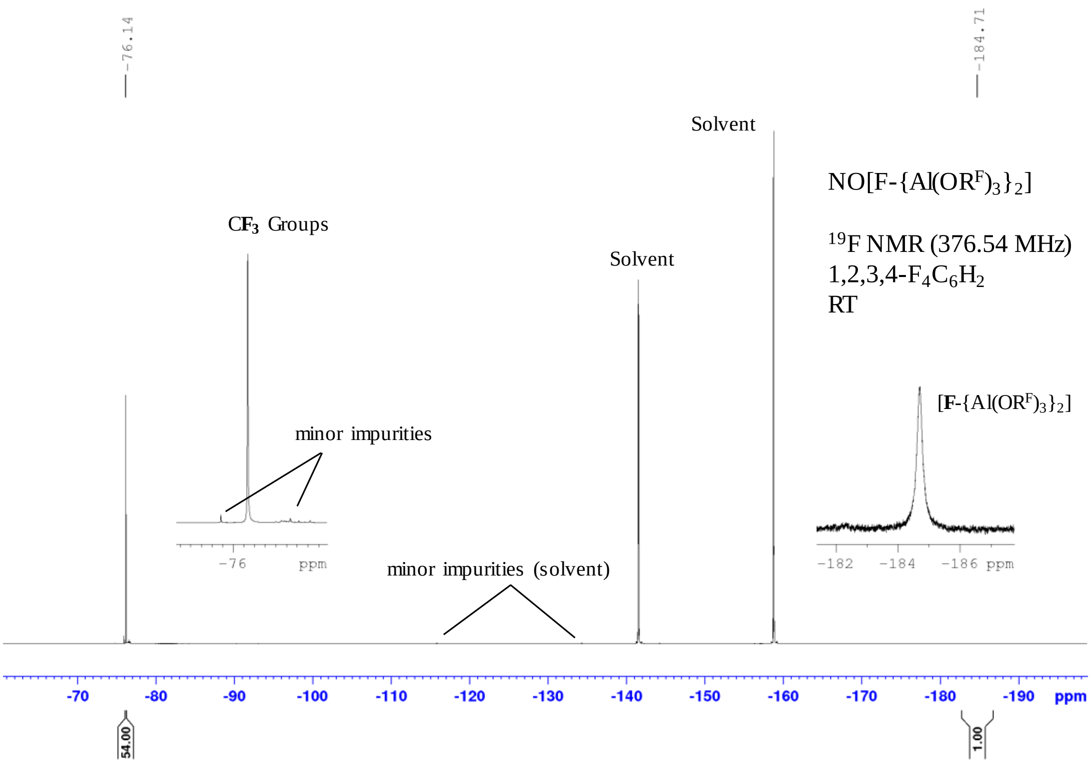

Supplementary Figure 7. ^{19}F NMR spectrum (376.54 MHz, 1,2,3,4-$F_4C_6H_2$, RT) of NO[F-{Al(OR^F)$_3$}$_2$].

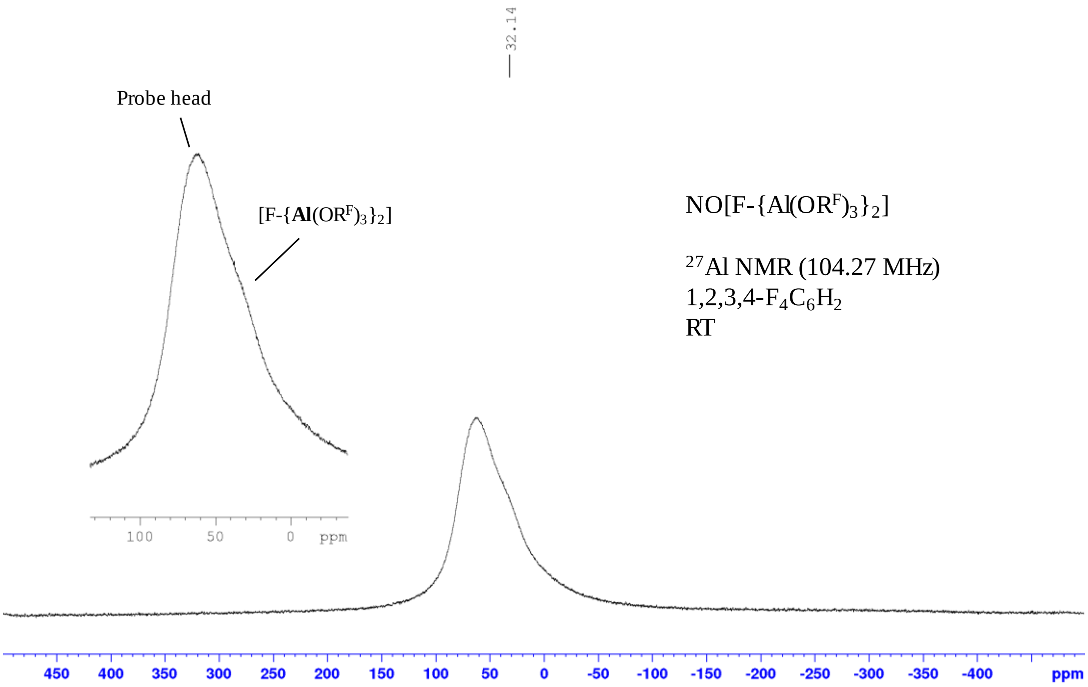

Supplementary Figure 8. ^{27}Al NMR spectrum (104.27 MHz, 1,2,3,4-$F_4C_6H_2$, RT) of NO[F-{Al(OR^F)$_3$}$_2$].

4. Detailed Synthesis and Characterization of compounds 1–4

[$Cr(CO)_6$][$Al(OR^F)_4$] (1)

A double-Schlenk flask was equipped inside the glove box with $Cr(CO)_6$ (51 mg, 0.232 mmol, 1 eq.) and NO[$Al(OR^F)_4$] (230 mg, 0.231 mmol, 1 eq.). Then, CH_2Cl_2 (~4 mL) was condensed at –196 °C onto the reaction mixture and it was allowed to thaw to –78 °C under dynamic vacuum. Upon dissolution, the colour turned initially blue and a gas evolution was visible. The mixture was stirred under dynamic vacuum (so that the solvent was very mildly boiling) until it reached r.t. over the period of an hour. The CH_2Cl_2 (~2 mL) was then filtered off and the remaining solid was dried *in vacuo* in order to yield **1** as an off-white to beige solid (260 mg, 0.219 mmol, 94%). Single crystals were obtained by slow vapour diffusion of *n*-pentane in a solution of **1** in 1,2-$F_2C_6H_4$ (*o*DFB) at r.t.

FTIR (ZnSe, ATR): $\tilde{\nu}$/cm^{-1} (intensity) = 2094 (s), 1843 (vvw, *trace of [$Cr(CO)_5(NO)$]$^+$*), 1508 (vvw, *trace of residual oDFB*), 1352 (vw), 1298 (mw), 1272 (ms), 1239 (s), 1208 (vvs), 1161 (ms), 969 (vvs), 831 (vw), 756 (vvw), 726 (vvs), 571 (vvw), 560 (vw). (See Supplementary Figure 42)

FT Raman (1000 scans, 100 mW, 4 cm^{-1}): $\tilde{\nu}$/cm^{-1} (intensity) = 2175 (vvs), 2128 (mw), 2062 (vw), 1304 (vvw), 1272 (vvw), 1235 (vvw), 1163 (vvw), 977 (vvw), 798 (w), 747 (w), 572 (vvw), 563 (vvw), 538 (vw), 368 (vvw), 332 (m), 288 (w), 234 (vvw), 173 (vvw). (See Supplementary Figure 43)

^{1}H NMR (400.17 MHz, *o*DFB, 298 K): *only solvent signals*; **^{13}C{^{1}H} NMR** (100.62 MHz, *o*DFB, 298 K): δ/ppm = 121.7 (q, ^1J(C,F) = 293 Hz, 12C, **C**F_3, *in part overlapped by solvent signals*); **^{19}F NMR** (376.54 MHz, *o*DFB, 298 K): δ /ppm = –75.3 (s, 36F, 4 × C(C**F**$_3$)$_3$); **^{27}Al NMR** (104.27 MHz, *o*DFB, 298 K): δ/ppm = 35.0 (s, 1Al, **Al**(OR^F)$_4$). (See Supplementary Figure 9 to Supplementary Figure 12)

Note: The solution of **1** in *o*DFB is of an intensive purple colour and yields light-yellow crystals (see Supplementary Figure 2 and Supplementary Figure 4 left). The content of orange [$Cr(CO)_5(NO)$][$Al(OR^F)_4$] impurities determine the colour of the product, giving the powder as well as the crystals a yellow to orange touch.

The respective UV/Vis spectra are shown in Supplementary Figure 50.

[Cr(CO)$_6$][F-{Al(OR^F)$_3$}$_2$] (2)

A double-Schlenk flask was equipped inside the glove box with Cr(CO)$_6$ (66 mg, 0.30 mmol, 1.3 eq) and NO[F-{Al(OR^F)$_3$}$_2$] (350 mg, 0.231 mmol, 1 eq.). Then, CH_2Cl_2 (~5 mL) was added at –20 °C to the reaction mixture and it was stirred at –20 °C under dynamic vacuum for 40 min. The reaction mixture was allowed to warm up to r.t. and the crude product was washed with the residual CH_2Cl_2 (three times). After dying *in vacuo,* **2** yielded as an off-white solid (340 mg, 0.20 mmol, 91%). Colourless single crystals were obtained by slow vapour diffusion of *n*-pentane in a solution of **2** in *o*DFB at r.t.

FTIR (ZnSe, ATR): $\tilde{\nu}$/cm^{-1} (intensity) = 2096 (s), 1841 (vvw, *trace of [Cr(CO)$_5$(NO)]$^+$*), 1509 (vvw, *trace of residual oDFB*), 1355 (vw), 1300 (mw), 1247 (vvs), 1216 (vvs), 1184 (s), 972 (vvs), 865 (w), 760 (vvw), 751 (vvw), 726 (vvs), 639 (w), 568 (vw). (See Supplementary Figure 44)

FT Raman (100 scans, 500 mW, 4 cm^{-1}): $\tilde{\nu}$/cm^{-1} (intensity) = 2173 (vvs), 2126 (mw), 2062 (vw), 1304 (vvw), 1281 (vvw), 1244 (vvw), 1180 (vvw), 1131 (vvw), 980 (vvw), 818 (vw), 753 (ms), 571 (vw), 539 (vw), 370 (vw), 328 (ms), 291 (vw), 234 (vvw). (See Supplementary Figure 45)

^{1}H NMR (400.17 MHz, *o*DFB, 298 K): *only solvent signals*; **^{13}C{^{1}H} NMR** (50.32 MHz, *o*DFB, 298 K): *only solvent signals*; **^{19}F NMR** (376.54 MHz, *o*DFB, 298 K): δ/ppm = –75.5 (s, 54F, F-{Al(OC(C**F**$_3$)$_3$)$_3$}$_2$, –184.5 (br. s, 1F, **F**-{Al(OR^F)$_3$}$_2$); **^{27}Al NMR** (104.27 MHz, *o*DFB, 298 K): δ/ppm = ~35 (v.br. s, 2Al, F-{**Al**(OR^F)$_3$}$_2$). (See Supplementary Figure 13 to Supplementary Figure 16)

The respective UV/Vis spectra are shown in Supplementary Figure 50.

[Cr(CO)$_5$(NO)][Al(OR^F)$_4$] (3)

A double-Schlenk flask was equipped inside the glove box with Cr(CO)$_6$ (51 mg, 0.232 mmol, 1 eq) and NO[Al(OR^F)$_4$] (228 mg, 0.229 mmol, 1 eq.). Then, CH_2Cl_2 (~3 mL) was added at r.t. to the reaction mixture and the closed vessel was stirred for 14 d at r.t. After drying *in vacuo*, **3** yielded as an orange solid (250 mg, 0.210 mmol, 92%). Orange single crystals were obtained by slow vapour diffusion of *n*-pentane in a solution of **3** in *o*DFB at r.t.

FTIR (ZnSe, ATR): $\tilde{\nu}$/cm^{-1} (intensity) = 2184 (vw), 2164 (vvw), 2127 (vvw), 2108 (ms), 2074 (vvw), 1841 (mw), 1821 (vvw, *trace of unknown impurity*), 1353 (vw),1299 (mw), 1273 (ms), 1251 (ms),1239 (ms), 1210 (vvs), 1162 (mw), 1140 (vw), 971 (vvs), 866 (vvw), 832 (vw), 756 (vvw), 727 (vs), 657 (w), 640 (vw), 571 (vvw), 561 (vw). (See Supplementary Figure 46)

FT Raman (100 scans, 500 mW, 4 cm^{-1}): $\tilde{\nu}$/cm^{-1} (intensity) = 2185 (m), 2175 (vw), 2164 (m), 2127 (vvs), 2112 (vw), 2076 (vvw), 1843 (vvw), 1304 (vvw), 1275 (vvw), 977 (vvw), 797 (vw), 747 (vw), 640 (vw), 562 (vvw), 539 (vvw), 487 (vvw), 369 (vvw), 332 (vw), 290 (vvw), 234 (vvw). (See Supplementary Figure 47)

^{1}H NMR (300.18 MHz, *o*DFB, 298 K): *only solvent signals*; **^{13}C{^{1}H} NMR** (100.62 MHz, *o*DFB, 298 K): δ/ppm = 201.9 (s, 4C$_{equatorial}$, Cr(**C**O)$_5$(NO)), 187.3 (s, 1C$_{axial}$, Cr(**C**O)$_5$(NO)) , 121.7 (q, ^1J(C,F) = 293 Hz, 12C, **C**F$_3$, *in part overlapped by solvent signals*), 79.5 (m, 4C, 4 × **C**(CF$_3$)$_3$); **^{14}N NMR** (21.69 MHz, *o*DFB, 298 K): δ /ppm = 17 (br. s, 1N, Cr(CO)$_5$(**N**O)); **^{19}F NMR** (376.54 MHz, *o*DFB, 298 K): δ/ppm = –75.3 (s, 36F, 4 × C(C**F**$_3$)$_3$), *minor unknown impurity at –75.1 ppm*; **^{27}Al NMR** (78.22 MHz, *o*DFB, 298 K): δ /ppm = 35.0 (s, 1Al, **Al**(OR^F)$_4$). (See Supplementary Figure 17 to Supplementary Figure 21)

Note: The solution of **3** in *o*DFB is of an orange colour and yields orange crystals (see Supplementary Figure 3 and Supplementary Figure 4).

[Cr(CO)$_5$(NO)][F-{Al(OR^F)$_3$}$_2$] (4)

A double-Schlenk flask was equipped inside the glove box with Cr(CO)$_6$ (50 mg, 0.227 mmol, 1 eq) and NO[F-{Al(OR^F)$_3$}$_2$] (350 mg, 0.231 mmol, 1 eq.). Then, CH_2Cl_2 (~3 mL) was added at r.t. to the reaction mixture and the closed vessel was stirred for 10 d at r.t. After extracting with CH_2Cl_2 (three times) and drying *in vacuo*, **4** yielded as an orange solid (360 mg, 0.211 mmol, 93%). Orange single crystals were obtained by slow vapour diffusion of *n*-pentane in a solution of **4** in *o*DFB at 0 °C.

FTIR (ZnSe, ATR): $\tilde{\nu}$/cm^{-1} (intensity) = 2183 (vw), 2162 (vvw), 2107 (vs), 2099 (m), 2072 (vvw), 1839 (ms), 1809 (vvw *trace of unknown impurity*), 1355 (vw), 1300 (mw), 1278 (m), 1248 (vvs), 1216 (vvs), 1185 (s), 973 (vvs), 865 (w), 760 (vvw), 727 (vvs), 657 (w), 638 (mw), 568 (vw). (See Supplementary Figure 48)

FT Raman (100 scans, 500 mW, 4 cm^{-1}): $\tilde{\nu}$/cm^{-1} (intensity) = 2184 (m), 2173 (vw), 2163 (m), 2125 (vvs), 2110 (vvw),2074 (vvw), 1842 (vvw), 1305 (vvw), 1281 (vvw), 1244 (vvw), 1180 (vvw), 1130 (vvw), 980 (vvw), 818 (vvw), 753 (w), 639 (vw), 572 (vvw), 539 (vvw), 487 (vvw), 370 (vvw), 332 (vw), 293 (vvw), 233 (vvw). (See Supplementary Figure 49)

^{1}H NMR (400.17 MHz, *o*DFB, 298 K): *only solvent signals*; **^{13}C{^{1}H} NMR** (100.62 MHz, *o*DFB, 298 K): δ/ppm = 201.2 (s, 4C$_{equatorial}$, Cr(**C**O)$_5$(NO)), 186.5 (s, 1C$_{axial}$, Cr(**C**O)$_5$(NO)) , 120.3 (q, ^1J(C,F) = 291 Hz, 12C, **C**F$_3$, *in part overlapped by solvent signals*), 78.1 (m, 6C, F-{Al(O**C**(CF$_3$)$_3$)$_3$}$_2$); **^{14}N NMR** (28.92 MHz, *o*DFB, 298 K): δ /ppm = 22 (br. s, 1N, Cr(CO)$_5$(**N**O)); **^{19}F NMR** (376.54 MHz, *o*DFB, 298 K): δ /ppm = –76.3 (s, 54F, F-{Al(OC(C**F**$_3$)$_3$)$_3$}$_2$, –185.2 (br. s, 1F, **F**-{Al(OR^F)$_3$}$_2$); **^{27}Al NMR** (104.27 MHz, *o*DFB, 298 K): δ /ppm = ~34 (v.br. s, 2Al, F-{**Al**(OR^F)$_3$}$_2$). (See Supplementary Figure 22 to Supplementary Figure 26)

5. NMR Spectra

[Cr(CO)$_6$][Al(OR^F)$_4$] (1)

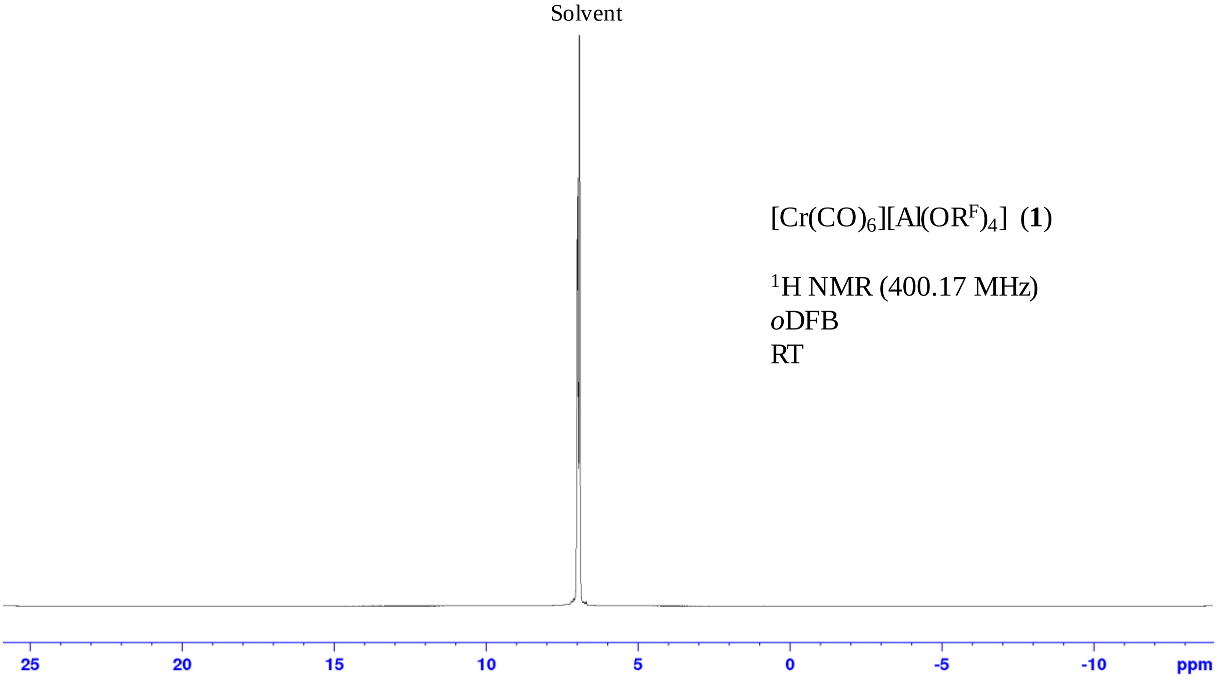

Supplementary Figure 9. ^{1}H NMR spectrum (400.17 MHz, *o*DFB, RT) of **1**

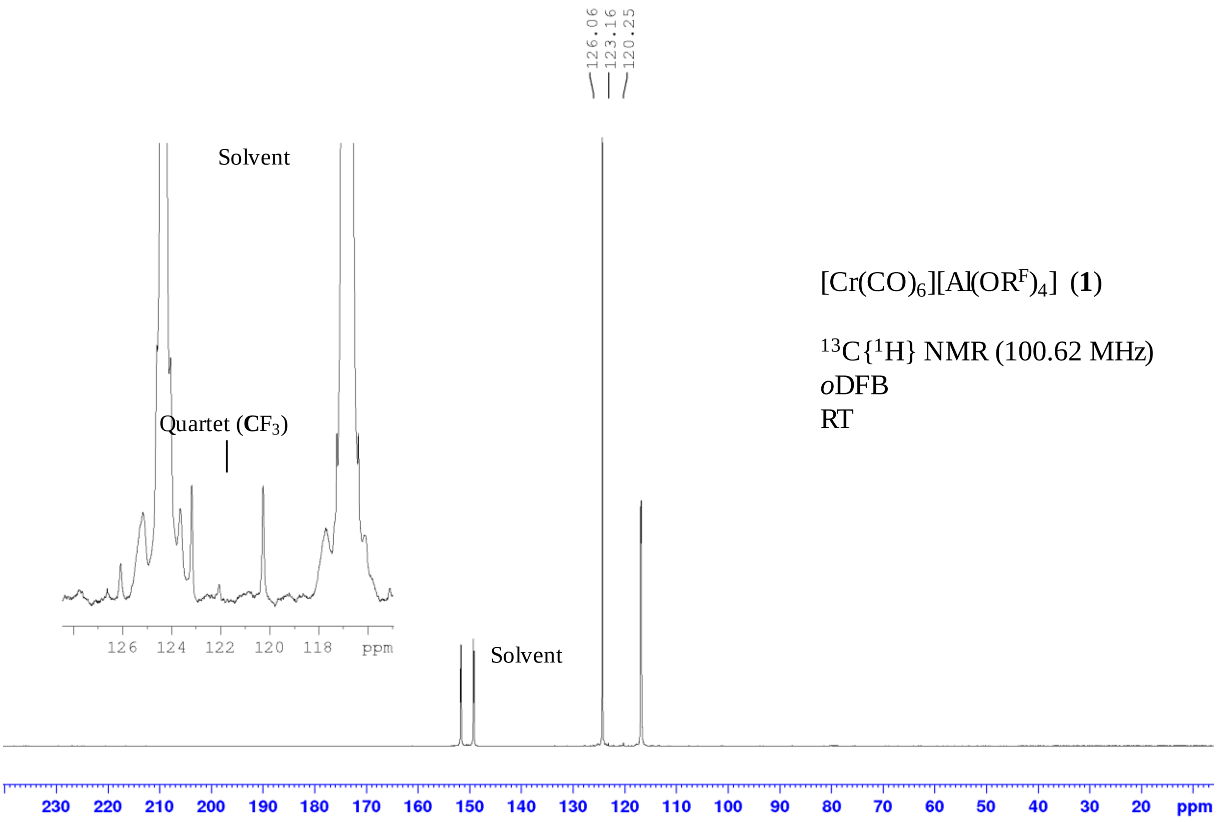

Supplementary Figure 10. ^{13}C{^{1}H} NMR spectrum (100.62 MHz, *o*DFB, RT) of **1**.

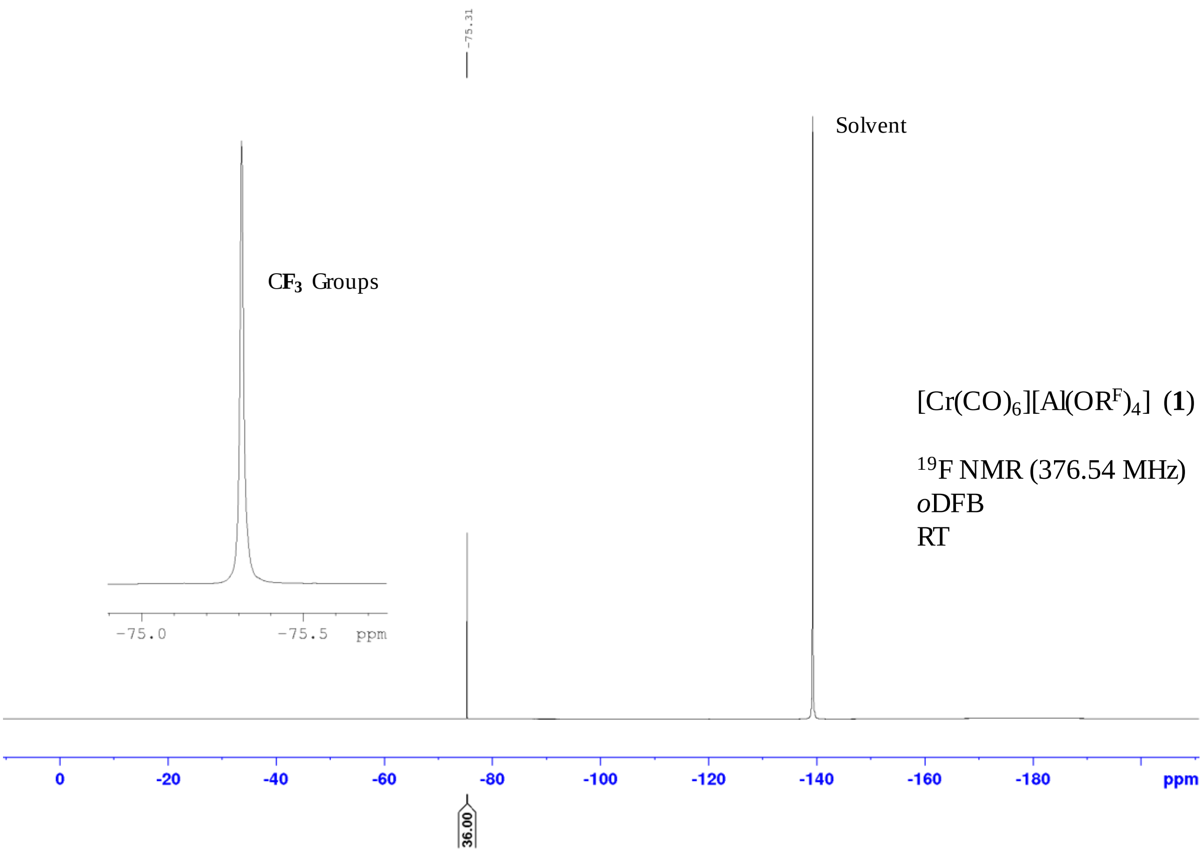

Supplementary Figure 11. ^{19}F NMR spectrum (376.54 MHz, *o*DFB, RT) of **1**.

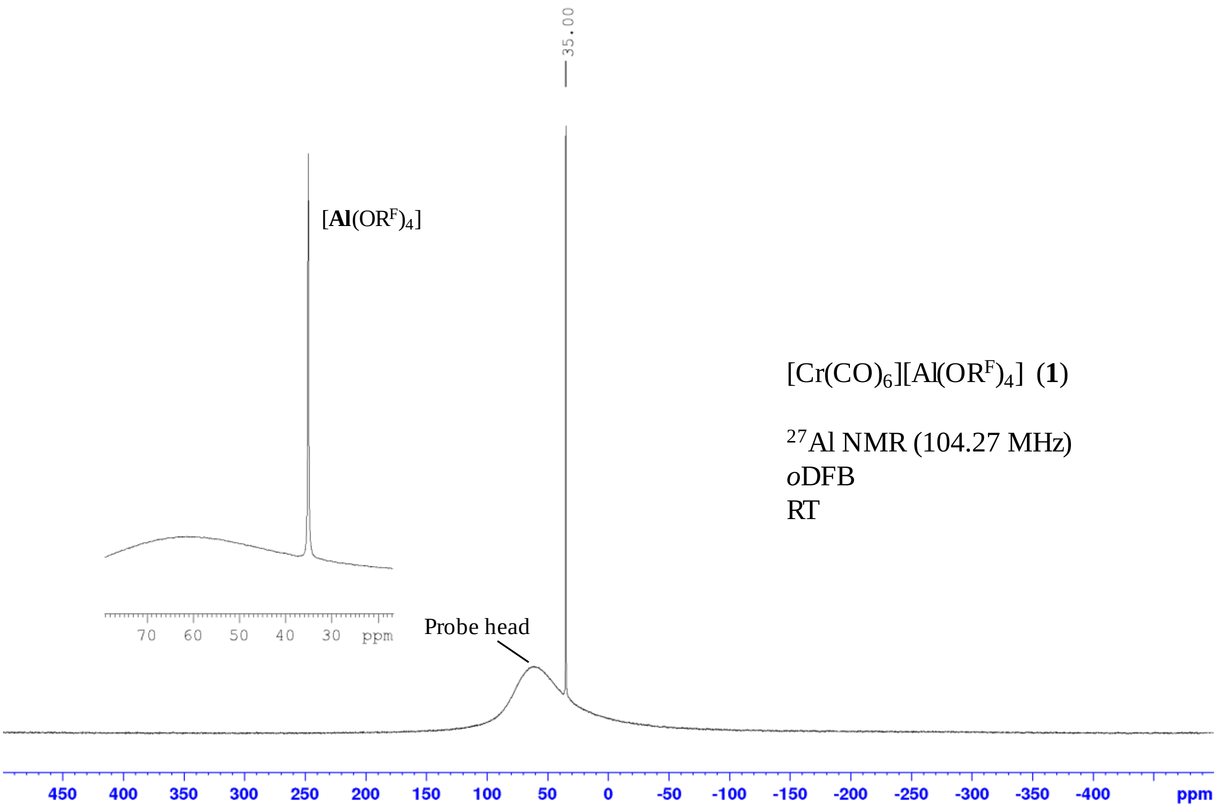

Supplementary Figure 12. ^{27}Al NMR spectrum (104.27 MHz, *o*DFB, RT) of **1**.

$[Cr(CO)_6][F\text{-}\{Al(OR^F)_3\}_2]$ (2)

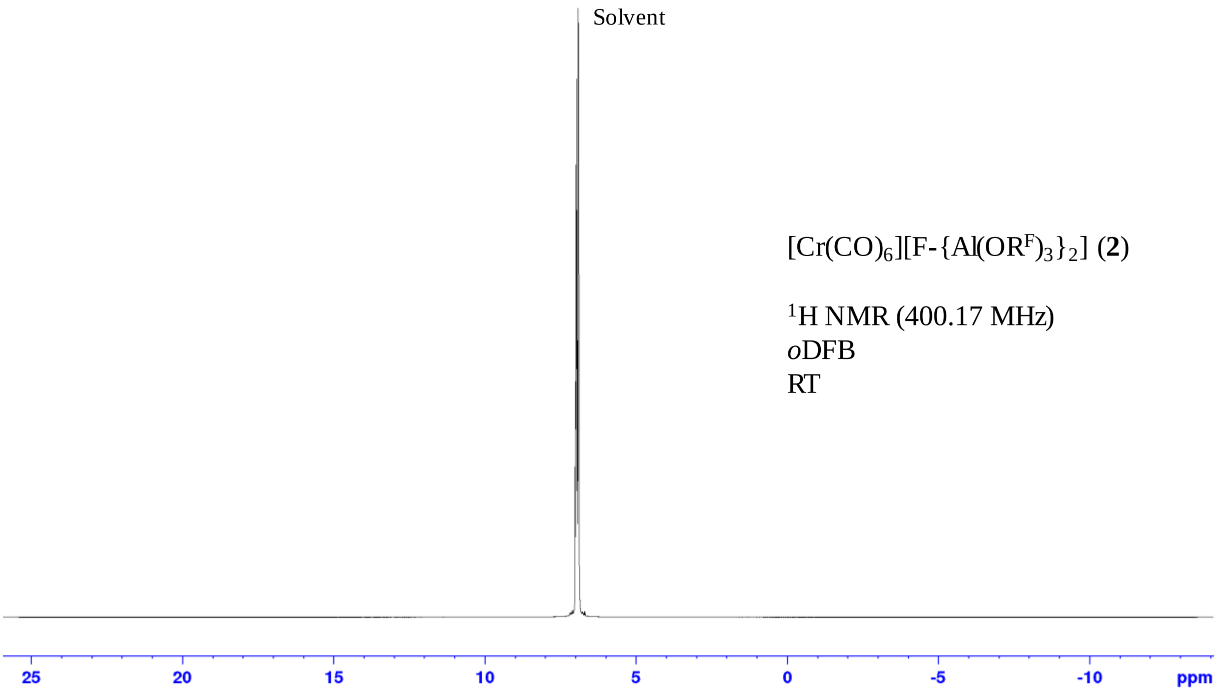

Supplementary Figure 13. ^1H NMR spectrum (400.17 MHz, *o*DFB, RT) of **2**.

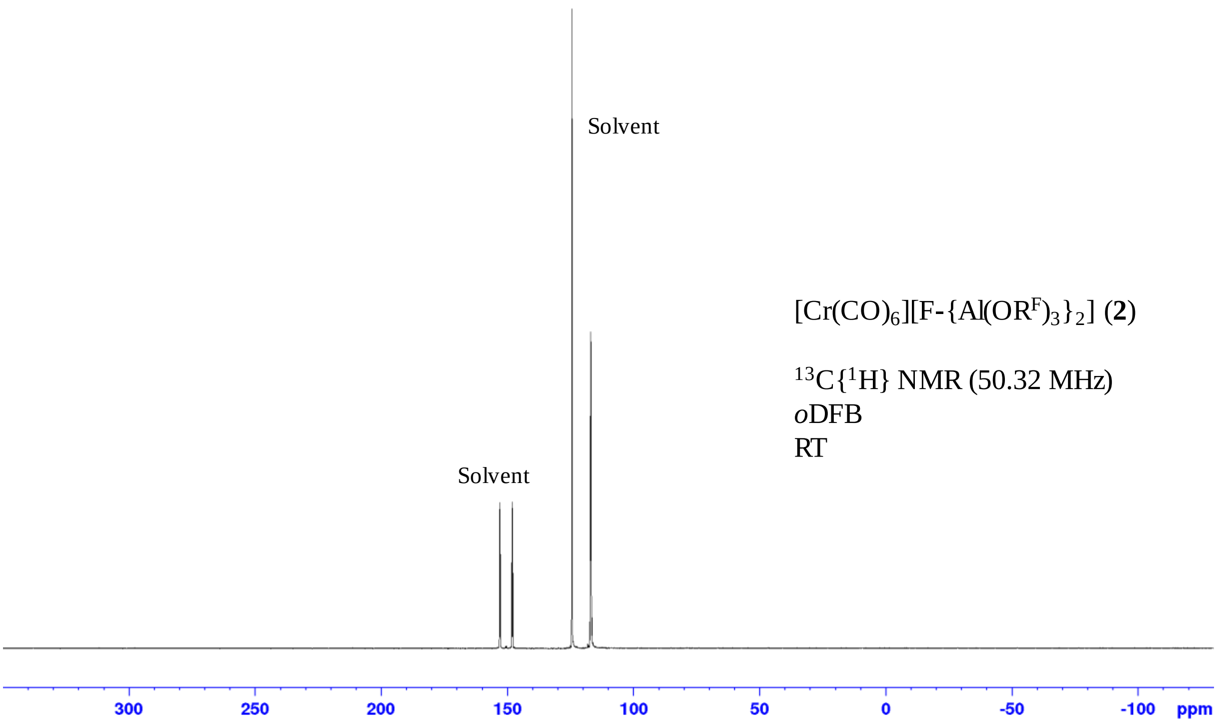

Supplementary Figure 14. $^{13}C\{^1H\}$ NMR spectrum (50.32 MHz, *o*DFB, RT) of **2**.

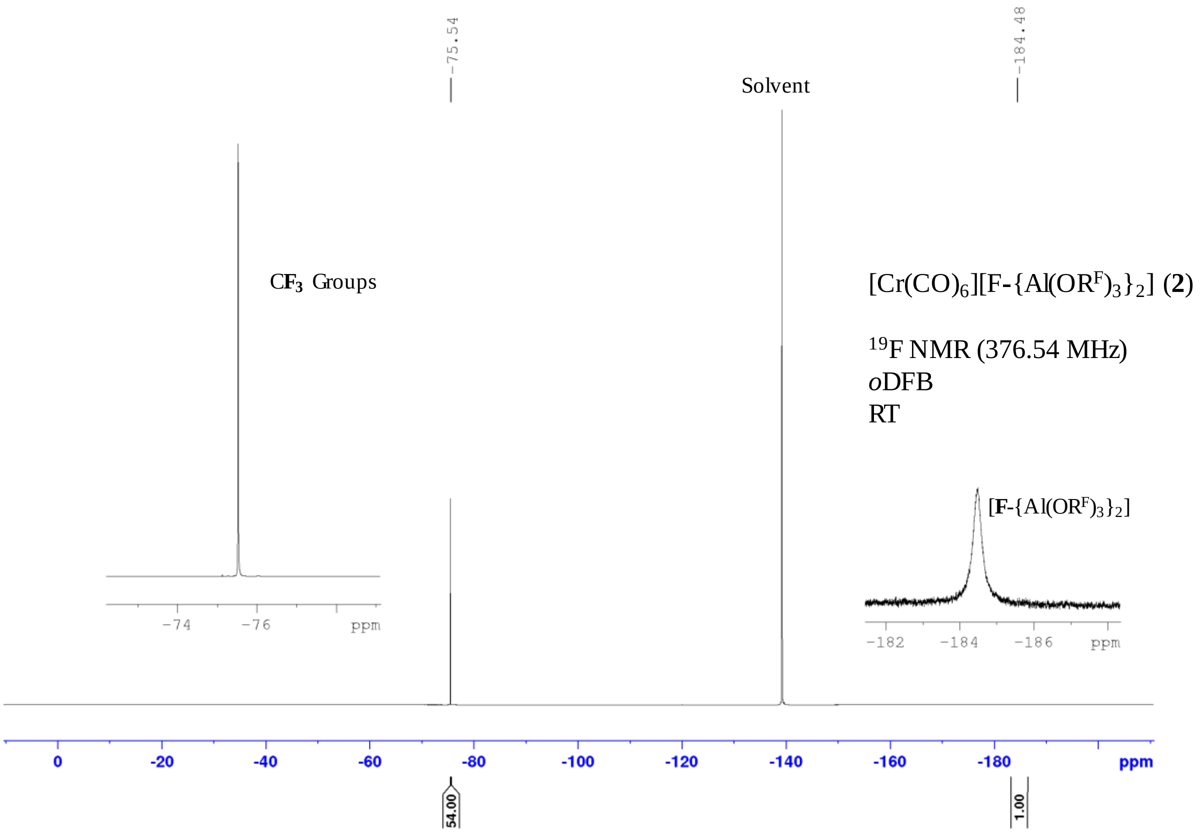

Supplementary Figure 15. ^{19}F NMR spectrum (376.54 MHz, *o*DFB, RT) of **2**.

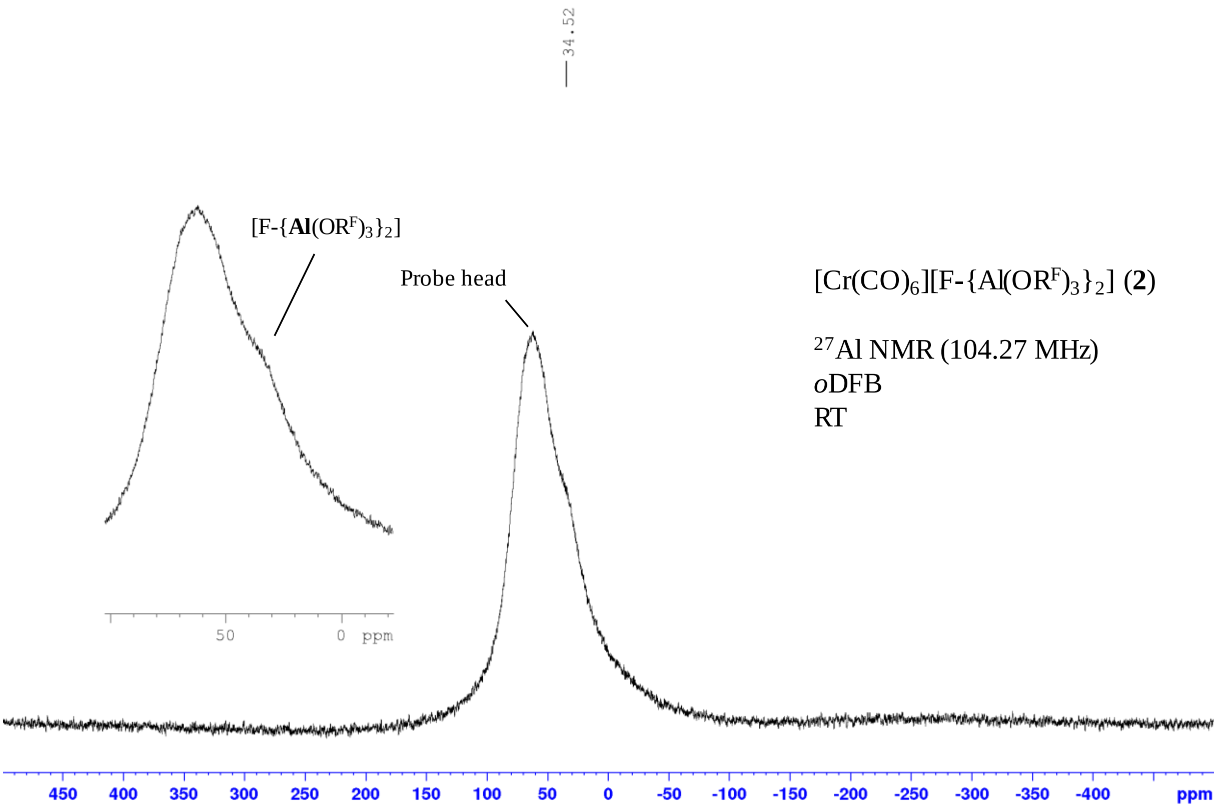

Supplementary Figure 16. ^{27}Al NMR spectrum (104.27 MHz, *o*DFB, RT) of **2**.

$[Cr(CO)_5(NO)][Al(OR^F)_4]$ (3)

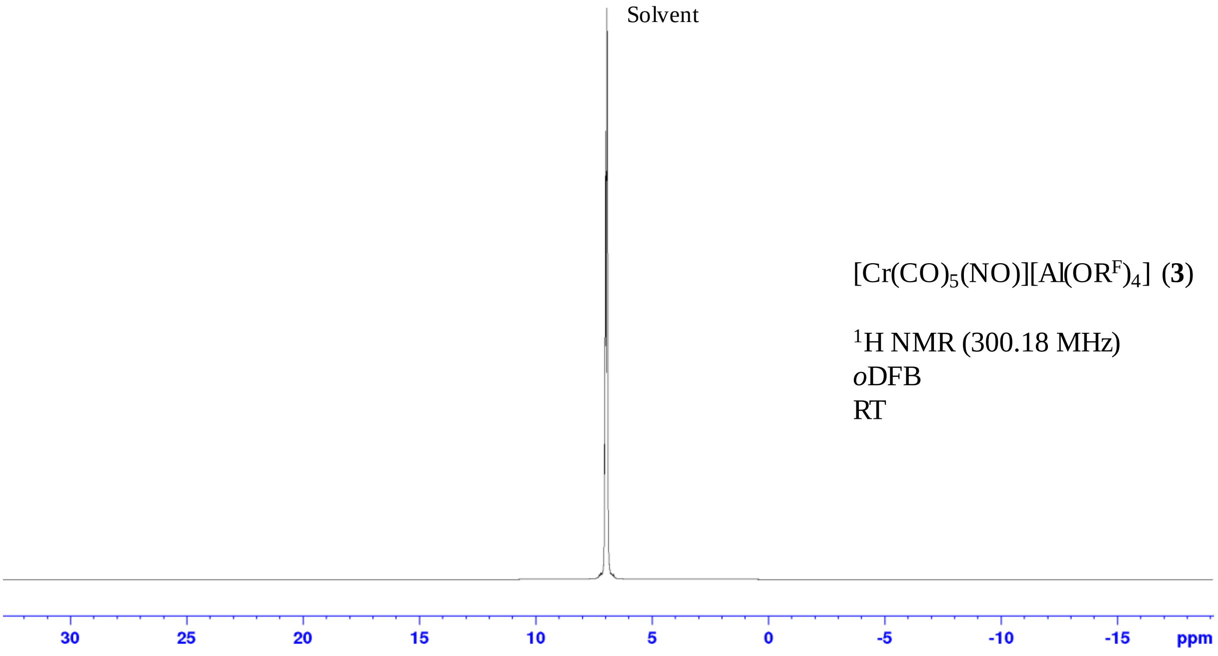

Supplementary Figure 17. ^1H NMR spectrum (400.17 MHz, *o*DFB, RT) of **3**.

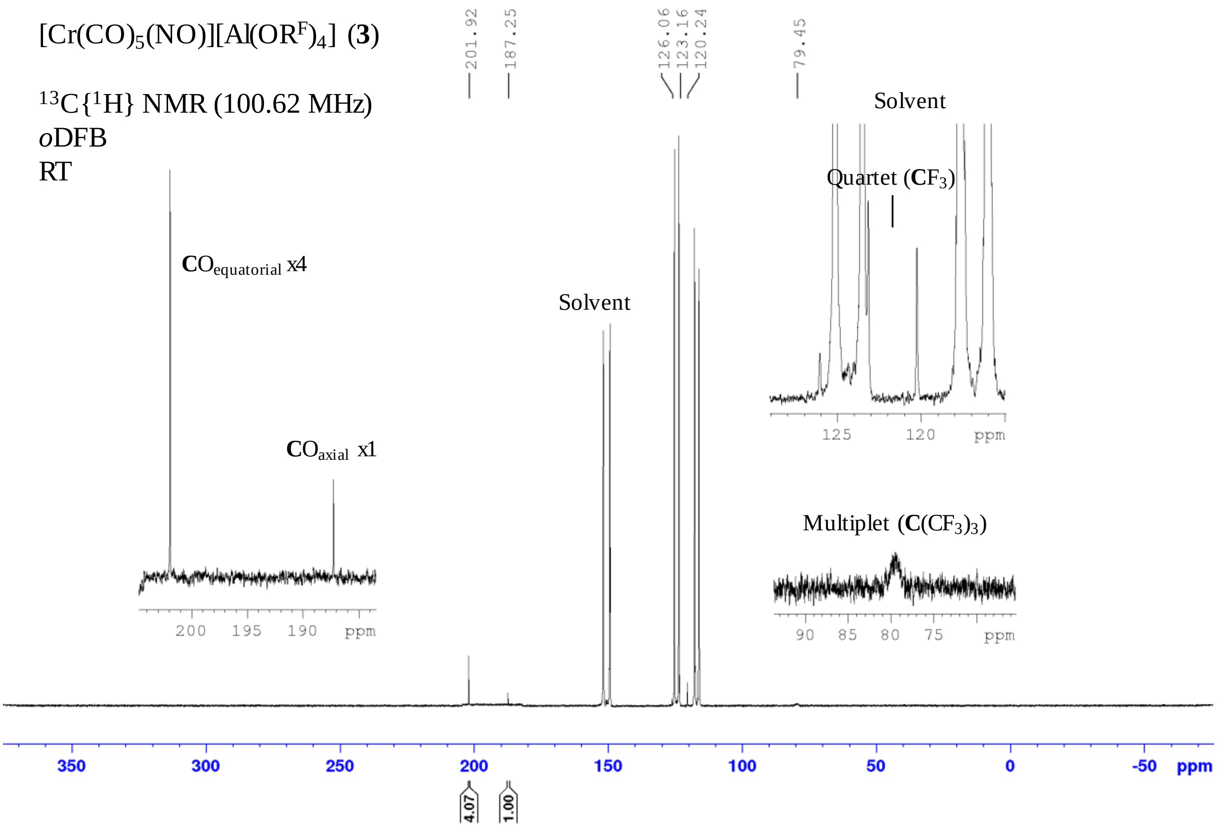

Supplementary Figure 18. $^{13}C\{^1H\}$ NMR spectrum (100.62 MHz, *o*DFB, RT) of **3**.

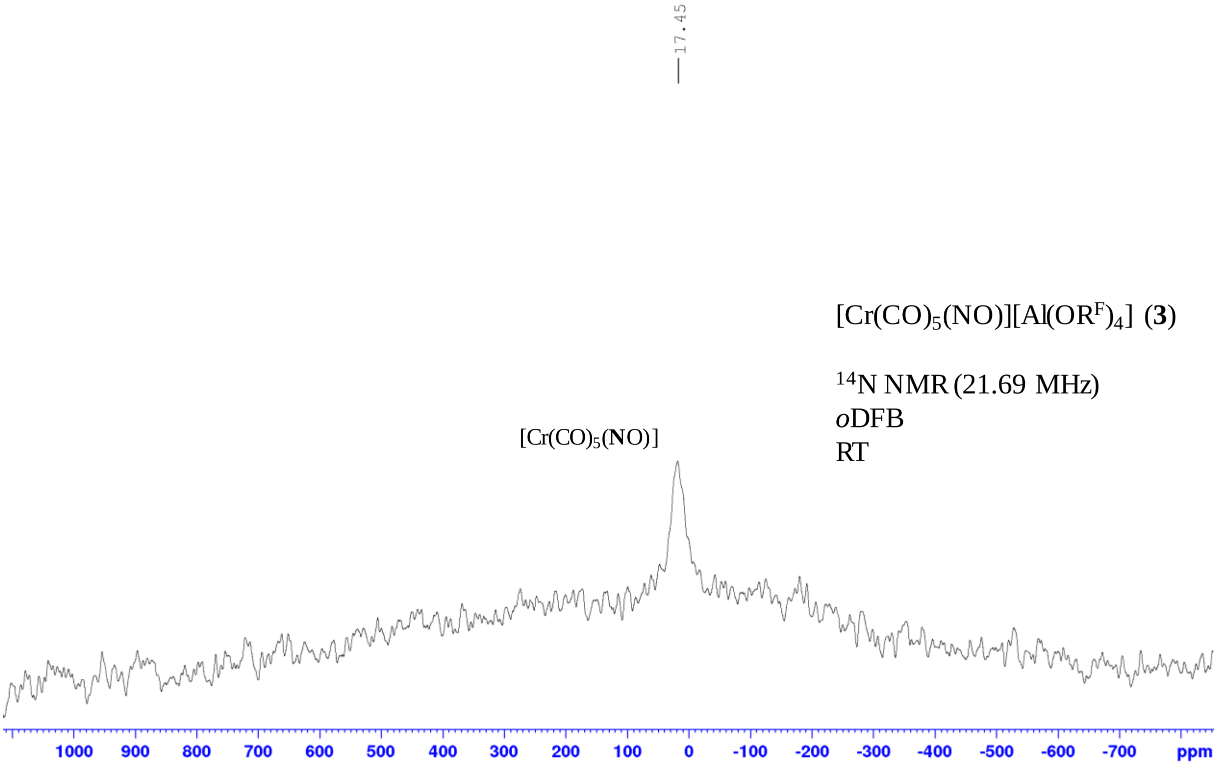

Supplementary Figure 19. ^{14}N NMR spectrum (21.69 MHz, *o*DFB, RT) of **3**.

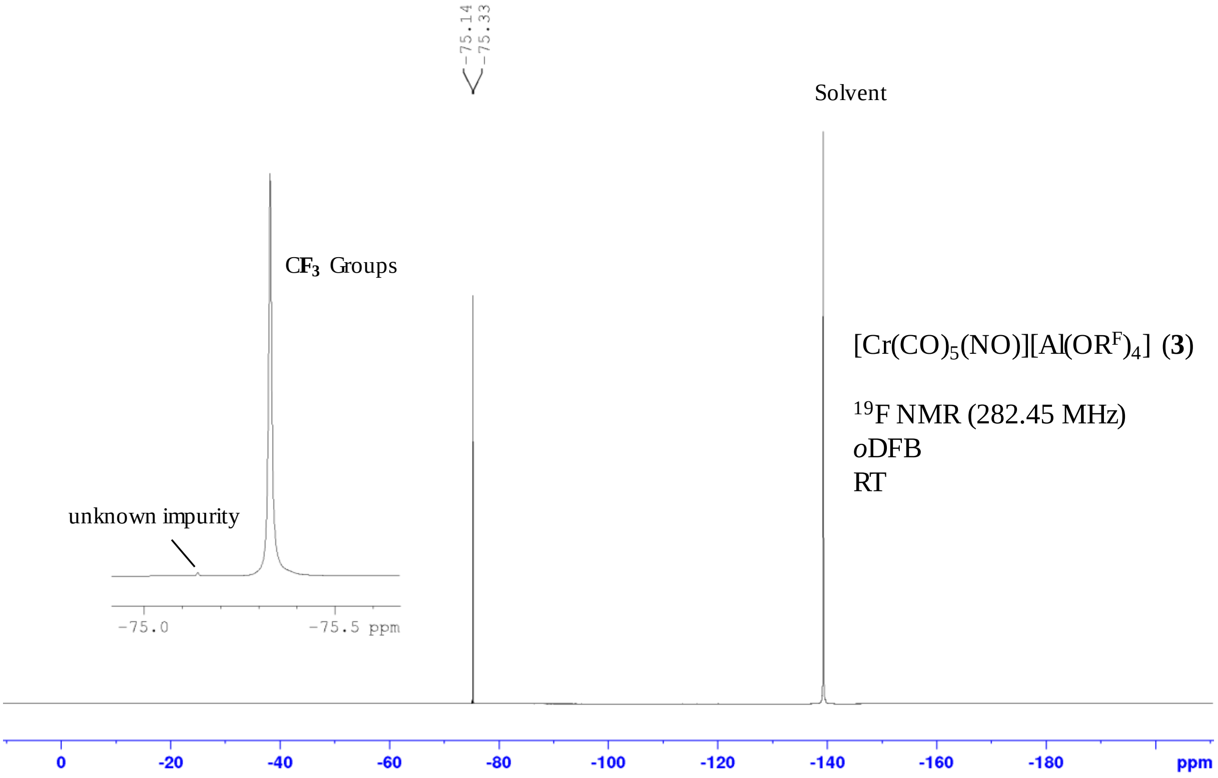

Supplementary Figure 20. ^{19}F NMR spectrum (282.45 MHz, *o*DFB, RT) of **3**.

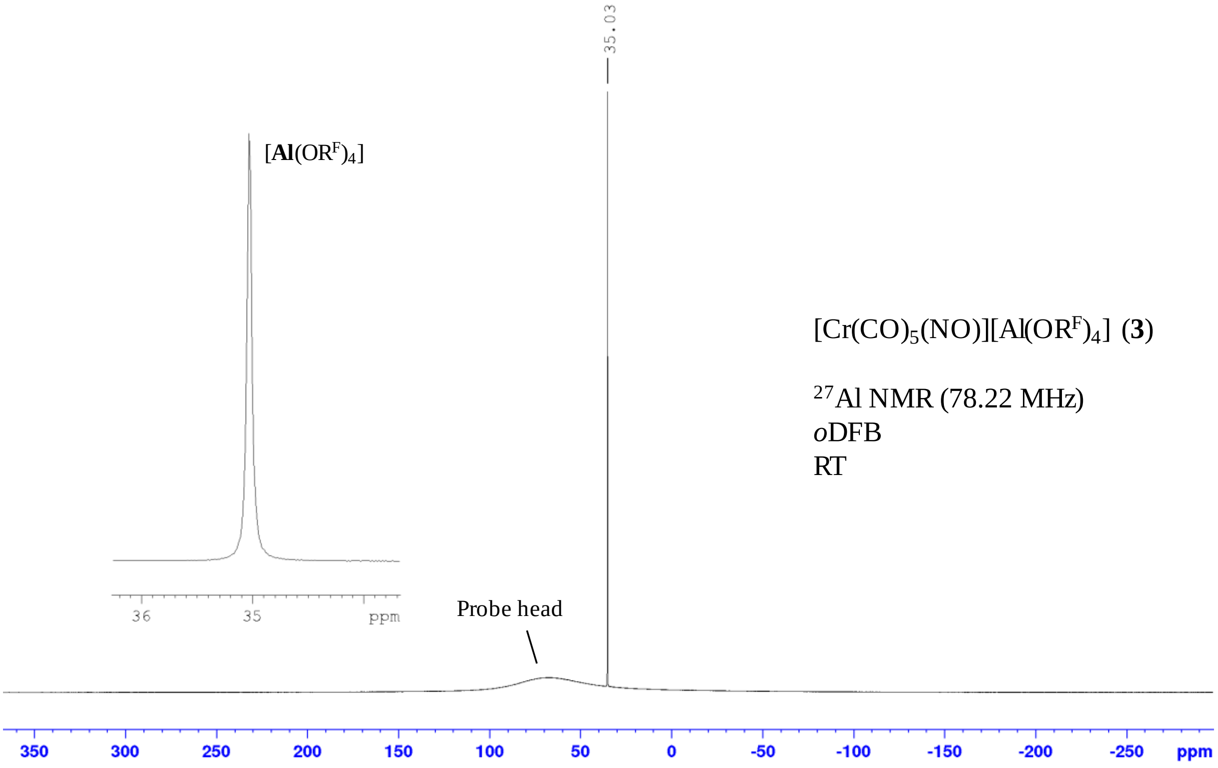

Supplementary Figure 21. ^{27}Al NMR spectrum (78.22 MHz, *o*DFB, RT) of **3**.

$[Cr(CO)_5(NO)][F-\{Al(OR^F)_3\}_2]$ (4)

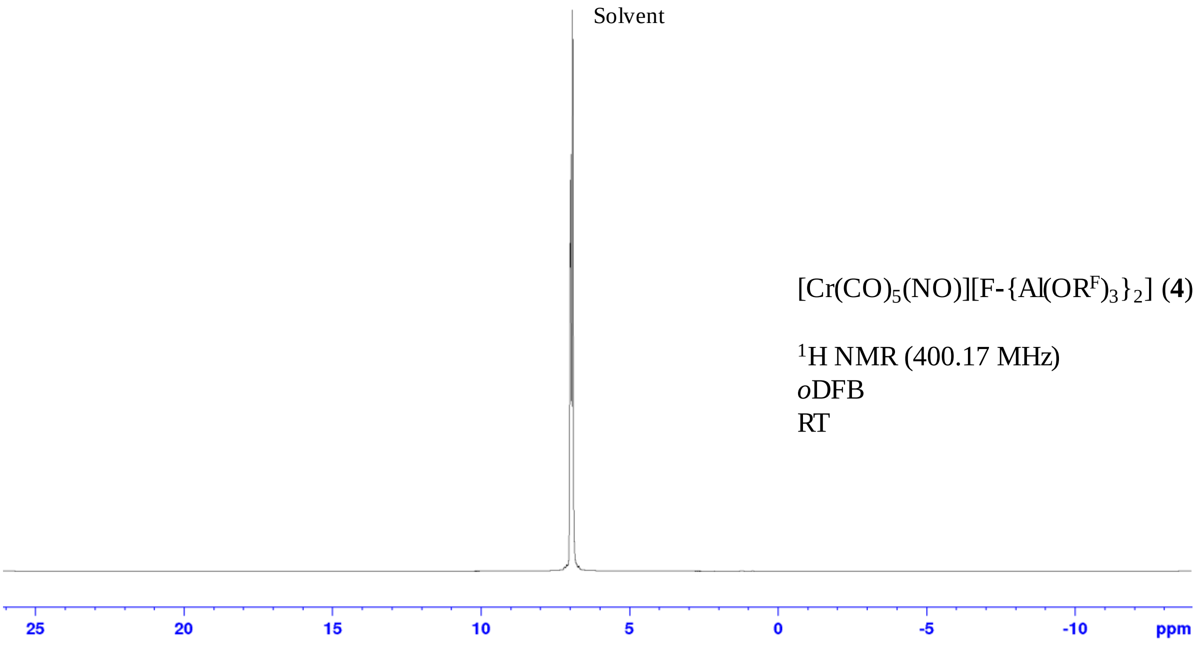

Supplementary Figure 22. ^1H NMR spectrum (400.17 MHz, *o*DFB, RT) of **4**.

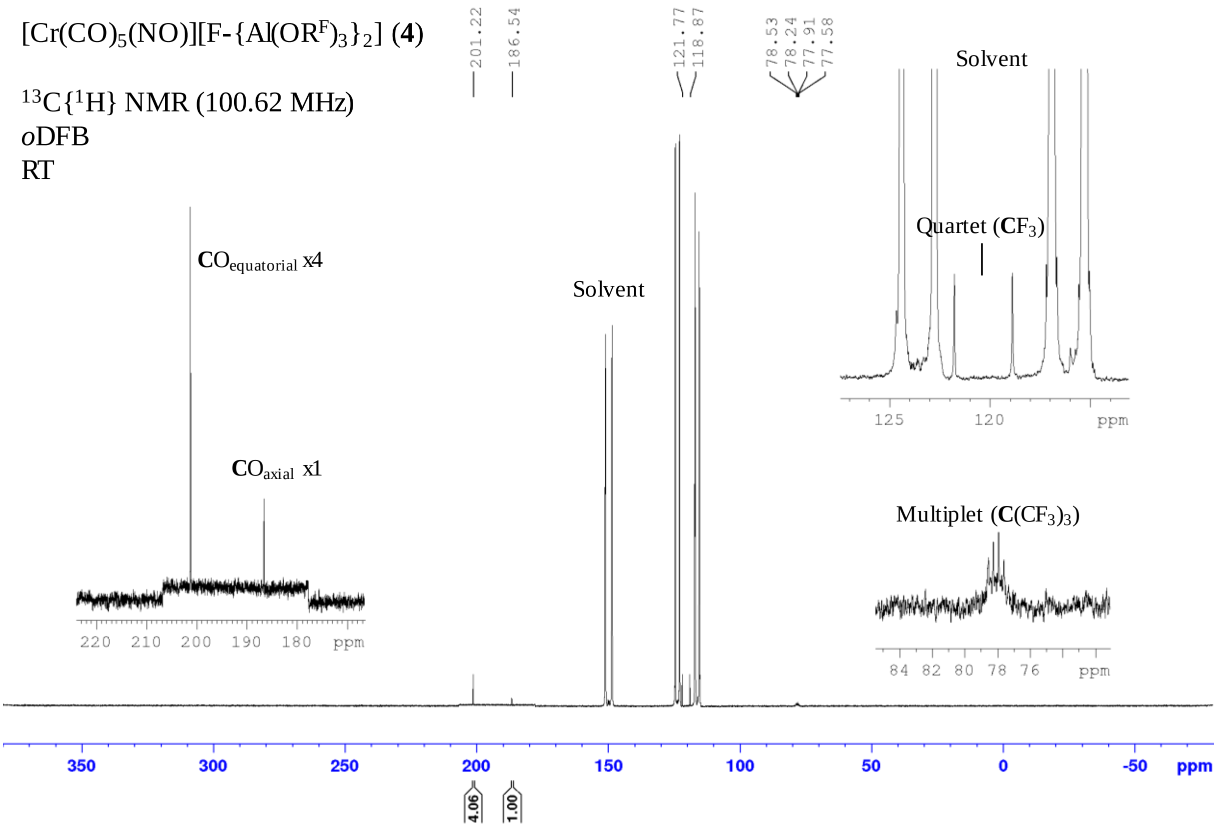

Supplementary Figure 23. $^{13}C\{^1H\}$ NMR spectrum (100.62 MHz, *o*DFB, RT) of **4**.

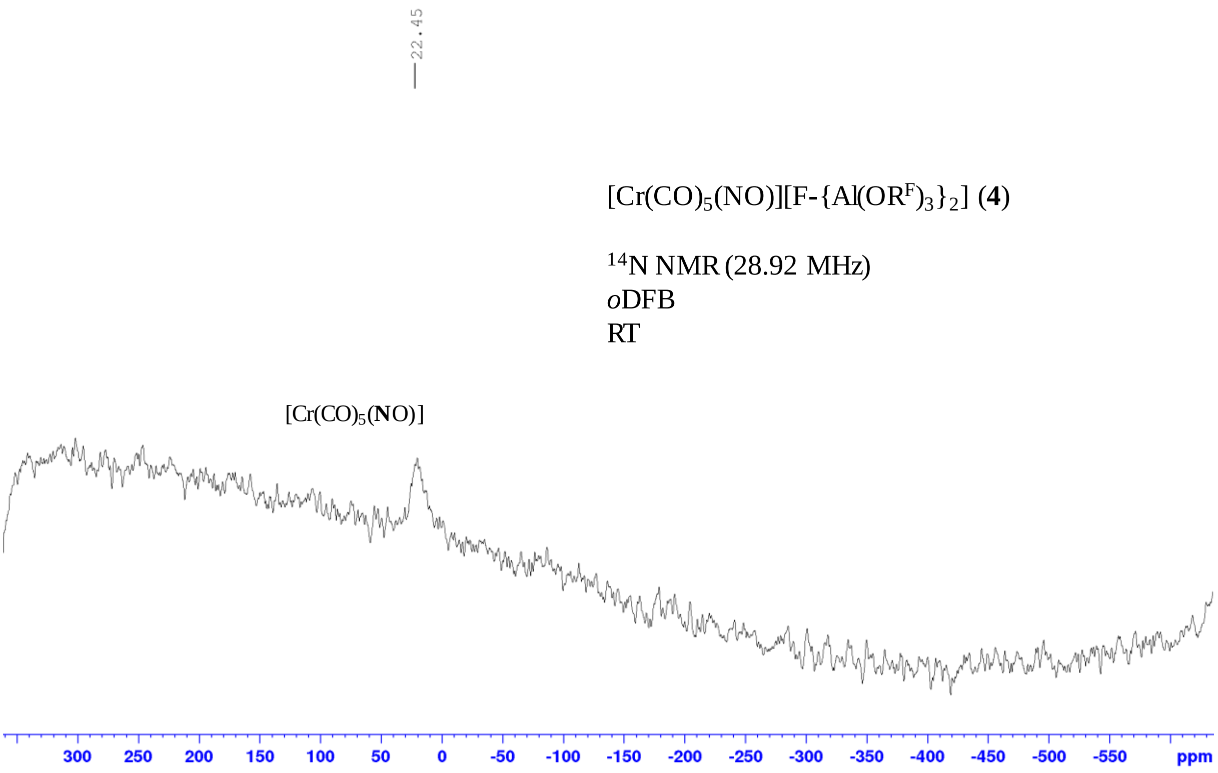

Supplementary Figure 24. ^{14}N NMR spectrum (28.92 MHz, *o*DFB, RT) of **4**.

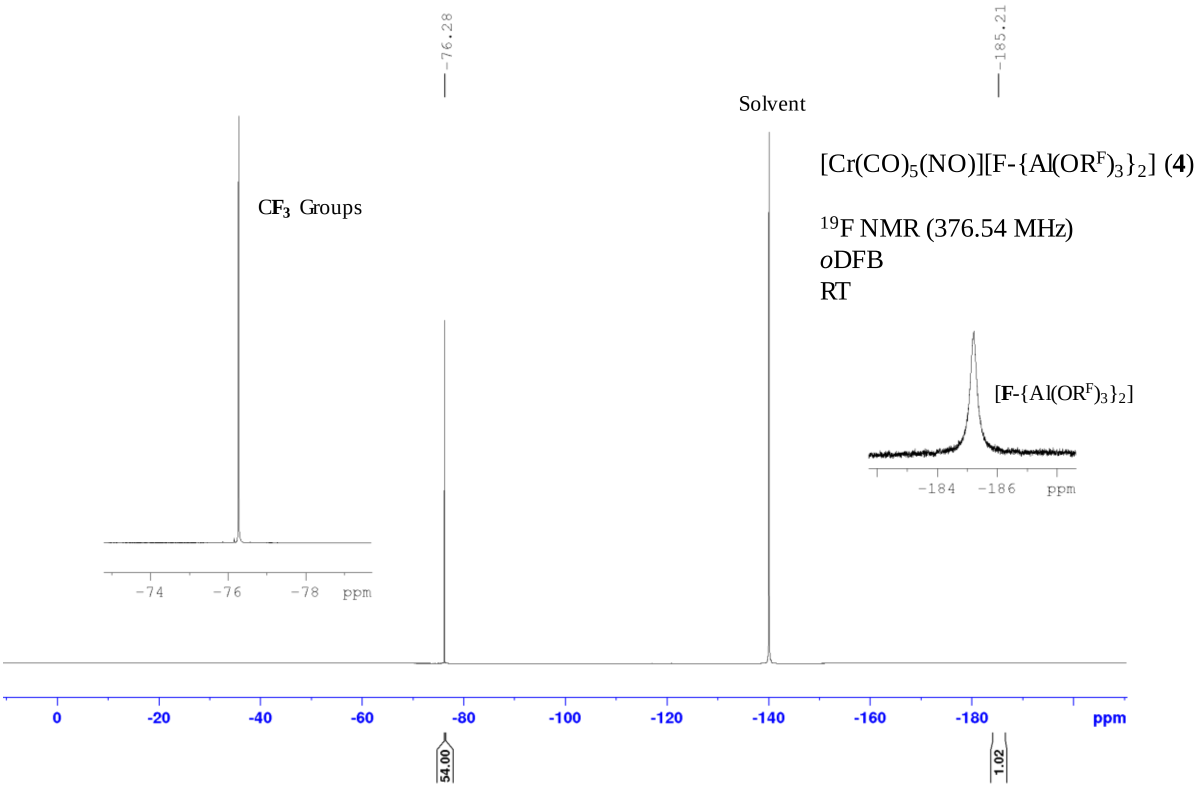

Supplementary Figure 25. ^{19}F NMR spectrum (376.54 MHz, *o*DFB, RT) of **4**.

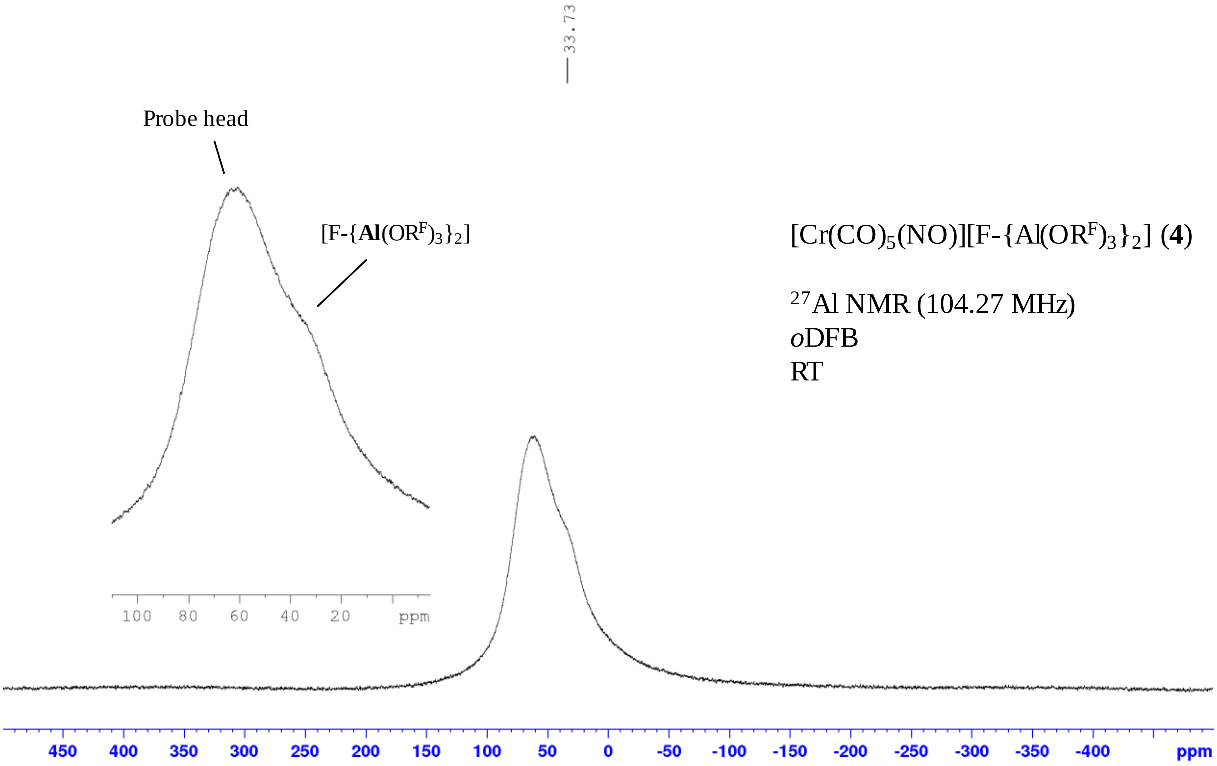

Supplementary Figure 26. ^{27}Al NMR spectrum (104.27 MHz, *o*DFB, RT) of **4**.

6. Evans NMR Method

Paramagnetic shifts via Evans' method were measured in a 1:6 solution of CD_2Cl_2/1,2-$F_2C_6H_4$ or CH_2Cl_2/1,2-$F_2C_6H_4$ respectively with the ^{1}H NMR shift of dichloromethane (δ = 5.32 ppm) as standard in an open 3 mm thick NMR tube inside the J. Young NMR tube containing the solution of the paramagnetic substance in the same solvent mixture. The average μ_{eff} of three different measurements was calculated. The formulae for the calculation of χ_{m} and μ_{eff} were taken from the literature[7,8] (Supplementary Equation 1).

$$\chi_{\mathrm{m}} = \frac{3\Delta\upsilon}{4\pi\upsilon_0 c}\,;\ \mu_{\mathrm{eff}} = \sqrt{8T\chi_m M} \qquad (1)$$

with Δν: paramagnetic shift (in Hz), ν_0: spectrometer frequency (Hz), c: concentration ($g_{\mathrm{substance}}$/mL_{solvent}), T: temperature (K), M: molar mass of the substance.

The summary of the results are shown in Supplementary Table 1, the respective NMR spectra are shown in Supplementary Figure 27 to Supplementary Figure 32.

Supplementary Table 1. Paramagnetic shifts calculated via Evans' method.

$[Cr(CO)_6][Al(OR^F)_4]$ (**1**)	$\Delta\nu$ [Hz]	ν_0 [10^6 Hz]	C [g/mL]	T [K]	M [g/mol]	μ_{eff} [μ_B]
**A**	71	300.17	0.04363	298.15	1187.2	1.91
**B**	85	400.17	0.03387	298.15	1187.2	2.06
**C**	112	300.17	0.05483	298.15	1187.2	2.14
**Avg. μ_{eff}**						**2.04**
$[Cr(CO)_6][F\text{-}\{Al(OR^F)_3\}_2]$ (**2**)						
**A**	90	300.17	0.06881	298.15	1703.2	2.06
**B**	48	300.17	0.04121	298.15	1703.2	1.94
**C**	72	400.17	0.03677	298.15	1703.2	2.18
**Avg. μ_{eff}**						**2.06**

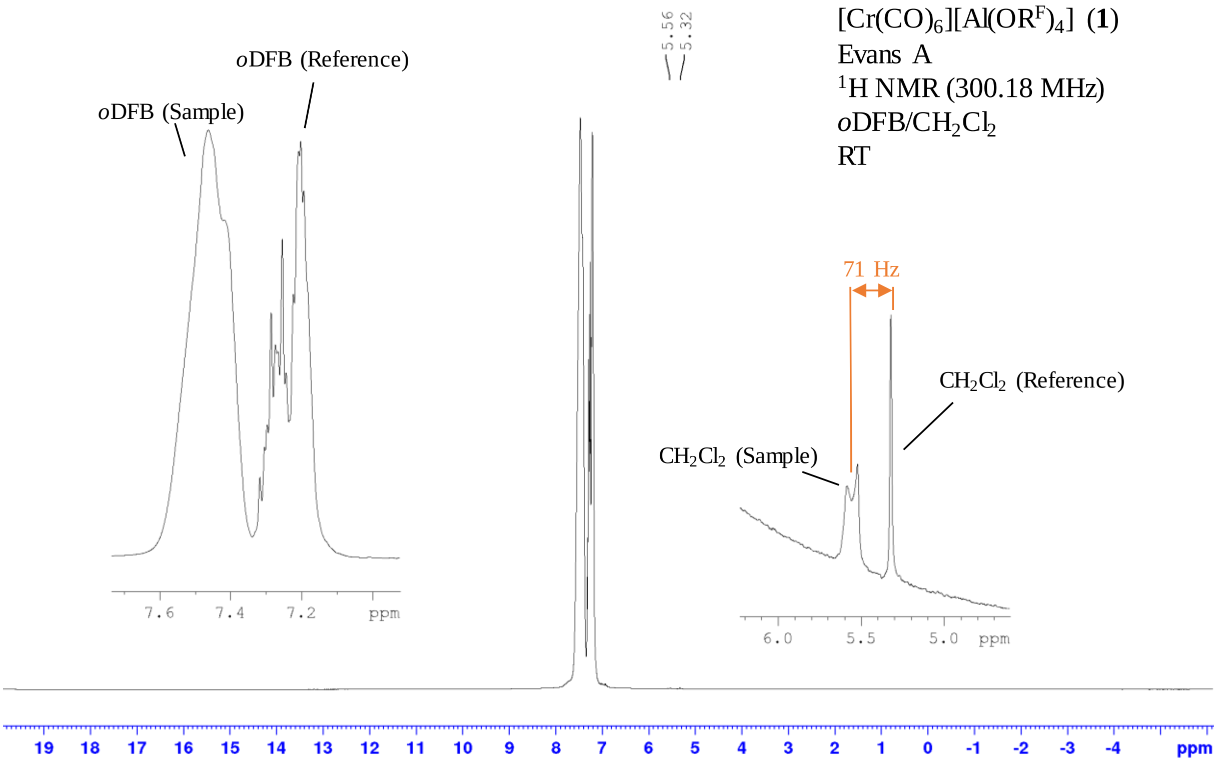

Supplementary Figure 27. Evans' measurement A: ^{1}H NMR spectrum (300.18 MHz, *o*DFB/CH_2Cl_2, RT) of **1**.

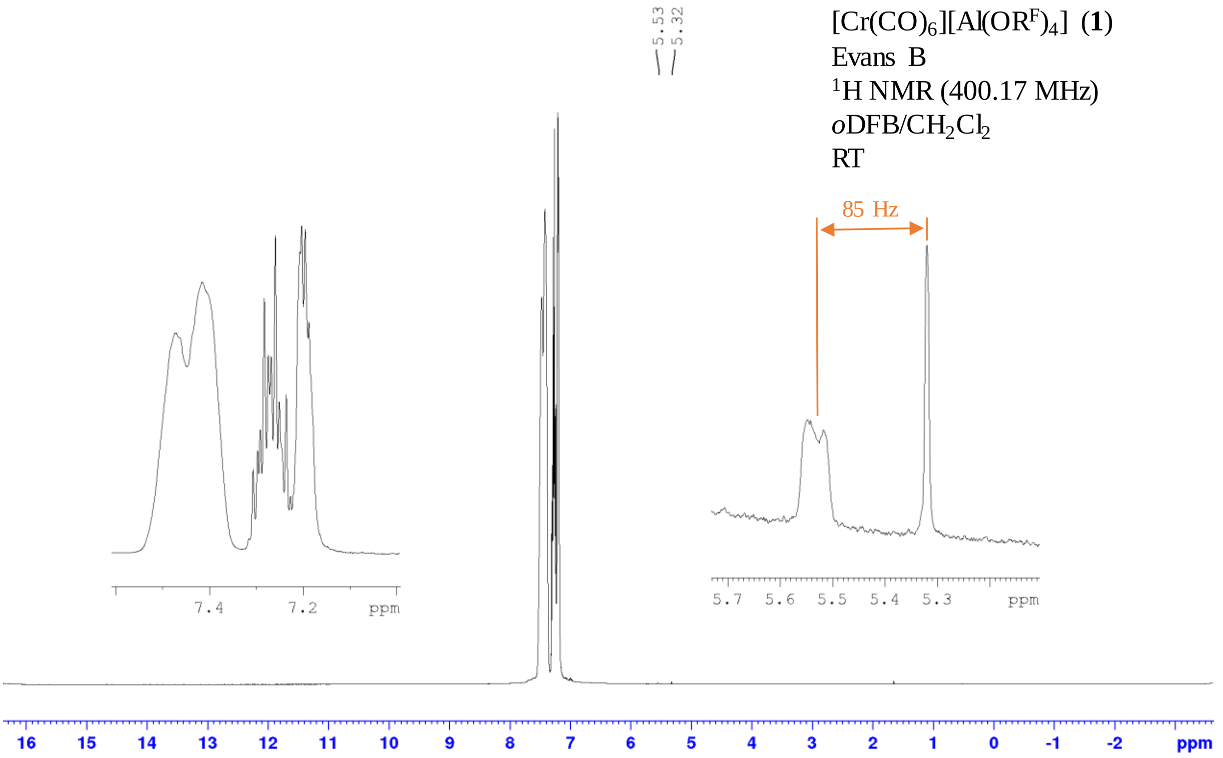

Supplementary Figure 28. Evans' measurement B: ^{1}H NMR spectrum (400.17 MHz, *o*DFB/CH_2Cl_2, RT) of **1**.

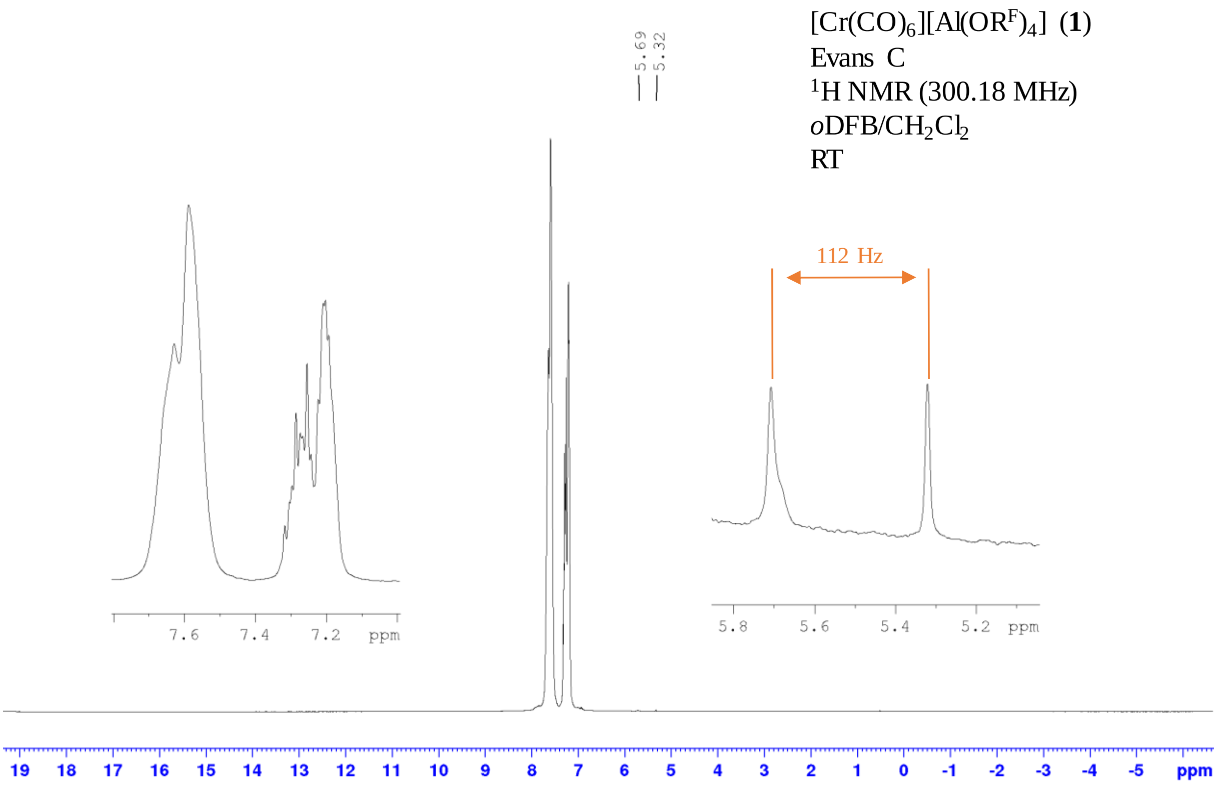

Supplementary Figure 29. Evans' measurement C: ^{1}H NMR spectrum (300.18 MHz, *o*DFB/CH_2Cl_2, RT) of **1.**

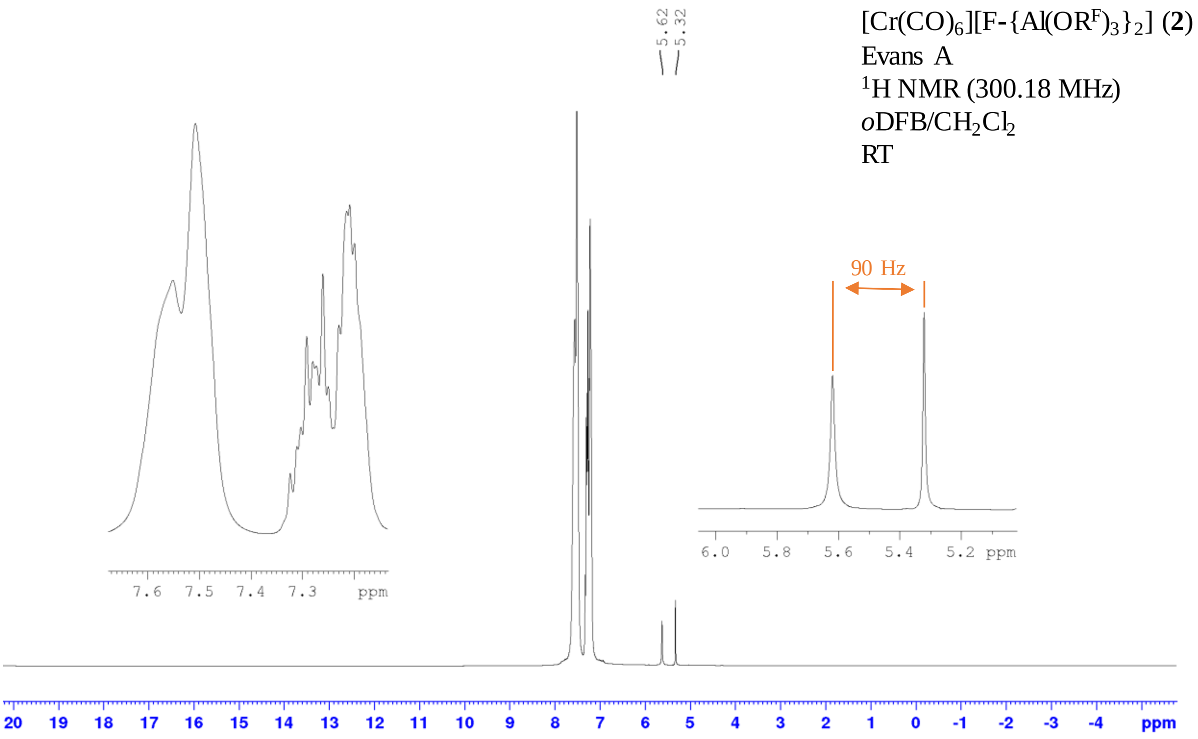

Supplementary Figure 30. Evans' measurement A: ^{1}H NMR spectrum (300.18 MHz, *o*DFB/CH_2Cl_2, RT) of **2.**

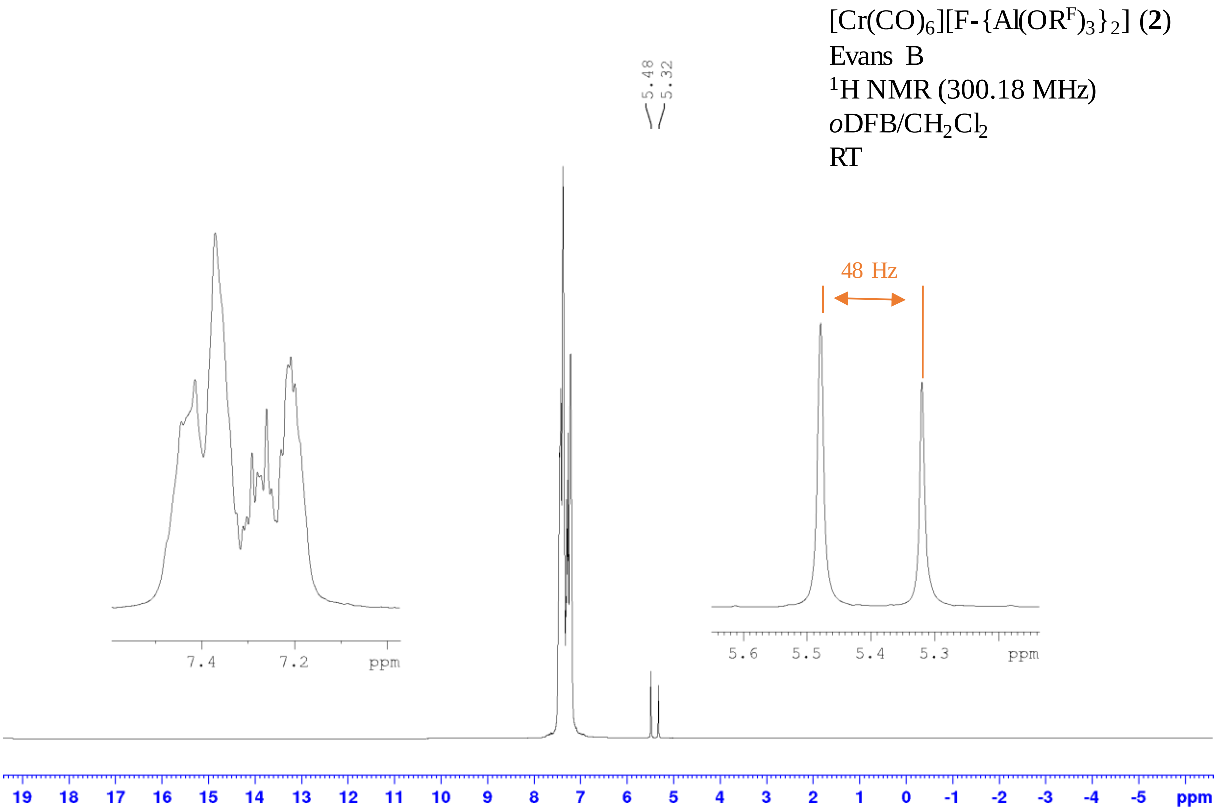

Supplementary Figure 31. Evans' measurement B: ^{1}H NMR spectrum (300.18 MHz, *o*DFB/CH_2Cl_2, RT) of **2.**

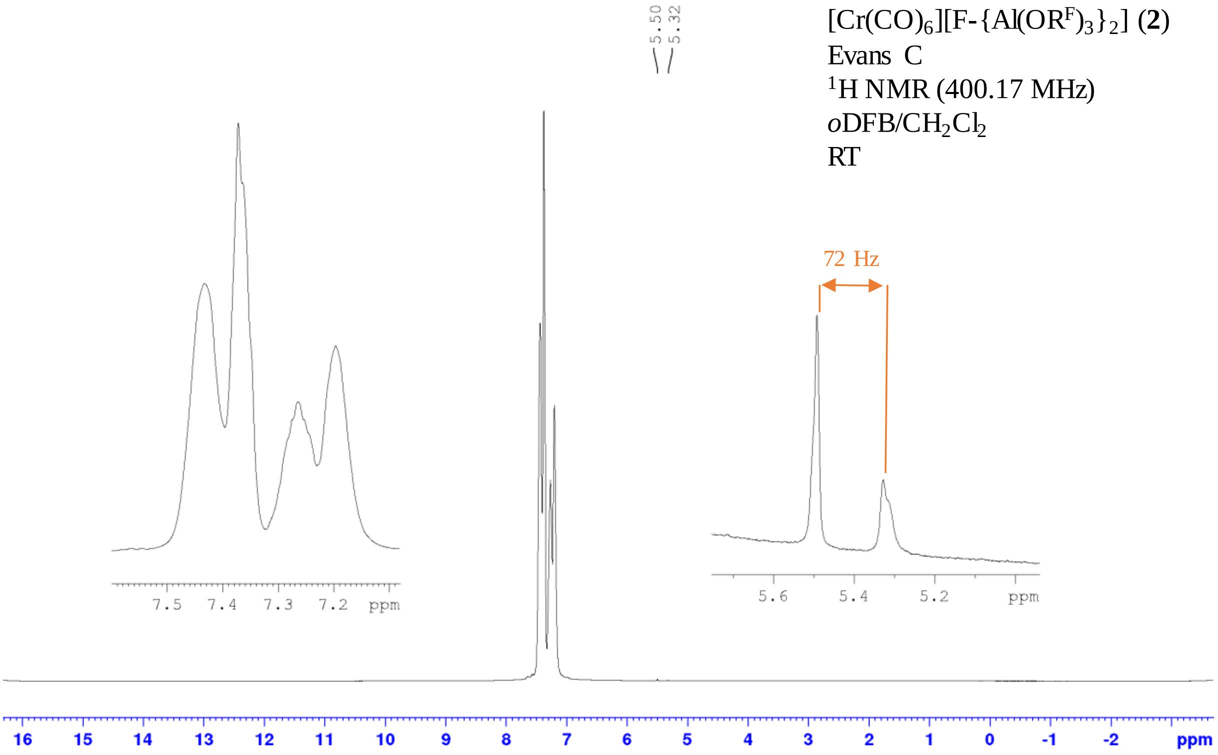

Supplementary Figure 32. Evans' measurement C: ^{1}H NMR spectrum (400.17 MHz, *o*DFB/CH_2Cl_2, RT) of **2.**

7. Vibrational Analysis

The full vibrational analysis of compounds 1 to 4 is shown in Supplementary Table 2 and Supplementary Table 3.

Supplementary Table 2. Full assignment of all IR and Raman vibrations for complexes **1** and **2**.

$[Cr(CO)_6][Al(OR^F)_4]$ (1)		$[Cr(CO)_6]$ $[F\text{-}\{Al(OR^F)_3\}_2]$ (2)		$[Cr(CO)_6]^+$ calcd.[a)]		$[Al(OR^F)_4]^-$ [47]		$[F\text{-}\{Al(OR^F)_3\}_2]^-$ [48]	Assignment [47,48]
IR	Raman	IR	Raman	IR[b)]	Raman[b)]	IR	Raman	IR	
					85 (10)				E_g
	173 (vvw)						-		-
	234 (vvw)		234 (vvw)				233 (w)		-
	288 (w)		291 (vw)				291 (w)		C–C
	332 (m)		328 (ms)				322 (s)		C–C, Al–O
					356 (25)				A_{1g}
	368 (vvw)		370 (vw)				369 (w)		C–C, C–F, Al–O
				391 (15)					E_u
				526 (85)					A_{2u}
	538 (vw)		539 (vw)				538 (mw)		C–C, C–O
560 (vw)	563 (vvw)					559 (mw)	561 (w)		Al–O, C–C
571 (vvw)	572 (vvw)	568 (vw)	571 (vw)			573 (w)	572 (w)	572 (m)	Al–O, C–C
				621 (126)					E_u
		639 (w)						*639 (m)*	*Al–F–Al*
726 (vvs)		726 (vvs)				726 (ms)	-	728 (s)	C–C, C–O
	747 (w)	751 (vvw)	753 (ms)				744 (ms)	-	-
756 (vvw)		760 (vvw)				*755 (w)*	-	-	*C–C, C–O*
	798 (w)						799 (ms)		-
			818 (vw)						-
831 (vw)						*830 (m)*			*Al–O, C–C*
		865 (w)				-	-	*865 (w)*	*Al–O, Al–F–Al*
969 (vvs)	977 (vvw)	972 (vvs)	980 (vvw)			975 (vs)	975 (mw)	975 (s)	C–C, C–F
			1131 (vvw)			-	1133 (mw)		C–C, C–F
1161 (ms)	1163 (vvw)					-	-		
						1176 (ms)	1173 (mw)		
		1184 (s)	1180 (vvw)			-	-	1183 (m)	C–C, C–F
1208 (vvs)		1216 (vvs)				1223 (vs)		1218 (s)	C–C, C–F
1239 (s)	1235 (vvw)	1247 (vvs)	1244 (vvw)			1236 (vs)	1237 (mw)	1249 (s)	C–C, C–F
1272 (ms)	1272 (vvw)		1281 (vvw)			1274 (s)	1276 (mw)	1268 (m)	C–C, C–F
1298 (mw)	1304 (vvw)	1300 (mw)	1304 (vvw)			1299 (s)	-	1301 (m)	C–C, C–F
1352 (vw)		1355 (vw)				1349 (ms)	1353 (w)	1355 (m)	C–C, C–F
~~1508 (vvw)~~		~~1509 (vvw)~~							~~*o*DFB~~
~~1843 (vvw)~~		~~1841 (vvw)~~							~~ν N–O A_1~~
	2128 (br, vs)		2126 (br, vs)		2074 (413)				ν C–O E_g
2094 (s)		2096 (s)		2081 (999)					ν C–O E_u
				2084 (816)					ν C–O A_{2u}
	2175 (s)		2173 (s)		2157 (208)				ν C–O A_{1g}

[a)] BP86-D3BJ/def2-TZVPP, D_{3d} symmetry, no scale factor was applied. w: weak, m: medium, s: strong, v: very, sh: shoulder. [b)] Raman and IR intensities were calculated with the Gaussian software. The assignments of the respective anion bands and their intensities are based on $[N(Bu)_4][Al(OR^F)_4]$ (IR and Raman) in ref.[47] and $[CBr_3][F\text{-}\{Al(OR^F)_3\}_2]$ (IR only) in ref.[48]. The characteristic IR bands that are suited best for differentiation between $[Al(OR^F)_4]^-$ and $[F\text{-}\{Al(OR^F)_3\}_2]^-$ are given in italics; in Raman spectroscopy, the difference is subtler and shows best in the (roughly) 1:1 intensity of the bands at 747 and 798 cm^{-1} ($[Al(OR^F)_4]$) compared to a (roughly) 1:3 intensity for the bands at 753 and 818 cm^{-1} ($[F\text{-}\{Al(OR^F)_3\}_2]^-$).

Bands of trace impurities are ~~crossed-out~~: 1843/1841 cm^{-1} belongs to traces of the $[Cr(CO)_5(NO)]^+$ cation, 1508/1509 cm^{-1} belongs to traces of *o*DFB. Note that the Cr–C vibrations cannot be assigned unambiguously due to overlap with the anion bands.

The plots of the calculated IR and Raman spectra for $[Cr(CO)_6]^+$ are shown in Supplementary Figure 38 and Supplementary Figure 39 .

Supplementary Table 3. Full assignment of all IR and Raman vibrations for complexes **3** and **4**.

**[Cr(CO)$_5$(NO)] [Al(OR^F)$_4$] (3)**		**[Cr(CO)$_5$(NO)] [F-{Al(OR^F)$_3$}$_2$] (4)**		**[Cr(CO)$_5$(NO)]$^+$ calcd.**[b)]		**[Al(OR^F)$_4$]$^{-}$**[47]		**[F-{Al(OR^F)$_3$}$_2$]$^{-}$**[48]	**Assignment**[47,48]
IR	Raman	IR	Raman	IR	Raman	IR	Raman	IR	
					89 (10)				B_1
					93 (10)				*E*
	234 (vvw)		233 (vvw)				233 (w)		-
	290 (vvw)		293 (vvw)				-		-
							291 (w)		C–C
	332 (vw)		332 (vw)				322 (s)		C–C, Al–O
				351 (10)					A_1
					367 (20)				A_1
	369 (vvw)		370 (vvw)				369 (w)		C–C, C–F, Al–O
				417 (22)					*E*
	487 (vvw)		487 (vvw)		504 (16)		-		ν Cr–N A_1
				532 (26)					*E*
	539 (vvw)		539 (vvw)				538 (mw)		C–C, C–O
561 (vw)	562 (vvw)	568 (vw)				559 (mw)	561 (w)	572 (m)	Al–O, C–C
571 (vvw)			572 (vvw)			573 (w)	572 (w)		Al–O, C–C
640 (vw)	640 (vw)	§*638 (mw)*	639 (vw)	660 (133)	660 (19)	-	-	*639 (m)*	ν Cr–N A_1; *Al–F–Al* (**4**)
657 (w)		657 (w)		693 (99)		-	-	-	δ Cr–C *E*
727 (vs)		727 (vvs)				726 (ms)	-	728 (s)	C–C, C–O
	747 (vw)		753 (w)			-	744 (ms)	-	-
756 (vvw)		760 (vvw)				*755 (w)*	-	-	*C–C, C–O*
	797 (vw)					-	799 (ms)	-	-
			818 (vvw)					-	
832 (vw)						*830 (m)*	-	-	*Al–O, C–C*
866 (vvw)		*865 (w)*				-	-	*865 (w)*	*Al–O, Al–F–Al*
971 (vvs)	977 (vvw)	973 (vvs)	980 (vvw)			975 (vs)	975 (mw)	975 (s)	C–C, C–F
1140 (vw)			1130 (vvw)			-	1133 (mw)	-	C–C, C–F
1162 (mw)		1185 (s)	1180 (vvw)			1176 (ms)	1173 (mw)	1183 (m)	C–C, C–F
1210 (vvs)		1216 (vvs)				1223 (vs)	-	1218 (s)	C–C, C–F
1239 (ms)		1248 (vvs)	1244 (vvw)			1236 (vs)	1237 (mw)	1249 (s)	C–C, C–F
1251 (ms)						-	-	-	
1273 (ms)	1275 (vvw)	1278 (m)	1281 (vvw)			1274 (s)	1276 (mw)	1268 (m)	C–C, C–F
1299 (mw)	1304 (vvw)	1300 (mw)	1305 (vvw)			1299 (s)	-	1301 (m)	C–C, C–F
1353 (vw)		1355 (vw)				1349 (ms)	1353 (w)	1355 (m)	C–C, C–F
~~1821 (vvw)~~		~~1809 (vvw)~~							
1841 (mw)	1843 (vvw)	1839 (ms)	1842 (vvw)	1899 (1052)	1899 (39)				ν N–O A_1
2074 (vvw)	2076 (vvw)	2072 (vvw)	2074 (vvw)	2048	2048				ν ^{13}C–O E
		2099 (m)*							ν C–O*
2108 (ms)	2112 (vw)	2107 (vs)	2110 (vvw)	2085 (1055)	2085 (5)				ν C–O *E*
2127 (vvw)	2127 (vvs)		2125 (vvs)		2097 (340)				ν C–O B_2
2164 (vvw)	2164 (m)	2162 (vvw)	2163 (m)	2123 (230)	2123 (217)				ν C–O A_1
	~~2175 (vw)~~		~~2173 (vw)~~						~~ν C–O A_{1g} of **1** and **2**~~
2184 (vw)	2185 (m)	2183 (vw)	2184 (m)	2161 (91)	2161 (177)				ν C–O A_1

[b)] BP86-D3BJ/def2-TZVPP, C_{4v} symmetry, no scale factor was applied. w: weak, m: medium, s: strong, v: very, sh: shoulder. The assignments of the respective anion bands and their intensities are based on $[N(Bu)_4][Al(OR^F)_4]$ (IR and Raman) in ref.[47] and $[CBr_3][F\text{-}\{Al(OR^F)_3\}_2]$ (IR only) in ref.[48]. The characteristic IR bands that are suited best for differentiation between $[Al(OR^F)_4]^-$ and $[F\text{-}\{Al(OR^F)_3\}_2]^-$ are given in italics; in Raman spectroscopy, the difference is subtler and shows best in the (roughly) 1:1 intensity of the bands at 747 and 798 cm^{-1} ($[Al(OR^F)_4]$) compared to a (roughly) 1:3 intensity for the bands at 753 and 818 cm^{-1} ($[F\text{-}\{Al(OR^F)_3\}_2]^-$).

Bands of trace impurities are ~~crossed out~~: 2174/2173 cm^{-1} belong to a small contamination with residual $[Cr(CO)_6]^+$, 1821/1809 cm^{-1} might belong to the isotope-shifted A_1 vibration, since it appears also in the spectra of single crystals – however, since the shift is different for **3** and **4**, we stated it as an unknown impurity.

The additional bands in the carbonyl region is due to minor interactions with the counter ion (which distorts the ideal C_{4v} structure). Note that here the Cr–N and Cr–C vibrations at 657 cm^{-1} and 640 cm^{-1} (§with overlapping Al–F–Al vibration of **4**) (IR) as well as 640 cm^{-1} and 487 cm^{-1} (Raman) can be assigned unambiguously (Supplementary Figure 33).*) Probably removal of the degeneracy of the *E*-mode in **4** (2107 cm^{-1}).

The plots of the calculated IR and Raman spectra for $[Cr(CO)_5(NO)]^+$ are shown in Supplementary Figure 40 and Supplementary Figure 41.

Assignable Cr–N vibrations

Unambiguously assignable Cr–N vibrations are shown in Supplementary Figure 33.

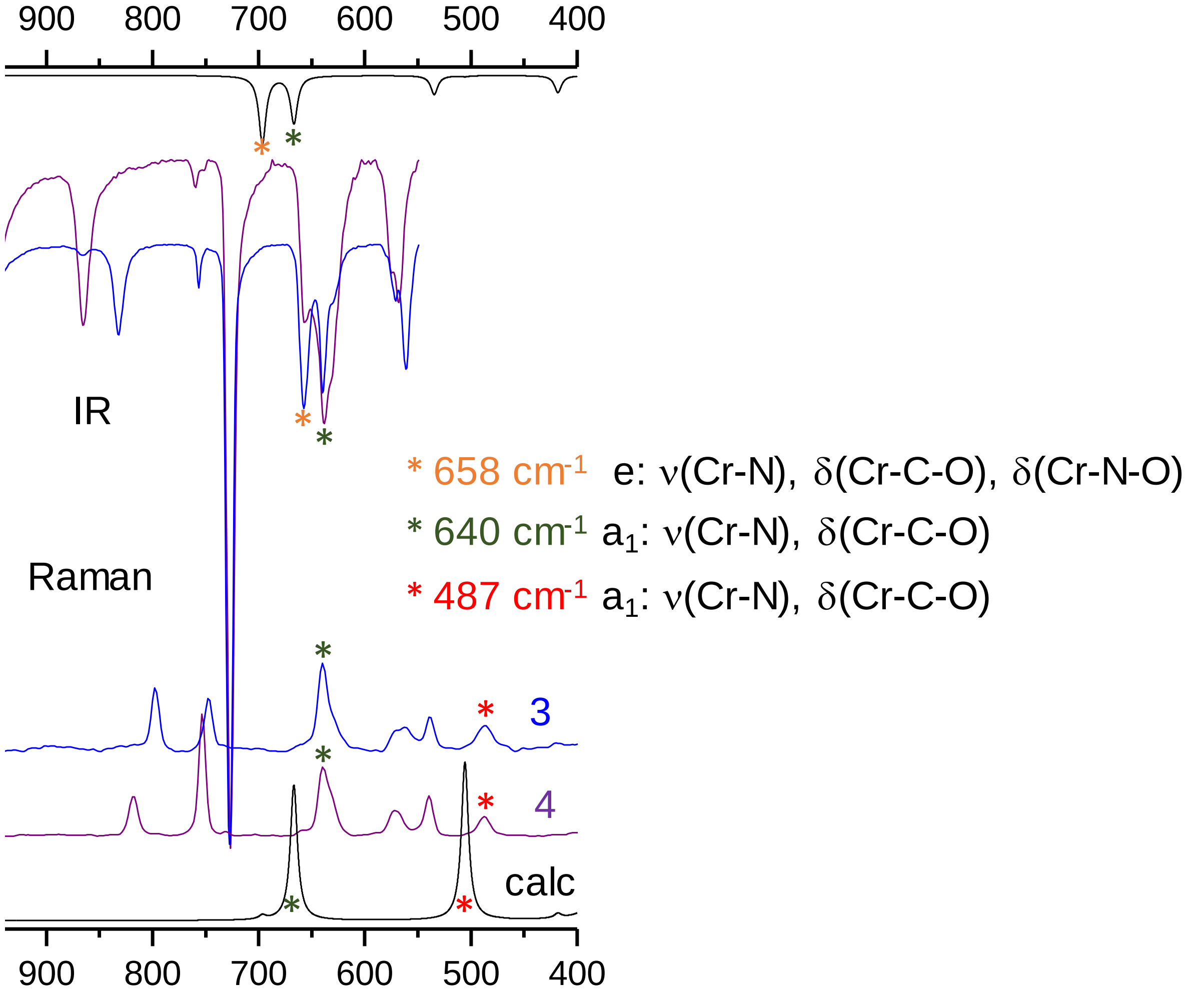

Supplementary Figure 33. Unambiguously assignable characteristic Cr–N and Cr–C vibrations of complexes **3** and **4**.

Comparison of $[Cr(CO)_6]^{•+}$ and $V(CO)_6$

Supplementary Figure 34 shows the comparison of IR and Raman spectra of $[Cr(CO)_6]^{•+}$ (this work) and the published spectra by Willner *et al.*[49] for the isoelectronic $V(CO)_6$. The extremely similar band pattern underlines the similarity between both isoelectronic species, both also showing the aforementioned fluctionality of a D_{3d} symmetric ground state.

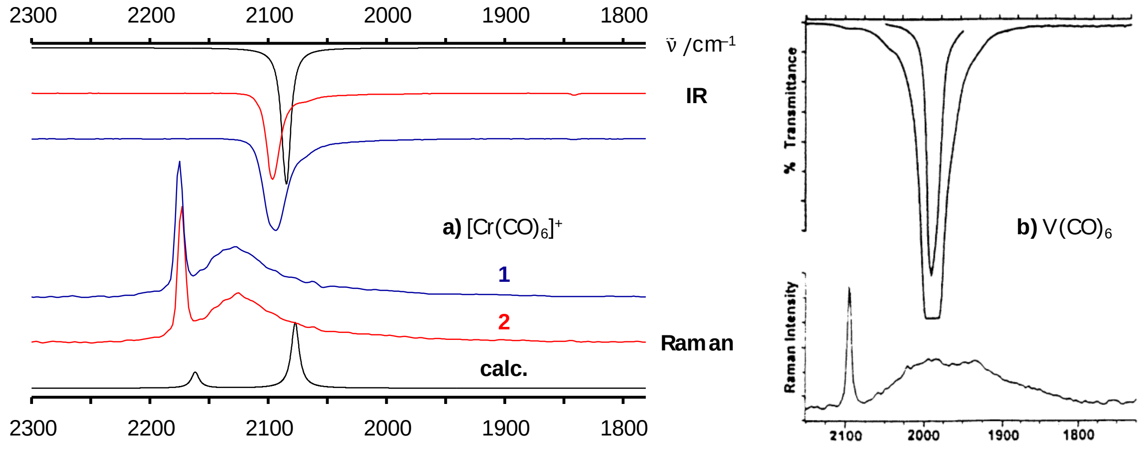

Supplementary Figure 34. Comparison between the vibrational spectra of $[Cr(CO)_6]^{•+}$ (this work, left) and the isoelectronic $V(CO)_6$ by Willner *et al*.[49] (IR gas-phase spectrum and Raman spectrum diluted in benzene at room temperature; right) supporting a fluctuating D_{3d} structure.

8. Gas Phase IR Spectra

Gas phase IR spectra (Supplementary Figure 35) were measured by two different methods to minimize undesired solvent bands of CH_2Cl_2. The IR gas cell is shown in Supplementary Figure 36.

a) The reaction gas phase was expanded into an evacuated cooling trap at about –110 °C (l. N_2/ethanol) to separate CH_2Cl_2 vapours into another cooling trap at –196 °C (l. N_2) to trap the NO. Then the NO containing cooling trap was separated and allowed to warm up and expand into the evacuated IR gas cell.

b) The reaction mixture after 14 d was cooled down to about –130 °C (l. N_2/*n*-pentane) until the solvent was frozen solid. Then the gas phase of the reaction vessel was expanded into the evacuated IR cell.

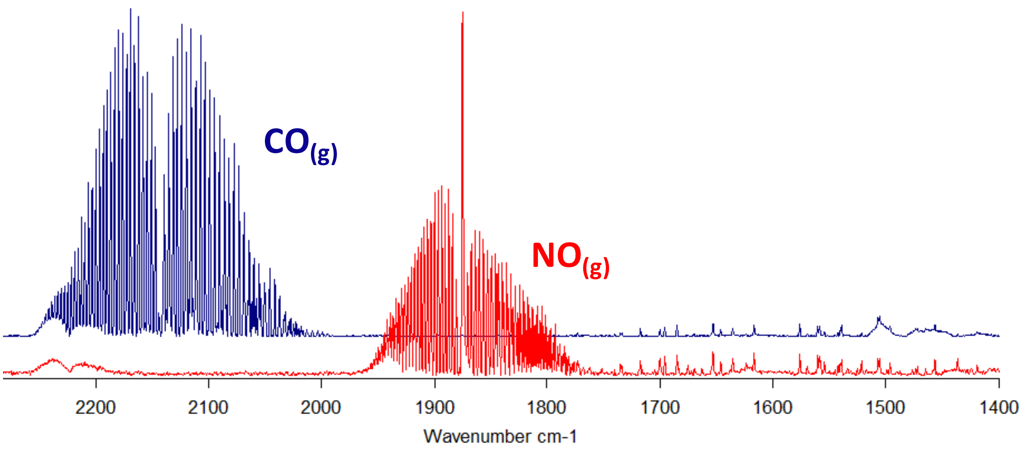

Supplementary Figure 35. Gas phase IR spectra of the reaction mixtures from the synthesis of $[Cr(CO)_6]^+[Al(OR^F)_4]^-$ after 20 min (bottom spectrum, red) and the synthesis of $[Cr(CO)_5(NO)]^+[Al(OR^F)_4]^-$ after 14 d (top spectrum, blue).

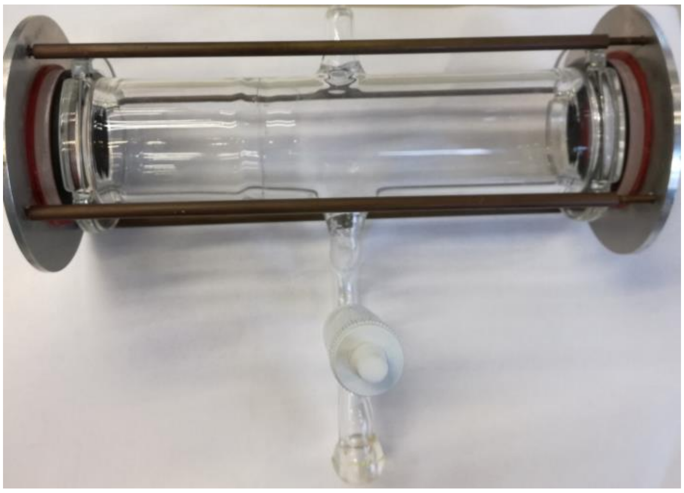

Supplementary Figure 36. IR gas cell.

9. EPR Spectroscopy

Functional Evaluation

Before performing symmetry restricted structure optimizations of the $[Cr(CO)_6]^{•+}$ cation we evaluated the reasonability of different density functionals, namely M06-L[50], TPSS[34], TPSSh[33] and BP86[51], the latter three extended by Grimme's dispersion correction and Becke-Johnson Damping. The def2-TZVPP basis set was used throughout. These functionals were used for structure optimizations (C_1 symmetry). We note, that for all investigated functionals the optimizations converged to a D_{3d} symmetric minimum structure. The so obtained structures served for DLPNO-CCSD(T) single-point energy calculations using the ORCA 4.0.1 program code without symmetry restrictions. It turned out, that structures obtained with the hybrid-meta-GGA functional TPSSh were lowest in energy on the DLPNO-CCSD(T) level of theory (Supplementary Table 4).

Supplementary Table 4. Single-point energies (DLPNO-CCSD(T)/def2-TZVPP) of D_{3d} symmetric structures of the $[Cr(CO)_6]^+$ cation optimized by different density functionals. The energies ΔE are given relative to the lowest energy of the TPSSh structure.

	M06-L	TPSS (D3BJ)	BP86 (D3BJ)	TPSSh (D3BJ)
ΔE/(kJ mol^{-1})	0.52	1.16	3.41	0.00

Group Theory

In order to understand the obtained EPR-spectra and the underlying dynamic of the $[CrCO_6]^{•+}$ cation we optimized its structure applying different point groups. Removal of one electron from the neutral $Cr(CO)_6$ d^6 complex breaks down its wave function symmetry resulting in an electronic B_{1g} state and making an O_h symmetric structure impossible. Following the normal vibrational modes of the O_h structure leads to the point groups the cationic d^5 species may take. The E_g symmetric vibrations according to an axial compression along the z-axis and stretching along the x- and y-axis (and vice versa) lead to D_{4h} symmetric structures. We found (TPSSh-D3BJ/def2-TZVPP) a D_{4h} symmetric structure, B_{1g} electronic ground state, with compressed z-axis to be a local minimum whose electronic energy is +1.63 kJ mol^{-1} (DLPNO-CCSD(T)/def2-TZVPP) above the global minimum.

Following the T_{2g} bending modes leads to a D_{2h} symmetric distortion. However, this D_{2h}^* structure 2.04 kJ mol^{-1} above the global minimum is not a stationary point (*cf.* asterisk) and shows a gradient linked to stretchings along the principal axes, i.e. an admixture with the E_g

symmetric stretching modes. The relaxed stationary D_{2h} structure with B_{1g} electronic ground state shows one imaginary frequency exposing it to be a transition state at +0.85 kJ mol^{-1} connecting two equivalent D_{3d} symmetric structures with an A_{1g} electronic ground state. The D_{3d} structure arises from a linear combination of all three T_{2g} bending modes of the parent O_h structure and was identified to be the global minimum structure.

We note, that the A_g electronic state of D_{2h} point group is identical with the above mentioned B_{1g} state in D_{4h}. Thus, probably the local D_{4h} minimum may be accessible via electronic transitions during distortions of the ground state D_{3d} structure via the D_{2h} symmetric transition state.

In addition, we identified a C_{2h} (A_g) symmetric local minimum (not depicted in Supplementary Figure 37) corresponding to a combination of all T_{2g} and E_g vibrational modes. It deviates only marginally from the D_{3d} structure and thus is only +0.31 kJ mol^{-1} in electronic energy above the global minimum.

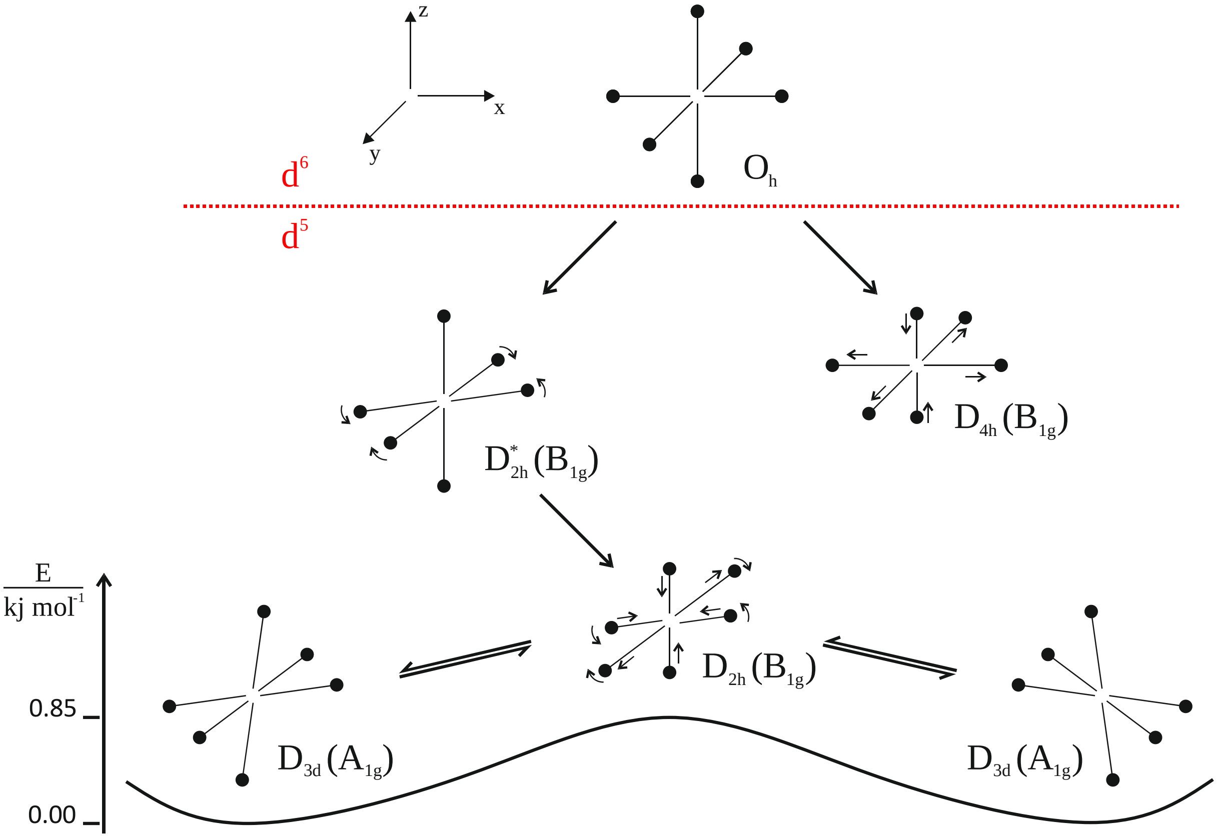

Supplementary Figure 37. Schematic representation of point groups the $[Cr(CO)_6]^{\bullet +}$ cation may take and their relation to the parent O_h symmetric $Cr(CO)_6$. The interconversion of equivalent D_{3d} symmetric structures via the D_{2h} transition state at higher temperatures leads to an exchange of the molecular principal axis, i.e. the g anisotropy is averaged on the EPR time scale.

The thermal fluctuation between equivalent D_{3d} structures *via* the D_{2h} transition state is accompanied with an exchange of the principal axes of the $[Cr(CO)_6]^{•+}$ cation. Hence, fast fluctuation prevents detection of the g-tensor anisotropy on the EPR time scale ($g_{\perp} = 2.185$, $g_{\parallel} = 1.947$, $g_{iso} = 2.106$). This results in the coalescent EPR spectrum, i.e. averaged over all three spatial axes, we obtained at 100 K in contrast to the 4 K measurement showing only the signatures of the D_{3d} ground state.

Hyperfine Coupling

The calculated hyperfine coupling constants of ^{53}Cr in $[Cr(CO)_6]^{•+}$ are shown in Supplementary Table 5.

Supplementary Table 5. Hyperfine Coupling constants in MHz (mT) of ^{53}Cr in $[Cr(CO)_6]^{•+}$ calculated at the TPSSh/ZORA-def2-TZVPP level of theory (for scalar relativistic ZORA calculations the def2-TZVPP basis set recontracted by D. A. Pantazis was used) including isotropic, dipolar and 2nd order contributions from spin orbit coupling. We made use of the Zero Order Regular Approximation (ZORA)[52] and applied a Gaussian finite nucleus model[53]; *iso = isotropic.*

	x	y	z	*iso*
D_{3d}	-4.31 (-0.16)	39.53 (1.30)	39.54 (1.30)	*24.92 (0.85)*
D_{4h}	10.96 (0.32)	10.96 (0.32)	62.11 (2.52))	*29.77 (0.96)*

XYZ-Coordinates of Optimized Structures

All coordinates (Supplementary Data 1) are given in Ångström. A given point group indicates symmetry restrictions made for the structure optimization.

Supplementary Data 1. Information on the atomic coordinates $[Cr(CO)_6]^{+}$.

$[Cr(CO)_6]^{•+}$ M06-L

```
Cr  -0.00172881229403    -0.00000077435582    -0.00000059255038
C    1.45791443823981     0.84451732385296    -1.05398409546532
O    2.29056405816007     1.33232564049600    -1.64060048426715
C    1.45791455196409    -0.84451765882305     1.05398432232663
O    2.29056403565950    -1.33232403210469     1.64060245246521
C   -1.46209777554421    -0.84488433912572     1.05338247919505
O   -2.29491191975216    -1.33281006106499     1.63962464693159
C   -1.46209814104130     0.84488431653632    -1.05338238385179
O   -2.29491223297483     1.33281241651990    -1.63962275123244
C    0.00140286706382     1.68803696294950     1.05008027361462
O    0.00799274317260     2.65375856122732     1.63556121936135
C    0.00140302010543    -1.68803831543978    -1.05008217907317
O    0.00799316724123    -2.65376004066797    -1.63556290745419
```

$[Cr(CO)_6]^{\bullet+}$ TPSS (D3BJ)

Cr	-0.00176354403789	-0.00000029237989	-0.00000050334991
C	1.43997387696736	0.83508298776808	-1.05879420594979
O	2.27268260531531	1.32299290787730	-1.65895003764923
C	1.43997361666532	-0.83508292133695	1.05879403408460
O	2.27268222566009	-1.32299213083920	1.65895059967334
C	-1.44430209108314	-0.83536860384086	1.05805395824889
O	-2.27728282342698	-1.32339176997282	1.65770412698598
C	-1.44430229719773	0.83536879245988	-1.05805398677128
O	-2.27728309349410	1.32339300581885	-1.65770321325069
C	0.00156170976816	1.66809536738832	1.05545797114075
O	0.00824874638401	2.63478221947750	1.65309074533049
C	0.00156191812613	-1.66809623484740	-1.05545856684236
O	0.00824915035347	-2.63478332757280	-1.65309092165079

$[Cr(CO)_6]^{\bullet+}$ BP86 (D3BJ)

Cr	-0.00108193827643	-0.00000003774586	-0.00000001680537
C	1.42878876782816	0.82665176985051	-1.05259641450257
O	2.26429955319072	1.31395264871178	-1.65393752786568
C	1.42878875997201	-0.82665184513319	1.05259639817745
O	2.26429954280894	-1.31395271330183	1.65393753581533
C	-1.43133916440627	-0.82694343705710	1.05210693272432
O	-2.26699650052138	-1.31448276196102	1.65303032661481
C	-1.43133914132449	0.82694345256530	-1.05210686683029
O	-2.26699645210755	1.31448298060838	-1.65303013044643
C	0.00064969158924	1.65262853966747	1.05068881877259
O	0.00513840903592	2.62081678038757	1.65050781022921
C	0.00064980201034	-1.65262857962956	-1.05068891907656
O	0.00513867020077	-2.62081679696247	-1.65050794680681

$[Cr(CO)_6]^{\bullet+}$ TPSSh (D3BJ)

Cr	-0.00198775920301	-0.00000009930945	-0.00000008659186
C	1.44282157372714	0.83711956793837	-1.06101148297952
O	2.27057831681706	1.32277509570077	-1.65748663600178
C	1.44282143834167	-0.83711968852719	1.06101133483033
O	2.27057817757719	-1.32277519665700	1.65748652016147
C	-1.44757901843402	-0.83736786873882	1.06039021993630
O	-2.27557934439513	-1.32305362786048	1.65646645952626
C	-1.44757897924176	0.83736794311149	-1.06039007017544
O	-2.27557926569748	1.32305411469813	-1.65646601378394
C	0.00180708088515	1.67195782004294	1.05733338944803
O	0.00894490335087	2.63322992065444	1.65128955473324
C	0.00180739298113	-1.67195794050553	-1.05733351299141
O	0.00894548329119	-2.63323004054768	-1.65128967611168

$[Cr(CO)_6]^{\bullet+}$ D_{3d} TPSSh (D3BJ)

Cr	0.0000000	0.0000000	0.0000000
C	1.4445495	0.8340111	-1.0579726
O	2.2782278	1.3153354	-1.6495390
C	1.4445495	-0.8340111	1.0579726
O	2.2782278	-1.3153354	1.6495390
C	-1.4445495	-0.8340111	1.0579726
O	-2.2782278	-1.3153354	1.6495390
C	-1.4445495	0.8340111	-1.0579726
O	-2.2782278	1.3153354	-1.6495390
C	0.0000000	1.6680221	1.0579726

```
O   -0.0000000    2.6306708    1.6495390
C    0.0000000   -1.6680221   -1.0579726
O   -0.0000000   -2.6306708   -1.6495390
```

$[Cr(CO)_6]^{•+}$ C_{2h} TPSSh (D3BJ)

```
Cr   0.0000000    0.0000000    0.0000000
C    1.9727849   -0.0977432    0.0000000
O    3.1003742   -0.1697695    0.0000000
C    0.0712709   -1.3452785   -1.4445565
O    0.1233153   -2.1061052   -2.2782681
C   -1.9727849    0.0977432    0.0000000
O   -3.1003742    0.1697695    0.0000000
C   -0.0712709    1.3452785    1.4445565
O   -0.1233153    2.1061052    2.2782681
C    0.0712709   -1.3452785    1.4445565
O    0.1233153   -2.1061052    2.2782681
C   -0.0712709    1.3452785   -1.4445565
O   -0.1233153    2.1061052   -2.2782681
```

$[Cr(CO)_6]^{•+}$ D_{2h} TPSSh (D3BJ)

```
Cr   0.0000000    0.0000000    0.0000000
C    1.3278985    0.0000000    1.4486680
C   -1.3278985    0.0000000    1.4486680
C   -1.3278985    0.0000000   -1.4486680
C    0.0000000    1.9959683    0.0000000
C    1.3278985    0.0000000   -1.4486680
C    0.0000000   -1.9959683    0.0000000
O    0.0000000    3.1237888    0.0000000
O   -2.0800590    0.0000000   -2.2931913
O   -2.0800590    0.0000000    2.2931913
O    0.0000000   -3.1237888    0.0000000
O    2.0800590    0.0000000    2.2931913
O    2.0800590    0.0000000   -2.2931913
```

$[Cr(CO)_6]^{•+}$ D_{2h}* TPSSh (D3BJ)

```
Cr   0.0000000    0.0000000    0.0000000
C   -1.3323658    0.0000000    1.4521414
C    1.3323658    0.0000000   -1.4521414
C    1.3323658    0.0000000    1.4521414
C    0.0000000   -1.9707600    0.0000000
C   -1.3323658    0.0000000   -1.4521414
C    0.0000000    1.9707600    0.0000000
O    0.0000000    3.1011400    0.0000000
O    2.0847636    0.0000000    2.2957135
O   -2.0847636    0.0000000    2.2957135
O    0.0000000   -3.1011400    0.0000000
O    2.0847636    0.0000000   -2.2957135
O   -2.0847636    0.0000000   -2.2957135
```

$[Cr(CO)_6]^{•+}$ D_{4h} TPSSh (D3BJ)

```
Cr   0.0000000    0.0000000    0.0000000
```

C	0.0000000	0.0000000	-1.9406398
O	0.0000000	0.0000000	-3.0740837
C	-1.4093344	1.4093344	0.0000000
O	-2.2070384	2.2070384	0.0000000
C	0.0000000	0.0000000	1.9406398
O	0.0000000	0.0000000	3.0740837
C	1.4093344	-1.4093344	0.0000000
O	2.2070384	-2.2070384	0.0000000
C	-1.4093344	-1.4093344	0.0000000
O	-2.2070384	-2.2070384	0.0000000
C	1.4093344	1.4093344	0.0000000
O	2.2070384	2.2070384	0.0000000

$[Cr(CO)_6]^{\bullet+}$ O_h TPSSh (D3BJ)

Cr	-0.0000000	-0.0000000	-0.0000000
C	-0.0000000	1.9707625	-0.0000000
C	0.0000000	-1.9707625	0.0000000
C	0.0000000	-0.0000000	1.9707625
C	-1.9707625	-0.0000000	-0.0000000
C	-0.0000000	-0.0000000	-1.9707625
C	1.9707625	0.0000000	0.0000000
O	3.1011426	0.0000000	-0.0000000
O	-0.0000000	0.0000000	3.1011426
O	-0.0000000	3.1011426	0.0000000
O	-3.1011426	-0.0000000	0.0000000
O	0.0000000	-3.1011426	0.0000000
O	0.0000000	-0.0000000	-3.101142

10. IR and Raman Spectra

Calculated $[Cr(CO)_6]^{\bullet+}$ (Turbomole)

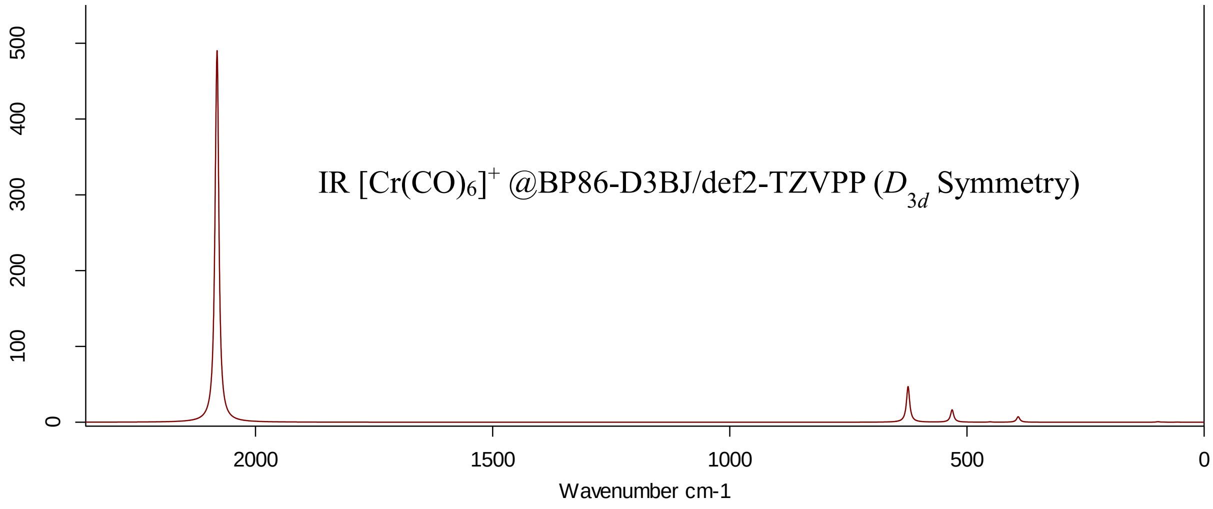

Supplementary Figure 38. Calculated IR spectrum of $[Cr(CO)_6]^+$.

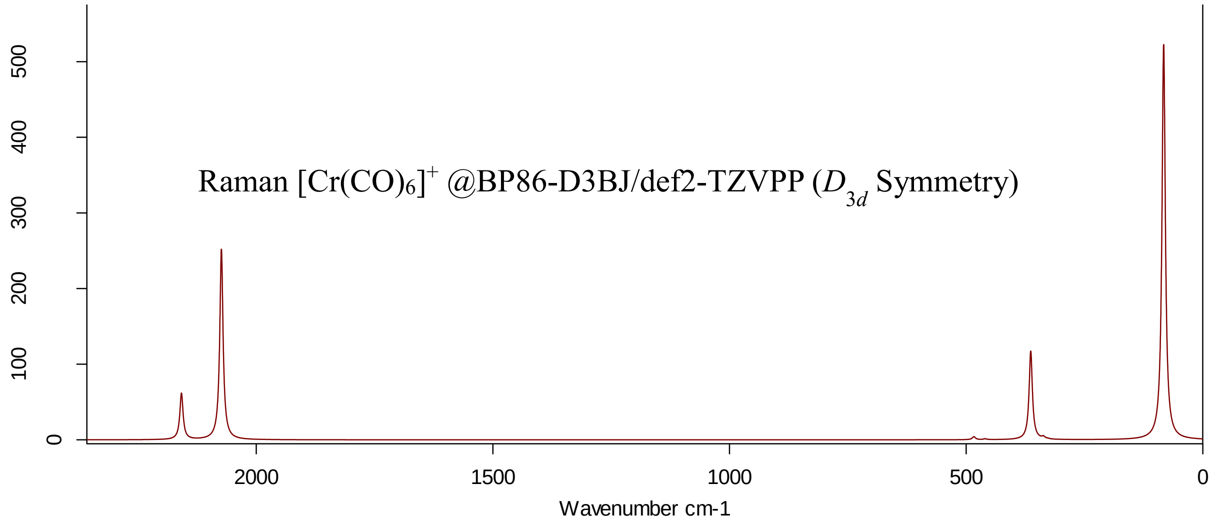

Supplementary Figure 39. Calculated Raman spectrum of $[Cr(CO)_6]^+$.

Calculated [Cr(CO)$_5$(NO)]$^+$ (Turbomole)

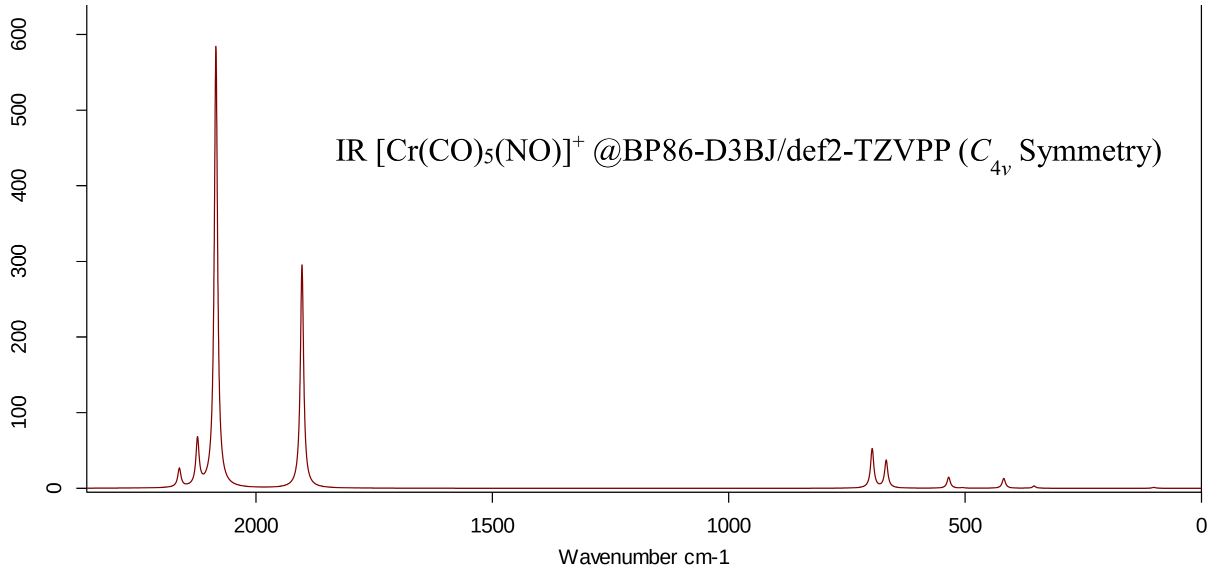

Supplementary Figure 40. Calculated IR spectrum of [Cr(CO)$_5$(NO)]$^+$.

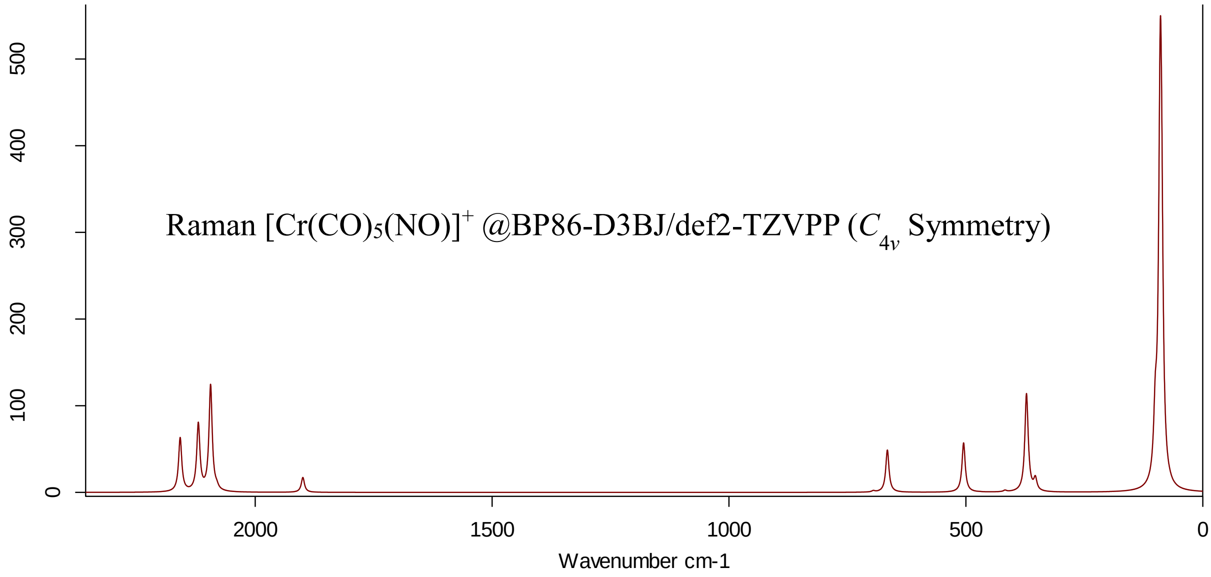

Supplementary Figure 41. Calculated Raman spectrum of [Cr(CO)$_5$(NO)]$^+$.

[$Cr(CO)_6$][$Al(OR^F)_4$] (1)

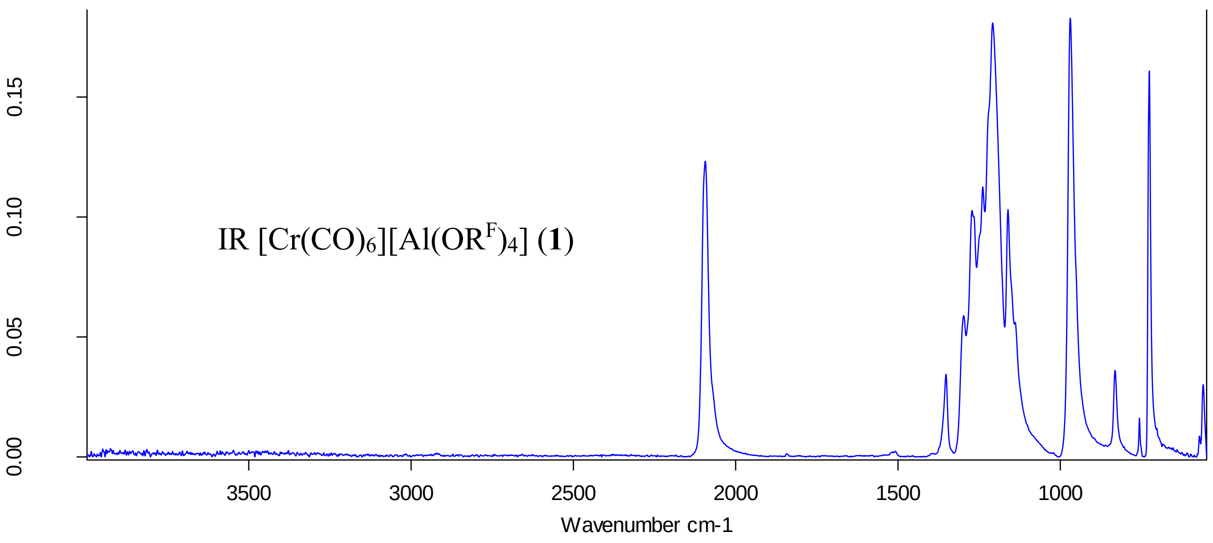

Supplementary Figure 42. IR spectrum of **1**.

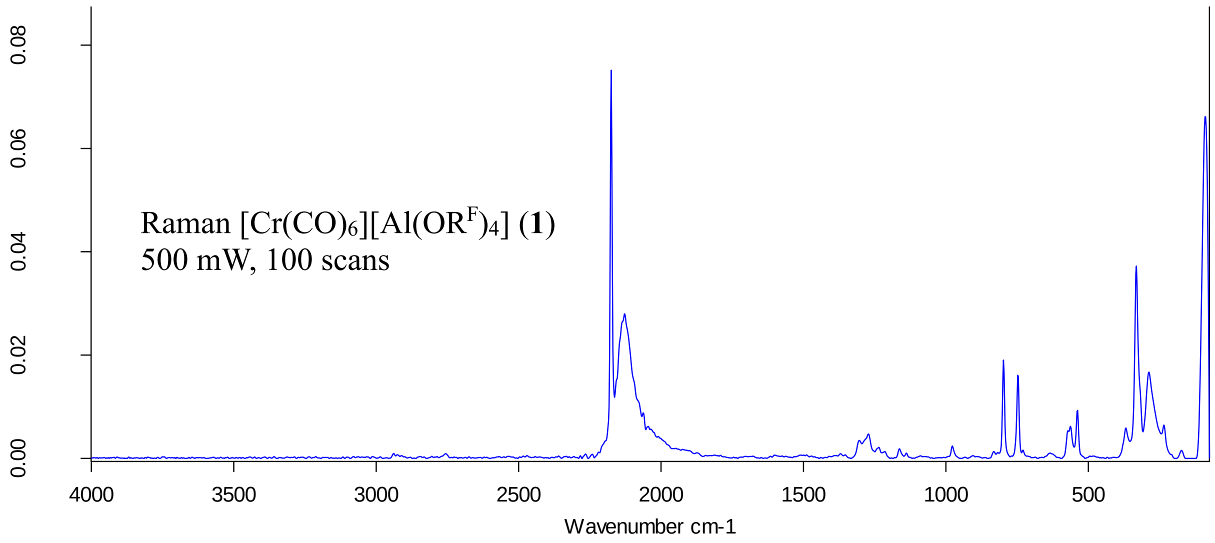

Supplementary Figure 43. Raman spectrum of **1**.

[Cr(CO)$_6$][F-{Al(OR^F)$_3$}$_2$] (2)

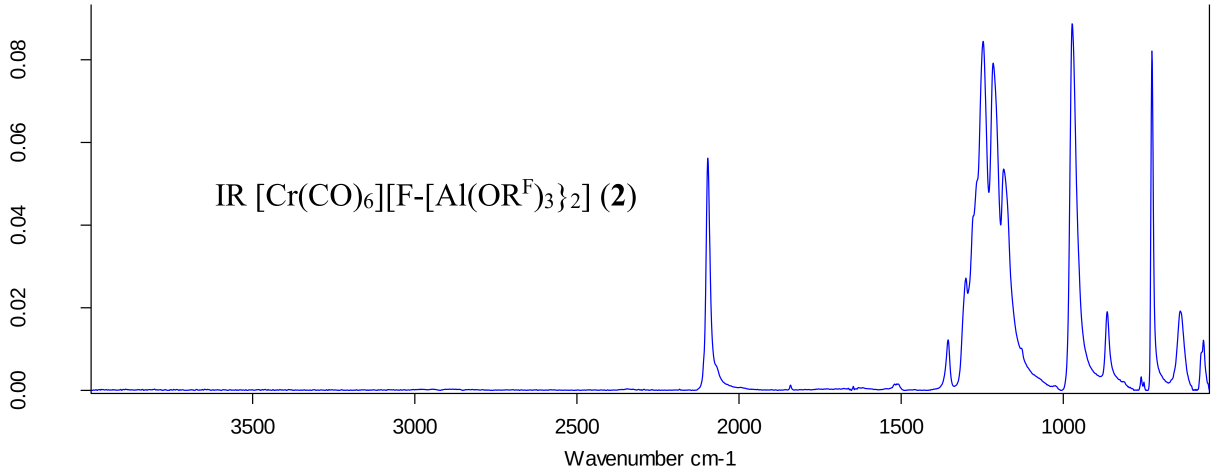

Supplementary Figure 44. IR spectrum of **2**.

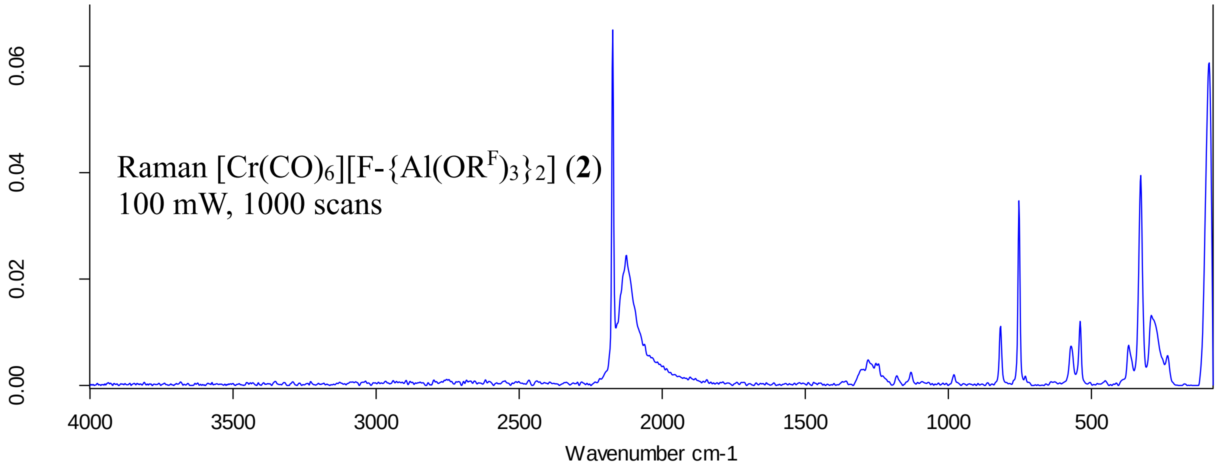

Supplementary Figure 45. Raman spectrum of **2**.

[Cr(CO)$_5$(NO)][Al(OR^F)$_4$] (3)

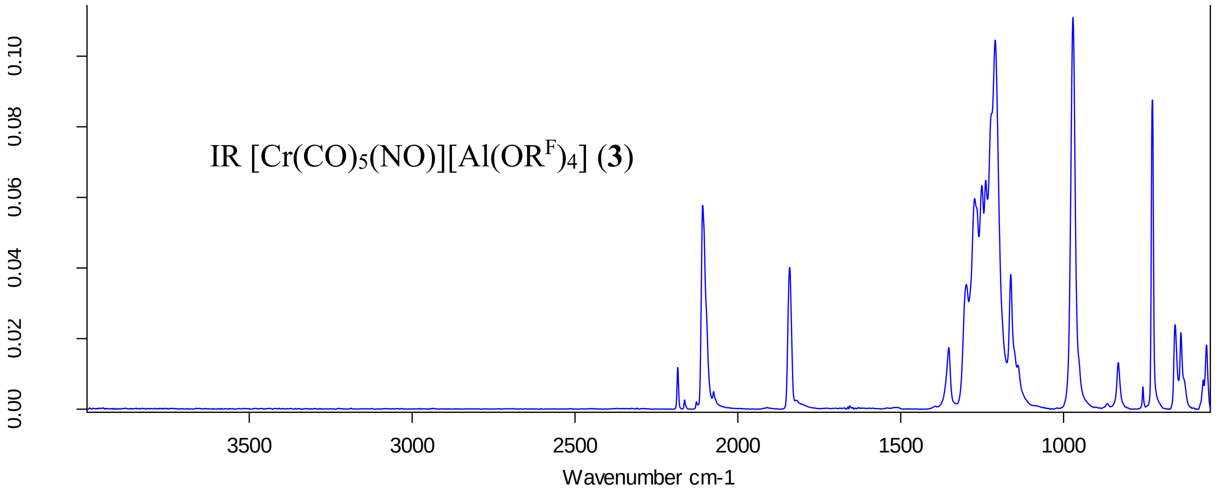

Supplementary Figure 46. IR spectrum of **3**.

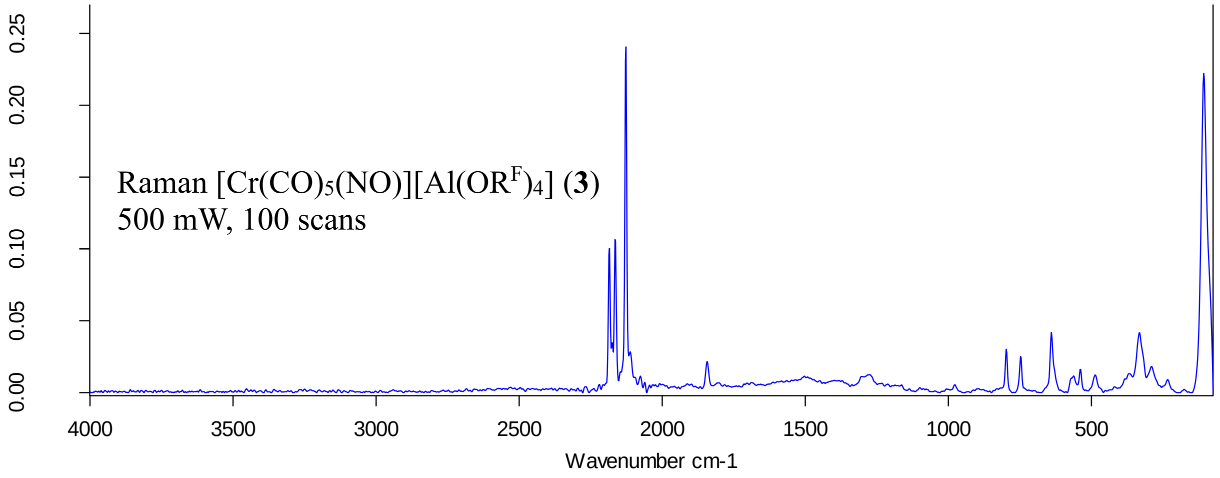

Supplementary Figure 47. Raman spectrum of **3**.

[Cr(CO)$_5$(NO)][F-{Al(OR^F)$_3$}$_2$] (4)

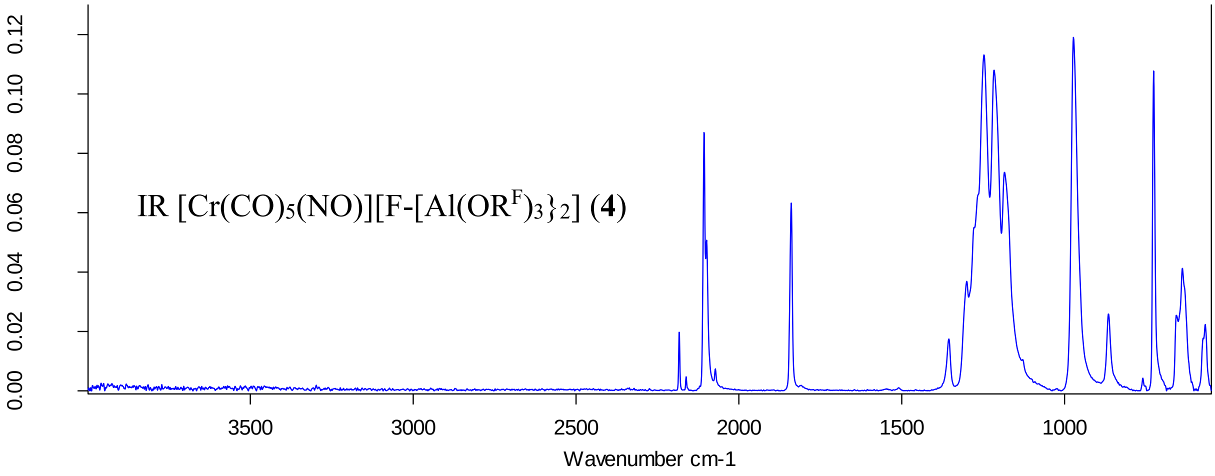

Supplementary Figure 48. IR spectrum of **4**.

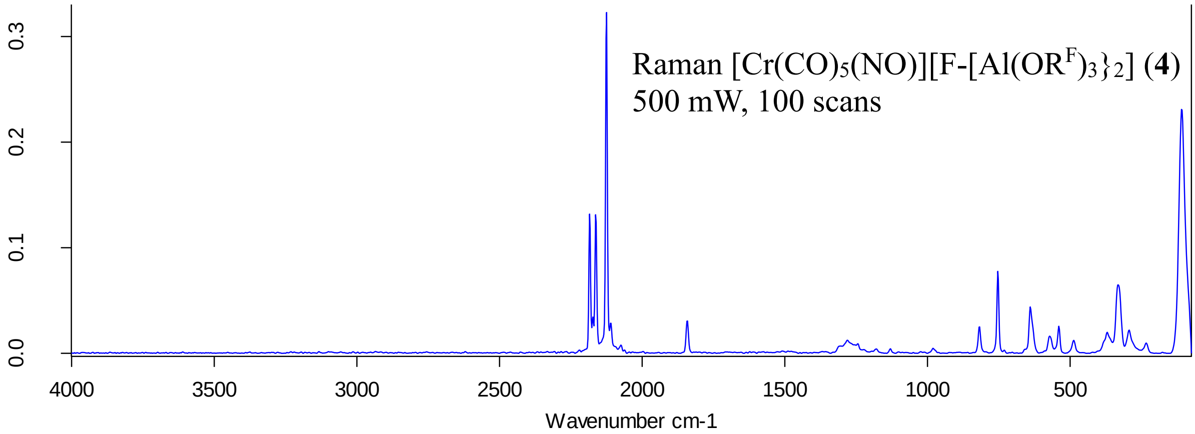

Supplementary Figure 49. Raman spectrum of **4**.

11. UV/Vis Spectra

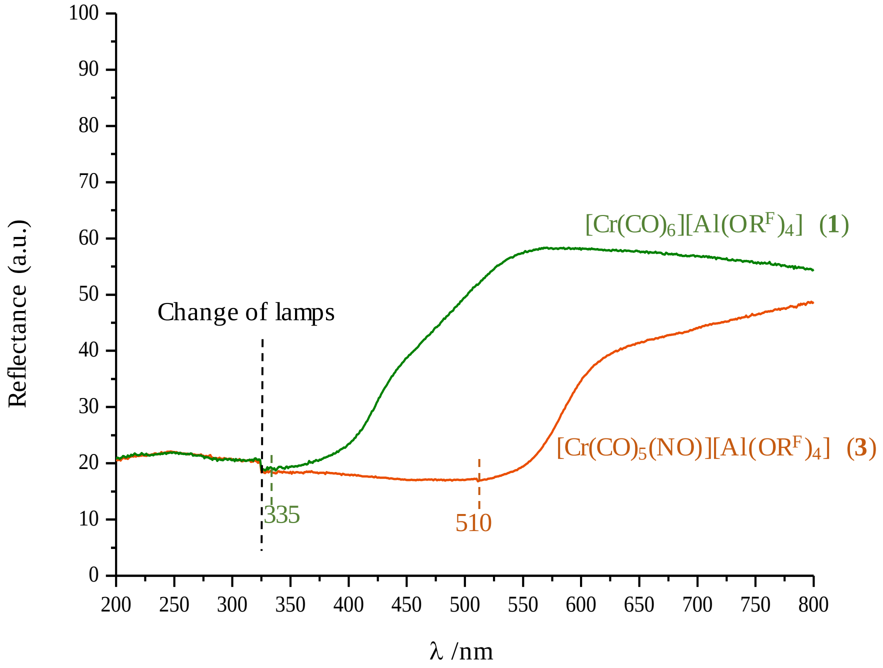

Supplementary Figure 50. Diffuse reflectance spectra of complexes **1** (green line) and **3** (orange line) and their maximum absorption at 335 nm and 510 nm respectively. The dashed line at 325 nm indicates the change of lamps of the spectrometer.

12. Elemental Analyses

Due to the high fluorine content (about 57% and 61%), reliable elemental analyses are problematic[54,55] (see for example: Marcó, A.; Compañó, R.; Rubio, R.; Casals, I. *Microchim. Acta* **2003**, *142*, 13−19 or the *Organometallics* editorial *Organometallics* **2016**, *35* (19), 3255–3256) and we refrained from using them in the first place.

In addition, we have done quite a few tests with our materials and using the equipment available to us in the chemistry department in Freiburg. Unfortunately, also with the new set up that was acquired only 3 years ago and supposedly able to treat CF_3 groups, the test elemental analyses of electrochemically and spectroscopically extremely pure air- and water-stable $[NBu_4][Al(OR^F)_4]$ gave combustion analysis values that were off by erratically 2-3 % for samples from the same batch. However, the facility at KIT Karlsruhe was able to deal with compounds with high fluorine contents. Exemplarily, we tested a sample of $[Cr(CO)_6][(Al(OR^F)_4]$ (calcd.: C 22.26; Al 2.27; Cr 4.38; F 57.61; O 13.48) there. The results are shown in the table below (Supplementary Table 6).

Supplementary Table 6. Elemental analysis of **1**.

Sample	N	C	H	S
1.178 mg	0.17	23.30	0.247	0.109
2.367 mg	0.08	22.71	0.130	0.049
Avg.	*0.13*	*23.00*	*0.189*	*0.079*
**Expected** $[Cr(CO)_6](Al(OR^F)_4]$	0.00	22.26	0.000	0.000
**Expected** $[Cr(CO)_5(NO)](Al(OR^F)_4]$	1.18	21.21	0.000	0.000

The discrepancy between theoretical and experimental carbon content is small enough to be acceptable for an inorganic organometallic compound like this and is considered publishable, especially with the reasons given above. However, elemental analyses does not answer the question of the purity of the bulk materials any better than the sum of vibrational and NMR spectroscopy as well as pXRD which we thoroughly deployed here. This is the reason, why we trust the combination of these methods more than a doubtful combustion analysis with large deviations and tolerance thereof.

13. Powder XRD Data and Rietveld Refinement

In order to evaluate the phase purity of the bulk materials **1**–**4**, powder XRD measurements were conducted at 100K. In the following section the experimental diffractogram and the from the single-crystal data calculated/simulated powder XRD are compared. They show perfect agreement of the reflexes and therefore the absence of crystalline impurities. Furthermore, an overlay of the background and non-background corrected diffractograms indicate the absence of large amounts of amorphous impurities (see Supplementary Figure 51 to Supplementary Figure 62 for all powder-XRD plots).

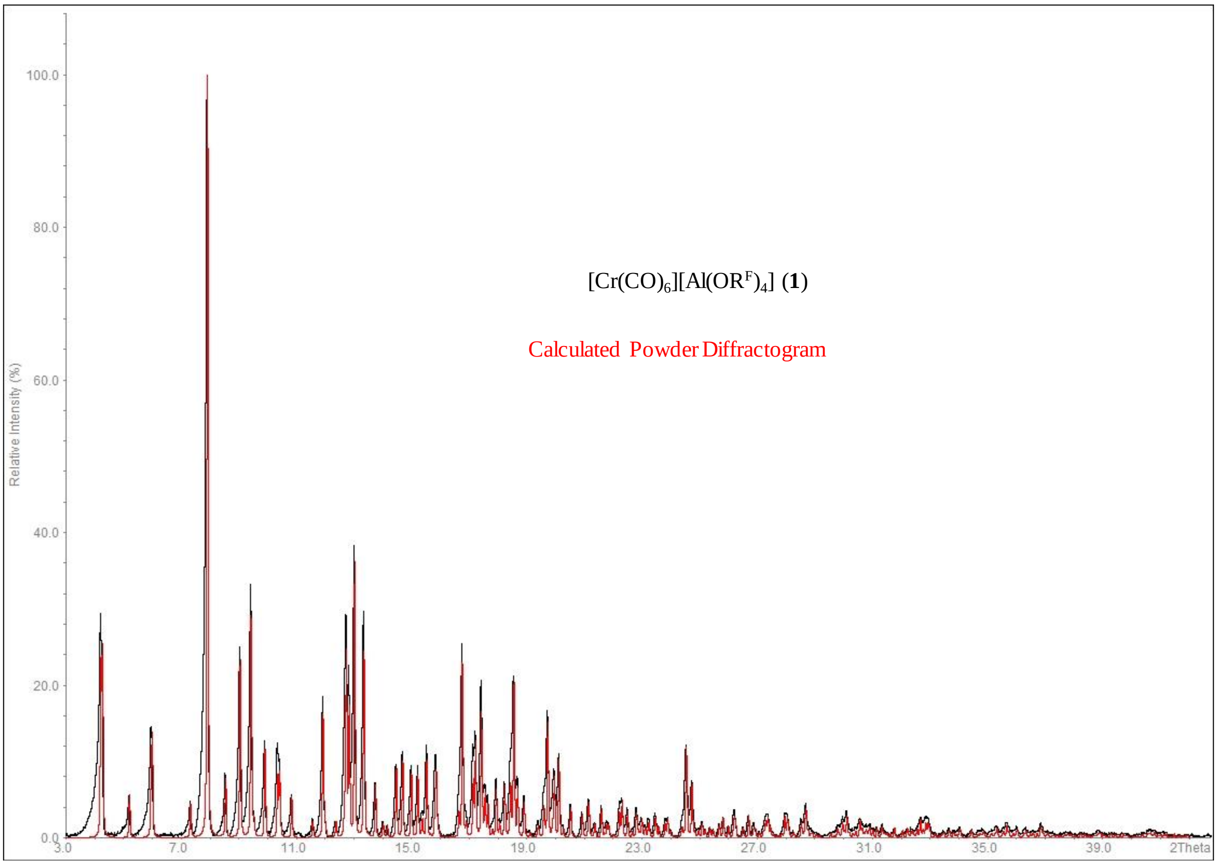

Supplementary Figure 51. Experimental (black) and calculated (red) powder diffractogram for **1**.

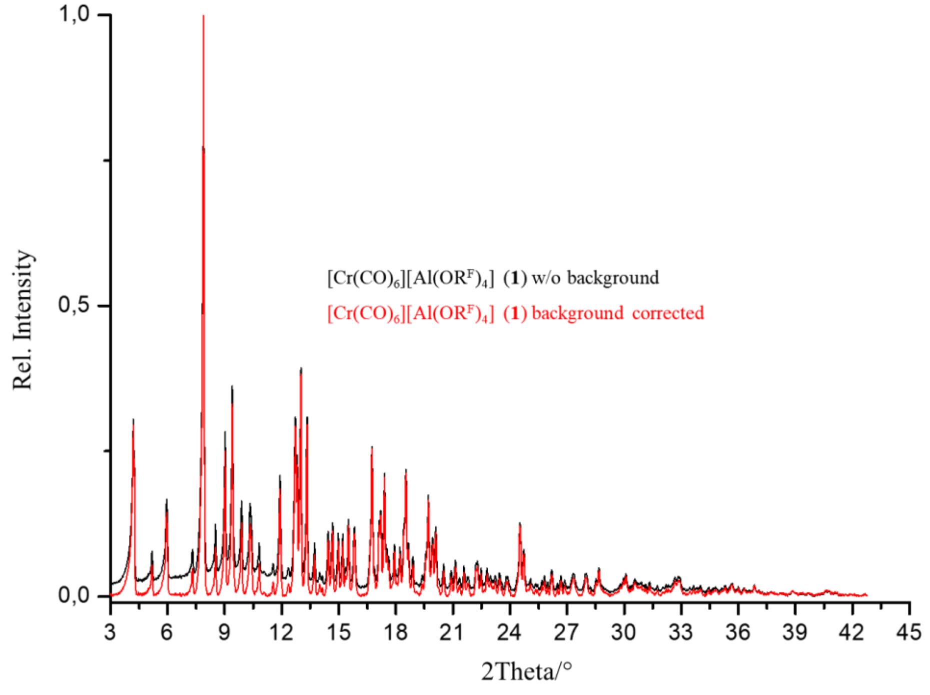

Supplementary Figure 52. Experimental powder diffractogram of **1** with (red) and without (black) background correction.

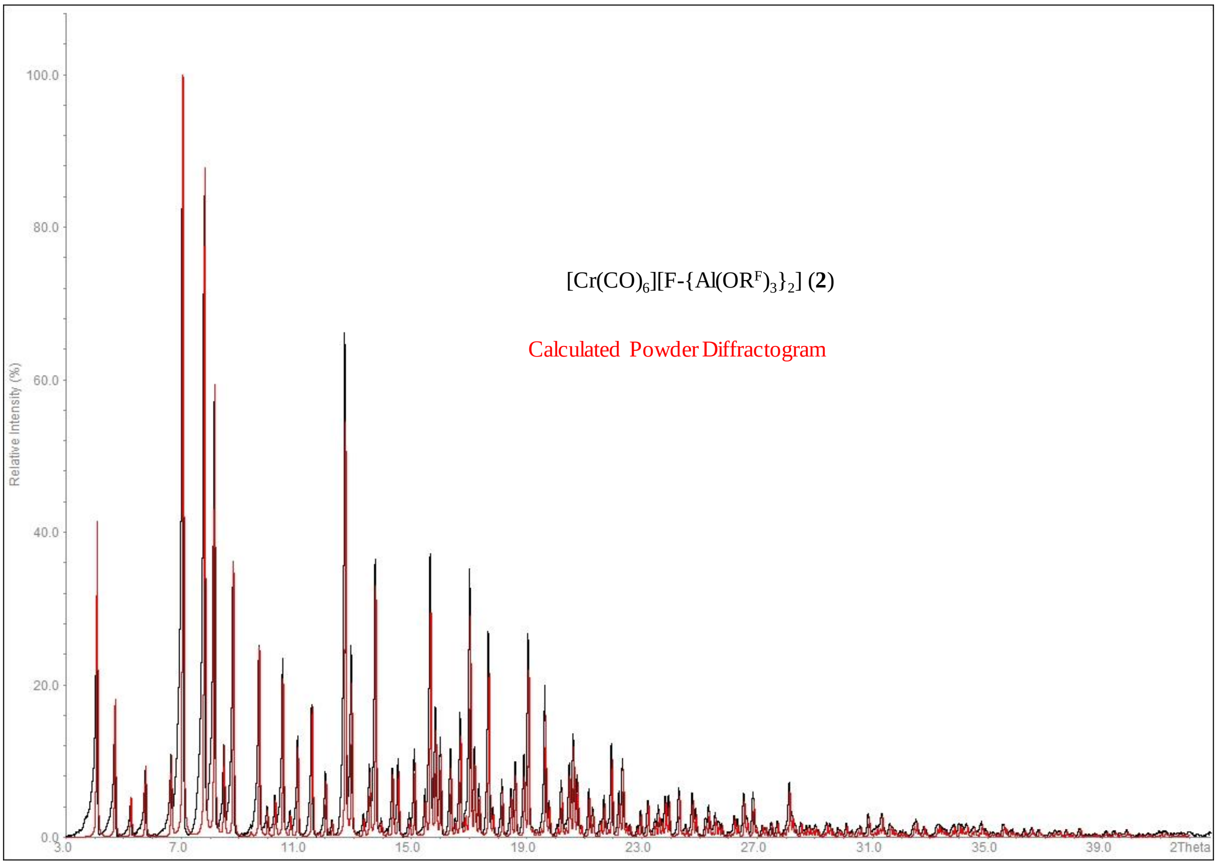

Supplementary Figure 53. Experimental (black) and calculated (red) powder diffractogram of **2**.

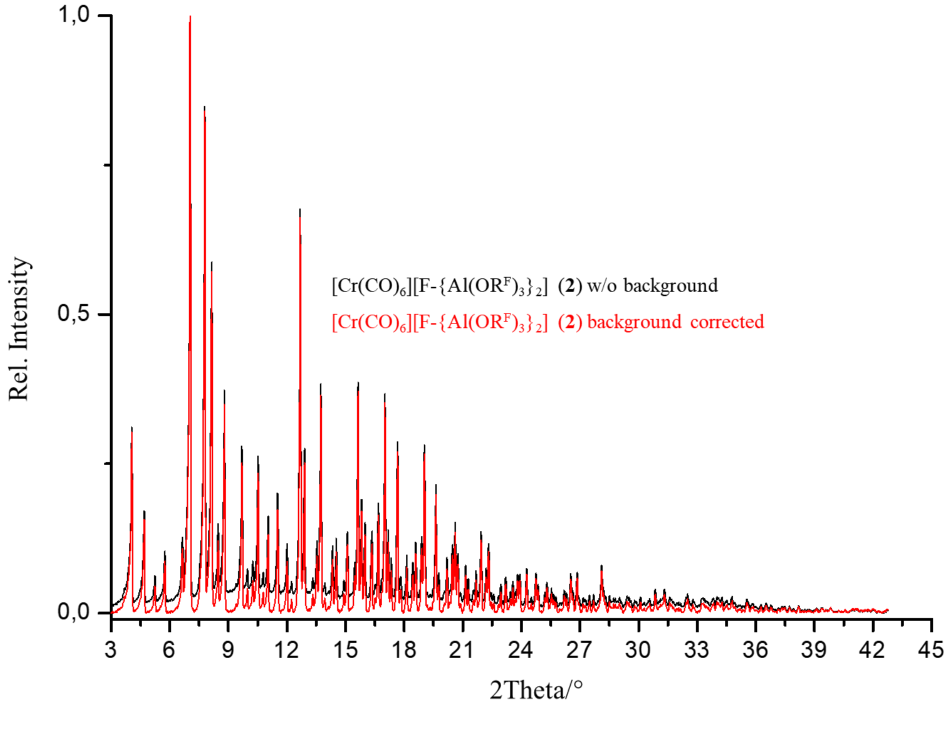

Supplementary Figure 54. Experimental powder diffractogram of **2** with (red) and without (black) background correction

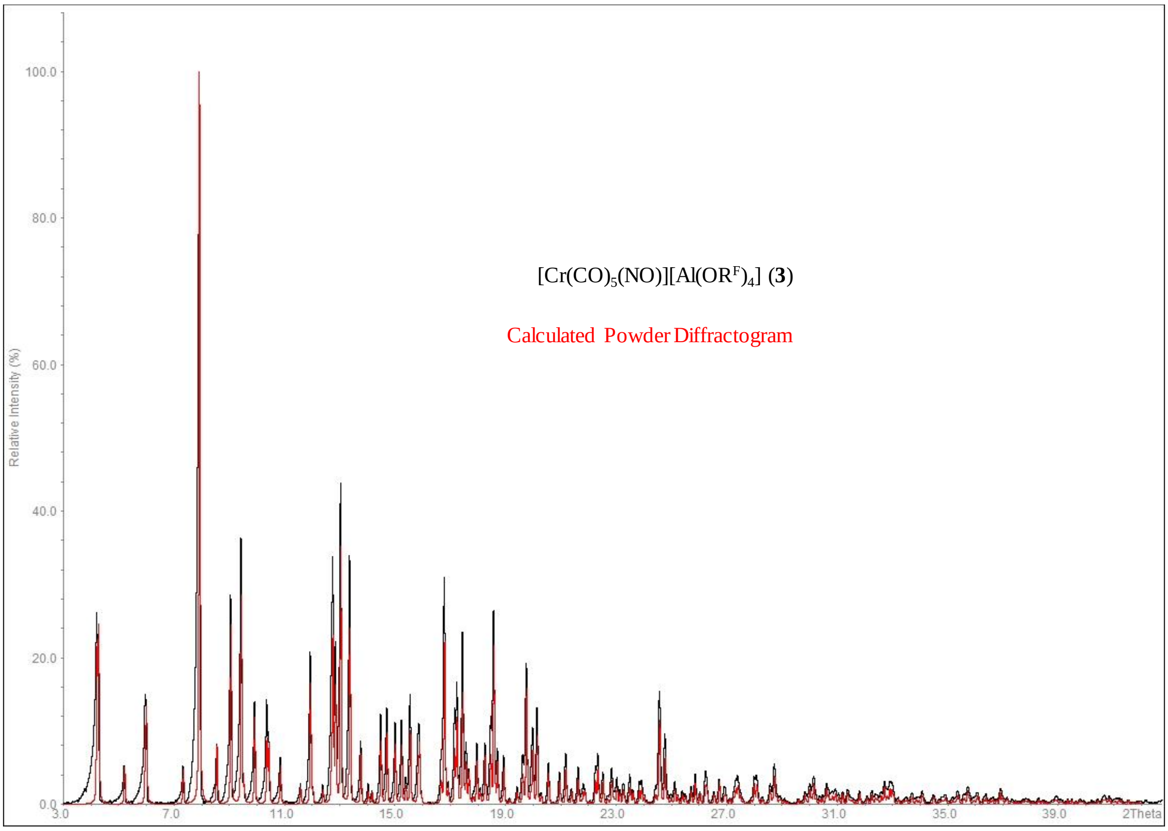

Supplementary Figure 55. Experimental (black) and calculated (red) powder diffractogram of **3**.

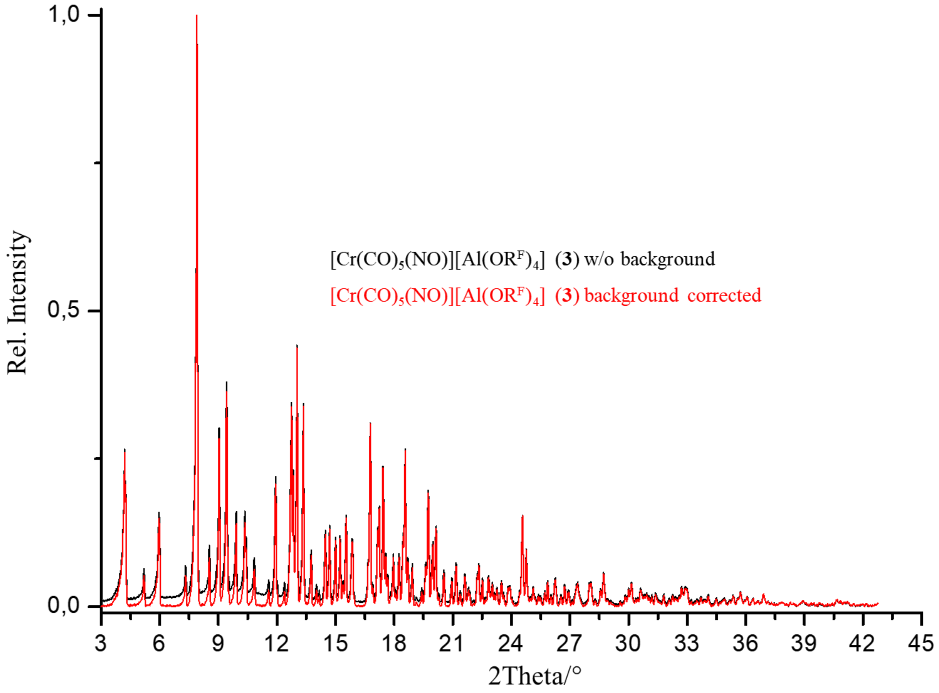

Supplementary Figure 56. Experimental powder diffractogram of **3** with (red) and without (black) background correction.

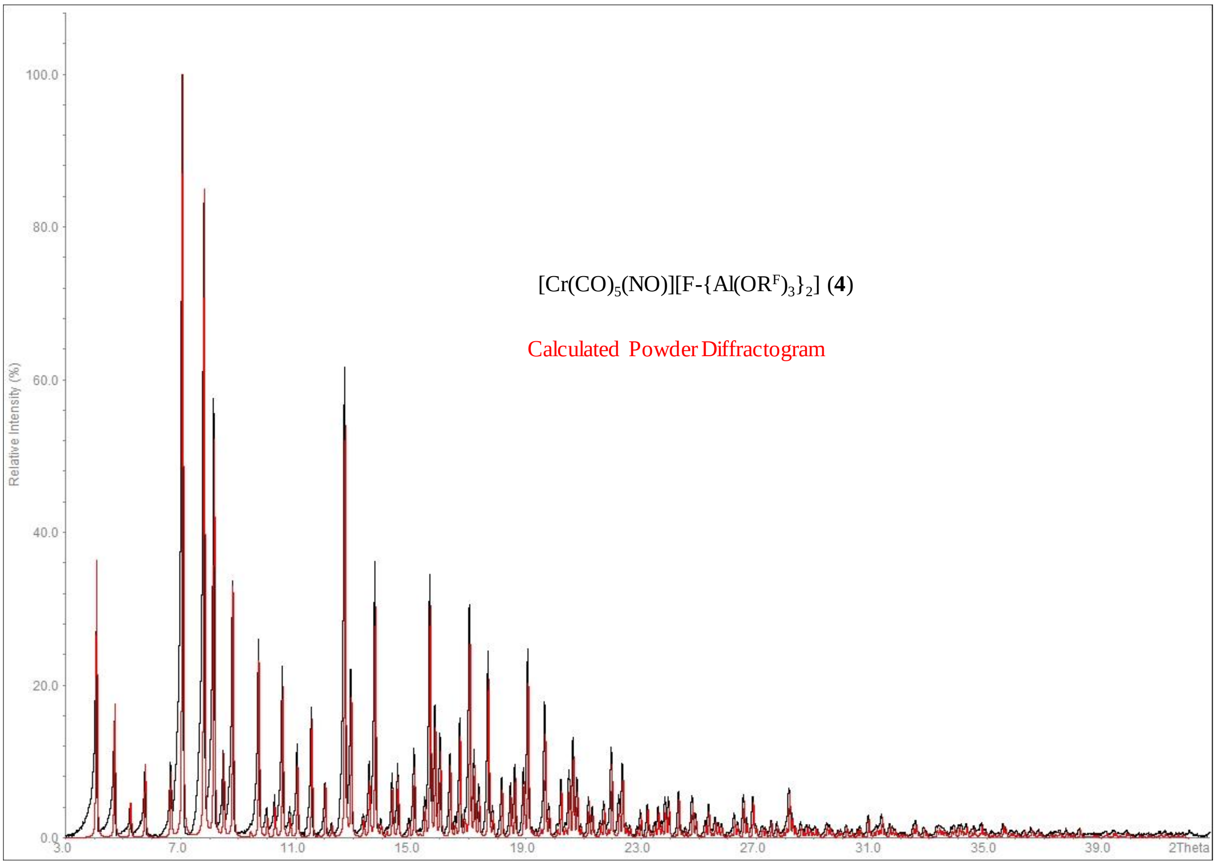

Supplementary Figure 57. Experimental (black) and calculated (red) powder diffractogram of **4.**

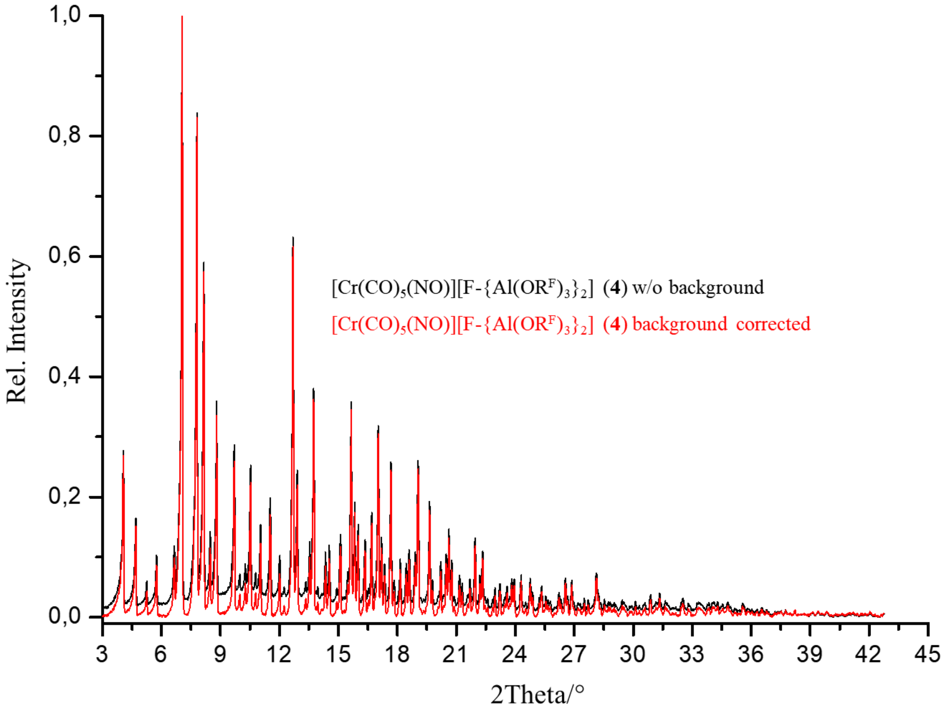

Supplementary Figure 58. Experimental powder diffractogram of **4** with (red) and without (black) background correction.

In order to emphasize the small difference in unit cell dimensions of (by single-crystal XRD) undistinguishable complexes **1** and **3** (**2** and **4** respectively), an overlay of the two powder diffractograms with a magnification of the 3-10° and 19-25° 2Theta range to emphasize the slight difference between the two diffractrograms.

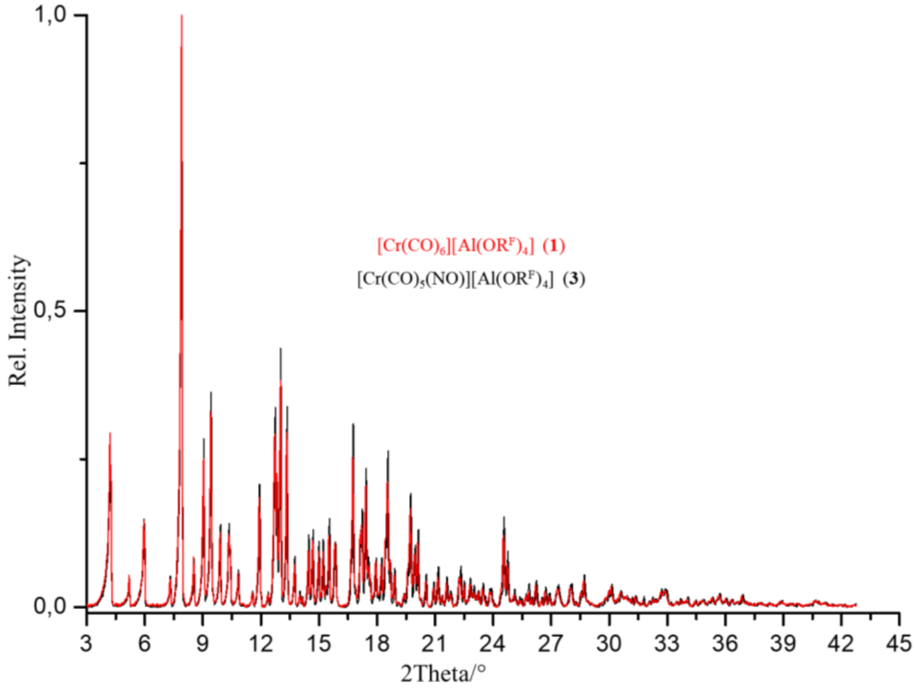

Supplementary Figure 59. Comparison of experimental powder diffractograms of **1** (red) and **3** (black).

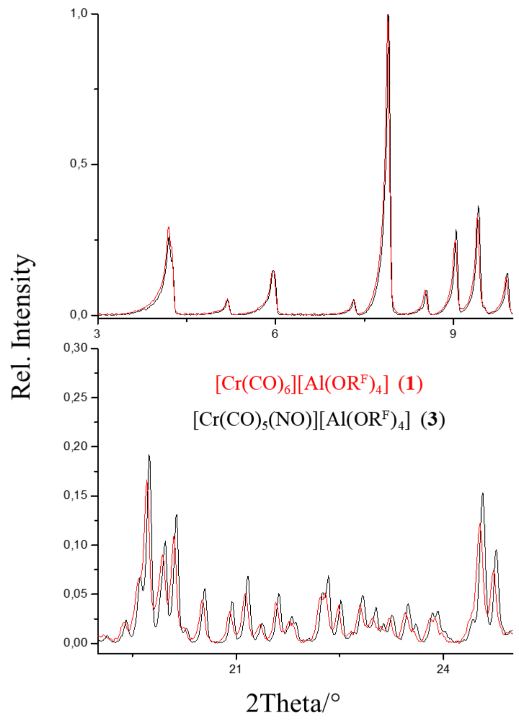

Supplementary Figure 60. Magnification of the 3-10° and 19-25° 2Theta range of the comparison of experimental powder diffractograms of **1** (red) and **3** (black).

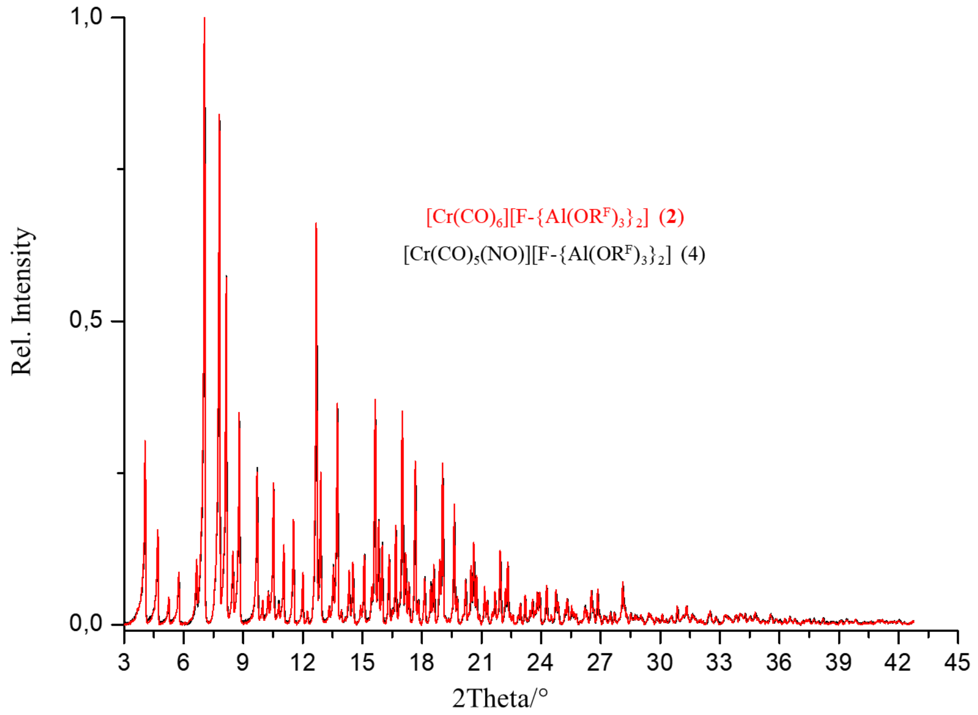

Supplementary Figure 61. Comparison of experimental powder diffractograms of **2** (red) and **4** (black).

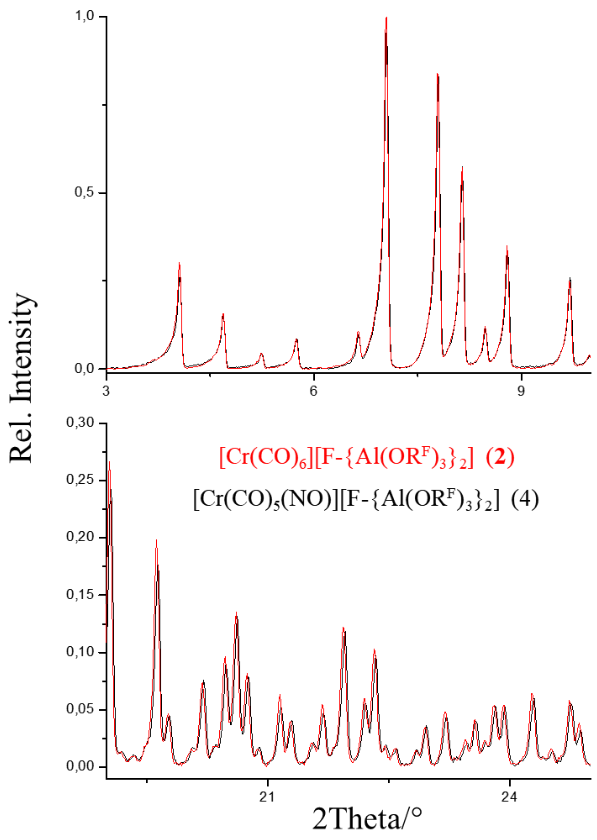

Supplementary Figure 62. Magnification of the 3-10° and 19-25° 2Theta range of the comparison of experimental powder diffractograms of **2** (red) and **4** (black).

Rietveld Refinement

Since Cr–N distances in the mixed complexes are expected to be shorter (see QM calculations), also their unit cell is slightly, but noticeably smaller than that of the all-carbonyl compounds. To show this also for the microcrystalline bulk, we performed Rietveld-refinements of the powder data recorded at the same temperature like the single crystal data (100 K). Since the pXRD record higher angle data than the scXRD data, the resolution is better and the standard deviations are further reduced. Thus, also the bulk of the material show the smaller lattice parameters for the mixed NO-carbonyl complexes (see Supplementary Table 7 and Supplementary Figure 63 to Supplementary Figure 66).

All reflections measured by X-ray powder diffraction have been indexed and their observed intensities are in very good agreement with the calculated diffraction patterns based on single crystal data. The Rietveld refinements of the structure models (see section 14) have been performed with the program GSAS[56,57] and confirm the single crystal data. The atomic parameters remain almost unchanged and therefore the detailed structural parameters are not reproduced herein.

Cell volumes of $[Al(OR^F)_4]^-$ salts **1** and **3**: The cell volumes are V = 1776.42(4) (**1**, all carbonyl) and V = 1767.39(3) (**3**, mixed-NO-carbonyl). With a standard deviation of only 0.04 and 0.03 Å^3, the volume difference of 9.03 Å^3 between both salts is more than statistically relevant.

Cell volumes of $[F\text{-}\{Al(OR^F)_3\}_2]$ salts **2** and **4**: here, the volumes are V = 5156.13(7) (**3**) and 5145.08(7) (**4**). This difference of 11.05 Å^3 is also statistically relevant and supports the claim that the presence of the shorter Cr–N bond leads to slightly but significantly smaller unit cells even for the bulk samples.

Supplementary Table 7. Results of the Rietveld refinement.

**Compound**	$[Cr(CO)_6]$ $[Al(OR^F)_4]$	$[Cr(CO)_5(NO)]$ $[Al(OR^F)_4]$	$[Cr(CO)_6]$ $[F\text{-}Al(OR^F)_3\}_2]$	$[Cr(CO)_5(NO)]$ $[F\text{-}Al(OR^F)_3\}_2]$
**Temp.**	100 K	100 K	100 K	100 K
**Space Group**	*P*4/*n*	*P*4/*n*	$Pa\bar{3}$	$Pa\bar{3}$
**Cell**	a = 13.65605(15)	a = 13.63318(11)	a = 17.27593(8)	a = 17.26357(8)
	c = 9.52568(17)	c = 9.50905(12)		
	V = 1776.42(4)	V = 1767.39(3)	V = 5156.13(7)	V = 5145.08(7)
$\boldsymbol{R_F2}$	0.0403	0.0428	0.0456	0.0457
$\boldsymbol{wR_p}$	0.0363	0.0408	0.0309	0.0341
$\boldsymbol{R_p}$	0.0285	0.0314	0.0243	0.0266
χ^2	2.690	4.038	2.564	2.890

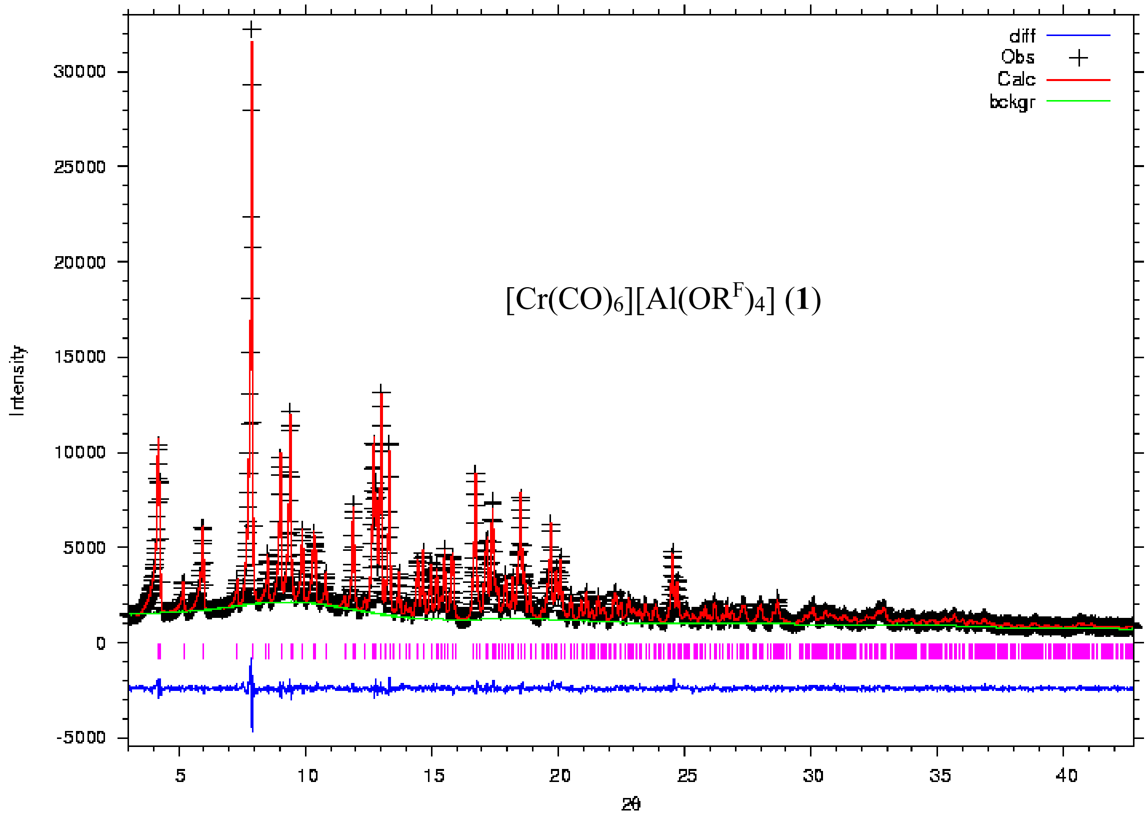

Supplementary Figure 63. Plot of the Rietveld refinement of **1**.

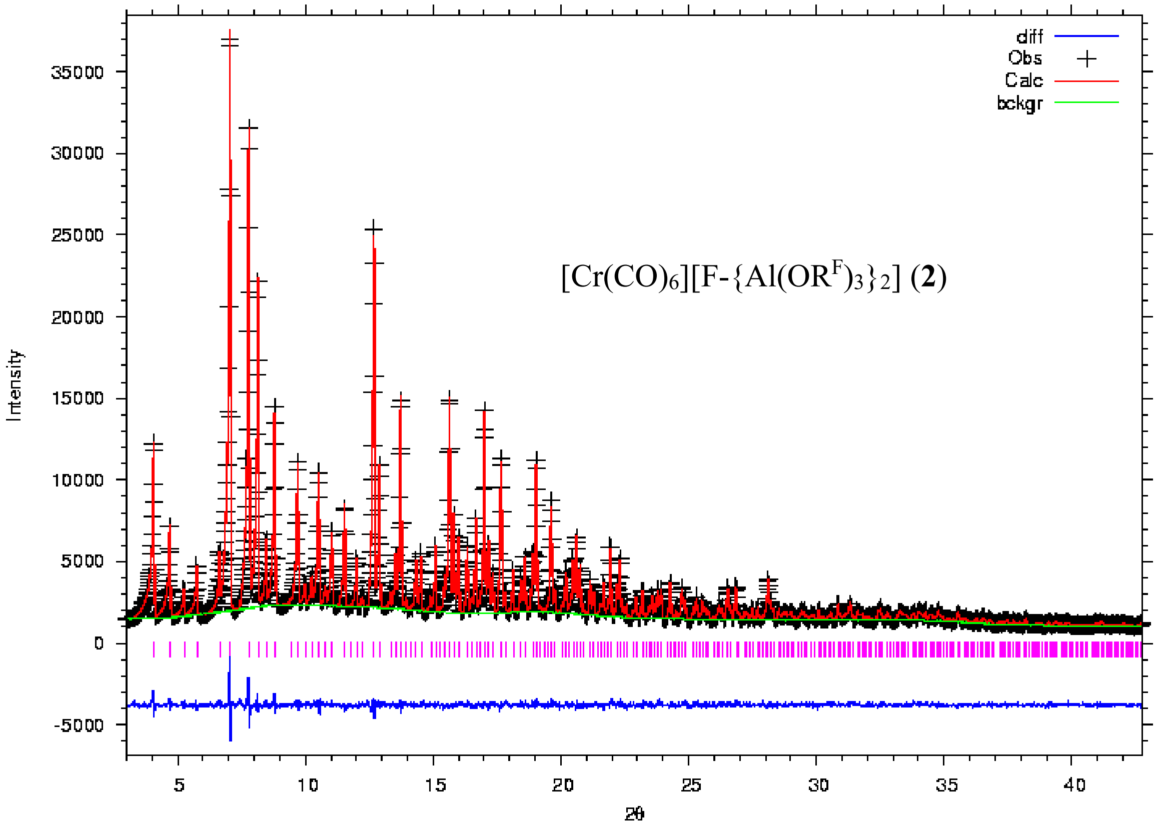

Supplementary Figure 64. Plot of the Rietveld refinement of **2**.

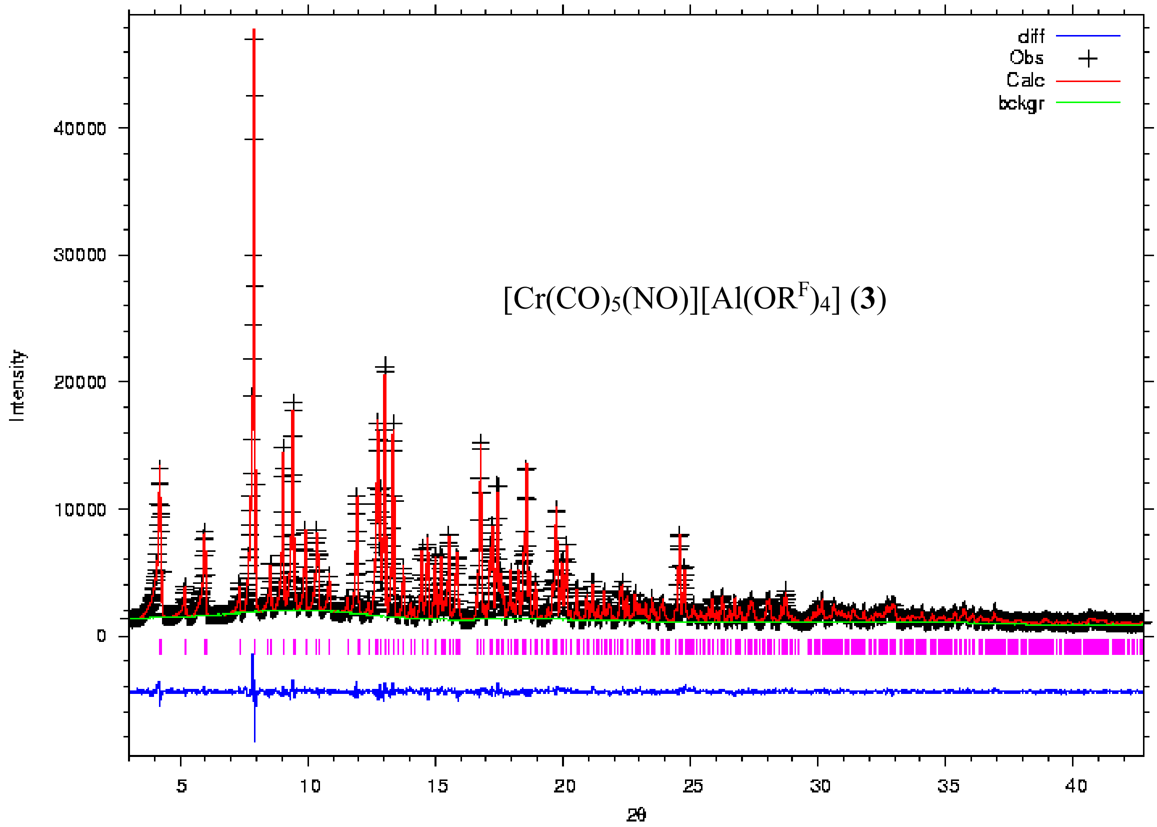

Supplementary Figure 65. Plot of the Rietveld refinement of **3**.

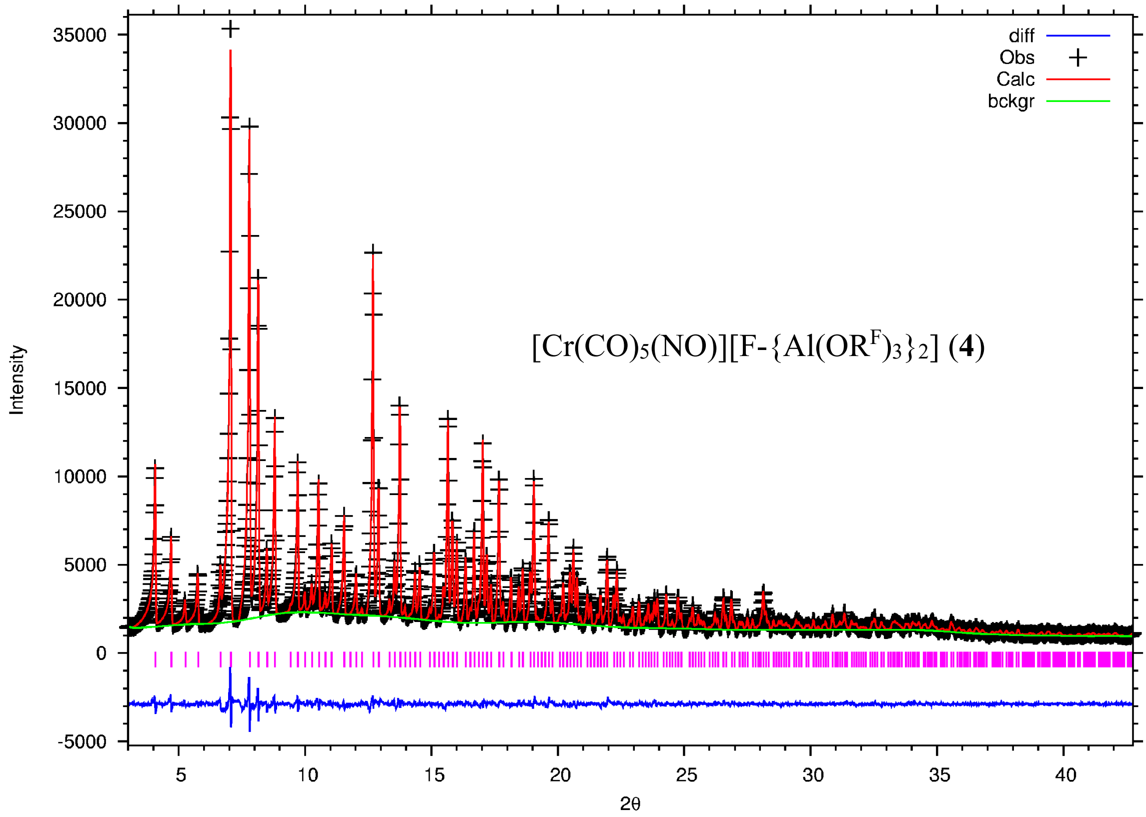

Supplementary Figure 66. Plot of the Rietveld refinement of **4**.

14. Single-Crystal XRD Data

Supplementary Figure 67 shows the crystal structure of **1**, Supplementary Data 2 gives full information on the crystallographic data.

<u>Crystal structure of 1, the symmetry-generated atom sites are greyed out.</u>

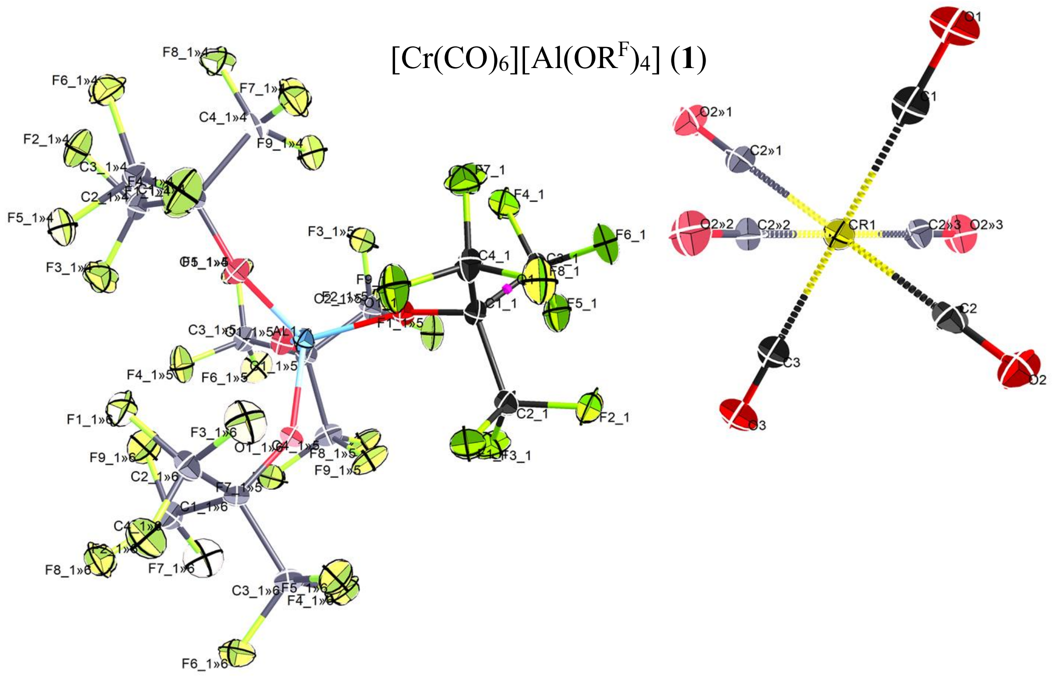

Supplementary Figure 67. Crystal structure of **1**.

Crystal data and structure refinement for p4n_a_final.

Identification code	p4n_a_final
Empirical formula	$C_{22}AlCrF_{36}O_{10}$
Formula weight	1187.20
Temperature/K	100(2)
Crystal system	tetragonal
Space group	P4/n
a/Å	13.6531(3)
b/Å	13.6531(3)
c/Å	9.5200(2)
α/°	90
β/°	90
γ/°	90
Volume/Å^3	1774.60(9)
Z	2
ρ_{calc}g/cm^3	2.222
μ/mm^{-1}	0.591
F(000)	1146.0
Crystal size/mm^3	0.1 × 0.08 × 0.08
Radiation	MoKα (λ = 0.71073)
2Θ range for data collection/°	4.218 to 59.068
Index ranges	-13 ≤ h ≤ 18, -18 ≤ k ≤ 11, -13 ≤ l ≤ 7
Reflections collected	11628
Independent reflections	2492 [R_{int} = 0.0143, R_{sigma} = 0.0129]
Data/restraints/parameters	2492/189/162
Goodness-of-fit on F^2	1.068
Final R indexes [I>=2σ (I)]	R_1 = 0.0273, wR_2 = 0.0704
Final R indexes [all data]	R_1 = 0.0308, wR_2 = 0.0723
Largest diff. peak/hole / e Å^{-3}	0.46/-0.43

Supplementary Data 2. Information on the crystallographic data of **1**.

Fractional Atomic Coordinates (×10^4) and Equivalent Isotropic Displacement Parameters (Å^2×10^3) for p4n_a_final. U_{eq} is defined as 1/3 of of the trace of the orthogonalised U_{IJ} tensor.

Atom	*x*	*y*	*z*	U(eq)
Al1	7500	2500	10000	14.20(14)
O1_1	6669.4(6)	3145.0(6)	8984.6(9)	18.52(18)
C1_1	5826.0(9)	3116.3(9)	8245.2(12)	16.5(2)
C2_1	4945.0(9)	2839.4(10)	9200.6(14)	21.7(2)
F1_1	4917.6(6)	1878.8(6)	9438.0(9)	28.68(19)
F2_1	4086.5(6)	3094.0(7)	8629.8(9)	30.9(2)
F3_1	5009.8(6)	3280.4(7)	10445.5(9)	31.0(2)
C3_1	5648.0(9)	4156.2(9)	7620.5(14)	21.2(2)
F4_1	6477.4(6)	4529.2(6)	7103.9(10)	30.2(2)
F5_1	5325.8(6)	4774.3(6)	8596.5(9)	28.38(19)
F6_1	4988.3(6)	4151.4(6)	6575.8(9)	29.27(19)
C4_1	5891.6(10)	2366.2(10)	7017.8(14)	22.7(3)
F7_1	6436.6(7)	2715.4(7)	5977.0(9)	33.8(2)
F8_1	5009.0(7)	2142.4(6)	6475.2(9)	30.58(19)
F9_1	6298.0(7)	1536.7(6)	7459.5(10)	31.8(2)
Cr1	2500	2500	3706.5(4)	16.09(9)
O1	2500	2500	465(2)	36.2(5)
C1	2500	2500	1645(3)	24.1(5)
O2	435.6(7)	1535.0(8)	3698.6(11)	29.2(2)
C2	1177.5(10)	1885.8(9)	3704.7(13)	20.8(2)
C3	2500	2500	5774(3)	22.2(5)
O3	2500	2500	6955(2)	31.9(5)

Anisotropic Displacement Parameters (Å^2×10^3) for p4n_a_final. The Anisotropic displacement factor exponent takes the form: $-2\pi^2[h^2a^{*2}U_{11}+2hka^*b^*U_{12}+\ldots]$.

Atom	U_{11}	U_{22}	U_{33}	U_{23}	U_{13}	U_{12}
Al1	12.76(19)	12.76(19)	17.1(3)	0	0	0
O1_1	14.9(4)	16.8(4)	23.9(4)	1.8(3)	-3.6(3)	0.1(3)
C1_1	14.9(5)	15.5(5)	19.1(5)	-0.2(4)	-0.5(4)	1.6(4)
C2_1	16.7(5)	24.0(6)	24.5(6)	0.1(5)	0.2(4)	-0.3(5)
F1_1	24.6(4)	24.1(4)	37.4(5)	7.4(3)	-1.9(3)	-6.9(3)
F2_1	14.3(4)	37.0(5)	41.4(5)	2.5(4)	-1.4(3)	2.8(3)
F3_1	30.3(4)	38.9(5)	23.8(4)	-5.5(3)	6.6(3)	-2.2(4)
C3_1	20.4(6)	18.4(6)	24.7(6)	1.1(5)	-3.3(5)	2.4(4)
F4_1	27.3(4)	25.4(4)	37.9(5)	12.3(3)	0.8(3)	-2.2(3)
F5_1	31.8(4)	18.9(4)	34.5(4)	-5.5(3)	-4.6(3)	8.2(3)
F6_1	30.9(4)	26.5(4)	30.3(4)	3.2(3)	-13.2(3)	5.1(3)
C4_1	23.4(6)	21.2(6)	23.6(6)	-4.0(5)	-2.9(5)	3.7(5)
F7_1	37.0(5)	39.0(5)	25.5(4)	-4.7(4)	9.1(4)	3.6(4)
F8_1	30.9(4)	28.6(4)	32.3(4)	-8.3(3)	-10.9(3)	-0.1(3)
F9_1	37.1(5)	19.3(4)	39.1(5)	-7.4(3)	-7.4(4)	9.8(3)
Cr1	15.97(12)	15.97(12)	16.34(17)	0	0	0
O1	44.5(8)	44.5(8)	19.5(9)	0	0	0
C1	23.4(8)	23.4(8)	25.7(12)	0	0	0
O2	21.9(5)	31.8(5)	33.9(5)	-2.1(4)	-0.3(4)	-4.9(4)
C2	23.5(6)	20.3(6)	18.6(5)	-1.2(4)	-0.3(4)	1.5(5)
C3	21.3(7)	21.3(7)	24.1(12)	0	0	0
O3	37.6(7)	37.6(7)	20.4(9)	0	0	0

Bond Lengths for p4n_a_final.

Atom	Atom	Length/Å	Atom	Atom	Length/Å
Al1	O1_1^1	1.7309(8)	C3_1	F6_1	1.3418(15)
Al1	O1_1^2	1.7309(8)	C4_1	F7_1	1.3277(16)
Al1	O1_1^3	1.7309(8)	C4_1	F9_1	1.3294(15)
Al1	O1_1	1.7309(8)	C4_1	F8_1	1.3461(16)
O1_1	C1_1	1.3501(14)	Cr1	C1	1.962(3)
C1_1	C2_1	1.5546(17)	Cr1	C3	1.969(3)
C1_1	C4_1	1.5565(17)	Cr1	C2	1.9909(13)
C1_1	C3_1	1.5583(17)	Cr1	C2^4	1.9909(13)
C2_1	F1_1	1.3314(15)	Cr1	C2^5	1.9909(13)
C2_1	F3_1	1.3322(15)	Cr1	C2^6	1.9909(13)
C2_1	F2_1	1.3379(15)	O1	C1	1.124(3)
C3_1	F5_1	1.3300(15)	O2	C2	1.1204(17)
C3_1	F4_1	1.3355(15)	C3	O3	1.124(3)

[1]1/2+Y,1-X,2-Z; [2]1-Y,-1/2+X,2-Z; [3]3/2-X,1/2-Y,+Z; [4]1/2-X,1/2-Y,+Z; [5]+Y,1/2-X,+Z; [6]1/2-Y,+X,+Z

Bond Angles for p4n_a_final.

Atom	Atom	Atom	Angle/°	Atom	Atom	Atom	Angle/°
O1_1[1]	Al1	O1_1[2]	112.10(6)	F7_1	C4_1	F9_1	107.94(11)
O1_1[1]	Al1	O1_1[3]	108.17(3)	F7_1	C4_1	F8_1	107.27(11)
O1_1[2]	Al1	O1_1[3]	108.17(3)	F9_1	C4_1	F8_1	107.56(11)
O1_1[1]	Al1	O1_1	108.17(3)	F7_1	C4_1	C1_1	110.87(11)
O1_1[2]	Al1	O1_1	108.17(3)	F9_1	C4_1	C1_1	110.31(10)
O1_1[3]	Al1	O1_1	112.10(6)	F8_1	C4_1	C1_1	112.70(10)
C1_1	O1_1	Al1	146.63(8)	C1	Cr1	C3	180.0
O1_1	C1_1	C2_1	111.22(10)	C1	Cr1	C2	89.95(4)
O1_1	C1_1	C4_1	111.19(10)	C3	Cr1	C2	90.05(4)
C2_1	C1_1	C4_1	108.89(10)	C1	Cr1	C2[4]	89.95(4)
O1_1	C1_1	C3_1	107.79(10)	C3	Cr1	C2[4]	90.05(4)
C2_1	C1_1	C3_1	108.92(10)	C2	Cr1	C2[4]	179.91(7)
C4_1	C1_1	C3_1	108.78(10)	C1	Cr1	C2[5]	89.95(4)
F1_1	C2_1	F3_1	107.22(11)	C3	Cr1	C2[5]	90.05(4)
F1_1	C2_1	F2_1	107.47(10)	C2	Cr1	C2[5]	90.0
F3_1	C2_1	F2_1	107.59(11)	C2[4]	Cr1	C2[5]	90.0
F1_1	C2_1	C1_1	111.14(10)	C1	Cr1	C2[6]	89.95(4)
F3_1	C2_1	C1_1	111.04(10)	C3	Cr1	C2[6]	90.05(4)
F2_1	C2_1	C1_1	112.15(10)	C2	Cr1	C2[6]	90.0
F5_1	C3_1	F4_1	107.20(10)	C2[4]	Cr1	C2[6]	90.000(1)
F5_1	C3_1	F6_1	107.39(10)	C2[5]	Cr1	C2[6]	179.91(7)
F4_1	C3_1	F6_1	107.34(11)	O1	C1	Cr1	180.0
F5_1	C3_1	C1_1	111.28(10)	O2	C2	Cr1	179.53(13)
F4_1	C3_1	C1_1	110.84(10)	O3	C3	Cr1	180.0
F6_1	C3_1	C1_1	112.53(10)				

[1]1/2+Y,1-X,2-Z; [2]1-Y,-1/2+X,2-Z; [3]3/2-X,1/2-Y,+Z; [4]1/2-X,1/2-Y,+Z; [5]+Y,1/2-X,+Z; [6]1/2-Y,+X,+Z

Torsion Angles for p4n_a_final.

A	B	C	D	Angle/°	A	B	C	D	Angle/°
O1_1[1]	Al1	O1_1	C1_1	-157.73(16)	C4_1	C1_1	C3_1	F5_1	163.12(10)
O1_1[2]	Al1	O1_1	C1_1	-36.10(13)	O1_1	C1_1	C3_1	F4_1	42.99(13)
O1_1[3]	Al1	O1_1	C1_1	83.08(15)	C2_1	C1_1	C3_1	F4_1	163.77(10)
Al1	O1_1	C1_1	C2_1	61.36(18)	C4_1	C1_1	C3_1	F4_1	-77.67(13)
Al1	O1_1	C1_1	C4_1	-60.17(18)	O1_1	C1_1	C3_1	F6_1	163.20(10)
Al1	O1_1	C1_1	C3_1	-179.32(11)	C2_1	C1_1	C3_1	F6_1	-76.02(13)
O1_1	C1_1	C2_1	F1_1	-78.96(13)	C4_1	C1_1	C3_1	F6_1	42.53(14)
C4_1	C1_1	C2_1	F1_1	43.90(13)	O1_1	C1_1	C4_1	F7_1	-74.82(13)
C3_1	C1_1	C2_1	F1_1	162.39(10)	C2_1	C1_1	C4_1	F7_1	162.30(10)
O1_1	C1_1	C2_1	F3_1	40.30(14)	C3_1	C1_1	C4_1	F7_1	43.73(13)
C4_1	C1_1	C2_1	F3_1	163.16(10)	O1_1	C1_1	C4_1	F9_1	44.69(14)
C3_1	C1_1	C2_1	F3_1	-78.35(12)	C2_1	C1_1	C4_1	F9_1	-78.19(13)
O1_1	C1_1	C2_1	F2_1	160.71(10)	C3_1	C1_1	C4_1	F9_1	163.24(11)
C4_1	C1_1	C2_1	F2_1	-76.43(13)	O1_1	C1_1	C4_1	F8_1	164.92(10)
C3_1	C1_1	C2_1	F2_1	42.06(14)	C2_1	C1_1	C4_1	F8_1	42.04(14)
O1_1	C1_1	C3_1	F5_1	-76.21(12)	C3_1	C1_1	C4_1	F8_1	-76.53(13)
C2_1	C1_1	C3_1	F5_1	44.57(13)					

[1]1/2+Y,1-X,2-Z; [2]1-Y,-1/2+X,2-Z; [3]3/2-X,1/2-Y,+Z

Crystal structure of 2, the symmetry-generated atom sites are greyed out.

Supplementary Figure 68 shows the crystal structure of **2**, Supplementary Data 3 gives full information on the crystallographic data.

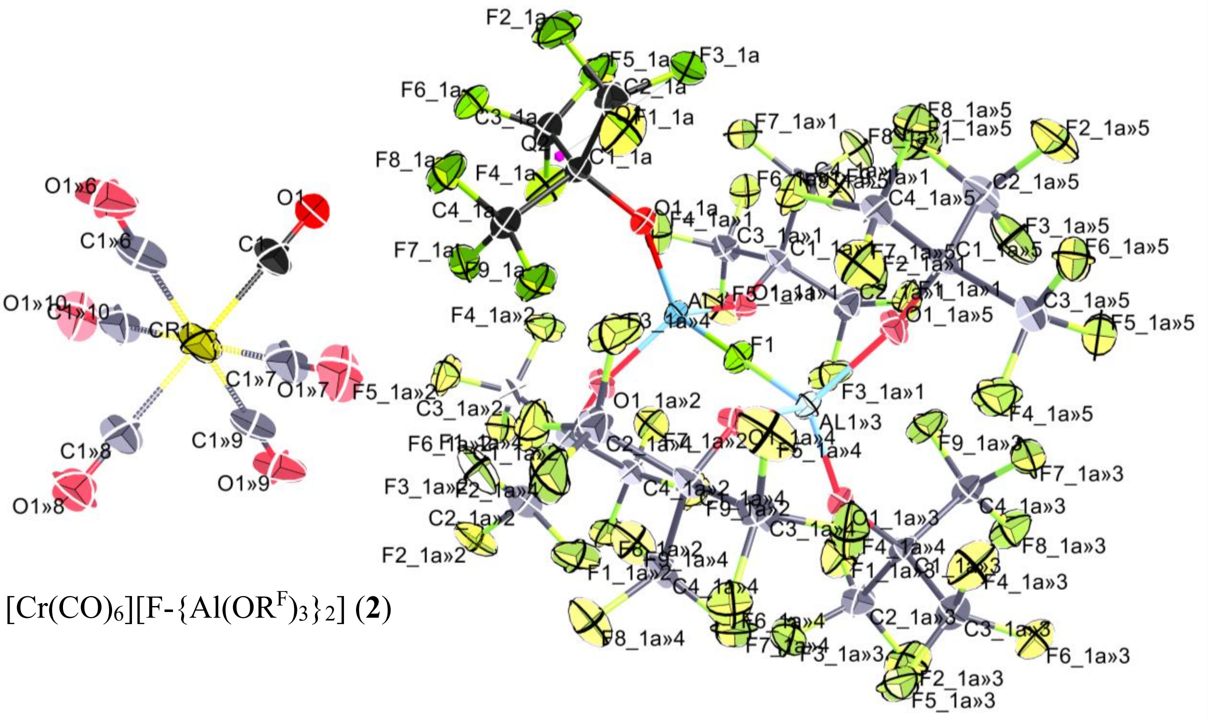

[Cr(CO)$_6$][F-{Al(OR^F)$_3$}$_2$] (**2**)

Supplementary Figure 68. Crystal structure of **2**.

Crystal data and structure refinement for pa-3_a_final.

Identification code	pa-3_a_final
Empirical formula	$C_{30}Al_2CrF_{55}O_{12}$
Formula weight	1703.26
Temperature/K	100(2)
Crystal system	cubic
Space group	Pa-3
a/Å	17.2568(3)
b/Å	17.2568(3)
c/Å	17.2568(3)
α/°	90
β/°	90
γ/°	90
Volume/Å^3	5139.0(3)
Z	4
ρ_{calc}g/cm^3	2.201
μ/mm^{-1}	0.514
F(000)	3284.0
Crystal size/mm^3	0.1 × 0.1 × 0.1
Radiation	MoKα (λ = 0.71073)
2Θ range for data collection/°	4.088 to 57.584
Index ranges	$-23 \le h \le 23$, $-21 \le k \le 22$, $-22 \le l \le 23$
Reflections collected	97143
Independent reflections	2246 [R_{int} = 0.0947, R_{sigma} = 0.0150]
Data/restraints/parameters	2246/518/267
Goodness-of-fit on F^2	1.138
Final R indexes [I>=2σ (I)]	R_1 = 0.0303, wR_2 = 0.0772
Final R indexes [all data]	R_1 = 0.0344, wR_2 = 0.0788
Largest diff. peak/hole / e Å^{-3}	0.27/-0.29

Supplementary Data 3. Information on the crystallographic data of **2**.

Fractional Atomic Coordinates (×10^4) and Equivalent Isotropic Displacement Parameters (Å^2×10^3) for pa-3_a_final. U_{eq} is defined as 1/3 of of the trace of the orthogonalised U_{IJ} tensor.

Atom	*x*	*y*	*z*	U(eq)
Al1	588.6(2)	9411.4(2)	4411.4(2)	16.82(14)
F1	0	10000	5000	22.8(4)
O1_1	921(14)	8709(13)	5036(14)	24.9(11)
C1_1	1518(9)	8367(8)	5421(8)	22.1(8)
C2_1	1186(2)	8000(2)	6160(2)	32.4(8)
F1_1	1059(5)	8528(3)	6695(5)	43.2(12)
F2_1	1681(3)	7481(2)	6451(2)	45.3(9)
F3_1	533(6)	7612(9)	6009(11)	43.4(17)
C3_1	1870(2)	7740(2)	4885(2)	29.6(8)
F4_1	1928(14)	8031(11)	4171(7)	40(2)
F5_1	1415.1(19)	7113.0(16)	4856(2)	39.4(7)
F6_1	2595(5)	7563(5)	5086(9)	30.7(12)
C4_1	2132.4(19)	8980(2)	5623.5(19)	26.9(8)
F7_1	2567.3(14)	9156(2)	5012.2(16)	35.0(6)
F8_1	2611(2)	8730(3)	6174(2)	36.9(8)
F9_1	1803(8)	9629(7)	5885(7)	34.7(14)
O1_2	851(9)	8662(8)	4985(9)	24.9(11)
F4_2	2564.7(10)	8782.7(15)	4769.4(14)	43.9(5)
C1_2	1425(6)	8305(5)	5383(5)	22.1(8)
F5_2	1939(10)	7890(8)	4167(5)	40.2(15)
F6_2	2583(4)	7564(4)	5197(6)	47(2)
C3_2	2146.2(16)	8138.4(18)	4865.0(15)	32.7(6)
F1_2	1066.3(17)	6999.2(11)	5119.3(13)	43.8(5)
C2_2	1102.9(17)	7520.3(15)	5690.2(15)	33.6(6)
F7_2	1173(4)	8778(2)	6660(3)	47.9(10)
F8_2	1758(7)	9537(5)	5856(6)	50(2)
F9_2	2373.0(17)	8576(2)	6377.3(19)	45.0(7)
F3_2	384(4)	7629(7)	5968(7)	44.3(13)
F2_2	1540.8(19)	7215.6(16)	6251.8(17)	45.1(6)
C4_2	1690.5(15)	8798.2(15)	6092.4(16)	33.9(6)
Cr1	5000	10000	5000	42.82(18)
O1	4174.2(9)	8476.7(9)	4517.4(11)	63.2(4)
C1	4475.4(11)	9028.1(12)	4685.0(12)	49.8(5)

Anisotropic Displacement Parameters (Å^2×10^3) for pa-3_a_final. The Anisotropic displacement factor exponent takes the form: $-2\pi^2[h^2a^{*2}U_{11}+2hka^*b^*U_{12}+\ldots]$.

Atom	U_{11}	U_{22}	U_{33}	U_{23}	U_{13}	U_{12}
Al1	16.82(14)	16.82(14)	16.82(14)	-2.05(13)	2.05(13)	2.05(13)
F1	22.8(4)	22.8(4)	22.8(4)	-4.8(4)	4.8(4)	4.8(4)
O1_1	19(2)	22.8(15)	32.4(18)	5.1(13)	-5.7(14)	1.7(15)
C1_1	20(2)	22.5(13)	23.8(10)	-0.8(8)	-4.8(13)	3.7(9)
C2_1	31.9(18)	35.8(19)	29.6(18)	7.3(14)	2.1(14)	4.3(14)
F1_1	44(3)	59(3)	26.2(15)	-1(2)	9.2(15)	14(3)
F2_1	52(2)	49(2)	35(2)	19.7(16)	-2.9(14)	12.7(17)
F3_1	30(3)	47(2)	53(3)	26(2)	5(3)	0(2)
C3_1	26.0(17)	31.5(19)	31.2(18)	-4.5(15)	-4.0(14)	7.3(15)
F4_1	50(3)	47(6)	24(2)	-2(2)	1.1(19)	17(4)
F5_1	39.8(16)	22.4(12)	55.9(19)	-8.8(12)	-13.5(13)	6.1(11)
F6_1	30(2)	32(2)	30(3)	-1.7(16)	0.9(15)	11.4(19)
C4_1	25.4(16)	27.3(16)	28.0(16)	-3.5(13)	-5.7(13)	5.9(12)
F7_1	24.9(11)	39.6(16)	40.4(14)	4.7(12)	-0.1(10)	-5.7(11)
F8_1	38(2)	37.0(17)	36.0(19)	-4.9(13)	-16.6(14)	7.8(15)
F9_1	38(3)	23(2)	43(3)	-12(2)	-9(2)	8(2)
O1_2	19(2)	22.8(15)	32.4(18)	5.1(13)	-5.7(14)	1.7(15)
F4_2	26.1(8)	47.0(12)	58.6(13)	6.9(10)	5.9(8)	2.6(8)
C1_2	20(2)	22.5(13)	23.8(10)	-0.8(8)	-4.8(13)	3.7(9)
F5_2	44.1(19)	48(4)	28.6(16)	-9.9(15)	0.1(13)	15(3)
F6_2	44(2)	60(3)	38(3)	-11.3(15)	-9.8(17)	35(2)
C3_2	28.2(13)	36.2(15)	33.8(13)	-1.5(11)	-3.4(10)	10.4(12)
F1_2	64.7(16)	21.9(8)	44.7(12)	-0.9(7)	-15.1(10)	-0.8(9)
C2_2	42.1(15)	28.3(12)	30.4(13)	5.2(10)	-7.4(11)	3.1(11)
F7_2	52(2)	57(2)	34.8(16)	-17.6(19)	1.0(12)	11.2(18)
F8_2	55(3)	28(2)	68(3)	-16.9(16)	-16.6(18)	0.5(16)

F9_2	37.5(15)	52.3(17)	45.1(17)	-17.2(12)	-19.2(11)	11.5(12)
F3_2	35(3)	52(2)	46.0(18)	15.5(15)	2(2)	-4(2)
F2_2	59.2(15)	38.0(14)	38.2(14)	13.1(10)	-14.3(10)	7.5(11)
C4_2	30.4(13)	33.0(13)	38.2(14)	-8.2(11)	-6.8(11)	6.8(10)
Cr1	42.82(18)	42.82(18)	42.82(18)	10.95(15)	10.95(15)	-10.95(15)
O1	50.3(8)	47.6(8)	91.7(12)	4.3(8)	12.1(8)	-13.6(7)
C1	42.9(10)	48.4(10)	58.1(11)	13.1(9)	12.5(8)	-5.3(8)

Bond Lengths for pa-3_a_final.

Atom	Atom	Length/Å	Atom	Atom	Length/Å
Al1	O1_2	1.690(12)	O1_2	C1_2	1.354(8)
Al1	O1_2[1]	1.690(12)	F4_2	C3_2	1.336(4)
Al1	O1_2[2]	1.690(13)	C1_2	C2_2	1.557(8)
Al1	O1_1	1.721(18)	C1_2	C3_2	1.559(8)
Al1	O1_1[1]	1.721(18)	C1_2	C4_2	1.559(8)
Al1	O1_1[2]	1.721(18)	F5_2	C3_2	1.327(10)
Al1	F1	1.7594(6)	F6_2	C3_2	1.371(7)
O1_1	C1_1	1.360(12)	F1_2	C2_2	1.335(3)
C1_1	C2_1	1.535(12)	C2_2	F2_2	1.337(4)
C1_1	C4_1	1.538(11)	C2_2	F3_2	1.343(9)
C1_1	C3_1	1.548(12)	F7_2	C4_2	1.326(6)
C2_1	F1_1	1.317(7)	F8_2	C4_2	1.344(9)
C2_1	F2_1	1.336(5)	F9_2	C4_2	1.333(4)
C2_1	F3_1	1.336(11)	Cr1	C1	1.982(2)
C3_1	F6_1	1.334(9)	Cr1	C1[3]	1.982(2)
C3_1	F4_1	1.334(12)	Cr1	C1[4]	1.982(2)
C3_1	F5_1	1.338(6)	Cr1	C1[5]	1.982(2)
C4_1	F7_1	1.330(4)	Cr1	C1[6]	1.982(2)
C4_1	F8_1	1.332(5)	Cr1	C1[7]	1.982(2)
C4_1	F9_1	1.334(10)	O1	C1	1.122(2)

[1]1/2-Z,1-X,-1/2+Y; [2]1-Y,1/2+Z,1/2-X; [3]1-X,2-Y,1-Z; [4]+Z,3/2-X,-1/2+Y; [5]1-Z,1/2+X,3/2-Y; [6]3/2-Y,1/2+Z,+X; [7]-1/2+Y,3/2-Z,1-X

Bond Angles for pa-3_a_final.

Atom	Atom	Atom	Angle/°	Atom	Atom	Atom	Angle/°
O1_2	Al1	O1_2[1]	113.6(5)	C1_2	O1_2	Al1	147.3(12)
O1_2	Al1	O1_1[1]	109.0(12)	O1_2	C1_2	C2_2	107.9(8)
O1_2[1]	Al1	O1_1[1]	5.6(13)	O1_2	C1_2	C3_2	112.1(10)
O1_2[2]	Al1	O1_1[1]	119.0(12)	C2_2	C1_2	C3_2	108.6(6)
O1_1	Al1	O1_1[1]	114.5(7)	O1_2	C1_2	C4_2	111.4(9)
O1_2	Al1	O1_1[2]	119.0(12)	C2_2	C1_2	C4_2	108.2(6)
O1_2[1]	Al1	O1_1[2]	109.0(12)	C3_2	C1_2	C4_2	108.5(6)
O1_2[2]	Al1	O1_1[2]	5.6(13)	F5_2	C3_2	F4_2	107.6(6)
O1_1	Al1	O1_1[2]	114.5(7)	F5_2	C3_2	F6_2	107.1(7)
O1_1[1]	Al1	O1_1[2]	114.5(7)	F4_2	C3_2	F6_2	110.8(4)
O1_2	Al1	F1	105.0(6)	F5_2	C3_2	C1_2	111.4(8)
O1_2[1]	Al1	F1	105.0(6)	F4_2	C3_2	C1_2	110.4(4)
O1_2[2]	Al1	F1	105.0(6)	F6_2	C3_2	C1_2	109.4(6)
O1_1	Al1	F1	103.8(9)	F1_2	C2_2	F2_2	107.3(2)
O1_1[1]	Al1	F1	103.8(9)	F1_2	C2_2	F3_2	108.3(5)
O1_1[2]	Al1	F1	103.8(9)	F2_2	C2_2	F3_2	108.5(6)
Al1[3]	F1	Al1	180.0	F1_2	C2_2	C1_2	110.6(3)
C1_1	O1_1	Al1	149.8(18)	F2_2	C2_2	C1_2	112.8(5)
O1_1	C1_1	C2_1	107.6(13)	F3_2	C2_2	C1_2	109.2(7)
O1_1	C1_1	C4_1	109.6(14)	F7_2	C4_2	F9_2	108.3(4)
C2_1	C1_1	C4_1	110.7(9)	F7_2	C4_2	F8_2	107.9(5)
O1_1	C1_1	C3_1	107.9(14)	F9_2	C4_2	F8_2	108.0(6)
C2_1	C1_1	C3_1	110.7(9)	F7_2	C4_2	C1_2	111.6(4)
C4_1	C1_1	C3_1	110.2(8)	F9_2	C4_2	C1_2	113.1(5)
F1_1	C2_1	F2_1	107.8(5)	F8_2	C4_2	C1_2	107.8(6)
F1_1	C2_1	F3_1	110.0(9)	C1	Cr1	C1[4]	180.0
F2_1	C2_1	F3_1	106.1(8)	C1	Cr1	C1[5]	91.67(8)
F1_1	C2_1	C1_1	111.0(6)	C1[4]	Cr1	C1[5]	88.33(8)
F2_1	C2_1	C1_1	110.6(8)	C1	Cr1	C1[6]	88.33(8)
F3_1	C2_1	C1_1	111.1(10)	C1[4]	Cr1	C1[6]	91.67(8)
F6_1	C3_1	F4_1	104.8(12)	C1[5]	Cr1	C1[6]	180.00(6)
F6_1	C3_1	F5_1	112.0(5)	C1	Cr1	C1[7]	91.67(8)
F4_1	C3_1	F5_1	108.3(9)	C1[4]	Cr1	C1[7]	88.33(8)

F6_1	C3_1	C1_1	111.9(9)	C1[5]	Cr1	C1[7]	88.33(8)
F4_1	C3_1	C1_1	108.6(11)	C1[6]	Cr1	C1[7]	91.67(8)
F5_1	C3_1	C1_1	110.9(5)	C1	Cr1	C1[8]	88.33(8)
F7_1	C4_1	F8_1	106.8(3)	C1[4]	Cr1	C1[8]	91.67(8)
F7_1	C4_1	F9_1	108.4(6)	C1[5]	Cr1	C1[8]	91.67(8)
F8_1	C4_1	F9_1	107.2(7)	C1[6]	Cr1	C1[8]	88.33(8)
F7_1	C4_1	C1_1	111.5(5)	C1[7]	Cr1	C1[8]	180.0
F8_1	C4_1	C1_1	111.5(8)	O1	C1	Cr1	178.98(19)
F9_1	C4_1	C1_1	111.1(9)				

[1]1/2-Z,1-X,-1/2+Y; [2]1-Y,1/2+Z,1/2-X; [3]-X,2-Y,1-Z; [4]1-X,2-Y,1-Z; [5]+Z,3/2-X,-1/2+Y; [6]1-Z,1/2+X,3/2-Y; [7]3/2-Y,1/2+Z,+X; [8]-1/2+Y,3/2-Z,1-X

Torsion Angles for pa-3_a_final.

A	B	C	D	Angle/°	A	B	C	D	Angle/°
O1_1[1]	Al1	O1_1	C1_1	134(3)	O1_2[1]	Al1	O1_2	C1_2	136.3(16)
O1_1[2]	Al1	O1_1	C1_1	-2(5)	O1_2[2]	Al1	O1_2	C1_2	5(3)
F1	Al1	O1_1	C1_1	-114(4)	F1	Al1	O1_2	C1_2	-110(2)
Al1	O1_1	C1_1	C2_1	152(3)	Al1	O1_2	C1_2	C2_2	-173(2)
Al1	O1_1	C1_1	C4_1	32(4)	Al1	O1_2	C1_2	C3_2	-53(3)
Al1	O1_1	C1_1	C3_1	-88(4)	Al1	O1_2	C1_2	C4_2	68(3)
O1_1	C1_1	C2_1	F1_1	-77.6(13)	O1_2	C1_2	C3_2	F5_2	-41.2(11)
C4_1	C1_1	C2_1	F1_1	42.2(13)	C2_2	C1_2	C3_2	F5_2	77.9(9)
C3_1	C1_1	C2_1	F1_1	164.7(8)	C4_2	C1_2	C3_2	F5_2	-164.7(7)
O1_1	C1_1	C2_1	F2_1	162.8(12)	O1_2	C1_2	C3_2	F4_2	78.2(8)
C4_1	C1_1	C2_1	F2_1	-77.5(10)	C2_2	C1_2	C3_2	F4_2	-162.6(4)
C3_1	C1_1	C2_1	F2_1	45.0(11)	C4_2	C1_2	C3_2	F4_2	-45.2(8)
O1_1	C1_1	C2_1	F3_1	45.2(15)	O1_2	C1_2	C3_2	F6_2	-159.5(9)
C4_1	C1_1	C2_1	F3_1	165.0(11)	C2_2	C1_2	C3_2	F6_2	-40.4(8)
C3_1	C1_1	C2_1	F3_1	-72.5(14)	C4_2	C1_2	C3_2	F6_2	77.0(7)
O1_1	C1_1	C3_1	F6_1	158.6(12)	O1_2	C1_2	C2_2	F1_2	76.3(9)
C2_1	C1_1	C3_1	F6_1	-83.9(10)	C3_2	C1_2	C2_2	F1_2	-45.5(8)
C4_1	C1_1	C3_1	F6_1	38.9(13)	C4_2	C1_2	C2_2	F1_2	-163.0(5)
O1_1	C1_1	C3_1	F4_1	43.4(16)	O1_2	C1_2	C2_2	F2_2	-163.6(9)
C2_1	C1_1	C3_1	F4_1	160.9(13)	C3_2	C1_2	C2_2	F2_2	74.6(6)
C4_1	C1_1	C3_1	F4_1	-76.3(15)	C4_2	C1_2	C2_2	F2_2	-43.0(7)
O1_1	C1_1	C3_1	F5_1	-75.6(13)	O1_2	C1_2	C2_2	F3_2	-42.8(11)
C2_1	C1_1	C3_1	F5_1	42.0(12)	C3_2	C1_2	C2_2	F3_2	-164.6(7)
C4_1	C1_1	C3_1	F5_1	164.8(7)	C4_2	C1_2	C2_2	F3_2	77.8(8)
O1_1	C1_1	C4_1	F7_1	-78.0(13)	O1_2	C1_2	C4_2	F7_2	73.9(10)
C2_1	C1_1	C4_1	F7_1	163.5(7)	C2_2	C1_2	C4_2	F7_2	-44.5(8)
C3_1	C1_1	C4_1	F7_1	40.7(13)	C3_2	C1_2	C4_2	F7_2	-162.2(5)
O1_1	C1_1	C4_1	F8_1	162.7(13)	O1_2	C1_2	C4_2	F9_2	-163.6(8)
C2_1	C1_1	C4_1	F8_1	44.1(11)	C2_2	C1_2	C4_2	F9_2	77.9(6)
C3_1	C1_1	C4_1	F8_1	-78.7(10)	C3_2	C1_2	C4_2	F9_2	-39.7(8)
O1_1	C1_1	C4_1	F9_1	43.1(15)	O1_2	C1_2	C4_2	F8_2	-44.4(10)
C2_1	C1_1	C4_1	F9_1	-75.4(13)	C2_2	C1_2	C4_2	F8_2	-162.8(7)
C3_1	C1_1	C4_1	F9_1	161.8(9)	C3_2	C1_2	C4_2	F8_2	79.5(8)

[1]1/2-Z,1-X,-1/2+Y; [2]1-Y,1/2+Z,1/2-X

Table 7 Atomic Occupancy for pa-3_a_final.

Atom	*Occupancy*	Atom	*Occupancy*	Atom	*Occupancy*
O1_1	0.408(3)	C1_1	0.408(3)	C2_1	0.408(3)
F1_1	0.408(3)	F2_1	0.408(3)	F3_1	0.408(3)
C3_1	0.408(3)	F4_1	0.408(3)	F5_1	0.408(3)
F6_1	0.408(3)	C4_1	0.408(3)	F7_1	0.408(3)
F8_1	0.408(3)	F9_1	0.408(3)	O1_2	0.592(3)
F4_2	0.592(3)	C1_2	0.592(3)	F5_2	0.592(3)
F6_2	0.592(3)	C3_2	0.592(3)	F1_2	0.592(3)
C2_2	0.592(3)	F7_2	0.592(3)	F8_2	0.592(3)
F9_2	0.592(3)	F3_2	0.592(3)	F2_2	0.592(3)
C4_2	0.592(3)				

Crystal structure of 3, the symmetry-generated atom sites are greyed out. Note that the nitrogen position cannot be crystallographically unambiguously assigned.

Supplementary Figure 69 shows the crystal structure of **3**, Supplementary Data 4 gives full information on the crystallographic data.

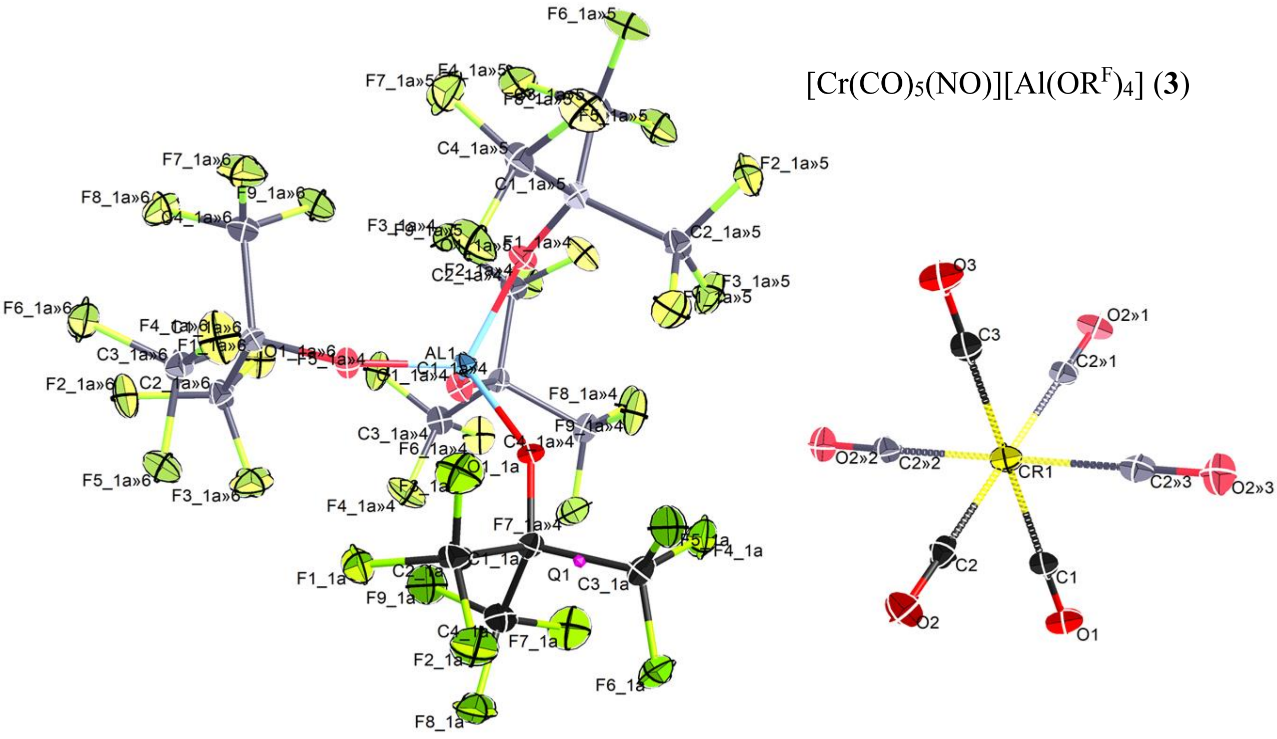

Supplementary Figure 69. Crystal structure of **3**.

Crystal data and structure refinement for p4n_a_final.

Identification code	p4n_a_final
Empirical formula	$C_{22}AlCrF_{36}O_{10}$
Formula weight	1187.20
Temperature/K	100(2)
Crystal system	tetragonal
Space group	P4/n
a/Å	13.6348(7)
b/Å	13.6348(7)
c/Å	9.5070(5)
α/°	90
β/°	90
γ/°	90
Volume/Å^3	1767.4(2)
Z	2
ρ_{calc}g/cm^3	2.231
μ/mm^{-1}	0.593
F(000)	1146.0
Crystal size/mm^3	0.15 × 0.1 × 0.1
Radiation	MoKα (λ = 0.71073)
2Θ range for data collection/°	4.224 to 56.6
Index ranges	-16 ≤ h ≤ 18, -15 ≤ k ≤ 18, -12 ≤ l ≤ 12
Reflections collected	11645
Independent reflections	2202 [R_{int} = 0.0165, R_{sigma} = 0.0112]
Data/restraints/parameters	2202/189/164
Goodness-of-fit on F^2	1.071
Final R indexes [I>=2σ (I)]	R_1 = 0.0252, wR_2 = 0.0688
Final R indexes [all data]	R_1 = 0.0267, wR_2 = 0.0698
Largest diff. peak/hole / e Å^{-3}	0.45/-0.45

Supplementary Data 4. Information on the crystallographic data of **3**.

Fractional Atomic Coordinates (×10⁴) and Equivalent Isotropic Displacement Parameters (Å²×10³) for p4n_a_final. U_{eq} is defined as 1/3 of of the trace of the orthogonalised U_{IJ} tensor.

Atom	*x*	*y*	*z*	U(eq)
O1_1	3333.0(6)	6856.0(6)	1017.4(9)	16.22(18)
C1_1	4177.0(8)	6885.8(8)	1757.3(12)	14.0(2)
C2_1	5059.8(9)	7162.8(9)	799.5(13)	18.8(2)
F1_1	5084.2(6)	8126.0(6)	558.7(9)	25.54(19)
F2_1	5920.5(6)	6912.1(7)	1373.6(9)	27.12(19)
F3_1	4995.8(6)	6719.1(7)	-443.4(8)	27.86(19)
C3_1	4357.2(9)	5843.9(9)	2384.9(13)	18.6(2)
F4_1	3526.9(6)	5471.0(6)	2902.3(9)	27.48(19)
F5_1	4680.9(6)	5226.5(6)	1408.9(9)	25.18(19)
F6_1	5017.3(6)	5851.2(6)	3432.2(9)	26.17(19)
C4_1	4111(1)	7639.0(9)	2987.7(14)	20.1(3)
F7_1	3565.9(7)	7288.5(7)	4027.9(9)	31.0(2)
F8_1	4995.5(6)	7863.9(6)	3527.8(9)	27.33(19)
F9_1	3703.6(7)	8468.6(6)	2543.6(9)	28.9(2)
C3	2500	2500	1670(3)	20.0(5)
Cr1	2500	2500	3694.0(4)	17.15(10)
Al1	2500	7500	0	11.73(14)
O1	2500	2500	6936(2)	27.2(4)
C1	2500	2500	5754(3)	18.9(5)
O2	4553.0(7)	3455.3(8)	3691.5(10)	26.8(2)
C2	3805.8(10)	3104.9(9)	3697.0(13)	19.0(2)
O3	2500	2500	482(2)	30.6(5)

Anisotropic Displacement Parameters (Å²×10³) for p4n_a_final. The Anisotropic displacement factor exponent takes the form: $-2\pi^2[h^2a^{*2}U_{11}+2hka^*b^*U_{12}+\ldots]$.

Atom	U_{11}	U_{22}	U_{33}	U_{23}	U_{13}	U_{12}
O1_1	12.8(4)	15.3(4)	20.6(4)	1.8(3)	-3.7(3)	0.4(3)
C1_1	12.9(5)	13.9(5)	15.3(5)	-0.8(4)	-0.6(4)	1.4(4)
C2_1	14.4(5)	21.1(6)	20.9(6)	0.0(5)	-0.4(4)	-0.6(4)
F1_1	22.4(4)	21.8(4)	32.5(4)	6.6(3)	-1.7(3)	-6.6(3)
F2_1	12.2(3)	33.9(5)	35.3(5)	2.4(4)	-1.3(3)	2.5(3)
F3_1	28.2(4)	36.0(5)	19.4(4)	-5.4(3)	6.0(3)	-2.2(3)
C3_1	18.3(6)	16.7(5)	20.9(6)	1.4(4)	-3.1(4)	2.0(4)
F4_1	24.7(4)	23.2(4)	34.6(4)	11.9(3)	1.4(3)	-2.3(3)
F5_1	28.9(4)	16.6(4)	30.0(4)	-4.8(3)	-4.2(3)	7.4(3)
F6_1	28.3(4)	24.4(4)	25.7(4)	3.5(3)	-11.9(3)	4.7(3)
C4_1	21.5(6)	18.7(6)	20.2(6)	-3.9(5)	-2.0(5)	3.2(5)
F7_1	34.7(5)	36.5(5)	21.9(4)	-4.3(3)	9.8(3)	2.9(4)
F8_1	28.3(4)	26.6(4)	27.1(4)	-7.8(3)	-9.9(3)	-0.6(3)
F9_1	34.1(5)	17.3(4)	35.2(4)	-6.8(3)	-5.8(4)	9.6(3)
C3	17.9(7)	17.9(7)	24.1(12)	0	0	0
Cr1	17.36(13)	17.36(13)	16.74(19)	0	0	0
Al1	10.70(19)	10.70(19)	13.8(3)	0	0	0
O1	31.2(6)	31.2(6)	19.2(9)	0	0	0
C1	17.2(7)	17.2(7)	22.3(12)	0	0	0
O2	21.8(5)	29.4(5)	29.0(5)	1.8(4)	0.7(4)	-3.0(4)
C2	22.7(6)	18.4(6)	15.8(5)	1.0(4)	0.8(4)	2.6(5)
O3	36.0(7)	36.0(7)	19.7(9)	0	0	0

Bond Lengths for p4n_a_final.

Atom	Atom	Length/Å	Atom	Atom	Length/Å
O1_1	C1_1	1.3493(14)	C4_1	F9_1	1.3290(15)
O1_1	Al1	1.7311(8)	C4_1	F8_1	1.3462(15)
C1_1	C2_1	1.5559(16)	C3	O3	1.130(3)
C1_1	C4_1	1.5591(16)	C3	Cr1	1.924(3)
C1_1	C3_1	1.5603(16)	Cr1	C1	1.958(3)
C2_1	F3_1	1.3304(15)	Cr1	C2	1.9621(13)
C2_1	F1_1	1.3335(15)	Cr1	$C2^1$	1.9622(13)
C2_1	F2_1	1.3386(14)	Cr1	$C2^2$	1.9622(13)
C3_1	F5_1	1.3283(15)	Cr1	$C2^3$	1.9622(13)
C3_1	F4_1	1.3349(15)	O1	C1	1.124(3)
C3_1	F6_1	1.3422(14)	O2	C2	1.1253(17)

C4_1	F7_1	1.3262(16)

[1]1/2-X,1/2-Y,+Z; [2]+Y,1/2-X,+Z; [3]1/2-Y,+X,+Z

Bond Angles for p4n_a_final.

Atom	Atom	Atom	Angle/°	Atom	Atom	Atom	Angle/°
C1_1	O1_1	Al1	146.67(8)	O3	C3	Cr1	180.0
O1_1	C1_1	C2_1	111.23(10)	C3	Cr1	C1	180.0
O1_1	C1_1	C4_1	111.21(10)	C3	Cr1	C2	90.08(4)
C2_1	C1_1	C4_1	108.89(10)	C1	Cr1	C2	89.92(4)
O1_1	C1_1	C3_1	107.83(9)	C3	Cr1	C2[1]	90.08(4)
C2_1	C1_1	C3_1	108.84(10)	C1	Cr1	C2[1]	89.92(4)
C4_1	C1_1	C3_1	108.78(10)	C2	Cr1	C2[1]	179.83(8)
F3_1	C2_1	F1_1	107.27(10)	C3	Cr1	C2[2]	90.08(4)
F3_1	C2_1	F2_1	107.67(10)	C1	Cr1	C2[2]	89.92(4)
F1_1	C2_1	F2_1	107.44(10)	C2	Cr1	C2[2]	90.0
F3_1	C2_1	C1_1	111.01(10)	C2[1]	Cr1	C2[2]	90.0
F1_1	C2_1	C1_1	111.03(10)	C3	Cr1	C2[3]	90.08(4)
F2_1	C2_1	C1_1	112.19(10)	C1	Cr1	C2[3]	89.92(4)
F5_1	C3_1	F4_1	107.33(10)	C2	Cr1	C2[3]	90.0
F5_1	C3_1	F6_1	107.46(10)	C2[1]	Cr1	C2[3]	90.0
F4_1	C3_1	F6_1	107.35(10)	C2[2]	Cr1	C2[3]	179.83(7)
F5_1	C3_1	C1_1	111.23(10)	O1_1[4]	Al1	O1_1[5]	112.06(6)
F4_1	C3_1	C1_1	110.74(10)	O1_1[4]	Al1	O1_1[6]	108.19(3)
F6_1	C3_1	C1_1	112.49(10)	O1_1[5]	Al1	O1_1[6]	108.19(3)
F7_1	C4_1	F9_1	108.02(11)	O1_1[4]	Al1	O1_1	108.19(3)
F7_1	C4_1	F8_1	107.44(10)	O1_1[5]	Al1	O1_1	108.19(3)
F9_1	C4_1	F8_1	107.56(11)	O1_1[6]	Al1	O1_1	112.06(6)
F7_1	C4_1	C1_1	110.76(10)	O1	C1	Cr1	180.0
F9_1	C4_1	C1_1	110.27(10)	O2	C2	Cr1	179.56(12)
F8_1	C4_1	C1_1	112.62(10)				

[1]1/2-X,1/2-Y,+Z; [2]+Y,1/2-X,+Z; [3]1/2-Y,+X,+Z; [4]-1/2+Y,1-X,-Z; [5]1-Y,1/2+X,-Z; [6]1/2-X,3/2-Y,+Z

Torsion Angles for p4n_a_final.

A	B	C	D	Angle/°	A	B	C	D	Angle/°
Al1	O1_1	C1_1	C2_1	-61.41(18)	C4_1	C1_1	C3_1	F4_1	77.71(12)
Al1	O1_1	C1_1	C4_1	60.14(18)	O1_1	C1_1	C3_1	F6_1	-163.12(10)
Al1	O1_1	C1_1	C3_1	179.32(11)	C2_1	C1_1	C3_1	F6_1	76.10(12)
O1_1	C1_1	C2_1	F3_1	-40.41(13)	C4_1	C1_1	C3_1	F6_1	-42.41(13)
C4_1	C1_1	C2_1	F3_1	-163.30(10)	O1_1	C1_1	C4_1	F7_1	74.82(13)
C3_1	C1_1	C2_1	F3_1	78.26(12)	C2_1	C1_1	C4_1	F7_1	-162.27(10)
O1_1	C1_1	C2_1	F1_1	78.83(12)	C3_1	C1_1	C4_1	F7_1	-43.79(13)
C4_1	C1_1	C2_1	F1_1	-44.06(13)	O1_1	C1_1	C4_1	F9_1	-44.69(14)
C3_1	C1_1	C2_1	F1_1	-162.50(10)	C2_1	C1_1	C4_1	F9_1	78.22(13)
O1_1	C1_1	C2_1	F2_1	-160.93(10)	C3_1	C1_1	C4_1	F9_1	-163.30(10)
C4_1	C1_1	C2_1	F2_1	76.17(13)	O1_1	C1_1	C4_1	F8_1	-164.84(10)
C3_1	C1_1	C2_1	F2_1	-42.27(13)	C2_1	C1_1	C4_1	F8_1	-41.94(14)
O1_1	C1_1	C3_1	F5_1	76.26(12)	C3_1	C1_1	C4_1	F8_1	76.54(13)
C2_1	C1_1	C3_1	F5_1	-44.52(13)	C1_1	O1_1	Al1	O1_1[1]	157.66(16)
C4_1	C1_1	C3_1	F5_1	-163.03(10)	C1_1	O1_1	Al1	O1_1[2]	36.06(13)
O1_1	C1_1	C3_1	F4_1	-43.00(13)	C1_1	O1_1	Al1	O1_1[3]	-83.14(14)
C2_1	C1_1	C3_1	F4_1	-163.78(10)					

[1]-1/2+Y,1-X,-Z; [2]1-Y,1/2+X,-Z; [3]1/2-X,3/2-Y,+Z

Modelling of the NO-disorder for $[Cr(CO)_5(NO)][Al(OR^F)_4]$

A roughly equal distribution in the NO disorder (16%/4x17%/16%) led to the best model (Supplementary Figure 70). The R_1 value changed from 2.52% to 2.46% when the disorder was included. A refinement of the N positions with a free variable, however, did not lead to a stable model. Supplementary Data 5 gives full information on crystallographic data.

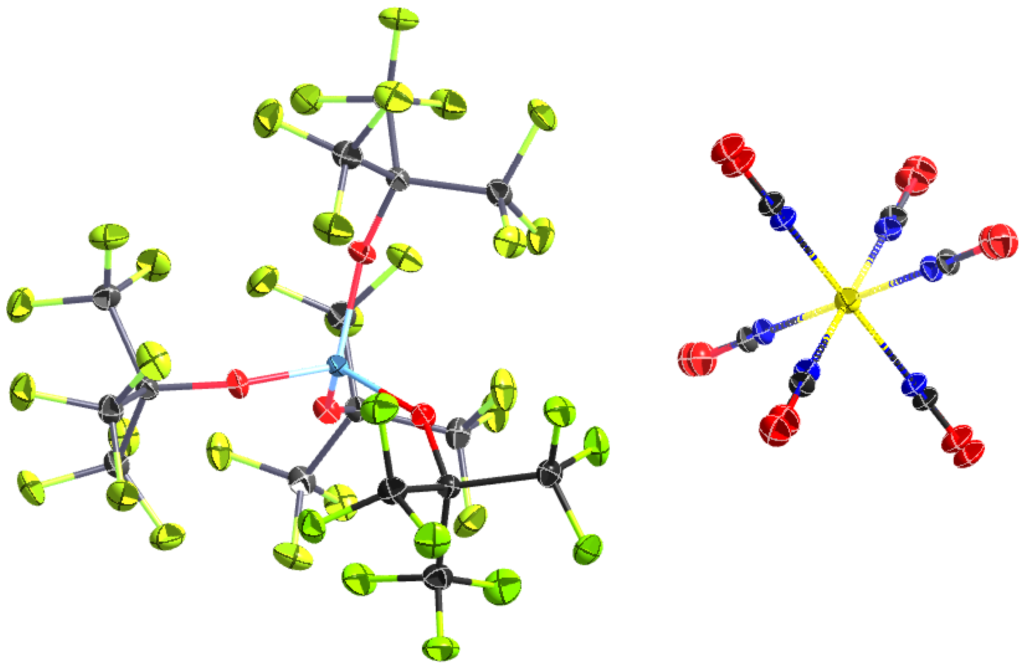

Supplementary Figure 70. Disorder model for **3**.

The resulting bond lengths are shown in Supplementary Table 8.

Supplementary Table 8. Resulting bond lengths of the disorder model for **3**.

w/o disorder [pm]		With NO disorder [pm]			
d(Cr-C1)	195.8(3)	d(Cr-C1)	199.6(3)	d(Cr-N1)	171(4)
d(Cr-C2)	196.2(1)	d(Cr-C2)	200.78(16)	d(Cr-N2)	170.2(16)
d(Cr-C3)	192.4(4)	d(Cr-C3)	196.5(5)	d(Cr-N3)	166(4)

We are aware that a disordered NO ligand describes the ‘chemical reality’ more accurately than an ‘only CO’ refinement for the $[Cr(CO)_5(NO)]^+$ system. However, based on the crystal data alone, to us it seemed not ‘scientifically correct’ to add and refine a ligand that is not differentiable by XRD means. The low bond precisions for the Cr-N bonds underlines the question, if the refinement of a disordered NO actually yields a scientifically more accurate structure model.

The disordered crystal structure was also deployed in the CCDC (Accession number: 1886541)

Crystal data and structure refinement for p4n_a_NO_disorder.

Identification code	p4n_a_NO_disorder
Empirical formula	$C_{21}O_{10}F_{36}AlCrN$
Formula weight	1189.15
Temperature/K	100.0
Crystal system	tetragonal
Space group	P4/n
a/Å	13.6348(7)
b/Å	13.6348(7)
c/Å	9.5070(5)
α/°	90
β/°	90
γ/°	90
Volume/Å^3	1767.43(16)
Z	2
ρ_{calc}g/cm^3	2.2343
μ/mm^{-1}	0.594
F(000)	1150.3
Crystal size/mm^3	0.15 × 0.1 × 0.1
Radiation	Mo Kα (λ = 0.71073)
2Θ range for data collection/°	4.22 to 56.6
Index ranges	-16 ≤ h ≤ 18, -15 ≤ k ≤ 18, -12 ≤ l ≤ 12
Reflections collected	11645
Independent reflections	2202 [R_{int} = 0.0165, R_{sigma} = 0.0112]
Data/restraints/parameters	2202/0/172
Goodness-of-fit on F^2	1.078
Final R indexes [I>=2σ (I)]	R_1 = 0.0246, wR_2 = 0.0659
Final R indexes [all data]	R_1 = 0.0261, wR_2 = 0.0669
Largest diff. peak/hole / e Å^{-3}	0.48/-0.41

Supplementary Data 5. Information on the crystallographic data of disordered **3**.

Fractional Atomic Coordinates (×10^4) and Equivalent Isotropic Displacement Parameters (Å^2×10^3) for p4n_a_NO_disorder. U_{eq} is defined as 1/3 of of the trace of the orthogonalised U_{IJ} tensor.

Atom	*x*	*y*	*z*	U(eq)
N3	2500	2500	1950(40)	16.5(11)
O6	2500	2500	730(50)	27.4(12)
N2	3623(11)	3044(14)	3690(20)	15.3(6)
O5	4450(30)	3360(20)	3700(40)	24.6(7)
N1	2500	2500	5490(40)	15.7(12)
O4	2500	2500	6650(40)	23.3(10)
C1	2500	2500	5794(4)	15.7(12)
O1	2500	2500	6975(3)	23.3(10)
C2	3837.7(13)	3116(3)	3698(4)	15.3(6)
O2	4570(5)	3471(4)	3690(8)	24.6(7)
C3	2500	2500	1627(3)	16.5(11)
O3	2500	2500	445(4)	27.4(12)
Cr1	2500	2500	3694.0(4)	17.02(10)
Al1	2500	7500	0	11.62(13)
O1_1	3333.0(6)	6856.0(6)	1017.7(9)	16.11(17)
C1_1	4177.0(8)	6885.7(8)	1757.2(12)	13.9(2)
C2_1	5059.7(9)	7162.8(9)	799.7(13)	18.7(2)
F1_1	5084.3(6)	8125.9(6)	558.9(9)	25.44(18)
F2_1	5920.4(5)	6912.0(6)	1373.3(9)	26.99(19)
F3_1	4995.4(6)	6719.4(6)	-443.7(8)	27.74(18)
C3_1	4357.3(9)	5844.1(9)	2385.3(13)	18.6(2)
F4_1	3527.0(6)	5471.1(6)	2902.4(9)	27.37(19)
F5_1	4680.7(6)	5226.6(6)	1408.8(8)	25.10(18)
F6_1	5017.2(6)	5851.0(6)	3432.1(8)	26.11(18)
C4_1	4111.1(9)	7638.6(9)	2987.9(13)	20.1(2)
F7_1	3566.3(6)	7288.6(7)	4027.9(8)	30.88(19)

F8_1	4995.4(6)	7863.7(6)	3527.5(8)	27.23(18)
F9_1	3703.5(6)	8468.5(6)	2543.7(9)	28.78(19)

Anisotropic Displacement Parameters (Å^2×10^3) for p4n_a_NO_disorder. The Anisotropic displacement factor exponent takes the form: $-2\pi^2[h^2a^{*2}U_{11}+2hka^*b^*U_{12}+\dots]$.

Atom	U_{11}	U_{22}	U_{33}	U_{12}	U_{13}	U_{23}
N3	19.4(7)	19.4(7)	11(4)	-0	-0	0
O6	35.2(7)	35.2(7)	12(3)	-0	-0	0
N2	10.8(16)	18.0(8)	17.2(5)	-2.0(11)	0.4(11)	0.8(5)
O5	19(2)	26.0(16)	28.4(5)	-5.4(9)	1.0(11)	1.9(10)
N1	18.9(7)	18.9(7)	9(4)	-0	-0	0
O4	30.4(7)	30.4(7)	9(2)	-0	-0	0
C1	18.9(7)	18.9(7)	9(4)	-0	-0	0
O1	30.4(7)	30.4(7)	9(2)	-0	-0	0
C2	10.8(16)	18.0(8)	17.2(5)	-2.0(11)	0.4(11)	0.8(5)
O2	19(2)	26.0(16)	28.4(5)	-5.4(9)	1.0(11)	1.9(10)
C3	19.4(7)	19.4(7)	11(4)	-0	-0	0
O3	35.2(7)	35.2(7)	12(3)	-0	-0	0
Cr1	17.18(13)	17.18(13)	16.71(19)	-0	-0	0
Al1	10.60(18)	10.60(18)	13.7(3)	-0	-0	0
O1_1	12.6(4)	15.2(4)	20.5(4)	0.5(3)	-3.6(3)	1.8(3)
C1_1	12.8(5)	13.8(5)	15.2(5)	1.3(4)	-0.5(4)	-0.8(4)
C2_1	14.2(5)	21.0(5)	20.8(5)	-0.5(4)	-0.3(4)	-0.1(4)
F1_1	22.2(4)	21.7(4)	32.4(4)	-6.6(3)	-1.6(3)	6.6(3)
F2_1	12.1(3)	33.8(4)	35.0(4)	2.5(3)	-1.4(3)	2.4(3)
F3_1	28.1(4)	35.9(4)	19.2(4)	-2.1(3)	6.0(3)	-5.4(3)
C3_1	18.1(5)	16.7(5)	20.9(5)	2.1(4)	-3.1(4)	1.3(4)
F4_1	24.5(4)	23.0(4)	34.5(4)	-2.3(3)	1.5(3)	12.0(3)
F5_1	28.9(4)	16.6(3)	29.9(4)	7.5(3)	-4.2(3)	-4.8(3)
F6_1	28.3(4)	24.4(4)	25.6(4)	4.6(3)	-12.0(3)	3.5(3)
C4_1	21.5(6)	18.7(5)	20.1(5)	3.3(4)	-1.9(4)	-3.9(4)
F7_1	34.6(5)	36.2(5)	21.9(4)	2.8(4)	9.7(3)	-4.3(3)
F8_1	28.2(4)	26.4(4)	27.1(4)	-0.6(3)	-9.9(3)	-7.7(3)
F9_1	34.0(4)	17.3(4)	35.1(4)	9.6(3)	-5.8(4)	-6.8(3)

Bond Lengths for p4n_a_NO_disorder.

Atom	Atom	Length/Å	Atom	Atom	Length/Å
N3	O6	1.15(4)	Al1	O1_1	1.7312(8)
N3	Cr1	1.66(4)	O1_1	C1_1	1.3491(13)
N2	O5	1.21(3)	C1_1	C2_1	1.5556(16)
N2	Cr1[1]	1.702(16)	C1_1	C3_1	1.5601(16)
N1	O4	1.10(3)	C1_1	C4_1	1.5591(16)
N1	Cr1	1.71(4)	C2_1	F1_1	1.3334(14)
C1	O1	1.124(5)	C2_1	F2_1	1.3384(14)
C1	Cr1	1.996(3)	C2_1	F3_1	1.3306(14)
C2	O2	1.111(5)	C3_1	F4_1	1.3348(14)
C2	Cr1[1]	2.0078(16)	C3_1	F5_1	1.3287(14)
C3	O3	1.124(5)	C3_1	F6_1	1.3417(14)
C3	Cr1	1.965(3)	C4_1	F7_1	1.3256(15)
Al1	O1_1[2]	1.7312(8)	C4_1	F8_1	1.3458(15)
Al1	O1_1[3]	1.7312(8)	C4_1	F9_1	1.3296(14)
Al1	O1_1[4]	1.7312(8)			

[1]1/2-X,1/2-Y,+Z; [2]1/2-X,3/2-Y,+Z; [3]-1/2+Y,1-X,-Z; [4]1-Y,1/2+X,-Z

Bond Angles for p4n_a_NO_disorder.

Atom	Atom	Atom	Angle/°	Atom	Atom	Atom	Angle/°
N2[1]	Cr1	N3	89.8(6)	O1_1	Al1	O1_1[4]	112.04(6)
N2	Cr1	N3	89.8(6)	O1_1[5]	Al1	O1_1[6]	112.04(6)
N2[2]	Cr1	N3	89.8(6)	O1_1[6]	Al1	O1_1[4]	108.20(3)
N2[3]	Cr1	N3	89.8(6)	O1_1[5]	Al1	O1_1	108.20(3)
N2[3]	Cr1	N2	90.00(12)	O1_1[6]	Al1	O1_1	108.20(3)
N2[2]	Cr1	N2	90.00(12)	O1_1[5]	Al1	O1_1[4]	108.20(3)
N2[3]	Cr1	N2[1]	90.00(12)	C2_1	C1_1	O1_1	111.24(9)
N2[3]	Cr1	N2[2]	179.6(13)	C3_1	C1_1	O1_1	107.85(9)
N2[2]	Cr1	N2[1]	90.00(12)	C3_1	C1_1	C2_1	108.85(9)
N2	Cr1	N2[1]	179.6(13)	C4_1	C1_1	O1_1	111.21(9)
N1	Cr1	N3	180.0	C4_1	C1_1	C2_1	108.89(9)
N1	Cr1	N2[2]	90.2(6)	C4_1	C1_1	C3_1	108.73(9)

N1	Cr1	N2[1]	90.2(6)	F1_1	C2_1	C1_1	111.04(10)
N1	Cr1	N2	90.2(6)	F2_1	C2_1	C1_1	112.21(10)
N1	Cr1	N2[3]	90.2(6)	F2_1	C2_1	F1_1	107.43(10)
C2[1]	Cr1	C1	89.88(11)	F3_1	C2_1	C1_1	111.01(10)
C2	Cr1	C1	89.88(11)	F3_1	C2_1	F1_1	107.25(10)
C2[2]	Cr1	C1	89.88(11)	F3_1	C2_1	F2_1	107.68(10)
C2[3]	Cr1	C1	89.88(11)	F4_1	C3_1	C1_1	110.74(10)
C2[3]	Cr1	C2	90.00(4)	F5_1	C3_1	C1_1	111.21(9)
C2[2]	Cr1	C2	90.00(4)	F5_1	C3_1	F4_1	107.30(10)
C2[3]	Cr1	C2[1]	90.00(4)	F6_1	C3_1	C1_1	112.54(10)
C2[3]	Cr1	C2[2]	179.8(2)	F6_1	C3_1	F4_1	107.35(10)
C2[2]	Cr1	C2[1]	90.00(4)	F6_1	C3_1	F5_1	107.46(10)
C2	Cr1	C2[1]	179.8(2)	F7_1	C4_1	C1_1	110.80(10)
C3	Cr1	C1	180.0	F8_1	C4_1	C1_1	112.62(10)
C3	Cr1	C2[2]	90.12(11)	F8_1	C4_1	F7_1	107.45(10)
C3	Cr1	C2[1]	90.12(11)	F9_1	C4_1	C1_1	110.25(10)
C3	Cr1	C2	90.12(11)	F9_1	C4_1	F7_1	108.00(10)
C3	Cr1	C2[3]	90.12(11)	F9_1	C4_1	F8_1	107.54(10)

[1]1/2-X,1/2-Y,+Z; [2]1/2-Y,+X,+Z; [3]+Y,1/2-X,+Z; [4]1/2-X,3/2-Y,+Z; [5]1-Y,1/2+X,-Z; [6]-1/2+Y,1-X,-Z

Atomic Occupancy for p4n_a_NO_disorder.

**Atom**	***Occupancy***	**Atom**	***Occupancy***	**Atom**	***Occupancy***
N3	0.160000	O6	0.160000	N2	0.170000
O5	0.170000	N1	0.160000	O4	0.160000
C1	0.840000	O1	0.840000	C2	0.830000
O2	0.830000	C3	0.840000	O3	0.840000

Crystal structure of 4, the symmetry-generated atom sites are greyed out. Note that the nitrogen position cannot be crystallographically unambiguously assigned.

Supplementary Figure 71 shows the crystal structure of **4**, Supplementary Data 6 gives full information in crystallographic data.

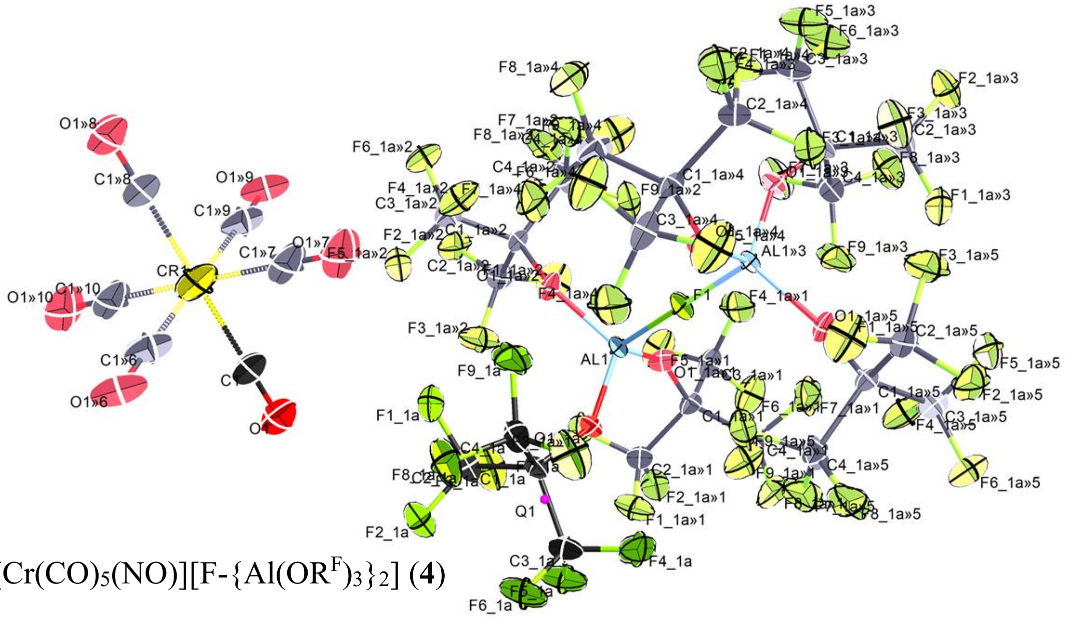

[Cr(CO)$_5$(NO)][F-{Al(OR^F)$_3$}$_2$] (**4**)

Supplementary Figure 71. Crystal structure of **4**.

Crystal data and structure refinement for pa-3_a_final.

Identification code	pa-3_a_final
Empirical formula	$C_{30}Al_2CrF_{55}O_{12}$
Formula weight	1703.26
Temperature/K	100(2)
Crystal system	cubic
Space group	Pa-3
a/Å	17.2474(3)
b/Å	17.2474(3)
c/Å	17.2474(3)
α/°	90
β/°	90
γ/°	90
Volume/Å^3	5130.6(3)
Z	4
ρ_{calc}g/cm^3	2.205
μ/mm^{-1}	0.515
F(000)	3284.0
Crystal size/mm^3	0.4 × 0.3 × 0.2
Radiation	MoKα (λ = 0.71073)
2Θ range for data collection/°	4.09 to 57.328
Index ranges	-23 ≤ h ≤ 12, -15 ≤ k ≤ 23, -19 ≤ l ≤ 22
Reflections collected	13204
Independent reflections	2197 [R_{int} = 0.0216, R_{sigma} = 0.0152]
Data/restraints/parameters	2197/614/250
Goodness-of-fit on F^2	1.272
Final R indexes [I>=2σ (I)]	R_1 = 0.0603, wR_2 = 0.1043
Final R indexes [all data]	R_1 = 0.0642, wR_2 = 0.1056
Largest diff. peak/hole / e Å^{-3}	0.30/-0.35

Supplementary Data 6. Information on the crystallographic data of **4**.

Fractional Atomic Coordinates (×10^4) and Equivalent Isotropic Displacement Parameters (Å^2×10^3) for pa-3_a_final. U_{eq} is defined as 1/3 of of the trace of the orthogonalised U_{IJ} tensor.

Atom	*x*	*y*	*z*	U(eq)
F1	10000	0	5000	21.6(7)
Al1	9410.8(4)	589.2(4)	5589.2(4)	14.3(2)
O1_2	9093(19)	1282(18)	4973(18)	23.8(13)
C1_2	8492(14)	1621(10)	4596(11)	20.8(8)
C2_2	8117(4)	2256(4)	5114(4)	29.2(14)
F1_2	8000(20)	2011(19)	5836(9)	41.6(16)
F2_2	7449(10)	2480(11)	4812(13)	43(4)
F3_2	8577(3)	2886(3)	5146(4)	38.9(13)
C3_2	8801(4)	1998(4)	3840(4)	30.5(14)
F4_2	9465(7)	2378(13)	3968(14)	43.3(15)
F5_2	8307(4)	2514(4)	3546(4)	45.9(17)
F6_2	8929(8)	1467(6)	3299(7)	44(2)
C4_2	7863(3)	1008(4)	4380(3)	26.4(13)
F7_2	8191(17)	356(8)	4136(16)	41.4(13)
F8_2	7380(4)	1262(4)	3827(4)	33.4(14)
F9_2	7424(3)	832(3)	4994(3)	34.1(11)
O1_1	9132(15)	1341(14)	5009(14)	23.8(13)
C1_1	8559(11)	1703(8)	4608(8)	20.8(8)
C2_1	7853(3)	1864(3)	5138(3)	30.0(11)
F1_1	7430(2)	1221(3)	5229(3)	41.5(10)
F2_1	7377(8)	2406(10)	4876(10)	35(2)
F3_1	8106(15)	2078(15)	5832(7)	41.6(16)
C3_1	8894(3)	2484(3)	4312(3)	32.1(12)
F4_1	9607(5)	2373(10)	4049(11)	43.3(15)
F5_1	8936(3)	3001(2)	4879(3)	42.7(11)
F6_1	8460(4)	2786(3)	3754(3)	45.8(13)
C4_1	8310(3)	1204(3)	3907(3)	32.5(12)
F7_1	8825(7)	1222(5)	3342(6)	46.0(19)
F8_1	7630(3)	1430(4)	3620(3)	43.4(13)
F9_1	8232(13)	466(6)	4133(12)	41.4(13)
O1	5816.3(17)	1504.5(17)	5497.5(19)	63.1(8)
C1	5513(2)	954(2)	5323(2)	50.3(9)
Cr1	5000	0	5000	48.8(4)

Anisotropic Displacement Parameters (Å^2×10^3) for pa-3_a_final. The Anisotropic displacement factor exponent takes the form: $-2\pi^2[h^2a^{*2}U_{11}+2hka^*b^*U_{12}+…]$.

Atom	U_{11}	U_{22}	U_{33}	U_{23}	U_{13}	U_{12}
F1	21.6(7)	21.6(7)	21.6(7)	-6.2(8)	6.2(8)	6.2(8)
Al1	14.3(2)	14.3(2)	14.3(2)	-2.4(3)	2.4(3)	2.4(3)
O1_2	20(2)	19(3)	32(2)	6.4(18)	-5.1(15)	3.2(18)
C1_2	21(3)	20(2)	21.8(13)	0.1(14)	-3.8(15)	4.5(14)
C2_2	26(3)	33(3)	29(3)	-5(3)	-2(3)	6(3)
F1_2	39(6)	61(4)	25.2(9)	-8.4(11)	0.7(15)	12(2)
F2_2	34(5)	41(6)	53(9)	-18(5)	-12(5)	27(4)
F3_2	39(3)	21(2)	57(3)	-9(2)	-12(2)	8.3(19)
C3_2	29(3)	37(3)	26(3)	9(2)	1(2)	6(2)
F4_2	38(3)	49.1(12)	43(4)	21.0(18)	-1(3)	-5(3)
F5_2	49(4)	56(4)	32(4)	23(3)	-2(3)	16(3)
F6_2	44(4)	63(6)	26(3)	-1(4)	10(3)	14(4)
C4_2	21(3)	29(3)	29(3)	-4(2)	-6(2)	6(2)
F7_2	45(2)	23(3)	55.9(13)	-14(3)	-12.1(13)	4(3)
F8_2	33(3)	36(3)	31(3)	-6(2)	-17(2)	6(3)
F9_2	24(2)	38(3)	40(3)	2(2)	-0.3(18)	-7(2)
O1_1	20(2)	19(3)	32(2)	6.4(18)	-5.1(15)	3.2(18)
C1_1	21(3)	20(2)	21.8(13)	0.1(14)	-3.8(15)	4.5(14)
C2_1	25(3)	36(3)	29(3)	0(2)	-2(2)	10(2)
F1_1	21.1(16)	46(2)	58(3)	8(2)	5.8(16)	2.7(17)
F2_1	32(3)	42(4)	31(3)	4(3)	1(2)	16(3)
F3_1	39(6)	61(4)	25.2(9)	-8.4(11)	0.7(15)	12(2)
C3_1	40(3)	27(2)	29(3)	3.5(19)	-7(2)	3(2)
F4_1	38(3)	49.1(12)	43(4)	21.0(18)	-1(3)	-5(3)
F5_1	63(3)	22.6(17)	43(2)	-2.9(15)	-13(2)	-1.5(19)
F6_1	62(3)	40(3)	35(3)	13(2)	-16(2)	9(2)

C4_1	28(3)	31(2)	39(3)	-10(2)	-7(2)	6(2)
F7_1	49(4)	57(4)	32(3)	-15(3)	2(2)	13(3)
F8_1	34(3)	53(3)	43(3)	-17(2)	-17(2)	12(2)
F9_1	45(2)	23(3)	55.9(13)	-14(3)	-12.1(13)	4(3)
O1	52.5(17)	51.4(17)	85(2)	9.7(16)	14.7(16)	-12.7(14)
C1	43(2)	48(2)	61(2)	16.1(18)	13.8(18)	-4.4(17)
Cr1	48.8(4)	48.8(4)	48.8(4)	13.3(3)	13.3(3)	-13.3(3)

Bond Lengths for pa-3_a_final.

Atom	Atom	Length/Å	Atom	Atom	Length/Å
F1	Al1	1.7602(12)	C4_2	F7_2	1.328(13)
F1	Al1[1]	1.7602(12)	C4_2	F9_2	1.337(7)
Al1	O1_2	1.69(3)	C4_2	F8_2	1.338(8)
Al1	O1_2[2]	1.69(3)	O1_1	C1_1	1.358(13)
Al1	O1_2[3]	1.69(3)	C1_1	C4_1	1.545(12)
Al1	O1_1[2]	1.71(3)	C1_1	C2_1	1.549(12)
Al1	O1_1[3]	1.71(3)	C1_1	C3_1	1.551(11)
Al1	O1_1	1.71(3)	C2_1	F2_1	1.324(11)
O1_2	C1_2	1.356(16)	C2_1	F3_1	1.326(12)
C1_2	C3_2	1.551(16)	C2_1	F1_1	1.337(7)
C1_2	C2_2	1.555(15)	C3_1	F4_1	1.324(11)
C1_2	C4_2	1.560(14)	C3_1	F5_1	1.325(6)
C2_2	F2_2	1.323(12)	C3_1	F6_1	1.325(7)
C2_2	F1_2	1.328(13)	C4_1	F7_1	1.319(9)
C2_2	F3_2	1.346(9)	C4_1	F8_1	1.331(7)
C3_2	F6_2	1.327(10)	C4_1	F9_1	1.338(11)
C3_2	F5_2	1.332(8)	O1	C1	1.125(5)
C3_2	F4_2	1.336(13)	C1	Cr1	1.949(4)

[1]2-X,-Y,1-Z; [2]3/2-Z,1-X,1/2+Y; [3]1-Y,-1/2+Z,3/2-X

Bond Angles for pa-3_a_final.

Atom	Atom	Atom	Angle/°	Atom	Atom	Atom	Angle/°
Al1	F1	Al1[1]	180.00(4)	C1_1	O1_1	Al1	148.5(17)
O1_2	Al1	O1_2[2]	114.8(8)	O1_1	C1_1	C4_1	110.2(14)
O1_2	Al1	O1_2[3]	114.8(8)	O1_1	C1_1	C2_1	110.7(14)
O1_2[2]	Al1	O1_2[3]	114.8(8)	C4_1	C1_1	C2_1	110.1(10)
O1_2	Al1	O1_1[2]	117.9(16)	O1_1	C1_1	C3_1	107.2(14)
O1_2[2]	Al1	O1_1[2]	4.5(18)	C4_1	C1_1	C3_1	109.3(10)
O1_2[3]	Al1	O1_1[2]	110.2(17)	C2_1	C1_1	C3_1	109.3(10)
O1_1[2]	Al1	O1_1[3]	113.4(7)	F2_1	C2_1	F3_1	108.4(14)
O1_1[2]	Al1	O1_1	113.4(7)	F2_1	C2_1	F1_1	106.7(8)
O1_1[3]	Al1	O1_1	113.4(7)	F3_1	C2_1	F1_1	107.7(8)
O1_2	Al1	F1	103.4(10)	F2_1	C2_1	C1_1	114.4(12)
O1_2[2]	Al1	F1	103.4(10)	F3_1	C2_1	C1_1	109.0(14)
O1_2[3]	Al1	F1	103.4(10)	F1_1	C2_1	C1_1	110.4(6)
O1_1[2]	Al1	F1	105.2(8)	F4_1	C3_1	F5_1	107.4(8)
O1_1[3]	Al1	F1	105.2(8)	F4_1	C3_1	F6_1	109.4(9)
O1_1	Al1	F1	105.2(8)	F5_1	C3_1	F6_1	107.6(5)
C1_2	O1_2	Al1	149(2)	F4_1	C3_1	C1_1	109.4(11)
O1_2	C1_2	C3_2	108.7(18)	F5_1	C3_1	C1_1	111.2(5)
O1_2	C1_2	C2_2	110.2(18)	F6_1	C3_1	C1_1	111.7(9)
C3_2	C1_2	C2_2	109.3(13)	F7_1	C4_1	F8_1	108.2(7)
O1_2	C1_2	C4_2	110.8(18)	F7_1	C4_1	F9_1	107.7(8)
C3_2	C1_2	C4_2	108.8(13)	F8_1	C4_1	F9_1	107.3(11)
C2_2	C1_2	C4_2	109.0(13)	F7_1	C4_1	C1_1	112.2(7)
F2_2	C2_2	F1_2	109.3(17)	F8_1	C4_1	C1_1	111.9(9)
F2_2	C2_2	F3_2	107.1(11)	F9_1	C4_1	C1_1	109.3(12)
F1_2	C2_2	F3_2	107.9(11)	O1	C1	Cr1	178.8(4)
F2_2	C2_2	C1_2	109.9(15)	C1[4]	Cr1	C1[5]	89.28(16)
F1_2	C2_2	C1_2	112.2(19)	C1[4]	Cr1	C1[6]	90.72(16)
F3_2	C2_2	C1_2	110.2(7)	C1[5]	Cr1	C1[6]	180.00(13)
F6_2	C3_2	F5_2	107.4(9)	C1[4]	Cr1	C1[7]	89.28(16)
F6_2	C3_2	F4_2	108.2(11)	C1[5]	Cr1	C1[7]	89.28(16)
F5_2	C3_2	F4_2	106.5(11)	C1[6]	Cr1	C1[7]	90.72(16)
F6_2	C3_2	C1_2	111.0(8)	C1[4]	Cr1	C1[8]	90.72(16)
F5_2	C3_2	C1_2	112.4(12)	C1[5]	Cr1	C1[8]	90.72(16)
F4_2	C3_2	C1_2	111.2(14)	C1[6]	Cr1	C1[8]	89.28(16)

F7_2	C4_2	F9_2	107.4(10)	C1^{7}	Cr1	C1^{8}	180.0(2)
F7_2	C4_2	F8_2	108.5(15)	C1^{4}	Cr1	C1	180.0
F9_2	C4_2	F8_2	106.7(5)	C1^{5}	Cr1	C1	90.72(16)
F7_2	C4_2	C1_2	110.6(16)	C1^{6}	Cr1	C1	89.28(16)
F9_2	C4_2	C1_2	110.9(6)	C1^{7}	Cr1	C1	90.72(16)
F8_2	C4_2	C1_2	112.4(12)	C1^{8}	Cr1	C1	89.28(16)

^{1}2-X,-Y,1-Z; ^{2}3/2-Z,1-X,1/2+Y; ^{3}1-Y,-1/2+Z,3/2-X; ^{4}1-X,-Y,1-Z; ^{5}+Z,1/2-X,1/2+Y; ^{6}1-Z,-1/2+X,1/2-Y; ^{7}1/2-Y,-1/2+Z,+X; ^{8}1/2+Y,1/2-Z,1-X

Torsion Angles for pa-3_a_final.

A	B	C	D	Angle/°	A	B	C	D	Angle/°
O1_2^{1}	Al1	O1_2	C1_2	-133(3)	O1_1^{1}	Al1	O1_1	C1_1	-136(2)
O1_2^{2}	Al1	O1_2	C1_2	3(6)	O1_1^{2}	Al1	O1_1	C1_1	-5(4)
F1	Al1	O1_2	C1_2	115(4)	F1	Al1	O1_1	C1_1	109(3)
Al1	O1_2	C1_2	C3_2	-153(4)	Al1	O1_1	C1_1	C4_1	-68(3)
Al1	O1_2	C1_2	C2_2	87(4)	Al1	O1_1	C1_1	C2_1	54(3)
Al1	O1_2	C1_2	C4_2	-33(5)	Al1	O1_1	C1_1	C3_1	173(3)
O1_2	C1_2	C2_2	F2_2	-168.1(18)	O1_1	C1_1	C2_1	F2_1	160.7(14)
C3_2	C1_2	C2_2	F2_2	72.5(18)	C4_1	C1_1	C2_1	F2_1	-77.3(14)
C4_2	C1_2	C2_2	F2_2	-46.3(19)	C3_1	C1_1	C2_1	F2_1	42.8(15)
O1_2	C1_2	C2_2	F1_2	-46(2)	O1_1	C1_1	C2_1	F3_1	39.2(16)
C3_2	C1_2	C2_2	F1_2	-165.7(17)	C4_1	C1_1	C2_1	F3_1	161.2(15)
C4_2	C1_2	C2_2	F1_2	76(2)	C3_1	C1_1	C2_1	F3_1	-78.7(16)
O1_2	C1_2	C2_2	F3_2	74.0(16)	O1_1	C1_1	C2_1	F1_1	-78.9(12)
C3_2	C1_2	C2_2	F3_2	-45.4(18)	C4_1	C1_1	C2_1	F1_1	43.1(15)
C4_2	C1_2	C2_2	F3_2	-164.2(11)	C3_1	C1_1	C2_1	F1_1	163.1(9)
O1_2	C1_2	C3_2	F6_2	76.8(17)	O1_1	C1_1	C3_1	F4_1	42.4(14)
C2_2	C1_2	C3_2	F6_2	-162.9(13)	C4_1	C1_1	C3_1	F4_1	-77.0(15)
C4_2	C1_2	C3_2	F6_2	-44(2)	C2_1	C1_1	C3_1	F4_1	162.4(12)
O1_2	C1_2	C3_2	F5_2	-162.9(15)	O1_1	C1_1	C3_1	F5_1	-76.1(12)
C2_2	C1_2	C3_2	F5_2	-42.6(17)	C4_1	C1_1	C3_1	F5_1	164.5(9)
C4_2	C1_2	C3_2	F5_2	76.3(14)	C2_1	C1_1	C3_1	F5_1	44.0(15)
O1_2	C1_2	C3_2	F4_2	-43.7(18)	O1_1	C1_1	C3_1	F6_1	163.6(12)
C2_2	C1_2	C3_2	F4_2	77(2)	C4_1	C1_1	C3_1	F6_1	44.3(13)
C4_2	C1_2	C3_2	F4_2	-164.5(15)	C2_1	C1_1	C3_1	F6_1	-76.3(11)
O1_2	C1_2	C4_2	F7_2	-41(2)	O1_1	C1_1	C4_1	F7_1	-74.5(14)
C3_2	C1_2	C4_2	F7_2	79(2)	C2_1	C1_1	C4_1	F7_1	163.2(10)
C2_2	C1_2	C4_2	F7_2	-162.0(17)	C3_1	C1_1	C4_1	F7_1	43.1(16)
O1_2	C1_2	C4_2	F9_2	78.6(16)	O1_1	C1_1	C4_1	F8_1	163.7(12)
C3_2	C1_2	C4_2	F9_2	-161.9(11)	C2_1	C1_1	C4_1	F8_1	41.4(13)
C2_2	C1_2	C4_2	F9_2	-42.9(19)	C3_1	C1_1	C4_1	F8_1	-78.7(12)
O1_2	C1_2	C4_2	F8_2	-162.0(15)	O1_1	C1_1	C4_1	F9_1	45.0(15)
C3_2	C1_2	C4_2	F8_2	-42.5(17)	C2_1	C1_1	C4_1	F9_1	-77.4(16)
C2_2	C1_2	C4_2	F8_2	76.5(15)	C3_1	C1_1	C4_1	F9_1	162.6(13)

^{1}3/2-Z,1-X,1/2+Y; ^{2}1-Y,-1/2+Z,3/2-X

Atomic Occupancy for pa-3_a_final.

**Atom**	***Occupancy***	**Atom**	***Occupancy***	**Atom**	***Occupancy***
O1_2	0.438(5)	C1_2	0.438(5)	C2_2	0.438(5)
F1_2	0.438(5)	F2_2	0.438(5)	F3_2	0.438(5)
C3_2	0.438(5)	F4_2	0.438(5)	F5_2	0.438(5)
F6_2	0.438(5)	C4_2	0.438(5)	F7_2	0.438(5)
F8_2	0.438(5)	F9_2	0.438(5)	O1_1	0.562(5)
C1_1	0.562(5)	C2_1	0.562(5)	F1_1	0.562(5)
F2_1	0.562(5)	F3_1	0.562(5)	C3_1	0.562(5)
F4_1	0.562(5)	F5_1	0.562(5)	F6_1	0.562(5)
C4_1	0.562(5)	F7_1	0.562(5)	F8_1	0.562(5)
F9_1	0.562(5)				

Modelling of the NO-disorder for $[Cr(CO)_5(NO)][F\text{-}\{Al(OR^F)_3\}_2]$

The inclusion of 1/6 NO led to a change of the R_1 value from 6.03% to 5.99% (Supplementary Figure 72). However, the NO ligands resulted in a slightly tilted octahedron. Supplementary Data 7 gives full information on crystallographic data.

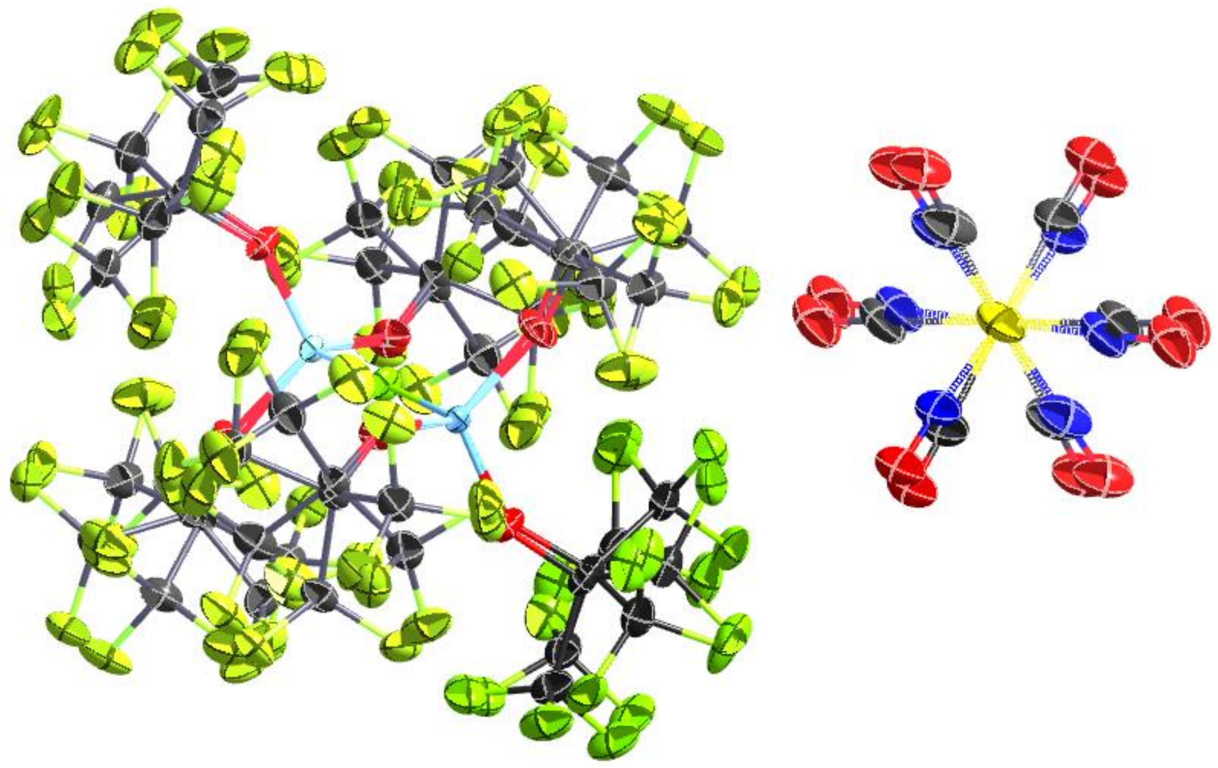

Supplementary Figure 72. Disorder model for **4**.

Supplementary Table 9. Resulting bond lengths from the disorder model for **4**.

w/o disorder [pm]		With NO disorder [pm]			
d(Cr-C1)	194.9(4)	d(Cr-C1)	197.3(14)	d(Cr-N1)	186(7)

The low bond precision for the Cr-N bond (Supplementary Table 9) as well as the tilted structure of the cation underline the question, if the refinement of a disordered NO actually yields a scientifically more accurate structure model. Therefore, we decided to leave the NO data out and just report the average bond lengths in the manuscript.

The disordered crystal structure was also deployed in the CCDC (Accession number: 1886542)

Crystal data and structure refinement for pa-3_a _NO_disorder.

Identification code	pa-3_a_NO_disorder
Empirical formula	$C_{29}NO_{12}F_{55}Al_2Cr$
Formula weight	1705.20
Temperature/K	100.03
Crystal system	cubic
Space group	Pa-3
a/Å	17.2474(3)
b/Å	17.2474(3)
c/Å	17.2474(3)
α/°	90
β/°	90
γ/°	90
Volume/Å^3	5130.63(15)
Z	4
ρ_{calc}g/cm^3	2.2074
μ/mm^{-1}	0.516
F(000)	3294.4
Crystal size/mm^3	0.4 × 0.3 × 0.2
Radiation	Mo Kα (λ = 0.71073)
2Θ range for data collection/°	4.1 to 57.32
Index ranges	-23 ≤ h ≤ 12, -15 ≤ k ≤ 23, -19 ≤ l ≤ 22
Reflections collected	13204
Independent reflections	2197 [R_{int} = 0.0216, R_{sigma} = 0.0152]
Data/restraints/parameters	2197/1/255
Goodness-of-fit on F^2	1.267
Final R indexes [I>=2σ (I)]	R_1 = 0.0599, wR_2 = 0.1028
Final R indexes [all data]	R_1 = 0.0638, wR_2 = 0.1042
Largest diff. peak/hole / e Å^{-3}	0.38/-0.48

Supplementary Data 7. Information on the crystallographic data of disordered **4**.

Fractional Atomic Coordinates (×10^4) and Equivalent Isotropic Displacement Parameters (Å^2×10^3) for pa-3_a_NO_disorder. U_{eq} is defined as 1/3 of of the trace of the orthogonalised U_{IJ} tensor.

Atom	*x*	*y*	*z*	U(eq)
F1	10000	0	5000	21.5(7)
Al1	9410.8(4)	589.2(4)	5589.2(4)	14.2(2)
O1	5807(5)	1523(4)	5474(4)	56.4(13)
C1	5507(8)	979(6)	5307(8)	48.2(16)
O2	5880(30)	1400(30)	5650(30)	56.4(13)
N1	5550(40)	840(30)	5400(40)	48.2(16)
Cr1	5000	0	5000	48.6(4)
O1_2	9120(20)	1236(14)	4950(20)	18.5(19)
C1_2	8500(20)	1620(20)	4518(15)	15.2(19)
C2_2	8117(4)	2256(5)	5114(4)	29.5(16)
F1_2	8010(50)	2020(70)	5830(60)	43(5)
F2_2	7474(15)	2471(13)	4824(11)	45(5)
F3_2	8577(3)	2886(3)	5146(4)	38.3(13)
C3_2	8801(4)	1997(4)	3841(4)	29.9(16)
F4_2	9480(20)	2370(30)	3950(20)	43(3)
F5_2	8308(5)	2512(5)	3545(4)	45.8(17)
F6_2	8928(13)	1466(9)	3295(13)	46(3)
C4_2	7863(4)	1007(4)	4379(4)	26.5(15)
F7_2	8200(40)	350(30)	4150(50)	41(4)
F8_2	7379(4)	1263(4)	3827(4)	32.8(14)
F9_2	7424(3)	831(3)	4993(3)	33.9(11)
O1_1	9111(17)	1375(10)	5027(15)	18.5(19)
C1_1	8555(15)	1699(15)	4665(10)	15.2(19)
C2_1	7854(3)	1866(4)	5139(3)	30.2(13)
F1_1	7430(2)	1220(3)	5228(3)	41.6(10)
F2_1	7359(11)	2414(10)	4868(7)	32(2)
F3_1	8100(40)	2070(50)	5830(40)	43(5)
C3_1	8894(3)	2483(3)	4312(3)	32.9(13)
F4_1	9598(17)	2380(20)	4066(16)	43(3)
F5_1	8936(3)	3001(2)	4879(3)	42.9(11)

F6_1	8459(4)	2786(3)	3754(3)	45.6(13)
C4_1	8311(3)	1206(3)	3906(3)	33.5(14)
F7_1	8827(11)	1225(7)	3345(10)	46(2)
F8_1	7631(3)	1429(4)	3621(4)	43.8(14)
F9_1	8230(30)	470(20)	4120(40)	41(4)

Anisotropic Displacement Parameters (Å^2×10^3) for pa-3_a_NO_disorder. The Anisotropic displacement factor exponent takes the form: $-2\pi^2[h^2a^{*2}U_{11}+2hka^*b^*U_{12}+\ldots]$.

Atom	U_{11}	U_{22}	U_{33}	U_{12}	U_{13}	U_{23}
F1	21.5(7)	21.5(7)	21.5(7)	6.4(8)	6.4(8)	-6.4(8)
Al1	14.2(2)	14.2(2)	14.2(2)	2.4(3)	2.4(3)	-2.4(3)
O1	51(2)	44(3)	75(4)	-10(2)	10(3)	18(2)
C1	44(3)	42(5)	59(4)	-1(3)	12(3)	23(3)
O2	51(2)	44(3)	75(4)	-10(2)	10(3)	18(2)
N1	44(3)	42(5)	59(4)	-1(3)	12(3)	23(3)
Cr1	48.6(4)	48.6(4)	48.6(4)	-13.2(3)	13.2(3)	13.2(3)
O1_2	21.6(9)	6(6)	28(5)	6(5)	-3(3)	-1(4)
C1_2	21(4)	20(5)	4(7)	4(3)	-10(4)	-9(4)
C2_2	25(4)	35(4)	28(3)	5(3)	-1(3)	-4(3)
F1_2	42(12)	62(8)	25.1(9)	14(8)	1(7)	-9(3)
F2_2	27(7)	48(6)	60(8)	24(5)	-6(4)	-26(6)
F3_2	38(3)	21(2)	56(4)	8(2)	-12(2)	-8(2)
C3_2	29(3)	36(4)	25(3)	6(3)	1(3)	8(3)
F4_2	42(8)	49.2(13)	37(8)	-7(5)	-3(4)	20(5)
F5_2	50(4)	55(5)	32(4)	16(3)	-2(3)	22(3)
F6_2	45(6)	67(9)	26(3)	16(6)	11(3)	1(6)
C4_2	21(3)	30(3)	29(3)	8(2)	-6(3)	-6(3)
F7_2	47(8)	20(9)	56(7)	3(6)	-13(5)	-11(7)
F8_2	32(4)	36(3)	31(3)	7(3)	-17(2)	-6(2)
F9_2	24(2)	38(3)	40(3)	-7(2)	0.2(18)	2(2)
O1_1	21.6(9)	6(6)	28(5)	6(5)	-3(3)	-1(4)
C1_1	21(4)	20(5)	4(7)	4(3)	-10(4)	-9(4)
C2_1	25(3)	37(3)	29(3)	11(3)	-2(2)	0(2)
F1_1	20.9(16)	46(3)	58(3)	2.6(17)	5.9(16)	8(2)
F2_1	26(5)	41(3)	30(3)	17(3)	-3(2)	5(3)
F3_1	42(12)	62(8)	25.1(9)	14(8)	1(7)	-9(3)
C3_1	41(3)	29(3)	29(3)	4(2)	-7(2)	3(2)
F4_1	42(8)	49.2(13)	37(8)	-7(5)	-3(4)	20(5)
F5_1	63(3)	22.7(17)	43(2)	-1(2)	-13(2)	-3.1(16)
F6_1	62(3)	39(3)	35(3)	10(2)	-15(2)	13(2)
C4_1	28(3)	31(3)	41(3)	7(2)	-7(2)	-9(2)
F7_1	50(5)	59(6)	31(4)	14(5)	2(3)	-14(5)
F8_1	33(3)	54(4)	44(3)	12(2)	-17(2)	-18(3)
F9_1	47(8)	20(9)	56(7)	3(6)	-13(5)	-11(7)

Bond Lengths for pa-3_a_NO_disorder.

Atom	Atom	Length/Å	Atom	Atom	Length/Å
F1	Al1	1.7602(12)	C3_2	F4_2	1.34(4)
F1	Al1[1]	1.7602(12)	C3_2	F5_2	1.331(10)
Al1	O1_2	1.65(4)	C3_2	F6_2	1.33(2)
Al1	O1_2[2]	1.65(4)	C4_2	F7_2	1.34(7)
Al1	O1_2[3]	1.65(4)	C4_2	F8_2	1.342(9)
Al1	O1_1[2]	1.75(3)	C4_2	F9_2	1.336(8)
Al1	O1_1	1.75(3)	O1_1	C1_1	1.27(4)
Al1	O1_1[3]	1.75(3)	C1_1	C2_1	1.49(3)
O1	C1	1.111(18)	C1_1	C3_1	1.59(3)
C1	Cr1[4]	1.973(14)	C1_1	C4_1	1.617(13)
O2	N1	1.20(9)	C2_1	F1_1	1.341(9)
N1	Cr1[4]	1.86(7)	C2_1	F2_1	1.357(14)
O1_2	C1_2	1.47(5)	C2_1	F3_1	1.32(7)
C1_2	C2_2	1.64(3)	C3_1	F4_1	1.30(4)
C1_2	C3_2	1.43(4)	C3_1	F5_1	1.326(7)
C1_2	C4_2	1.54(3)	C3_1	F6_1	1.328(8)
C2_2	F1_2	1.32(10)	C4_1	F7_1	1.316(17)
C2_2	F2_2	1.27(2)	C4_1	F8_1	1.330(8)
C2_2	F3_2	1.346(11)	C4_1	F9_1	1.33(4)

[1]2-X,-Y,1-Z; [2]1-Y,-1/2+Z,3/2-X; [3]3/2-Z,1-X,1/2+Y; [4]1/2+Y,1/2-Z,1-X

Bond Angles for pa-3_a_NO_disorder.

Atom	Atom	Atom	Angle/°	Atom	Atom	Atom	Angle/°
Al1[1]	F1	Al1	180.0	F1_2	C2_2	C1_2	116(5)
O1_2[2]	Al1	O1_2	116.9(6)	F2_2	C2_2	C1_2	107.2(14)
O1_2[2]	Al1	O1_2[3]	116.9(5)	F2_2	C2_2	F1_2	110(4)
O1_2[3]	Al1	O1_2	116.9(5)	F3_2	C2_2	C1_2	109.1(14)
O1_1	Al1	O1_1[3]	111.5(7)	F3_2	C2_2	F1_2	107(4)
O1_1[2]	Al1	O1_1[3]	111.5(6)	F3_2	C2_2	F2_2	107.2(13)
O1_1[2]	Al1	O1_1	111.5(7)	F4_2	C3_2	C1_2	115.1(19)
C1[4]	Cr1	C1[5]	91.7(5)	F5_2	C3_2	C1_2	112.2(16)
C1[6]	Cr1	C1[5]	91.7(6)	F5_2	C3_2	F4_2	106.5(19)
C1[7]	Cr1	C1[4]	88.3(6)	F6_2	C3_2	C1_2	109.1(14)
C1[7]	Cr1	C1[8]	91.7(6)	F6_2	C3_2	F4_2	106(2)
C1[7]	Cr1	C1	91.7(5)	F6_2	C3_2	F5_2	107.0(12)
C1[7]	Cr1	C1[5]	180.0	F7_2	C4_2	C1_2	109(3)
C1[7]	Cr1	C1[6]	88.3(5)	F8_2	C4_2	C1_2	108.9(14)
C1	Cr1	C1[8]	88.3(5)	F8_2	C4_2	F7_2	110(4)
C1[4]	Cr1	C1	91.7(6)	F9_2	C4_2	C1_2	115.8(12)
C1[5]	Cr1	C1[8]	88.3(6)	F9_2	C4_2	F7_2	107(3)
C1[6]	Cr1	C1	180.0	F9_2	C4_2	F8_2	106.6(5)
C1[5]	Cr1	C1	88.3(5)	C2_1	C1_1	O1_1	115.3(19)
C1[4]	Cr1	C1[8]	180.0	C3_1	C1_1	O1_1	106(2)
C1[4]	Cr1	C1[6]	88.3(6)	C3_1	C1_1	C2_1	110.1(16)
C1[6]	Cr1	C1[8]	91.7(5)	C4_1	C1_1	O1_1	111.2(17)
N1	Cr1	N1[8]	95(2)	C4_1	C1_1	C2_1	109.6(14)
N1[7]	Cr1	N1[8]	85(3)	C4_1	C1_1	C3_1	103.5(12)
N1[4]	Cr1	N1[8]	179.999999146(19)	F1_1	C2_1	C1_1	110.2(10)
N1[4]	Cr1	N1[5]	85(2)	F2_1	C2_1	C1_1	117.2(13)
N1[6]	Cr1	N1[5]	85(3)	F2_1	C2_1	F1_1	106.0(10)
N1[7]	Cr1	N1[6]	95(2)	F3_1	C2_1	C1_1	107(3)
N1[6]	Cr1	N1[8]	85(2)	F3_1	C2_1	F1_1	107(3)
N1[5]	Cr1	N1[8]	95(3)	F3_1	C2_1	F2_1	109(4)
N1[7]	Cr1	N1[5]	180.0	F4_1	C3_1	C1_1	110.4(19)
N1[7]	Cr1	N1	85(2)	F5_1	C3_1	C1_1	108.1(7)
N1[7]	Cr1	N1[4]	95(3)	F5_1	C3_1	F4_1	106.5(15)
N1[5]	Cr1	N1	95(2)	F6_1	C3_1	C1_1	113.8(9)
N1[4]	Cr1	N1[6]	95(3)	F6_1	C3_1	F4_1	110.2(11)
N1[4]	Cr1	N1	85(3)	F6_1	C3_1	F5_1	107.6(5)
N1[6]	Cr1	N1	180.0	F7_1	C4_1	C1_1	113.9(13)
C2_2	C1_2	O1_2	106.2(16)	F8_1	C4_1	C1_1	112.1(11)
C3_2	C1_2	O1_2	110(3)	F8_1	C4_1	F7_1	108.5(10)
C3_2	C1_2	C2_2	111(2)	F9_1	C4_1	C1_1	108(3)
C4_2	C1_2	O1_2	106(3)	F9_1	C4_1	F7_1	108(3)
C4_2	C1_2	C2_2	106(2)	F9_1	C4_1	F8_1	107(3)
C4_2	C1_2	C3_2	116.3(13)				

[1]2-X,-Y,1-Z; [2]3/2-Z,1-X,1/2+Y; [3]1-Y,-1/2+Z,3/2-X; [4]+Z,1/2-X,1/2+Y; [5]1/2+Y,1/2-Z,1-X; [6]1-X,-Y,1-Z; [7]1/2-Y,-1/2+Z,+X; [8]1-Z,-1/2+X,1/2-Y

Atomic Occupancy for pa-3_a_NO_disorder.

Atom	*Occupancy*	Atom	*Occupancy*	Atom	*Occupancy*
F1	1.000020	O1	0.833330	C1	0.833330
O2	0.166670	N1	0.166670	Cr1	1.000020
O1_2	0.436(6)	C1_2	0.436(6)	C2_2	0.436(6)
F1_2	0.436(6)	F2_2	0.436(6)	F3_2	0.436(6)
C3_2	0.436(6)	F4_2	0.436(6)	F5_2	0.436(6)
F6_2	0.436(6)	C4_2	0.436(6)	F7_2	0.436(6)
F8_2	0.436(6)	F9_2	0.436(6)	O1_1	0.564(6)
C1_1	0.564(6)	C2_1	0.564(6)	F1_1	0.564(6)
F2_1	0.564(6)	F3_1	0.564(6)	C3_1	0.564(6)
F4_1	0.564(6)	F5_1	0.564(6)	F6_1	0.564(6)
C4_1	0.564(6)	F7_1	0.564(6)	F8_1	0.564(6)
F9_1	0.564(6)				

15. Hirshfeld Plots

The Hirshfeld plots show that in **1** slight interactions of O1 with the counterion disturb the local symmetry, leading to small distortions and therefore only a tetragonal symmetry and three crystallographically independent CO positions. For **2** however, the even less coordinating [F-{Al(OR^F)$_3$}$_2$] shows hardly any interaction with the [Cr(CO)$_6$]$^{\bullet+}$ cation, therefore an undisturbed D_{3d} local symmetry and only one crystallographically independent CO position (Supplementary Figure 73).

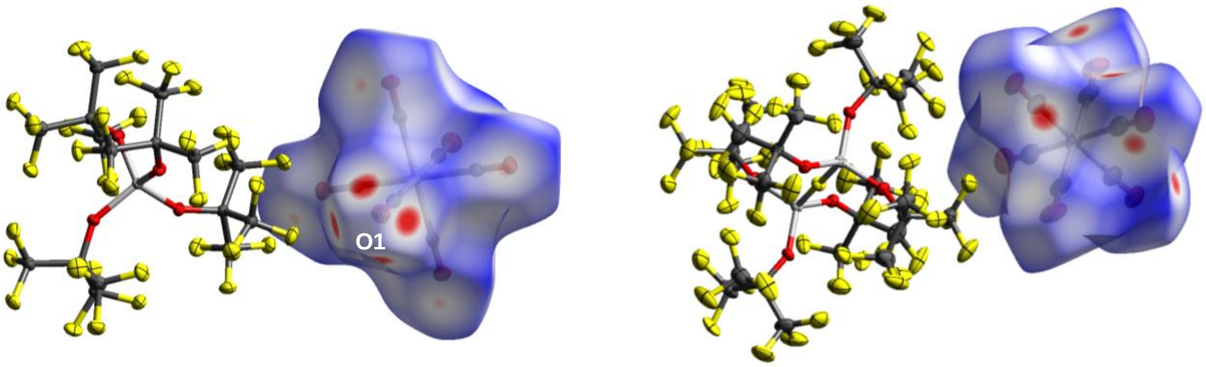

Supplementary Figure 73. Hirshfeld plots of [Cr(CO)$_6$][Al(OR^F)$_4$] (**1**, left) and [Cr(CO)$_6$][F-{Al(OR^F)$_3$}$_2$] (**2**, right). Red faces indicate strong(er) interionic interactions between anion and cation.

Supplementary Figure 74 shows the Hirshfeld plot of the crystal structure of **3** (without a model for the NO-disorder). It can be seen that the interactions between cation and anion is corresponding to the Cr–C1 bond of 195.8 pm bond (other bond lengths: 196.2 (Cr-C2; 4-fold-Symmetry axis), Cr-C3: 192.4 pm), indicating that this is not a favored position for the disordered NO-ligand.

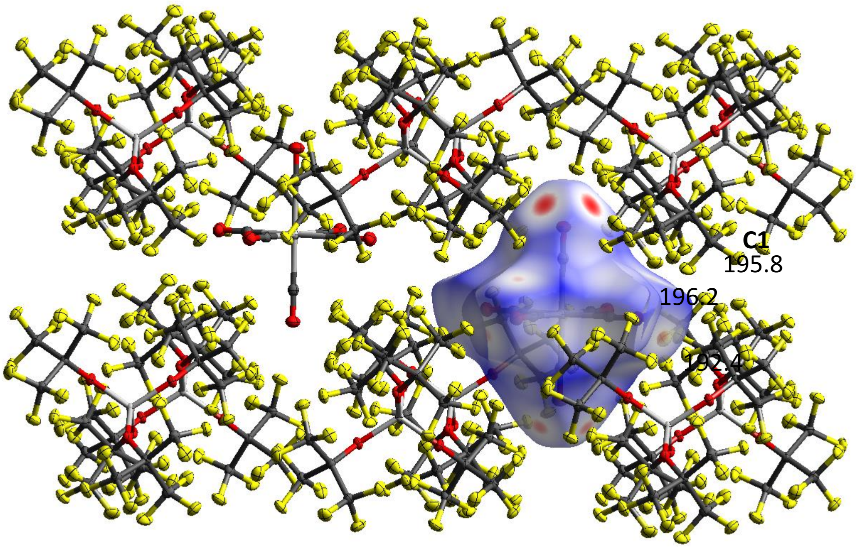

Supplementary Figure 74. Hirshfeld plot of [Cr(CO)$_5$(NO)][Al(OR^F)$_4$] **3** in an enlarged unit cell. Red faces indicate strong(er) interionic interactions between anion and cation. Bond lengths are indicated in pm.

16. Solution Thermodynamics Calculations

Thermodynamics in solution were calculated via Born-Fajans-Haber Cycles (Supplementary Figure 75). Gas phase energetics were calculated with a CCSD(T)/def2-TZVPP[28] - MP2/def2-QZVPPD[28] addition scheme based on B3LYP[51,58]-D3[30](BJ)[29]/def2-TZVPP structures. This compound method approximates CCSD(T)/def2-QZPPPD energies by the following formula (Supplementary Equation 2).

$$E_{\text{comp}} = E_{\text{CCSD(T)/def2-TZVPP}} + E_{\text{MP2/def2-QZVPPD}} - E_{\text{MP2/def2-TZVPP}} \approx E_{\text{CCSD(T)/def2-QZVPPD}} \quad (2)$$

Thermal contributions to reaction energies were calculated with BP86[24,25,59]-D3(BJ)/def-TZVP[60]

$$U° = E_{\text{comp.}} + E_{\text{vrt}} \quad (3)$$

(Where E_{vrt} = sum of translational, rotational, and vibrational energy incl. zero point vibrational energy @BP86-D3(BJ)/def-TZVP).

U° was then corrected to the standard enthalpy *H*° and Gibbs energy *G*° by adding *RT*; cf. RT = 2.48 kJ mol^{-1} @ 298.15 K) as well as subtracting thereof $T \bullet S°$, and in turn generate *H*° and corrected *G*° values.

$$H° = U° + RT \quad (4)$$

$G° = H° - T \bullet S°$ (T = 298.15 K)The RI-C approximation with corresponding auxiliary bases[61] was used to calculate MP2 and CCSD(T) correlation energies, while RI-J auxiliary bases[62–6465] were used to speed up the DFT calculations. The 3s3p orbitals at Cr were taken into the correlation treatment by setting the frozen core cut off to –7.0 H.

COSMO-RS[66,67] solvation thermodynamics were calculated with COSMOthermX[68,69] (Version C30_1501) at the BP86-D3/def2-TZVPD//BP86-D3/def-TZVP level.

Overview (all values Δ*G*° in kJ mol^{-1})

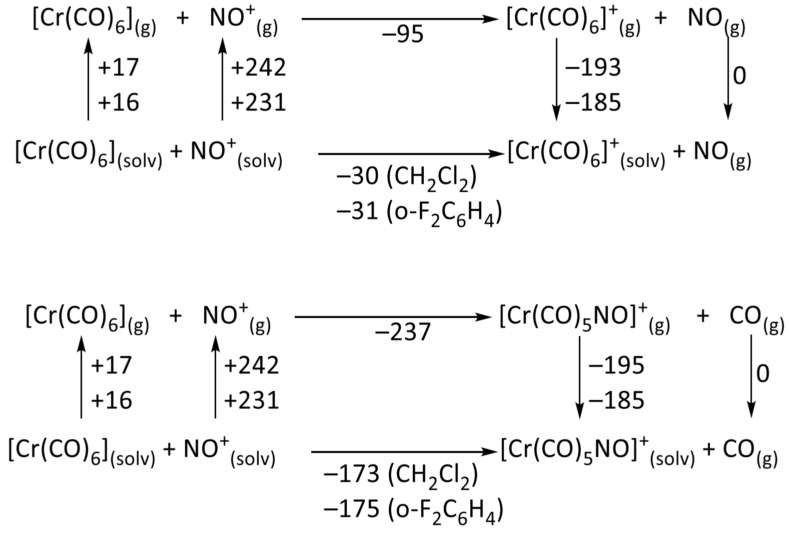

Supplementary Figure 75. Born-Fajans-Haber Cycles for the formations of $[Cr(CO)_6]^+$ and $[Cr(CO)_5(NO)]^+$.

17. Details on the gase phase energetics and DFT calculations

Details on the energetics and DFT calculations are given in Supplementary Table 10, details on the COSMO-RS calculations are given in Supplementary Table 11.

Supplementary Table 10. Details on the energetics of the DFT calculations.

	BP86-D3(bj)/ def-TZVP [H]	Evrt (BP86) [kJ mol^{-1}]	S0(BP86) [kJ mol^{-1}K^{-1}]	B3LYP/ def2-TZVPP [H]	CCSC(T)/ def2-TZVPP [H]	MP2/ def2-TZVPP [H]	MP2/ def2-QZVPPD [H]	Eext [kJ mol^{-1}]	H°(g) [kJ mol^{-1}]	G°(g) [kJ mol^{-1}]	T1diag (CCSD/ def2-TZVPP)
**CO**	-113.360695	18.93	0.19792	-113.311076	-113.158870	-113.138493	-113.1700894	-113.190466	-297160.16	-297219.17	0.0184
**NO**	-129.954339	17.44	*0.20557**	-129.891256	-129.721155	-129.697075	-129.733416	-129.757496	-340658.39	-340719.68	0.0356
**NO^+**	-129.600283	20.37	0.19847	-129.540134	-129.387253	-129.373692	-129.407270	-129.420830	-339771.54	-339830.71	0.0167
**$Cr(CO)_6$**	-1725.129548	167.61	0.47974	-1724.500893	-1722.965619	-1722.980897	-1723.241860	-1723.226582	-4524161.30	-4524304.34	0.0265
**$[Cr(CO)_6]^+$ D_{3d}**	-1724.804481	165.67	*0.51271**	-1724.201660	-1722.664889	-1722.558308	-1722.812970	-1722.919551	-4523357.13	-4523510.00	0.0516
**$[Cr(CO)_6]^+$ D_{4h}**	-1724.801142	168.79	*0.50476**	-1724.199405	-1722.663242	-1722.552409	-1722.807149	-1722.917982	-4523349.89	-4523500.39	0.0528
**$[Cr(CO)_5NO]^+$**	-1741.459256	167.93	0.49858	-1740.823037	-1739.283014	-1739.300498	-1739.562220	-1739.544736	-4567004.29	-4567152.94	0.0301

*RT ln2 added to FREEH entropy to account for the electron spin degeneracy

Details on the COSMO-RS calculations

Supplementary Table 11. Details on the COSMO-RS calculations.

Compound	lgP[kPa]	x°	p(x°) [bar]	ΔsolvG° [kJ mol^{-1}]
o-DFB in o-DFB	0.88148	1.0000	$7.612 \cdot 10^{-2}$	-6.38
NO^+ in o-DFB	-37.52002	0.1010	$3.050 \cdot 10^{-41}$	-231.27
$Cr(CO)_6$ in o-DFB	0.17512	0.1010	$1.512 \cdot 10^{-3}$	-16.10
$Cr(C_6H_6)^+$ in o-DFB	-29.25225	0.1010	$5.650 \cdot 10^{-33}$	-184.07
$Cr(CO)_5NO^+$ in o-DFB	-29.37021	0.1010	$4.306 \cdot 10^{-33}$	-184.75
DCM in DCM	1.82198	1.0000	$6.637 \cdot 10^{-1}$	-1.02
NO^+ in DCM	-39.16843	0.0639	$4.333 \cdot 10^{-43}$	-241.81
$Cr(CO)_6$ in DCM	0.30644	0.0639	$1.293 \cdot 10^{-03}$	-16.49
$Cr(CO)_6^+$ in DCM	-30.64946	0.0639	$1.431 \cdot 10^{-34}$	-193.18
$Cr(CO)_5NO^+$ in DCM	-30.90082	0.0639	$8.024 \cdot 10^{-35}$	-194.62

18. Additional information on the DFT calculations

Supplementary Figure 76 shows the calculated structure of **$[Cr(CO)_6]^{\bullet+}$** (@BP86-D3BJ/def2-TZVPP, D_{3d} Symmetry), Supplementary Data 8 gives full information on the atomic coordinates and the calculated spectra.

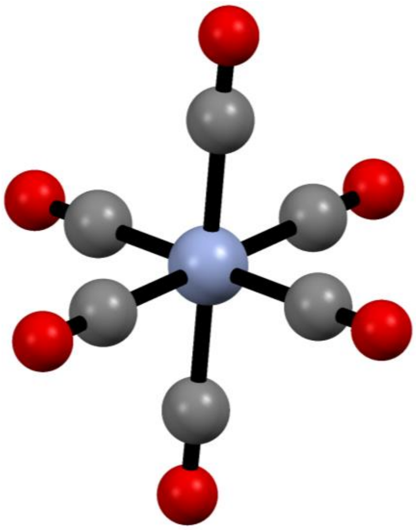

Supplementary Figure 76. Optimized calculated structure of $[Cr(CO)_6]^{\bullet+}$.

Coordinates and vibrational spectrum

Supplementary Data 8. Information on the atomic coordinates and vibrational spectra of $[Cr(CO)_6]^{+}$.

```
$coord
    0.00000000000000      0.00000000000000      0.00000000000000  cr
    2.70377210067779      1.56102355015373     -1.98661068300968  c
    4.29003072522233      2.47685039403888     -3.11633557947232  o
    2.70377210067779     -1.56102355015373      1.98661068300968  c
    4.29003072522233     -2.47685039403888      3.11633557947232  o
   -2.70377210067779     -1.56102355015373      1.98661068300968  c
   -4.29003072522233     -2.47685039403887      3.11633557947232  o
   -2.70377210067779      1.56102355015373     -1.98661068300968  c
   -4.29003072522233      2.47685039403887     -3.11633557947232  o
    0.00000000000000      3.12204710030743      1.98661068300968  c
    0.00000000000000      4.95370078807776      3.11633557947232  o
    0.00000000000000     -3.12204710030743     -1.98661068300968  c
    0.00000000000000     -4.95370078807776     -3.11633557947232  o
```

```
$vibrational spectrum
#  mode     symmetry     wave number   IR intensity    selection rules
#                         cm**(-1)        km/mol         IR      RAMAN
     1                       -0.00        0.00000        -        -
     2                       -0.00        0.00000        -        -
     3                       -0.00        0.00000        -        -
     4                        0.00        0.00000        -        -
     5                        0.00        0.00000        -        -
     6                        0.00        0.00000        -        -
     7         eu            56.17        0.48754       YES      NO
     8         eu            56.17        0.48754       YES      NO
     9         a1u           71.46        0.00000       NO       NO
    10         a1g           81.72        0.00000       NO       YES
    11         eg            83.02        0.00000       NO       YES
    12         eg            83.02        0.00000       NO       YES
    13         a2u           85.86        0.99846       YES      NO
    14         eu            97.42        1.72444       YES      NO
    15         eu            97.42        1.72444       YES      NO
    16         eg           327.40        0.00000       NO       YES
```

```
   17        eg           327.40         0.00000       NO      YES
   18        eg           336.79         0.00000       NO      YES
   19        eg           336.79         0.00000       NO      YES
   20        a2g          342.08         0.00000       NO      NO
   21        a1g          363.50         0.00000       NO      YES
   22        a2u          390.93         5.80762       YES     NO
   23        eu           392.17        15.72340       YES     NO
   24        eu           392.17        15.72340       YES     NO
   25        eu           451.05         1.11300       YES     NO
   26        eu           451.05         1.11300       YES     NO
   27        a1g          459.87         0.00000       NO      YES
   28        eg           483.72         0.00000       NO      YES
   29        eg           483.72         0.00000       NO      YES
   30        a1u          509.43         0.00000       NO      NO
   31        a2u          531.29        82.99160       YES     NO
   32        eu           624.15       119.64769       YES     NO
   33        eu           624.15       119.64769       YES     NO
   34        eg          2073.61         0.00000       NO      YES
   35        eg          2073.61         0.00000       NO      YES
   36        eu          2080.43       975.48463       YES     NO
   37        eu          2080.43       975.48463       YES     NO
   38        a2u         2083.38       786.33708       YES     NO
   39        a1g         2158.03         0.00000       NO      YES
```

$raman spectrum

```
# mode symmetry wave  selection  derivative of  derivative of     raman
#             number   rule       isotropic   polarizability   scattering
#                               polarizability   anisotropy   cross sections
#             cm**(-1)                a.u.          a.u.        bohr**2/sr
#                                                                T,T        II,II
    1             0.00    -         0.000000     0.000000 0.00000D+00 0.00000D+00
    2             0.00    -         0.000000     0.000000 0.00000D+00 0.00000D+00
    3             0.00    -         0.000000     0.000000 0.00000D+00 0.00000D+00
    4             0.00    -         0.000000     0.000000 0.00000D+00 0.00000D+00
    5             0.00    -         0.000000     0.000000 0.00000D+00 0.00000D+00
    6             0.00    -         0.000000     0.000000 0.00000D+00 0.00000D+00
    7    eu      56.17   NO         0.000000     0.000000 0.00000D+00 0.00000D+00
    8    eu      56.17   NO         0.000000     0.000000 0.00000D+00 0.00000D+00
    9    a1u     71.46   NO         0.000000     0.000000 0.00000D+00 0.00000D+00
   10    a1g     81.72   YES        0.000484     0.089709 0.27152D-13 0.20358D-13
   11    eg      83.02   YES        0.000000     0.097543 0.31168D-13 0.23376D-13
   12    eg      83.02   YES        0.000000     0.097543 0.31168D-13 0.23376D-13
   13    a2u     85.86   NO         0.000000     0.000000 0.00000D+00 0.00000D+00
   14    eu      97.42   NO         0.000000     0.000000 0.00000D+00 0.00000D+00
   15    eu      97.42   NO         0.000000     0.000000 0.00000D+00 0.00000D+00
   16    eg     327.40   YES        0.000000     0.007290 0.16502D-16 0.12376D-16
   17    eg     327.40   YES        0.000000     0.007290 0.16502D-16 0.12376D-16
   18    eg     336.79   YES        0.000000     0.017925 0.95487D-16 0.71615D-16
   19    eg     336.79   YES        0.000000     0.017925 0.95487D-16 0.71615D-16
   20    a2g    342.08   NO         0.000000     0.000000 0.93991D-33 0.70493D-33
   21    a1g    363.50   YES        0.067516     0.015615 0.13617D-13 0.48331D-16
   22    a2u    390.93   NO         0.000000     0.000000 0.00000D+00 0.00000D+00
   23    eu     392.17   NO         0.000000     0.000000 0.00000D+00 0.00000D+00
   24    eu     392.17   NO         0.000000     0.000000 0.00000D+00 0.00000D+00
   25    eu     451.05   NO         0.000000     0.000000 0.00000D+00 0.00000D+00
   26    eu     451.05   NO         0.000000     0.000000 0.00000D+00 0.00000D+00
   27    a1g    459.87   YES       -0.006331     0.010234 0.10317D-15 0.14586D-16
   28    eg     483.72   YES       -0.000000     0.026452 0.12060D-15 0.90450D-16
   29    eg     483.72   YES        0.000000     0.026452 0.12060D-15 0.90450D-16
   30    a1u    509.43   NO         0.000000     0.000000 0.00000D+00 0.00000D+00
   31    a2u    531.29   NO         0.000000     0.000000 0.00000D+00 0.00000D+00
   32    eu     624.15   NO         0.000000     0.000000 0.00000D+00 0.00000D+00
   33    eu     624.15   NO         0.000000     0.000000 0.00000D+00 0.00000D+00
   34    eg    2073.61   YES        0.000000     0.647459 0.69382D-14 0.52036D-14
   35    eg    2073.61   YES        0.000000     0.647459 0.69382D-14 0.52036D-14
   36    eu    2080.43   NO         0.000000     0.000000 0.00000D+00 0.00000D+00
   37    eu    2080.43   NO         0.000000     0.000000 0.00000D+00 0.00000D+00
   38    a2u   2083.38   NO         0.000000     0.000000 0.00000D+00 0.00000D+00
   39    a1g   2158.03   YES        0.185543     0.033106 0.58968D-14 0.12480D-16
```

Supplementary Figure 77 shows the calculated structure of **$[Cr(CO)_5(NO)]^+$** (@BP86-D3BJ/def2-TZVPP, C_{4v} Symmetry), Supplementary Data 9 gives full information on the atomic coordinates and the calculated spectra.

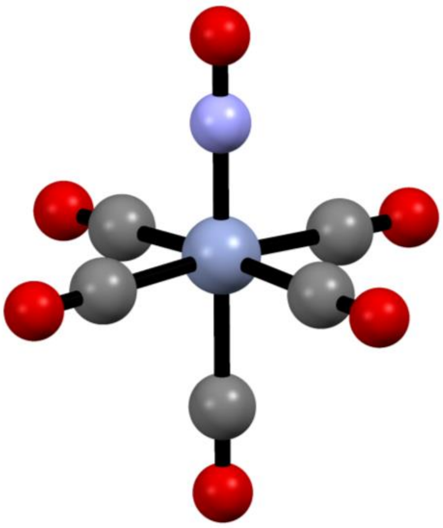

Supplementary Figure 77. Optimized calculated structure of $[Cr(CO)_5(NO)]^+$.

Coordinates and vibrational spectrum

Supplementary Data 9. Information on the atomic coordinates and vibrational spectra of $[Cr(CO)_5(NO)]^+$.

```
$coord
    0.00000000000000     -0.00000000000000      0.20282980067283   cr
    0.00000000000000      0.00000000000000      3.49466707406178   n
    0.00000000000000      0.00000000000000     -3.56906595476235   c
    0.00000000000000      3.67489222252525      0.04366159906058   c
    0.00000000000000     -3.67489222252525      0.04366159906058   c
   -3.67489222252525      0.00000000000000      0.04366159906058   c
    3.67489222252525      0.00000000000000      0.04366159906058   c
    0.00000000000000      0.00000000000000      5.65792323415895   o
   -5.82666319957331     -0.00000000000000     -0.02605702713823   o
    0.00000000000000      5.82666319957331     -0.02605702713823   o
   -0.00000000000000     -5.82666319957331     -0.02605702713823   o
    0.00000000000000      0.00000000000000     -5.71360688969806   o
    5.82666319957331     -0.00000000000000     -0.02605702713823   o


$vibrational spectrum
#  mode     symmetry     wave number   IR intensity    selection rules
#                         cm**(-1)        km/mol         IR     RAMAN
     1                      -0.00         0.00000        -       -
     2                       0.00         0.00000        -       -
     3                       0.00         0.00000        -       -
     4                       0.00         0.00000        -       -
     5                       0.00         0.00000        -       -
     6                       0.00         0.00000        -       -
     7        e             58.57         0.00136       YES     YES
     8        e             58.57         0.00136       YES     YES
     9        b1            69.45         0.00000       NO      YES
    10        b2            87.09         0.00000       NO      YES
    11        e             90.05         0.09603       YES     YES
    12        e             90.05         0.09603       YES     YES
    13        e            100.33         1.03821       YES     YES
    14        e            100.33         1.03821       YES     YES
    15        a1           101.15         2.22404       YES     YES
    16        e            339.48         0.09897       YES     YES
    17        e            339.48         0.09897       YES     YES
    18        a2           346.10         0.00000       NO      NO
    19        a1           353.98        10.43425       YES     YES
    20        b1           369.48         0.00000       NO      YES
    21        a1           372.81         0.34551       YES     YES
```

22	e	418.09	23.07694	YES	YES
23	e	418.09	23.07694	YES	YES
24	e	471.87	0.14618	YES	YES
25	e	471.87	0.14618	YES	YES
26	b1	477.89	0.00000	NO	YES
27	b2	504.72	0.00000	NO	YES
28	a1	505.67	2.58879	YES	YES
29	e	534.68	25.72743	YES	YES
30	e	534.68	25.72743	YES	YES
31	a1	666.84	128.55458	YES	YES
32	e	696.49	91.76903	YES	YES
33	e	696.49	91.76903	YES	YES
34	a1	1902.64	1039.11465	YES	YES
35	e	2084.71	1027.37403	YES	YES
36	e	2084.71	1027.37403	YES	YES
37	b1	2097.73	0.00000	NO	YES
38	a1	2123.66	218.20731	YES	YES
39	a1	2162.05	86.63598	YES	YES

$raman spectrum

# mode	symmetry	wave number cm**(-1)	selection rule	derivative of isotropic polarizability a.u.	derivative of polarizability anisotropy a.u.	raman scattering cross sections bohr**2/sr T,T	II,II
1		0.00	-	0.000000	0.000000	0.00000D+00	0.00000D+00
2		0.00	-	0.000000	0.000000	0.00000D+00	0.00000D+00
3		0.00	-	0.000000	0.000000	0.00000D+00	0.00000D+00
4		0.00	-	0.000000	0.000000	0.00000D+00	0.00000D+00
5		0.00	-	0.000000	0.000000	0.00000D+00	0.00000D+00
6		0.00	-	0.000000	0.000000	0.00000D+00	0.00000D+00
7	e	58.57	YES	0.000000	0.001355	0.11556D-16	0.86672D-17
8	e	58.57	YES	0.000000	0.001355	0.11556D-16	0.86672D-17
9	b1	69.45	YES	0.000000	0.005316	0.12903D-15	0.96771D-16
10	b2	87.09	YES	0.000000	0.097362	0.28428D-13	0.21321D-13
11	e	90.05	YES	0.000000	0.094990	0.25446D-13	0.19084D-13
12	e	90.05	YES	0.000000	0.094990	0.25446D-13	0.19084D-13
13	e	100.33	YES	0.000000	0.040387	0.37746D-14	0.28310D-14
14	e	100.33	YES	0.000000	0.040387	0.37746D-14	0.28310D-14
15	a1	101.15	YES	-0.001832	0.016246	0.68789D-15	0.45136D-15
16	e	339.48	YES	0.000000	0.002243	0.14774D-17	0.11081D-17
17	e	339.48	YES	0.000000	0.002243	0.14774D-17	0.11081D-17
18	a2	346.10	NO	0.000000	0.000000	0.00000D+00	0.00000D+00
19	a1	353.98	YES	-0.019387	0.020927	0.12843D-14	0.90407D-16
20	b1	369.48	YES	0.000000	0.044185	0.50325D-15	0.37744D-15
21	a1	372.81	YES	-0.057923	0.040255	0.10010D-13	0.30903D-15
22	e	418.09	YES	0.000000	0.014174	0.42973D-16	0.32230D-16
23	e	418.09	YES	0.000000	0.014174	0.42973D-16	0.32230D-16
24	e	471.87	YES	0.000000	0.003611	0.23311D-17	0.17483D-17
25	e	471.87	YES	0.000000	0.003611	0.23311D-17	0.17483D-17
26	b1	477.89	YES	0.000000	0.001595	0.44624D-18	0.33468D-18
27	b2	504.72	YES	0.000000	0.007279	0.85822D-17	0.64366D-17
28	a1	505.67	YES	-0.043698	0.073286	0.43371D-14	0.65061D-15
29	e	534.68	YES	0.000000	0.001108	0.18282D-18	0.13711D-18
30	e	534.68	YES	0.000000	0.001108	0.18282D-18	0.13711D-18
31	a1	666.84	YES	-0.041065	0.103367	0.32103D-14	0.86748D-15
32	e	696.49	YES	0.000000	0.018140	0.33459D-16	0.25094D-16
33	e	696.49	YES	0.000000	0.018140	0.33459D-16	0.25094D-16
34	a1	1902.64	YES	0.037570	0.175169	0.92119D-15	0.45527D-15
35	e	2084.71	YES	0.000000	0.069320	0.78629D-16	0.58972D-16
36	e	2084.71	YES	0.000000	0.069320	0.78629D-16	0.58972D-16
37	b1	2097.73	YES	0.000000	0.589111	0.56035D-14	0.42026D-14
38	a1	2123.66	YES	0.001080	0.474507	0.35405D-14	0.26552D-14
39	a1	2162.05	YES	0.171112	0.000886	0.49807D-14	0.88931D-20

Gaussian Calculations

Supplementary Data 10 gives full information on the Gaussian calculations of **$[Cr(CO)_6]^{\bullet+}$**.

Supplementary Data 10. Information on the atomic coordinates and vibrational spectra of $[Cr(CO)_6]^{+}$.

```
Harmonic frequencies (cm**-1), IR intensities (KM/Mole), Raman scattering
 activities (A**4/AMU), depolarization ratios for plane and unpolarized
 incident light, reduced masses (AMU), force constants (mDyne/A),
 and normal coordinates:
                     1                      2                      3
                    EU                     EU                     A1U
 Frequencies --    59.1444                59.1444                73.6203
 Red. masses --    15.2953                15.2953                15.4222
 Frc consts  --     0.0315                 0.0315                 0.0492
 IR Inten    --     0.4145                 0.4145                 0.0000
 Raman Activ --     0.0000                 0.0000                 0.0000
 Depolar (P) --     0.0000                 0.0000                 0.0000
 Depolar (U) --     0.0000                 0.0000                 0.0000
 Atom AN      X      Y      Z        X      Y      Z        X      Y      Z
   1  24     0.00   0.00   0.00     0.00   0.00   0.00     0.00   0.00   0.00
   2   6    -0.11   0.00   0.00     0.00  -0.11   0.19     0.15   0.00   0.00
   3   8    -0.24   0.00   0.00     0.00  -0.24   0.40     0.38   0.00   0.00
   4   6    -0.11   0.00   0.00     0.00  -0.11   0.19     0.15   0.00   0.00
   5   8    -0.24   0.00   0.00     0.00  -0.24   0.40     0.38   0.00   0.00
   6   6     0.05   0.09   0.16    -0.09   0.05  -0.09    -0.08   0.13   0.00
   7   8     0.12   0.21   0.35    -0.21   0.12  -0.20    -0.19   0.33   0.00
   8   6     0.05   0.09   0.16    -0.09   0.05  -0.09    -0.08   0.13   0.00
   9   8     0.12   0.21   0.35    -0.21   0.12  -0.20    -0.19   0.33   0.00
  10   6     0.05  -0.09  -0.16     0.09   0.05  -0.09    -0.08  -0.13   0.00
  11   8     0.12  -0.21  -0.35     0.21   0.12  -0.20    -0.19  -0.33   0.00
  12   6     0.05  -0.09  -0.16     0.09   0.05  -0.09    -0.08  -0.13   0.00
  13   8     0.12  -0.21  -0.35     0.21   0.12  -0.20    -0.19  -0.33   0.00
                     4                      5                      6
                    A1G                    EG                     EG
 Frequencies --    84.0735                85.4517                85.4517
 Red. masses --    15.5007                15.4985                15.4985
 Frc consts  --     0.0646                 0.0667                 0.0667
 IR Inten    --     0.0000                 0.0000                 0.0000
 Raman Activ --     8.5938                10.0021                10.0021
 Depolar (P) --     0.7499                 0.7500                 0.7500
 Depolar (U) --     0.8571                 0.8571                 0.8571
 Atom AN      X      Y      Z        X      Y      Z        X      Y      Z
   1  24     0.00   0.00   0.00     0.00   0.00   0.00     0.00   0.00   0.00
   2   6     0.00   0.08  -0.12    -0.15  -0.02   0.05    -0.10   0.03  -0.07
   3   8     0.00   0.20  -0.32    -0.39  -0.07   0.13    -0.27   0.10  -0.18
   4   6     0.00  -0.08   0.12     0.15   0.02  -0.05     0.10  -0.03   0.07
   5   8     0.00  -0.20   0.32     0.39   0.07  -0.13     0.27  -0.10   0.18
   6   6    -0.07  -0.04  -0.12    -0.07   0.15   0.04     0.07  -0.05   0.08
   7   8    -0.18  -0.10  -0.32    -0.17   0.40   0.09     0.20  -0.12   0.20
   8   6     0.07   0.04   0.12     0.07  -0.15  -0.04    -0.07   0.05  -0.08
   9   8     0.18   0.10   0.32     0.17  -0.40  -0.09    -0.20   0.12  -0.20
  10   6     0.07  -0.04  -0.12     0.04  -0.01  -0.09    -0.09  -0.16  -0.01
  11   8     0.18  -0.10  -0.32     0.13  -0.03  -0.22    -0.23  -0.42  -0.02
  12   6    -0.07   0.04   0.12    -0.04   0.01   0.09     0.09   0.16   0.01
  13   8    -0.18   0.10   0.32    -0.13   0.03   0.22     0.23   0.42   0.02
                     7                      8                      9
                    A2U                    EU                     EU
 Frequencies --    90.7141               102.6922               102.6933
 Red. masses --    17.8330                17.1262                17.1262
 Frc consts  --     0.0865                 0.1064                 0.1064
 IR Inten    --     0.8081                 1.4800                 1.4800
 Raman Activ --     0.0000                 0.0000                 0.0000
 Depolar (P) --     0.0000                 0.0000                 0.0000
 Depolar (U) --     0.0000                 0.0000                 0.0000
 Atom AN      X      Y      Z        X      Y      Z        X      Y      Z
   1  24     0.00   0.00   0.26     0.22   0.00   0.00     0.00   0.22   0.00
   2   6     0.00   0.14   0.07    -0.02   0.00   0.00     0.00   0.18   0.12
   3   8     0.00   0.30  -0.20    -0.43   0.00   0.00     0.00   0.07   0.29
   4   6     0.00   0.14   0.07    -0.02   0.00   0.00     0.00   0.18   0.12
   5   8     0.00   0.30  -0.20    -0.43   0.00   0.00     0.00   0.07   0.29
   6   6    -0.12  -0.07   0.07     0.13   0.08  -0.11     0.08   0.03  -0.06
   7   8    -0.26  -0.15  -0.20    -0.05   0.22  -0.25     0.22  -0.30  -0.14
   8   6    -0.12  -0.07   0.07     0.13   0.08  -0.11     0.08   0.03  -0.06
   9   8    -0.26  -0.15  -0.20    -0.05   0.22  -0.25     0.22  -0.30  -0.14
  10   6     0.12  -0.07   0.07     0.13  -0.08   0.11    -0.08   0.03  -0.06
  11   8     0.26  -0.15  -0.20    -0.05  -0.22   0.25    -0.22  -0.30  -0.14
```

```
 12   6     0.12  -0.07   0.07     0.13  -0.08   0.11    -0.08   0.03  -0.06
 13   8     0.26  -0.15  -0.20    -0.05  -0.22   0.25    -0.22  -0.30  -0.14
                    10                     11                     12
                    EG                     EG                     EG
Frequencies --   323.5861               323.5861               331.5603
Red. masses --    12.9494                12.9494                13.7648
Frc consts  --     0.7989                 0.7989                 0.8915
IR Inten    --     0.0000                 0.0000                 0.0000
Raman Activ --     0.0268                 0.0268                 0.1953
Depolar (P) --     0.7500                 0.7500                 0.7500
Depolar (U) --     0.8571                 0.8571                 0.8571
Atom AN      X      Y      Z        X      Y      Z        X      Y      Z
  1  24     0.00   0.00   0.00     0.00   0.00   0.00     0.00   0.00   0.00
  2   6     0.19   0.01  -0.04     0.02  -0.09   0.46     0.14  -0.04   0.00
  3   8    -0.09  -0.02   0.01    -0.01   0.25  -0.08    -0.08  -0.03  -0.03
  4   6    -0.19  -0.01   0.04    -0.02   0.09  -0.46    -0.14   0.04   0.00
  5   8     0.09   0.02  -0.01     0.01  -0.25   0.08     0.08   0.03   0.03
  6   6     0.11  -0.03   0.42     0.05  -0.17  -0.19     0.33   0.09  -0.02
  7   8    -0.22  -0.08  -0.08     0.05   0.12   0.03     0.16   0.14  -0.22
  8   6    -0.11   0.03  -0.42    -0.05   0.17   0.19    -0.33  -0.09   0.02
  9   8     0.22   0.08   0.08    -0.05  -0.12  -0.03    -0.16  -0.14   0.22
 10   6     0.12   0.06  -0.37    -0.03  -0.16  -0.27     0.35  -0.14   0.02
 11   8    -0.20   0.06   0.07    -0.09   0.14   0.05     0.19  -0.15   0.25
 12   6    -0.12  -0.06   0.37     0.03   0.16   0.27    -0.35   0.14  -0.02
 13   8     0.20  -0.06  -0.07     0.09  -0.14  -0.05    -0.19   0.15  -0.25
                    13                     14                     15
                    EG                     A2G                    A1G
Frequencies --   331.5603               338.3605               356.7004
Red. masses --    13.7648                12.7318                14.0974
Frc consts  --     0.8915                 0.8588                 1.0568
IR Inten    --     0.0000                 0.0000                 0.0000
Raman Activ --     0.1960                 0.0000                24.8498
Depolar (P) --     0.7500                 0.7500                 0.0030
Depolar (U) --     0.8571                 0.8571                 0.0060
Atom AN      X      Y      Z        X      Y      Z        X      Y      Z
  1  24     0.00   0.00   0.00     0.00   0.00   0.00     0.00   0.00   0.00
  2   6     0.01   0.40   0.03    -0.37   0.00   0.00     0.00   0.25   0.13
  3   8    -0.01   0.26   0.27     0.17   0.00   0.00     0.00   0.25   0.16
  4   6    -0.01  -0.40  -0.03     0.37   0.00   0.00     0.00  -0.25  -0.13
  5   8     0.01  -0.26  -0.27    -0.17   0.00   0.00     0.00  -0.25  -0.16
  6   6     0.15   0.22  -0.02     0.18  -0.32   0.00    -0.22  -0.12   0.13
  7   8     0.16   0.02  -0.16    -0.09   0.15   0.00    -0.21  -0.12   0.16
  8   6    -0.15  -0.22   0.02    -0.18   0.32   0.00     0.22   0.12  -0.13
  9   8    -0.16  -0.02   0.16     0.09  -0.15   0.00     0.21   0.12  -0.16
 10   6    -0.08   0.19  -0.01     0.18   0.32   0.00     0.22  -0.12   0.13
 11   8    -0.13  -0.01  -0.11    -0.09  -0.15   0.00     0.21  -0.12   0.16
 12   6     0.08  -0.19   0.01    -0.18  -0.32   0.00    -0.22   0.12  -0.13
 13   8     0.13   0.01   0.11     0.09   0.15   0.00    -0.21   0.12  -0.16
                    16                     17                     18
                    A2U                    EU                     EU
Frequencies --   388.6730               391.1545               391.1572
Red. masses --    14.8602                14.9905                14.9903
Frc consts  --     1.3226                 1.3513                 1.3513
IR Inten    --     5.5761                14.7496                14.7482
Raman Activ --     0.0000                 0.0000                 0.0000
Depolar (P) --     0.0000                 0.0000                 0.0000
Depolar (U) --     0.0000                 0.0000                 0.0000
Atom AN      X      Y      Z        X      Y      Z        X      Y      Z
  1  24     0.00   0.00   0.20     0.00   0.22   0.00     0.22   0.00   0.00
  2   6     0.00  -0.31   0.11     0.00  -0.02  -0.45     0.15   0.00   0.00
  3   8     0.00  -0.13  -0.19     0.00  -0.29  -0.02    -0.05   0.00   0.00
  4   6     0.00  -0.31   0.11     0.00  -0.02  -0.45     0.15   0.00   0.00
  5   8     0.00  -0.13  -0.19     0.00  -0.29  -0.02    -0.05   0.00   0.00
  6   6     0.27   0.15   0.11    -0.07   0.11   0.23     0.03  -0.07   0.39
  7   8     0.11   0.06  -0.19    -0.10  -0.11   0.01    -0.23  -0.10   0.02
  8   6     0.27   0.15   0.11    -0.07   0.11   0.23     0.03  -0.07   0.39
  9   8     0.11   0.06  -0.19    -0.10  -0.11   0.01    -0.23  -0.10   0.02
 10   6    -0.27   0.15   0.11     0.07   0.11   0.23     0.03   0.07  -0.39
 11   8    -0.11   0.06  -0.19     0.10  -0.11   0.01    -0.23   0.10  -0.02
 12   6    -0.27   0.15   0.11     0.07   0.11   0.23     0.03   0.07  -0.39
 13   8    -0.11   0.06  -0.19     0.10  -0.11   0.01    -0.23   0.10  -0.02
                    19                     20                     21
                    EU                     EU                     A1G
Frequencies --   448.7896               448.7901               458.5015
Red. masses --    13.3852                13.3853                12.2969
Frc consts  --     1.5884                 1.5884                 1.5231
IR Inten    --     1.0332                 1.0336                 0.0000
Raman Activ --     0.0000                 0.0000                 0.2146
```

```
Depolar (P) --     0.0000                 0.0000                 0.0002
Depolar (U) --     0.0000                 0.0000                 0.0004
Atom AN      X      Y      Z        X      Y      Z        X      Y      Z
  1  24     0.00   0.14   0.00     0.14   0.00   0.00     0.00   0.00   0.00
  2   6     0.00  -0.32   0.28     0.31   0.00   0.00     0.00  -0.20   0.34
  3   8     0.00  -0.04  -0.19    -0.11   0.00   0.00     0.00   0.07  -0.09
  4   6     0.00  -0.32   0.28     0.31   0.00   0.00     0.00   0.20  -0.34
  5   8     0.00  -0.04  -0.19    -0.11   0.00   0.00     0.00  -0.07   0.09
  6   6    -0.28   0.15  -0.14    -0.16  -0.28  -0.24     0.17   0.10   0.34
  7   8     0.03  -0.09   0.09    -0.06   0.03   0.16    -0.06  -0.03  -0.09
  8   6    -0.28   0.15  -0.14    -0.16  -0.28  -0.24    -0.17  -0.10  -0.34
  9   8     0.03  -0.09   0.09    -0.06   0.03   0.16     0.06   0.03   0.09
 10   6     0.28   0.15  -0.14    -0.16   0.28   0.24    -0.17   0.10   0.34
 11   8    -0.03  -0.09   0.09    -0.06  -0.03  -0.16     0.06  -0.03  -0.09
 12   6     0.28   0.15  -0.14    -0.16   0.28   0.24     0.17  -0.10  -0.34
 13   8    -0.03  -0.09   0.09    -0.06  -0.03  -0.16    -0.06   0.03   0.09
                    22                     23                     24
                    EG                     EG                     A1U
Frequencies --   481.2280               481.2280               506.1805
Red. masses --    12.3056                12.3056                12.3439
Frc consts  --     1.6790                 1.6790                 1.8634
IR Inten    --     0.0000                 0.0000                 0.0000
Raman Activ --     0.8569                 0.8584                 0.0000
Depolar (P) --     0.7500                 0.7500                 0.0000
Depolar (U) --     0.8571                 0.8571                 0.0000
Atom AN      X      Y      Z        X      Y      Z        X      Y      Z
  1  24     0.00   0.00   0.00     0.00   0.00   0.00     0.00   0.00   0.00
  2   6     0.49  -0.03   0.03    -0.09  -0.15   0.19     0.39   0.00   0.00
  3   8    -0.14   0.00  -0.01     0.03   0.00  -0.07    -0.12   0.00   0.00
  4   6    -0.49   0.03  -0.03     0.09   0.15  -0.19     0.39   0.00   0.00
  5   8     0.14   0.00   0.01    -0.03   0.00   0.07    -0.12   0.00   0.00
  6   6    -0.04  -0.22  -0.18    -0.28   0.38  -0.06    -0.20   0.34   0.00
  7   8    -0.02   0.04   0.07     0.07  -0.12   0.02     0.06  -0.10   0.00
  8   6     0.04   0.22   0.18     0.28  -0.38   0.06    -0.20   0.34   0.00
  9   8     0.02  -0.04  -0.07    -0.07   0.12  -0.02     0.06  -0.10   0.00
 10   6     0.06   0.34   0.14     0.28   0.28  -0.12    -0.20  -0.34   0.00
 11   8    -0.04  -0.08  -0.06    -0.05  -0.09   0.05     0.06   0.10   0.00
 12   6    -0.06  -0.34  -0.14    -0.28  -0.28   0.12    -0.20  -0.34   0.00
 13   8     0.04   0.08   0.06     0.05   0.09  -0.05     0.06   0.10   0.00
                    25                     26                     27
                    A2U                    EU                     EU
Frequencies --   526.6732               621.4873               621.4876
Red. masses --    18.5426                17.2378                17.2379
Frc consts  --     3.0304                 3.9228                 3.9228
IR Inten    --    84.6786               126.2013               126.2037
Raman Activ --     0.0000                 0.0000                 0.0000
Depolar (P) --     0.0000                 0.0000                 0.0000
Depolar (U) --     0.0000                 0.0000                 0.0000
Atom AN      X      Y      Z        X      Y      Z        X      Y      Z
  1  24     0.00   0.00   0.39     0.00   0.35   0.00     0.35   0.00   0.00
  2   6     0.00   0.10  -0.33     0.00  -0.15   0.11    -0.48   0.00   0.00
  3   8     0.00  -0.13   0.04     0.00  -0.04  -0.07     0.13   0.00   0.00
  4   6     0.00   0.10  -0.33     0.00  -0.15   0.11    -0.48   0.00   0.00
  5   8     0.00  -0.13   0.04     0.00  -0.04  -0.07     0.13   0.00   0.00
  6   6    -0.09  -0.05  -0.33     0.14  -0.40  -0.05    -0.23   0.14  -0.09
  7   8     0.11   0.07   0.04    -0.07   0.09   0.04     0.01  -0.07   0.06
  8   6    -0.09  -0.05  -0.33     0.14  -0.40  -0.05    -0.23   0.14  -0.09
  9   8     0.11   0.07   0.04    -0.07   0.09   0.04     0.01  -0.07   0.06
 10   6     0.09  -0.05  -0.33    -0.14  -0.40  -0.05    -0.23  -0.14   0.09
 11   8    -0.11   0.07   0.04     0.07   0.09   0.04     0.01   0.07  -0.06
 12   6     0.09  -0.05  -0.33    -0.14  -0.40  -0.05    -0.23  -0.14   0.09
 13   8    -0.11   0.07   0.04     0.07   0.09   0.04     0.01   0.07  -0.06
                    28                     29                     30
                    EG                     EG                     EU
Frequencies --  2073.8421              2073.8421              2081.0179
Red. masses --    13.4118                13.4118                13.4233
Frc consts  --    33.9851                33.9851                34.2500
IR Inten    --     0.0000                 0.0000               999.4578
Raman Activ --   412.8145               412.6342                 0.0000
Depolar (P) --     0.7500                 0.7500                 0.0000
Depolar (U) --     0.8571                 0.8571                 0.0000
Atom AN      X      Y      Z        X      Y      Z        X      Y      Z
  1  24     0.00   0.00   0.00     0.00   0.00   0.00     0.00   0.00   0.00
  2   6     0.00  -0.04  -0.03     0.00  -0.39  -0.24     0.00   0.39   0.24
  3   8     0.00   0.03   0.02     0.00   0.29   0.18     0.00  -0.29  -0.18
  4   6     0.00   0.04   0.03     0.00   0.39   0.24     0.00   0.39   0.24
  5   8     0.00  -0.03  -0.02     0.00  -0.29  -0.18     0.00  -0.29  -0.18
  6   6    -0.31  -0.18   0.22    -0.14  -0.08   0.10     0.17   0.10  -0.12
```

```
  7   8     0.23   0.13  -0.17     0.10   0.06  -0.07    -0.13  -0.07   0.09
  8   6     0.31   0.18  -0.22     0.14   0.08  -0.10     0.17   0.10  -0.12
  9   8    -0.23  -0.13   0.17    -0.10  -0.06   0.07    -0.13  -0.07   0.09
 10   6    -0.27   0.16  -0.20     0.21  -0.11   0.14    -0.17   0.10  -0.12
 11   8     0.20  -0.12   0.15    -0.15   0.09  -0.11     0.13  -0.07   0.09
 12   6     0.27  -0.16   0.20    -0.21   0.11  -0.14    -0.17   0.10  -0.12
 13   8    -0.20   0.12  -0.15     0.15  -0.09   0.11     0.13  -0.07   0.09
                     31                     32                     33
                     EU                     A2U                    A1G
Frequencies --  2081.0179              2083.8290              2157.1878
Red. masses --    13.4233                13.4148                13.3446
Frc consts  --    34.2500                34.3209                36.5875
IR Inten    --   999.4682               816.0331                 0.0000
Raman Activ --     0.0000                 0.0000               208.3320
Depolar (P) --     0.0000                 0.0000                 0.0022
Depolar (U) --     0.0000                 0.0000                 0.0043
Atom AN      X      Y      Z        X      Y      Z        X      Y      Z
  1  24     0.00   0.00   0.00     0.00   0.00   0.00     0.00   0.00   0.00
  2   6     0.00   0.00   0.00     0.00   0.28   0.17     0.00  -0.28  -0.18
  3   8     0.00   0.00   0.00     0.00  -0.21  -0.13     0.00   0.20   0.13
  4   6     0.00   0.00   0.00     0.00   0.28   0.17     0.00   0.28   0.18
  5   8     0.00   0.00   0.00     0.00  -0.21  -0.13     0.00  -0.20  -0.13
  6   6     0.29   0.17  -0.21    -0.24  -0.14   0.17     0.24   0.14  -0.18
  7   8    -0.22  -0.13   0.16     0.18   0.10  -0.13    -0.17  -0.10   0.13
  8   6     0.29   0.17  -0.21    -0.24  -0.14   0.17    -0.24  -0.14   0.18
  9   8    -0.22  -0.13   0.16     0.18   0.10  -0.13     0.17   0.10  -0.13
 10   6     0.30  -0.17   0.21     0.24  -0.14   0.17    -0.24   0.14  -0.18
 11   8    -0.22   0.13  -0.16    -0.18   0.10  -0.13     0.17  -0.10   0.13
 12   6     0.30  -0.17   0.21     0.24  -0.14   0.17     0.24  -0.14   0.18
 13   8    -0.22   0.13  -0.16    -0.18   0.10  -0.13    -0.17   0.10  -0.13
```

Supplementary Data 11 gives full information on the Gaussian calculations of **$[Cr(CO)_5(NO)]^+$**.

Supplementary Data 11. Information on the atomic coordinates and vibrational spectra of $[Cr(CO)_5(NO)]^+$.

```
Harmonic frequencies (cm**-1), IR intensities (KM/Mole), Raman scattering
 activities (A**4/AMU), depolarization ratios for plane and unpolarized
 incident light, reduced masses (AMU), force constants (mDyne/A),
 and normal coordinates:
                      1                      2                      3
                      E                      E                      B2
 Frequencies --    62.7133                62.7133                72.5640
 Red. masses --    15.4012                15.4012                15.4145
 Frc consts  --     0.0357                 0.0357                 0.0478
 IR Inten    --     0.0046                 0.0046                 0.0000
 Raman Activ --     0.0005                 0.0005                 0.0313
 Depolar (P) --     0.7500                 0.7500                 0.7500
 Depolar (U) --     0.8571                 0.8571                 0.8571
 Atom AN      X      Y      Z        X      Y      Z        X      Y      Z
   1  24     0.00  -0.01   0.00    -0.01   0.00   0.00     0.00   0.00   0.00
   2   7     0.00   0.16   0.00     0.16   0.00   0.00     0.00   0.00   0.00
   3   8     0.00   0.41   0.00     0.41   0.00   0.00     0.00   0.00   0.00
   4   6     0.00   0.23   0.00     0.23   0.00   0.00     0.00   0.00   0.00
   5   8     0.00   0.53   0.00     0.53   0.00   0.00     0.00   0.00   0.00
   6   6     0.00  -0.02  -0.03    -0.20   0.00   0.00     0.00   0.01   0.19
   7   8     0.00  -0.02  -0.06    -0.44   0.00   0.00     0.00   0.02   0.46
   8   6     0.00  -0.02   0.03    -0.20   0.00   0.00     0.00  -0.01   0.19
   9   8     0.00  -0.02   0.06    -0.44   0.00   0.00     0.00  -0.02   0.46
  10   6     0.00  -0.20   0.00    -0.02   0.00   0.03     0.01   0.00  -0.19
  11   8     0.00  -0.44   0.00    -0.02   0.00   0.06     0.02   0.00  -0.46
  12   6     0.00  -0.20   0.00    -0.02   0.00  -0.03    -0.01   0.00  -0.19
  13   8     0.00  -0.44   0.00    -0.02   0.00  -0.06    -0.02   0.00  -0.46
                      4                      5                      6
                     B1                      E                      E
 Frequencies --    89.5189                92.9482                92.9482
 Red. masses --    15.5371                15.7449                15.7449
 Frc consts  --     0.0734                 0.0801                 0.0801
 IR Inten    --     0.0000                 0.0788                 0.0788
 Raman Activ --    10.0475                 9.8102                 9.8102
 Depolar (P) --     0.7500                 0.7500                 0.7500
 Depolar (U) --     0.8571                 0.8571                 0.8571
 Atom AN      X      Y      Z        X      Y      Z        X      Y      Z
```

```
  1  24     0.00   0.00   0.00    -0.06   0.00   0.00     0.00  -0.06   0.00
  2   7     0.00   0.00   0.00    -0.18   0.00   0.00     0.00  -0.18   0.00
  3   8     0.00   0.00   0.00    -0.40   0.00   0.00     0.00  -0.40   0.00
  4   6     0.00   0.00   0.00     0.14   0.00   0.00     0.00   0.14   0.00
  5   8     0.00   0.00   0.00     0.50   0.00   0.00     0.00   0.50   0.00
  6   6    -0.17   0.00   0.00     0.03   0.00   0.00     0.00  -0.07  -0.15
  7   8    -0.47   0.00   0.00     0.17   0.00   0.00     0.00  -0.08  -0.45
  8   6     0.17   0.00   0.00     0.03   0.00   0.00     0.00  -0.07   0.15
  9   8     0.47   0.00   0.00     0.17   0.00   0.00     0.00  -0.08   0.45
 10   6     0.00   0.17   0.00    -0.07   0.00   0.15     0.00   0.03   0.00
 11   8     0.00   0.47   0.00    -0.08   0.00   0.45     0.00   0.17   0.00
 12   6     0.00  -0.17   0.00    -0.07   0.00  -0.15     0.00   0.03   0.00
 13   8     0.00  -0.47   0.00    -0.08   0.00  -0.45     0.00   0.17   0.00
                     7                      8                      9
                     E                      E                      A1
Frequencies --   105.1021               105.1021               105.7963
Red. masses --    17.1048                17.1048                17.4420
Frc consts  --     0.1113                 0.1113                 0.1150
IR Inten    --     0.7855                 0.7855                 1.9952
Raman Activ --     1.4061                 1.4061                 0.2794
Depolar (P) --     0.7500                 0.7500                 0.6147
Depolar (U) --     0.8571                 0.8571                 0.7614
Atom AN      X      Y      Z        X      Y      Z        X      Y      Z
  1  24    -0.21   0.02   0.00    -0.02  -0.21   0.00     0.00   0.00   0.23
  2   7     0.06  -0.01   0.00     0.01   0.06   0.00     0.00   0.00   0.24
  3   8     0.53  -0.06   0.00     0.06   0.53   0.00     0.00   0.00   0.24
  4   6    -0.04   0.00   0.00     0.00  -0.04   0.00     0.00   0.00   0.27
  5   8     0.23  -0.02   0.00     0.02   0.23   0.00     0.00   0.00   0.27
  6   6     0.00   0.03   0.00     0.00  -0.25   0.03     0.00  -0.01   0.00
  7   8     0.39   0.03  -0.01     0.04  -0.25   0.14     0.00  -0.03  -0.41
  8   6     0.00   0.03   0.00     0.00  -0.25  -0.03     0.00   0.01   0.00
  9   8     0.39   0.03   0.01     0.04  -0.25  -0.14     0.00   0.03  -0.41
 10   6    -0.25   0.00  -0.03    -0.03   0.00   0.00     0.01   0.00   0.00
 11   8    -0.25  -0.04  -0.14    -0.03   0.39  -0.01     0.03   0.00  -0.41
 12   6    -0.25   0.00   0.03    -0.03   0.00   0.00    -0.01   0.00   0.00
 13   8    -0.25  -0.04   0.14    -0.03   0.39   0.01    -0.03   0.00  -0.41
                    10                     11                     12
                     E                      E                      A2
Frequencies --   336.1628               336.1628               343.0159
Red. masses --    12.9201                12.9201                12.7317
Frc consts  --     0.8602                 0.8602                 0.8826
IR Inten    --     0.1362                 0.1362                 0.0000
Raman Activ --     0.0023                 0.0023                 0.0000
Depolar (P) --     0.7500                 0.7500                 0.0000
Depolar (U) --     0.8571                 0.8571                 0.0000
Atom AN      X      Y      Z        X      Y      Z        X      Y      Z
  1  24     0.01   0.00   0.00     0.00   0.01   0.00     0.00   0.00   0.00
  2   7    -0.27  -0.10   0.00     0.10  -0.27   0.00     0.00   0.00   0.00
  3   8     0.17   0.06   0.00    -0.06   0.17   0.00     0.00   0.00   0.00
  4   6     0.53   0.19   0.00    -0.19   0.53   0.00     0.00   0.00   0.00
  5   8    -0.23  -0.09   0.00     0.09  -0.23   0.00     0.00   0.00   0.00
  6   6     0.00  -0.01   0.16     0.00  -0.03   0.43     0.45   0.00   0.00
  7   8     0.00  -0.02  -0.07     0.00  -0.05  -0.20    -0.21   0.00   0.00
  8   6     0.00  -0.01  -0.16     0.00  -0.03  -0.43    -0.45   0.00   0.00
  9   8     0.00  -0.02   0.07     0.00  -0.05   0.20     0.21   0.00   0.00
 10   6    -0.03   0.00  -0.43     0.01   0.00   0.16     0.00   0.45   0.00
 11   8    -0.05   0.00   0.20     0.02   0.00  -0.07     0.00  -0.21   0.00
 12   6    -0.03   0.00   0.43     0.01   0.00  -0.16     0.00  -0.45   0.00
 13   8    -0.05   0.00  -0.20     0.02   0.00   0.07     0.00   0.21   0.00
                    13                     14                     15
                    A1                     B2                     A1
Frequencies --   351.3566               364.6065               366.9204
Red. masses --    14.9933                14.0709                14.0998
Frc consts  --     1.0905                 1.1021                 1.1184
IR Inten    --    10.0335                 0.0000                 0.2793
Raman Activ --     2.8271                 1.6041                19.5696
Depolar (P) --     0.0639                 0.7500                 0.0320
Depolar (U) --     0.1201                 0.8571                 0.0620
Atom AN      X      Y      Z        X      Y      Z        X      Y      Z
  1  24     0.00   0.00  -0.16     0.00   0.00   0.00     0.00   0.00  -0.01
  2   7     0.00   0.00  -0.19     0.00   0.00   0.00     0.00   0.00   0.00
  3   8     0.00   0.00  -0.21     0.00   0.00   0.00     0.00   0.00  -0.01
  4   6     0.00   0.00   0.62     0.00   0.00   0.00     0.00   0.00  -0.01
  5   8     0.00   0.00   0.64     0.00   0.00   0.00     0.00   0.00  -0.01
  6   6     0.00   0.00  -0.14     0.00   0.35   0.01     0.00  -0.34  -0.02
  7   8     0.00   0.00   0.05     0.00   0.36  -0.02     0.00  -0.36   0.03
  8   6     0.00   0.00  -0.14     0.00  -0.35   0.01     0.00   0.34  -0.02
  9   8     0.00   0.00   0.05     0.00  -0.36  -0.02     0.00   0.36   0.03
```

```
 10   6     0.00   0.00  -0.14     0.35   0.00  -0.01     0.34   0.00  -0.02
 11   8     0.00   0.00   0.05     0.36   0.00   0.02     0.36   0.00   0.03
 12   6     0.00   0.00  -0.14    -0.35   0.00  -0.01    -0.34   0.00  -0.02
 13   8     0.00   0.00   0.05    -0.36   0.00   0.02    -0.36   0.00   0.03
                     16                     17                     18
                      E                      E                      E
Frequencies --   416.8436               416.8436               471.5384
Red. masses --    16.4996                16.4996                12.3459
Frc consts  --     1.6892                 1.6892                 1.6174
IR Inten    --    22.0302                22.0302                 0.1213
Raman Activ --     0.2098                 0.2098                 0.1307
Depolar (P) --     0.7500                 0.7500                 0.7500
Depolar (U) --     0.8571                 0.8571                 0.8571
Atom AN      X      Y      Z        X      Y      Z        X      Y      Z
  1  24    -0.01   0.27   0.00     0.27   0.01   0.00    -0.01   0.00   0.00
  2   7    -0.01   0.29   0.00     0.29   0.01   0.00     0.03   0.00   0.00
  3   8     0.01  -0.14   0.00    -0.14  -0.01   0.00    -0.05   0.00   0.00
  4   6    -0.01   0.19   0.00     0.19   0.01   0.00     0.67  -0.02   0.00
  5   8     0.00  -0.06   0.00    -0.06   0.00   0.00    -0.20   0.00   0.00
  6   6    -0.01  -0.37  -0.06     0.30  -0.02   0.00    -0.29   0.00   0.01
  7   8     0.00  -0.38   0.04    -0.10  -0.02   0.00     0.10   0.00   0.00
  8   6    -0.01  -0.37   0.06     0.30  -0.02   0.00    -0.29   0.00  -0.01
  9   8     0.00  -0.38  -0.04    -0.10  -0.02   0.00     0.10   0.00   0.00
 10   6     0.02   0.30   0.00    -0.37   0.01   0.06    -0.01   0.01   0.39
 11   8     0.02  -0.10   0.00    -0.38   0.00  -0.04     0.01   0.00  -0.11
 12   6     0.02   0.30   0.00    -0.37   0.01  -0.06    -0.01   0.01  -0.39
 13   8     0.02  -0.10   0.00    -0.38   0.00   0.04     0.01   0.00   0.11
                     19                     20                     21
                      E                     B2                     B1
Frequencies --   471.5384               476.6143               503.0172
Red. masses --    12.3459                12.3497                12.2713
Frc consts  --     1.6174                 1.6529                 1.8294
IR Inten    --     0.1213                 0.0000                 0.0000
Raman Activ --     0.1307                 0.0024                 0.0181
Depolar (P) --     0.7500                 0.7500                 0.7500
Depolar (U) --     0.8571                 0.8571                 0.8571
Atom AN      X      Y      Z        X      Y      Z        X      Y      Z
  1  24     0.00  -0.01   0.00     0.00   0.00   0.00     0.00   0.00   0.00
  2   7     0.00   0.03   0.00     0.00   0.00   0.00     0.00   0.00   0.00
  3   8     0.00  -0.05   0.00     0.00   0.00   0.00     0.00   0.00   0.00
  4   6     0.02   0.67   0.00     0.00   0.00   0.00     0.00   0.00   0.00
  5   8     0.00  -0.20   0.00     0.00   0.00   0.00     0.00   0.00   0.00
  6   6    -0.01  -0.01  -0.39     0.00   0.00   0.48    -0.48   0.00   0.00
  7   8     0.00   0.01   0.11     0.00  -0.02  -0.15     0.13   0.00   0.00
  8   6    -0.01  -0.01   0.39     0.00   0.00   0.48     0.48   0.00   0.00
  9   8     0.00   0.01  -0.11     0.00   0.02  -0.15    -0.13   0.00   0.00
 10   6     0.00  -0.29   0.01     0.00   0.00  -0.48     0.00   0.48   0.00
 11   8     0.00   0.10   0.00    -0.02   0.00   0.15     0.00  -0.13   0.00
 12   6     0.00  -0.29  -0.01     0.00   0.00  -0.48     0.00  -0.48   0.00
 13   8     0.00   0.10   0.00     0.02   0.00   0.15     0.00   0.13   0.00
                     22                     23                     24
                     A1                      E                      E
Frequencies --   503.9992               531.9981               531.9981
Red. masses --    13.6139                13.3178                13.3178
Frc consts  --     2.0375                 2.2208                 2.2208
IR Inten    --     2.4584                25.9963                25.9963
Raman Activ --    16.4508                 0.0211                 0.0211
Depolar (P) --     0.1515                 0.7500                 0.7500
Depolar (U) --     0.2631                 0.8571                 0.8571
Atom AN      X      Y      Z        X      Y      Z        X      Y      Z
  1  24     0.00   0.00  -0.05     0.10   0.01   0.00    -0.01   0.10   0.00
  2   7     0.00   0.00   0.44     0.52   0.03   0.00    -0.03   0.52   0.00
  3   8     0.00   0.00   0.49    -0.18  -0.01   0.00     0.01  -0.18   0.00
  4   6     0.00   0.00  -0.02    -0.14  -0.01   0.00     0.01  -0.14   0.00
  5   8     0.00   0.00  -0.03     0.02   0.00   0.00     0.00   0.02   0.00
  6   6     0.00   0.00  -0.36    -0.47   0.00   0.02     0.03  -0.02   0.28
  7   8     0.00   0.02   0.10     0.14   0.00   0.00    -0.01  -0.03  -0.07
  8   6     0.00   0.00  -0.36    -0.47   0.00  -0.02     0.03  -0.02  -0.28
  9   8     0.00  -0.02   0.10     0.14   0.00   0.00    -0.01  -0.03   0.07
 10   6     0.00   0.00  -0.36    -0.02  -0.03  -0.28     0.00  -0.47   0.02
 11   8    -0.02   0.00   0.10    -0.03   0.01   0.07     0.00   0.14   0.00
 12   6     0.00   0.00  -0.36    -0.02  -0.03   0.28     0.00  -0.47  -0.02
 13   8     0.02   0.00   0.10    -0.03   0.01  -0.07     0.00   0.14   0.00
                     25                     26                     27
                     A1                      E                      E
Frequencies --   660.4401               692.9789               692.9789
Red. masses --    21.4994                17.9241                17.9241
Frc consts  --     5.5251                 5.0714                 5.0714
```

```
IR Inten    --   133.2899               98.8835                98.8835
Raman Activ --    18.8371                0.3012                 0.3012
Depolar (P) --     0.2838                0.7500                 0.7500
Depolar (U) --     0.4421                0.8571                 0.8571
Atom AN      X      Y      Z        X      Y      Z        X      Y      Z
  1  24     0.00   0.00   0.46     0.34  -0.01   0.00     0.01   0.34   0.00
  2   7     0.00   0.00  -0.35    -0.69   0.01   0.00    -0.01  -0.69   0.00
  3   8     0.00   0.00  -0.41     0.22   0.00   0.00     0.00   0.22   0.00
  4   6     0.00   0.00  -0.06    -0.32   0.00   0.00     0.00  -0.32   0.00
  5   8     0.00   0.00  -0.06     0.08   0.00   0.00     0.00   0.08   0.00
  6   6     0.00   0.00  -0.34    -0.31   0.00   0.00     0.00  -0.07  -0.11
  7   8     0.00   0.01   0.09     0.08   0.00   0.00     0.00  -0.07   0.03
  8   6     0.00   0.00  -0.34    -0.31   0.00   0.00     0.00  -0.07   0.11
  9   8     0.00  -0.01   0.09     0.08   0.00   0.00     0.00  -0.07  -0.03
 10   6     0.00   0.00  -0.34    -0.07   0.00   0.11     0.00  -0.31   0.00
 11   8    -0.01   0.00   0.09    -0.07   0.00  -0.03     0.00   0.08   0.00
 12   6     0.00   0.00  -0.34    -0.07   0.00  -0.11     0.00  -0.31   0.00
 13   8     0.01   0.00   0.09    -0.07   0.00   0.03     0.00   0.08   0.00
                     28                     29                     30
                     A1                      E                      E
Frequencies --  1899.4584              2085.2401              2085.2401
Red. masses --    14.7517                13.4125                13.4125
Frc consts  --    31.3582                34.3614                34.3614
IR Inten    --  1052.1302              1054.6934              1054.6934
Raman Activ --    38.9177                 4.7039                 4.7039
Depolar (P) --     0.4821                 0.7500                 0.7500
Depolar (U) --     0.6506                 0.8571                 0.8571
Atom AN      X      Y      Z        X      Y      Z        X      Y      Z
  1  24     0.00   0.00  -0.01     0.00   0.00   0.00     0.00   0.00   0.00
  2   7     0.00   0.00   0.77     0.00   0.00   0.00     0.00   0.00   0.00
  3   8     0.00   0.00  -0.61     0.00   0.00   0.00     0.00   0.00   0.00
  4   6     0.00   0.00   0.07     0.00   0.00   0.00     0.00   0.00   0.00
  5   8     0.00   0.00  -0.07     0.00   0.00   0.00     0.00   0.00   0.00
  6   6     0.00  -0.06   0.00     0.00   0.57  -0.02     0.00   0.04   0.00
  7   8     0.00   0.04   0.00     0.00  -0.42   0.01     0.00  -0.03   0.00
  8   6     0.00   0.06   0.00     0.00   0.57   0.02     0.00   0.04   0.00
  9   8     0.00  -0.04   0.00     0.00  -0.42  -0.01     0.00  -0.03   0.00
 10   6     0.06   0.00   0.00    -0.04   0.00   0.00     0.57   0.00   0.02
 11   8    -0.04   0.00   0.00     0.03   0.00   0.00    -0.42   0.00  -0.01
 12   6    -0.06   0.00   0.00    -0.04   0.00   0.00     0.57   0.00  -0.02
 13   8     0.04   0.00   0.00     0.03   0.00   0.00    -0.42   0.00   0.01
                     31                     32                     33
                     B2                     A1                     A1
Frequencies --  2097.1764              2122.7714              2160.8866
Red. masses --    13.3708                13.3830                13.3810
Frc consts  --    34.6478                35.5311                36.8130
IR Inten    --     0.0000               230.2615                91.4583
Raman Activ --   340.0713               217.2408               176.5583
Depolar (P) --     0.7500                 0.7500                 0.0001
Depolar (U) --     0.8571                 0.8571                 0.0002
Atom AN      X      Y      Z        X      Y      Z        X      Y      Z
  1  24     0.00   0.00   0.00     0.00   0.00   0.00     0.00   0.00   0.00
  2   7     0.00   0.00   0.00     0.00   0.00   0.00     0.00   0.00  -0.13
  3   8     0.00   0.00   0.00     0.00   0.00   0.00     0.00   0.00   0.09
  4   6     0.00   0.00   0.00     0.00   0.00   0.66     0.00   0.00   0.45
  5   8     0.00   0.00   0.00     0.00   0.00  -0.49     0.00   0.00  -0.33
  6   6     0.00  -0.41   0.01     0.00   0.23  -0.01     0.00  -0.33   0.01
  7   8     0.00   0.29  -0.01     0.00  -0.16   0.01     0.00   0.24  -0.01
  8   6     0.00   0.41   0.01     0.00  -0.23  -0.01     0.00   0.33   0.01
  9   8     0.00  -0.29  -0.01     0.00   0.16   0.01     0.00  -0.24  -0.01
 10   6    -0.41   0.00  -0.01    -0.23   0.00  -0.01     0.33   0.00   0.01
 11   8     0.29   0.00   0.01     0.16   0.00   0.01    -0.24   0.00  -0.01
 12   6     0.41   0.00  -0.01     0.23   0.00  -0.01    -0.33   0.00   0.01
 13   8    -0.29   0.00   0.01    -0.16   0.00   0.01     0.24   0.00  -0.01
```

1. A. Reisinger. *Chemistry with weakly coordinating anions. Strong Brønsted acids and group 11 metal complexes.* (Dissertation, University of Freiburg (Breisgau), 2006, Cuvillier, Göttingen, 2007).
2. Engesser, T. A. *et al.* Homoleptic Gold Acetonitrile Complexes with Medium to Very Weakly Coordinating Counterions. Effect on Aurophilicity? *Chem. Eur. J.* **22,** 15085–15094; 10.1002/chem.201602797 (2016).
3. Rohde, M., Müller, L. O., Himmel, D., Scherer, H. & Krossing, I. A Janus-headed Lewis superacid. Simple access to, and first application of $Me_3Si\text{-}F\text{-}Al(OR^F)_3$. *Chem. Eur. J.* **20,** 1218–1222; 10.1002/chem.201303671 (2014).
4. Saielli, G., Bini, R. & Bagno, A. Computational ^{19}F NMR. 2. Organic compounds. *RSC Adv.* **4,** 41605–41611; 10.1039/C4RA08290G (2014).
5. Fulmer, G. R. *et al.* NMR Chemical Shifts of Trace Impurities. Common Laboratory Solvents, Organics, and Gases in Deuterated Solvents Relevant to the Organometallic Chemist. *Organometallics* **29,** 2176–2179; 10.1021/om100106e (2010).
6. Wray, V., Ernst, L. & Lustig, E. The complete ^{13}C ^{19}F, and ^{1}H spectral analysis of the fluorobenzenes $C_6H_nF_{6-n}$. III. The remaining members of the series; INDO MO calculations of JFH, JFF, JCH, and JCF. *Journal of Magnetic Resonance (1969)* **27,** 1–21; 10.1016/0022-2364(77)90188-3 (1977).
7. Bain, G. A. & Berry, J. F. Diamagnetic Corrections and Pascal's Constants. *J. Chem. Educ.* **85,** 532; 10.1021/ed085p532 (2008).
8. Grant, D. H. Paramagnetic Susceptibility by NMR. The "Solvent Correction" Reexamined. *J. Chem. Educ.* **72,** 39; 10.1021/ed072p39 (1995).
9. Evans, D. F. The determination of the paramagnetic susceptibility of substances in solution by nuclear magnetic resonance. *J. Chem. Soc.*, 2003; 10.1039/jr9590002003 (1959).
10. SAINT V8.37A, Bruker AXS, Madison, Wisconsin, USA (2015).
11. Krause, L., Herbst-Irmer, R., Sheldrick, G. M. & Stalke, D. Comparison of silver and molybdenum microfocus X-ray sources for single-crystal structure determination. *J. Appl. Crystallogr.* **48,** 3–10; 10.1107/S1600576714022985 (2015).
12. Sheldrick, G. M. A short history of SHELX. *Acta Cryst. A* **64,** 112–122; 10.1107/S0108767307043930 (2008).
13. Sheldrick, G. M. SHELXT - integrated space-group and crystal-structure determination. *Acta Cryst. A* **71,** 3–8; 10.1107/S2053273314026370 (2015).
14. Sheldrick, G. M. Crystal structure refinement with SHELXL. *Acta Cryst. C* **71,** 3–8; 10.1107/S2053229614024218 (2015).

15. Hübschle, C. B., Sheldrick, G. M. & Dittrich, B. ShelXle. A Qt graphical user interface for SHELXL. *J. Appl. Crystallogr.* **44,** 1281–1284; 10.1107/S0021889811043202 (2011).
16. Kratzert, D., Holstein, J. J. & Krossing, I. DSR. Enhanced modelling and refinement of disordered structures with SHELXL. *J. Appl. Crystallogr.* **48,** 933–938; 10.1107/S1600576715005580 (2015).
17. Macrae, C. F. *et al.* Mercury CSD 2.0 – new features for the visualization and investigation of crystal structures. *J. Appl. Crystallogr.* **41,** 466–470; 10.1107/S0021889807067908 (2008).
18. Dolomanov, O. V., Bourhis, L. J., Gildea, R. J., Howard, J. A. K. & Puschmann, H. OLEX2. A complete structure solution, refinement and analysis program. *J. Appl. Crystallogr.* **42,** 339–341; 10.1107/S0021889808042726 (2009).
19. Turner M.J., e. a. CrystalExplorer17, University of Western Australia. *http://hirshfeldsurface.net* (2017).
20. MATLAB R2017a (9.2.0). *The MathWorks* (2017).
21. Stoll, S. & Schweiger, A. EasySpin, a comprehensive software package for spectral simulation and analysis in EPR. *J. Magn. Reson.* **178,** 42–55; 10.1016/j.jmr.2005.08.013 (2006).
22. Treutler, O. & Ahlrichs, R. Efficient molecular numerical integration schemes. *J. Chem. Phys.* **102,** 346–354; 10.1063/1.469408 (1995).
23. Ahlrichs, R., Bär, M., Häser, M., Horn, H. & Kölmel, C. Electronic structure calculations on workstation computers. The program system turbomole. *Chem. Phys. Lett.* **162,** 165–169; 10.1016/0009-2614(89)85118-8 (1989).
24. Perdew, J. P. Erratum. Density-functional approximation for the correlation energy of the inhomogeneous electron gas. *Phys. Rev. B* **34,** 7406; 10.1103/PhysRevB.34.7406 (1986).
25. Perdew, J. P. Density-functional approximation for the correlation energy of the inhomogeneous electron gas. *Phys. Rev. B* **33,** 8822–8824; 10.1103/PhysRevB.33.8822 (1986).
26. Ahlrichs, R. Efficient evaluation of three-center two-electron integrals over Gaussian functions. *Phys. Chem. Chem. Phys.* **6,** 5119; 10.1039/b413539c (2004).
27. Sierka, M., Hogekamp, A. & Ahlrichs, R. Fast evaluation of the Coulomb potential for electron densities using multipole accelerated resolution of identity approximation. *J. Chem. Phys.* **118,** 9136–9148; 10.1063/1.1567253 (2003).

28. Weigend, F. & Ahlrichs, R. Balanced basis sets of split valence, triple zeta valence and quadruple zeta valence quality for H to Rn. Design and assessment of accuracy. *Phys. Chem. Chem. Phys.* **7,** 3297–3305; 10.1039/b508541a (2005).
29. Grimme, S., Ehrlich, S. & Goerigk, L. Effect of the damping function in dispersion corrected density functional theory. *J. Comput. Chem.* **32,** 1456–1465; 10.1002/jcc.21759 (2011).
30. Grimme, S., Antony, J., Ehrlich, S. & Krieg, H. A consistent and accurate ab initio parametrization of density functional dispersion correction (DFT-D) for the 94 elements H-Pu. *J. Chem. Phys.* **132,** 154104; 10.1063/1.3382344 (2010).
31. Deglmann, P., Furche, F. & Ahlrichs, R. An efficient implementation of second analytical derivatives for density functional methods. *Chem. Phys. Lett.* **362,** 511–518; 10.1016/S0009-2614(02)01084-9 (2002).
32. M. J. Frisch, G. W. Trucks, H. B. Schlegel, G. E. Scuseria, M. A. Robb, J. R. Cheeseman, J. A. Montgomery, Jr., T. Vreven, K. N. Kudin, J. C. Burant, J. M. Millam, S. S. Iyengar, J. Tomasi, V. Barone, B. Mennucci, M. Cossi, G. Scalmani, N. Rega, G. A. Petersson, H. Nakatsuji, M. Hada, M. Ehara, K. Toyota, R. Fukuda, J. Hasegawa, M. Ishida, T. Nakajima, Y. Honda, O. Kitao, H. Nakai, M. Klene, X. Li, J. E. Knox, H. P. Hratchian, J. B. Cross, V. Bakken, C. Adamo, J. Jaramillo, R. Gomperts, R. E. Stratmann, O. Yazyev, A. J. Austin, R. Cammi, C. Pomelli, J. W. Ochterski, P. Y. Ayala, K. Morokuma, G. A. Voth, P. Salvador, J. J. Dannenberg, V. G. Zakrzewski, S. Dapprich, A. D. Daniels, M. C. Strain, O. Farkas, D. K. Malick, A. D. Rabuck, K. Raghavachari, J. B. Foresman, J. V. Ortiz, Q. Cui, A. G. Baboul, S. Clifford, J. Cioslowski, B. B. Stefanov, G. Liu, A. Liashenko, P. Piskorz, I. Komaromi, R. L. Martin, D. J. Fox, T. Keith, M. A. Al-Laham, C. Y. Peng, A. Nanayakkara, M. Challacombe, P. M. W. Gill, B. Johnson, W. Chen, M. W. Wong, C. Gonzalez, and J. A. Pople. Gaussian 03, Revision D.01. *Gaussian, Inc.* (2004).
33. Staroverov, V. N., Scuseria, G. E., Tao, J. & Perdew, J. P. Comparative assessment of a new nonempirical density functional. Molecules and hydrogen-bonded complexes. *J. Chem. Phys.* **119,** 12129–12137; 10.1063/1.1626543 (2003).
34. Tao, J., Perdew, J. P., Staroverov, V. N. & Scuseria, G. E. Climbing the density functional ladder. Nonempirical meta-generalized gradient approximation designed for molecules and solids. *Phys. Rev. Lett.* **91,** 146401; 10.1103/PhysRevLett.91.146401 (2003).
35. Liakos, D. G., Sparta, M., Kesharwani, M. K., Martin, J. M. L. & Neese, F. Exploring the Accuracy Limits of Local Pair Natural Orbital Coupled-Cluster Theory. *J. Chem. Theory Comput.* **11,** 1525–1539; 10.1021/ct501129s (2015).

36. Riplinger, C., Sandhoefer, B., Hansen, A. & Neese, F. Natural triple excitations in local coupled cluster calculations with pair natural orbitals. *J. Chem. Phys.* **139,** 134101; 10.1063/1.4821834 (2013).
37. Neese, F. The ORCA program system. *WIREs Comput. Mol. Sci. (Wiley Interdisciplinary Reviews: Computational Molecular Science)* **2,** 73–78; 10.1002/wcms.81 (2012).
38. Neese, F. An improvement of the resolution of the identity approximation for the formation of the Coulomb matrix. *J. Comput. Chem.* **24,** 1740–1747; 10.1002/jcc.10318 (2003).
39. Izsák, R. & Neese, F. An overlap fitted chain of spheres exchange method. *J. Chem. Phys.* **135,** 144105; 10.1063/1.3646921 (2011).
40. Izsák, R. & Neese, F. Speeding up spin-component-scaled third-order pertubation theory with the chain of spheres approximation. The COSX-SCS-MP3 method. *Mol. Phys.* **111,** 1190–1195; 10.1080/00268976.2013.796071 (2013).
41. Helgaker, T., Jørgensen, P. & Olsen, J. *Molecular electronic-structure theory* (Wiley, Chichester, New York, 2012).
42. Balabanov, N. B. & Peterson, K. A. Systematically convergent basis sets for transition metals. I. All-electron correlation consistent basis sets for the 3d elements Sc-Zn. *The Journal of Chemical Physics* **123,** 64107; 10.1063/1.1998907 (2005).
43. Dunning, T. H. Gaussian basis sets for use in correlated molecular calculations. I. The atoms boron through neon and hydrogen. *The Journal of Chemical Physics* **90,** 1007–1023; 10.1063/1.456153 (1989).
44. Angeli, C., Cimiraglia, R. & Malrieu, J.-P. n -electron valence state perturbation theory. A spinless formulation and an efficient implementation of the strongly contracted and of the partially contracted variants. *J. Chem. Phys.* **117,** 9138–9153; 10.1063/1.1515317 (2002).
45. Angeli, C., Cimiraglia, R., Evangelisti, S., Leininger, T. & Malrieu, J.-P. Introduction of n -electron valence states for multireference perturbation theory. *J. Chem. Phys.* **114,** 10252–10264; 10.1063/1.1361246 (2001).
46. Miller, J. S. & Drillon, M. eds. *Magnetism. Molecules to Materials V* (Wiley-VCH, Weinheim, 2006).
47. Raabe, I. *et al.* Tetraalkylammonium salts of weakly coordinating aluminates. Ionic liquids, materials for electrochemical applications and useful compounds for anion investigation. *Chem. Eur. J.* **15,** 1966–1976; 10.1002/chem.200800417 (2009).
48. Lehner, A. J., Trapp, N., Scherer, H. & Krossing, I. CCl_3^+ and CBr_3^+ salts with the $[Al(OR^F)_4]^-$ and $[(^FRO)_3Al\text{-}F\text{-}Al(OR^F)_3]^-$ anions ($R^F = C(CF_3)_3$). *Dalton Trans.* **40,** 1448–1452; 10.1039/c0dt01076f (2011).

49. Bernhardt, E. *et al.* D_{3d} Ground-State Structure of $V(CO)_6$. A Combined Matrix Isolation and ab Initio Study of the Jahn−Teller Effect. *J. Phys. Chem. A* **107,** 859–868; 10.1021/jp021835u (2003).
50. Zhao, Y. & Truhlar, D. G. A new local density functional for main-group thermochemistry, transition metal bonding, thermochemical kinetics, and noncovalent interactions. *J. Chem. Phys.* **125,** 194101; 10.1063/1.2370993 (2006).
51. Becke, A. D. Density-functional thermochemistry. III. The role of exact exchange. *J. Chem. Phys.* **98,** 5648–5652; 10.1063/1.464913 (1993).
52. van Lenthe, E., Baerends, E. J. & Snijders, J. G. Relativistic total energy using regular approximations. *The Journal of Chemical Physics* **101,** 9783–9792; 10.1063/1.467943 (1994).
53. Visscher, L. & Dyall, K. G. Dirac–Fock Atomic Electronic Structure Calculations Using Different Nuclear Charge Distributions. *Atom. Data Nucl. Data* **67,** 207–224; 10.1006/adnd.1997.0751 (1997).
54. Gabbaï, F. P. *et al.* An Editorial About Elemental Analysis. *Organometallics* **35,** 3255–3256; 10.1021/acs.organomet.6b00720 (2016).
55. Marc, A., Compa, R., Rubio, R. & Casals, I. Assessment of Additives for Nitrogen, Carbon, Hydrogen and Sulfur Determination by Organic Elemental Analysis. *Microchimica Acta* **142,** 13–19; 10.1007/s00604-002-0956-y (2003).
56. Toby, B. H. EXPGUI , a graphical user interface for GSAS. *J Appl Crystallogr* **34,** 210–213; 10.1107/S0021889801002242 (2001).
57. R. B. von Dreele, A. C. Larson. General Structure Analysis System (GSAS). *Los Alamos National Laboratory Report LAUR 86-748* (2000).
58. Lee, C., Yang, W. & Parr, R. G. Development of the Colle-Salvetti correlation-energy formula into a functional of the electron density. *Phys. Rev. B* **37,** 785–789; 10.1103/PhysRevB.37.785 (1988).
59. Becke, A. D. BP86 Funktional. *Phys. Rev. A* **38,** 3098–3100 (1988).
60. Schäfer, A., Horn, H. & Ahlrichs, R. SVP (teilweise auch def2-SVP). *J. Chem. Phys.* **97,** 2571–2577; 10.1063/1.463096 (1992).
61. Weigend, F., Köhn, A. & Hättig, C. Efficient use of the correlation consistent basis sets in resolution of the identity MP2 calculations. *J. Chem. Phys.* **116,** 3175–3183; 10.1063/1.1445115 (2002).

62. Eichkorn, K., Weigend, F., Treutler, O. & Ahlrichs, R. Auxiliary basis sets for main row atoms and transition metals and their use to approximate Coulomb potentials. *Theor. Chim. Acta* **97,** 119–124; 10.1007/s002140050244 (1997).
63. Eichkorn, K., Treutler, O., Öhm, H., Häser, M. & Ahlrichs, R. Auxiliary basis sets to approximate Coulomb potentials (Chem. Phys. Letters 240 (1995) 283-290). *Chem. Phys. Lett.* **242,** 652–660; 10.1016/0009-2614(95)00838-U (1995).
64. Eichkorn, K., Treutler, O., Öhm, H., Häser, M. & Ahlrichs, R. Auxiliary basis sets to approximate Coulomb potentials. *Chem. Phys. Lett.* **240,** 283–290; 10.1016/0009-2614(95)00621-A (1995).
65. Weigend, F. Accurate Coulomb-fitting basis sets for H to Rn. *Phys. Chem. Chem. Phys.* **8,** 1057–1065; 10.1039/b515623h (2006).
66. Klamt, A., Jonas, V., Bürger, T. & Lohrenz, J. C. W. Refinement and Parametrization of COSMO-RS. *J. Phys. Chem. A* **102,** 5074–5085; 10.1021/jp980017s (1998).
67. Klamt, A. Conductor-like Screening Model for Real Solvents. A New Approach to the Quantitative Calculation of Solvation Phenomena. *J. Phys. Chem.* **99,** 2224–2235; 10.1021/j100007a062 (1995).
68. Eckert, F. & Klamt, A. *COSMOTherm* (COSMOtherm, C3.0, release 1501, COSMOlogic GmbH & Co KG, http://www.cosmologic.de, 2015).
69. Eckert, F. & Klamt, A. Fast solvent screening via quantum chemistry. COSMO-RS approach. *AIChE J.* **48,** 369–385; 10.1002/aic.690480220 (2002).
